# High-throughput sequencing identifies STAT3 as the DNA-associated factor for p53-NF-κB-complex-dependent gene expression in human heart failure

**DOI:** 10.1186/gm158

**Published:** 2010-06-14

**Authors:** Mun-Kit Choy, Mehregan Movassagh, Lee Siggens, Ana Vujic, Martin Goddard, Ana Sánchez, Neil Perkins, Nichola Figg, Martin Bennett, Jason Carroll, Roger Foo

**Affiliations:** 1 Department of Medicine, University of Cambridge, Addenbrooke's Centre for Clinical Investigation, Hills Road, Cambridge, CB2 0QQ, UK; 2 Department of Histopathology, Papworth Hospital, Papworth Everard, Cambridge, CB23 3RE, UK; 3 Department of Cellular and Molecular Medicine, University of Bristol, School of Medical Sciences, University Walk, Bristol, BS8 1TD, UK; 4 Cancer Research UK, Cambridge Research Institute, Li Ka Shing Centre, Robinson Way, Cambridge CB2 0RE, UK

## Abstract

**Background:**

Genome-wide maps of DNA regulatory elements and their interaction with transcription factors may form a framework for understanding regulatory circuits and gene expression control in human disease, but how these networks, comprising transcription factors and DNA-binding proteins, form complexes, interact with DNA and modulate gene expression remains largely unknown.

**Methods:**

Using microRNA-21 (mir-21), which is an example of genes that are regulated in heart failure, we performed chromatin immunoprecipitation (ChIP) assays to determine the occupancy of transcription factors at this genetic locus. Tissue ChIP was further performed using human hearts and genome-wide occupancies of these transcription factors were analyzed by high-throughput sequencing.

**Results:**

We show that the transcription factor p53 piggy-backs onto NF-κB/RELA and utilizes the κB-motif at a *cis*-regulatory region to control mir-21 expression. p53 behaves as a co-factor in this complex because despite a mutation in its DNA binding domain, mutant p53 was still capable of binding RELA and the *cis*-element, and inducing mir-21 expression. In dilated human hearts where mir-21 upregulation was previously demonstrated, the p53-RELA complex was also associated with this *cis*-element. Using high-throughput sequencing, we analyzed genome-wide binding sites for the p53-RELA complex in diseased and control human hearts and found a significant overrepresentation of the STAT3 motif. We further determined that STAT3 was necessary for the p53-RELA complex to associate with this *cis*-element and for mir-21 expression.

**Conclusions:**

Our results uncover a mechanism by which transcription factors cooperate in a multi-molecular complex at a *cis*-regulatory element to control gene expression.

## Background

Gene transcription is modulated by the dynamic interaction between DNA and protein complexes. Genome-wide maps of these interactions are now generated using a combination of chromatin immunoprecipitation (ChIP) and powerful tools such as high-throughput sequencing (ChIP-seq), and they provide a framework for interpreting the genome in different contexts, including in embryonic stem cells and oncogenesis [1-3]. Genome-wide maps for these transcription factors also show that much remains to be discovered to complete our understanding of transcriptional regulatory networks: empirical binding sites for a transcription factor often lack the expected consensus motif, reflecting that different mechanisms exist for transcription factor recruitment, with some likely to involve indirect binding through components of a multi-molecular transcription complex [3]. Moreover, the numerous means by which a factor is recruited to the genome may also allow it to participate in multiple signaling pathways. In fact, Chen *et al. *[1] observed that, in embryonic stem cells, a significant subset of transcription factor binding regions is extensively co-occupied by several different transcription factors to form multiple transcription-factor binding loci. Our work here proposes a simple analytical model that is potentially representative of multiple transcription-factor binding loci in human disease. We have dissected the behavior and functional roles of the different components of a multi-molecular transcription complex, capitalizing on a regulatory pathway that controls mir-21 expression in human heart disease.

MicroRNA genes transcribe short (approximately 22 nucleotide) non-coding RNAs (miRNAs) that direct mRNA degradation or disrupt mRNA translation in a sequence-dependent manner. Like protein-coding mRNA, miRNAs are initially generated by RNA polymerase II as long primary transcripts before being processed to mature miRNA [4]. Based on the genome-wide chromatin marks of transcription start sites and transcriptional elongation, promoters of human miRNAs were recently identified [5], but the diverse expression profiles of miRNAs indicate that miRNA expression must be under elaborate control during development and disease states, similar to other genes that are transcribed by RNA polymerase II. A consistent pattern of miRNA expression is found in failing hearts [6,7] and the roles of key miRNAs in heart failure development and progression have been studied [8,9].

We and others [10,11] found that the transcription factor p53 is highly activated and accumulates in hypoxic hearts in response to stress. Experimentally, p53 regulates at least 34 different miRNAs in oncogenesis (for example, mir-34; reviewed in [12]). We therefore investigated the possibility that p53 regulates some part of the miRNA expression in failing hearts.

## Materials and methods

### Ethics statement

Human left ventricular tissue was collected with a protocol approved by the Papworth (Cambridge) Hospital Tissue Bank review board and the Cambridgeshire Research Ethics Committee (UK). Written consent was obtained from every individual according to the Papworth Tissue Bank protocol.

### Cell isolation, culture and human cardiac tissue

Rat neonatal cardiac fibroblasts were isolated from 0- to 5-day-old Wistar or Sprague-Dawley rats by an enzymatic isolation method as described before [13] and in accordance with UK Home Office regulations. Primary cardiac fibroblasts, immortalized *RelA*^-/- ^mouse embryo fibroblast (MEF) cells (from Professor R Hay, University of Dundee), *p53*^*-/- *^MEF cells (from Dr G Lozano, MD Anderson Cancer Centre) and Soas2 osteosarcoma cells were cultured in Dulbecco's modified Eagle's medium containing 10% fetal calf serum at 5% CO_2 _and 37°C, and maintained at 60 to 80% confluency. *Stat3*^*-/- *^MEF cells and *Stat3*^*-/- *^MEF cells re-constituted with wild-type *Stat3 *were obtained from Dr David Levy (NYU, School of Medicine). Where indicated, cells were treated with 10 μM doxorubicin (Sigma Dorset, UK) for 2 h before being allowed to grow for another 24 h, 200 μM deferroxamine (DFX; Sigma) for 24 h, with or without 1 μM NF-κB activation inhibitor (NFI; 6-amino-4-(4-phenoxyphenylethylamino) quinazoline; Calbiochem, Nottingham, UK) for 24 h, and with or without 100 μM STAT3 inhibitor (S3I-201, Calbiochem) for 24 h. Hypoxia treatment was performed in an Invivo_2 _400 Hypoxia Workstation (Ruskinn, Bridgend, UK) at 1% O_2_, 5% CO_2 _and 37°C for 48 h.

Cardiac left ventricular tissues were obtained from patients undergoing cardiac transplantation for end-stage dilated cardiomyopathy (three males and one female aged 49 to 60 years). Normal human ventricular tissues were from four healthy male individuals involved in road traffic accidents (aged 41 to 52 years). At the time of transplantation or donor harvest, whole hearts were removed after preservation and transported in cold cardioplegic solution (cardioplegia formula and Hartmann's solution) similar to the procedure described before at Imperial College, London [14]. Following analysis by a cardiovascular pathologist (MG), left ventricular segments were cut and stored immediately in RNAlater (Ambion, Applied Biosystems, Warrington, UK). Individual patient details are listed in Additional file 1.

### miRNA quantitative PCR

Total RNA from cells was extracted using mirVana miRNA Isolation Kit (Ambion). Reverse transcription and quantitative PCR (qPCR) amplification of cDNA of mature miRNAs were performed using primer sets and protocols obtained from Applied Biosystems (TaqMan MicroRNA Assay, Warrington, UK), and Rotor-Gene 6000 qPCR machine from Corbett (Qiagen, Crawley, UK). PCR signals from cDNA of miRNAs were standardized with signals from amplification of cDNA of 18s rRNA, which was reverse transcribed using SuperScript III First-Strand Synthesis System for RT-PCR (Invitrogen, Paisley, UK), using a set of primers/probe (18s forward primer, 5'-CGGCTACCACATCCAAGGAA-3'; reverse primer, 5'-AGCTGGAATTACCGCGGC-3'; probe, 5'-FAM-TGCTGGCACCAGACTTGCCCTC-BHQ1-3') and the protocol for TaqMan Gene Expression Assays (Applied Biosystems).

### Immunoblot analysis

Whole-cell lysates were prepared by scraping cells into a lysis buffer (50 mM Tris pH 8.0, 150 mM NaCl, 0.02% sodium azide, 1% Nonidet P-40, 1x Roche Complete Mini Protease Inhibitor Cocktail, Burgess Hill, UK). To prepare nuclear lysates, cell membranes/cytoplasms of harvested cells were lysed in an ice-cold cytoplasmic lysis buffer (0.33 M sucrose, 10 mM HEPES pH 7.4, 1 mM MgCl_2_, 0.1% Triton-X 100, 1x Roche Complete Mini Protease Inhibitor Cocktail) and the nuclei were washed with the same buffer before being lysed with a buffer containing 0.45 M NaCl, 10 mM HEPES pH7.4 and 1x Roche Complete Mini Protease Inhibitor Cocktail. Protein concentration was determined using a BCA Protein Assay Kit (Pierce, Thermo Scientific, Cramlington, UK). Equal protein amounts were resolved by SDS-PAGE, transferred to a polyvinylidene fluoride membrane and incubated with primary antibodies against the following proteins: p53 (1C12; Cell Signaling, New England Biolabs, Hitchin, UK), NF-κB (p65 SC-109 and SC-372; Santa Cruz, Heidelberg, Germany), STAT3 (Cell Signaling), SF2 (loading control; Zymed, Invitrogen, Paisley, UK) and RhoGDI (loading control; A-20; Santa Cruz). Goat anti-mouse and anti-rabbit (Jackson ImmunoResearch, Newmarket, UK) were used as secondary antibodies. The membrane was incubated with SuperSignal West Pico Chemiluminescent Substrate, SuperSignal West Dura Extended Duration Substrate, SuperSignal West Femto Maximum Sensitivity Chemiluminescent Substrate (Pierce) or ECL Advance Western Blotting Detection Kit (GE Healthcare, Chalfont St Giles, UK) before being exposed to an X-ray film.

### Immunoprecipitation

Cells were scraped into the lysis buffer and protein concentration was determined as described above. Protein A beads (Sigma) were prewashed twice with phosphate-buffered saline and suspended in the lysis buffer. Total protein lysate (500 μg) was incubated with 30 μl of the protein A beads for an hour at 4°C and centrifuged. The supernatant was then removed to a fresh tube and incubated overnight at 4°C with primary antibodies and IgG (mouse/rabbit Fc fraction; Jackson ImmunoResearch) as indicated. The next day, 30 μl of the protein A beads were added and incubated for an hour at 4°C. Following the incubation, beads were washed three times with phosphate-buffered saline. Proteins were eluted with SDS loading buffer (100 mM Tris pH 6.8, 4% SDS, 0.2% bromophenol blue, 20% glycerol, 10% β-mercaptoethanol), resolved by SDS-PAGE, and transferred to a membrane for immunoblot analysis as described above.

### Chromatin immunoprecipitation and sequential ChIP

Chromatin immunoprecipitation (ChIP) was performed using a ChIP Assay Kit (Upstate, Millipore, Watford, UK), primary antibodies raised against p53 (CM5, Vector Laboratories for rat, Orton Southgate, UK); DO-1, sc-126 Santa Cruz for human), NF-κB (C-20/sc-372, Santa Cruz) and histone H3 (tri methyl K4; ab8580, Abcam, Cambridge, UK), and IgG. For tissue ChIP, the heart tissues were finely chopped, cross-linked and homogenized prior to the procedure. Cross-linked chromatin was fragmentized to 1 kb by sonication. Regions of interest were amplified from the immunoprecipitated DNA by qPCR using SYBR GreenER qPCR SuperMix Universal (Invitrogen). The primers for the rat 32280-7 site were 5'-TAGGCAAGCCTCAAGCTCTC-3' and 5'-TCGTTTGGCATAGCTTTGTG-3', and primers for the human GIS site were 5'-TGCAGAAATTGGAGTGGATG-3' and 5'-TTGCAAGTTTGCTGCTGAAC-3' (95°C for 10 minutes followed by 40 to 50 cycles of 94°C for 15 s, 59°C for 20 s, 72°C for 30 s and 76°C for 5 s (signals acquired)), whereas the primers for the promoter of mir-21 were 5'-TACAAACTGGGGAGCTTGGT-3' and 5'-AACCCCTGCGTCATCCTTAT-3' (95°C for 10 minutes followed by 40 to 50 cycles of 94°C for 15 s, 59°C for 20 s and 72°C for 30 s (signals acquired)). PCR signals were standardized with signals from amplification of 18s rRNA genes with the same primers/probe and protocol as described above. For sequential ChIP (re-ChIP) assays, complexes from the primary ChIP were eluted twice with 10 mM dithiothreitol for 20 minutes at 37°C, diluted 10 times with re-ChIP buffer (20 mM Tris-HCl pH 8.1, 0.1% Triton X-100, 2 mM EDTA, 150 mM NaCl) followed by re-immunoprecipitation with the indicated second primary antibody, and then again subjected to the ChIP procedure.

### Biotinylated oligonucleotide precipitation assay

Cells were lysed by sonication in HKMG buffer (10 mM HEPES pH 7.9, 100 mM KCl, 5 mM MgCl_2_, 10% glycerol, 1 mM dithiothreitol, 0.5% of NP-40 and 1x Roche Complete Mini Protease Inhibitor Cocktail). Cell extracts were pre-cleared with Promega Magnesphere beads for 1 h at 4°C, then incubated with 10 μg of biotinylated double-stranded oligonucleotides pre-bound to the beads for 16 h at 4°C (5' biotinylated 5'-GGCTCTCACCAGGAAGGAAGATCCCCATTTCCAACCTGTAC-3' (Promega, Southampton, UK)). DNA-bound proteins were eluted with SDS loading buffer, separated by SDS-PAGE, and identified by immunoblotting as described above.

### Cell transfection, recombinant proteins and luciferase activity assay

Cells were transfected with plasmid DNA using Superfect Transfection Reagent (Qiagen, Crawley, UK) following the manufacturer's protocol or by electroporation using Amaxa Nucleofector according to manufacturer's instructions (Wokingham, UK). *RelA*^-/- ^MEF cells were reconstituted with a full length human *RELA *cDNA using the pHR-SIN-CSGW retroviral vector. Recombinant RELA and p53 proteins were produced in BL21 *Escherichia coli *as glutathione-S-transferase (GST)- and His-fusions, respectively, and purified on Glutathione Sepharose column (Amersham Biosciences, GE Healthcare, Chalfont St Giles, UK) or Ni-nitrilotriacetic acid agarose (Invitrogen). For luciferase assays, *p53*^*-/- *^MEF cells in 6-well plates were transfected with 2.1 μg of total plasmid DNA containing human *p53 *(0.5 μg), *p53R175H *(0.5 μg), *NF-κB *(0.5 μg; p65-EYFP from Dr M Schaaf, Leiden University, The Netherlands) and/or empty vector (0.5 to 1 μg; pcDNA; Invitrogen) together with respective reporter plasmids: GIS-luciferase (GIS is a highly conserved putative p53 binding site approximately 1.1 kb upstream of mir-21), GIS-luciferase with mutation or deletion, or miPPR21-luciferase (1 μg) with phRG-TK (0.1 μg; Transfection control; Promega). Cell lysates harvested 24 h later were assayed for firefly and renilla luciferase activities by using a Dual-Glo Luciferase Reporter Assay (Promega).

### Immunohistochemistry

Paraffin sections were prepared from human left ventricular tissues that were previously collected and stored in RNAlater (Ambion). Tissues were perfused *in situ *with 10% neutral-buffered formalin, cut in 5-μm sections, and stained using antibodies NF-κB (SC-372, Santa Cruz), phospho-p53^ser15 ^and phospho-p53^ser20 ^(Cell Signaling), as previously described [15].

### ChIP-Seq

High-throughput sequencing of the sequential-ChIP fragments from human hearts was performed using Illumina Genome Analyser by GeneService, UK following the manufacturer's protocols. Two flowcell lanes were used for sequencing of each pooled sample (control versus disease) on the Genome Analyzer II. The Genome Analyzer was run for 36 cycles. The reference genome used for sequence alignment was the human build 36.1 finished human genome assembly (hg18, March 2006). Images from the Genome Analyzer were analyzed with the Genome Analyzer pipeline software (version 1.3, Illumina software) for base calling and sequence alignment to the reference human genome. Sequence alignment stage was performed using the ELAND algorithm with the 'ELAND extended' option to enable better handling of reads >32 bp. The length and abundance of ChIP fragments were modeled from sequencing reads using Model-Based Analysis of ChIP-Seq (MACS) with model fold at 100 and *P*-value cutoff at 1 × 10^-3 ^[16]. Motif analysis and searches were performed using the Cis-regulatory Element Annotation System (CEAS) [17] and FIMO [18]. To identify NF-κB motifs, matrices used in the FIMO search were M00052(V$NFKAPPAB65_01), M00054 (V$NFKAPPAB_01), M00194 (V$NFKB_Q6) and M00208 (V$NFKB_C) using a *P*-value cutoff at 1 × 10^-3^.

## Results

**Figure 1:**
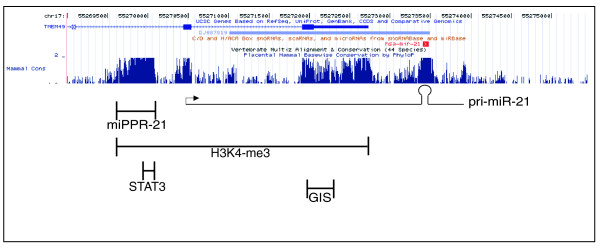
**Genomic structure of mir-21**. Location of a previously described promoter (miPPR-21) [30], our putative regulatory region (GIS) [19], a H3K4me3 binding site as determined by previous ChIP-seq [32], and a STAT3 binding site according to Loffler *et al. *[34]. Both miPPR-21 and GIS regions are highly conserved.

Using a global map of p53 transcription factor binding sites in the human genome that was generated by the ChIP-seq method [19], we searched for p53 binding at locations adjacent to miRNAs that had been shown by expression profiling to be differentially expressed in heart failure [6,7]. At least one p53 binding site was located within 3,000 bp upstream or downstream of mir-15b, mir-21 and mir-125b. We tested and found that the expression of mir-21, but not mir-15b or mir-125b, was responsive to p53 activation by doxorubicin (Additional file 2). Similarly, mir-21 was upregulated by hypoxia, which is another stimulus known to activate p53 in cardiac cells [11].

mir-21 belongs to a conserved miRNA family with single recognizable orthologs in many different invertebrate species [20]. A previous gene structure study of mir-21 identified a promoter sequence (miPPR21) in a highly conserved region approximately 2.5 kb upstream of the putative p53-binding site (which we called 'GIS') [21] (Figure 1). Aside from mir-21 itself and miPPR21, GIS is the only other region of significant sequence conservation in this genomic region (Figure 1; Additional file 3). However, analysis of GIS revealed not a p53 consensus motif, but a consensus motif for κB binding (Additional file 3). This motif is consistent with that reported in another genome-wide analysis of ChIP mapping NF-κB/RELA binding sites [22].

**Figure 2:**
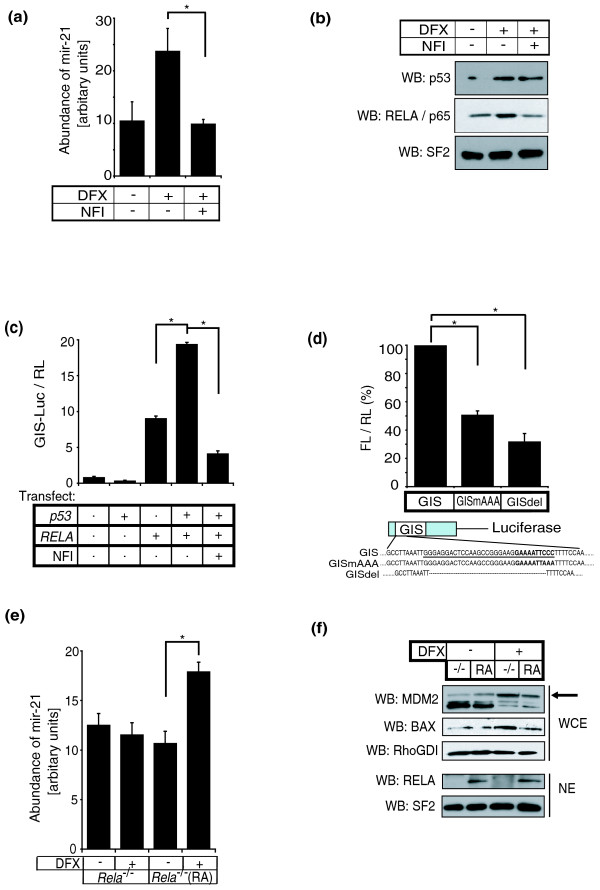
**p53 and NF-κB cooperate to induce mir-21. (a) **Primary neonatal rat cardiac fibroblasts were treated with or without DFX and the NF-κB inactivator (NFI; 1 μM quinazoline) and mir-21 was quantified using the TaqMan miRNA assay. **(b) **Nuclear extracts from cardiac fibroblasts with or without DFX and NFI as in (a) were isolated and western blotted (WB) for p53 and RELA. Splicing factor 2 (SF2), was used to confirm equal protein loading. {**(c) **GIS-luciferase (GIS-Luc) and TK-renilla control (RL) were transfected into *p53*^-/- ^MEF cells with or without plasmids encoding p53 or RELA, and incubated with or without NFI as indicated. Firefly luciferase gene reporter activity was normalized to renilla control. **(d) **GIS, GIS with an AAA mutation engineered into the putative NF-κB binding site (GISmAAA), and GIS with the NF-κB binding site deleted (GISdel) were cloned upstream of firefly luciferase. Constructs were transiently transfected together with a TK-renilla luciferase plasmid and plasmids encoding p53 and RELA into *p53*^-/- ^MEF cells and firefly luciferase reporter gene activity (FL) was quantified and normalized against renilla (RL). Results represent a fold-difference between the three different GIS-constructs. **(e) ***RelA*^-/- ^(-/-) MEF cells and *RelA*^-/- ^MEF cells that were reconstituted with *RELA *using lentiviral overexpression (RA) were treated with or without DFX, and mir-21 quantification was performed. **(f) ***RelA*^-/- ^and reconstituted *RelA*^-/- ^MEF cells were treated with or without DFX, and whole cell (WCE) and nuclear (NE) extracts were western blotted for MDM2 (arrow), BAX and RELA. RhoGDI and SF2 were used to demonstrate protein loading. miRNA quantification is shown as mean ± standard error, at least n = 3. Luciferase reporter assays are presented as mean ± standard error for at least four independent replicates. Asterisks represent *P *< 0.05 (paired *t*-test).

In the stressed myocardium, mir-21 is significantly upregulated in cardiac fibroblasts and is responsible for fibroblast growth factor secretion as well as for the extent of interstitial fibrosis in heart failure via its effect on its target gene, *Spry1 *[23]. Moreover, the therapeutic benefit of inhibiting mir-21 in heart failure was also demonstrated. We therefore focused our attention on mir-21 expression in cardiac fibroblasts and found that, as with hypoxia, the hypoxia-mimetic DFX, which effectively activates p53 *in vitro *[11], also upregulated mir-21 in primary rat cardiac fibroblasts (Figure 2a). It was also recently shown that NF-κB signaling is critical for the response to hypoxia [24] because hypoxia may directly induce NF-κB activation through a complex sequence of signals involving decreased prolyl hydroxylase-mediated prolyl hydroxylation of IKKβ leading to phosphorylation-dependent degradation of the endogenous NF-κB inhibitor, IκBα, and nuclear translocation of NF-κB [25]. Consistent with this and other data [26], we found that DFX induced NF-κB/RELA nuclear accumulation and this was significantly inhibited by the cell-permeable NF-κB inactivator quinazoline [27] (1 μM NFI; Figure 2b). Quinazoline (6-amino-4-(4-phenoxyphenylethylamino)) specifically inhibits NF-kB activation and nuclear translocation [28,29]. Correspondingly, NFI significantly inhibited DFX-induced mir-21 upregulation (Figure 2a). We also noted that DFX induced p53 nuclear accumulation as predicted but mir-21 levels were effectively inhibited by NFI, despite unchanged levels of nuclear p53 following DFX+NFI treatment (Figure 2b). These data suggested that NF-κB was the primary mediator of mir-21 induction by DFX and/or p53 induction of mir-21 required activation of NF-κB.

Next we tested the activity of the putative p53-binding site GIS by cloning it upstream of firefly luciferase and examining reporter gene expression. Supporting the hypothesis that p53 requires and cooperates with NF-κB/RELA, p53 alone did not upregulate luciferase activity, whereas p53 significantly augmented the activity that was induced by NF-κB/RELA (Figure 2c). As before, inactivation of NF-κB by NFI abrogated GIS-driven gene expression. Mutation or deletion of the κB-consensus motif in this regulatory sequence reduced p53-RELA-mediated luciferase reporter gene expression by 50% and 30%, respectively (Figure 2d). The previously described mir-21 promoter (miPPPR21) approximately 2.5 kb upstream of GIS was shown to respond through conserved AP1 and PU.1 binding sites [30]. Neither p53 nor NF-κB/RELA upregulated expression of the reporter construct based on this promoter (miPPPR21-luciferase; Additional file 4), indicating that p53/NF-κB regulated mir-21 expression through GIS but not miPPPR21.

To determine the necessity for NF-κB/RELA in mir-21 induction by DFX or p53, we incubated *RelA*^-/- ^MEF cells with or without DFX and detected no change in mir-21 levels (Figure 2e), despite DFX-induced activation of p53 as shown by an increase in p53 target gene expression (MDM2 and BAX) (Figure 2f) and an increase in reporter activity using a luciferase construct driven by 13 p53-binding sites (PG13-luciferase, data not shown). Importantly, *RelA*^-/- ^MEF cells reconstituted with ectopic *RelA *showed rescue of DFX induced mir-21 upregulation (Figure 2e).

**Figure 3:**
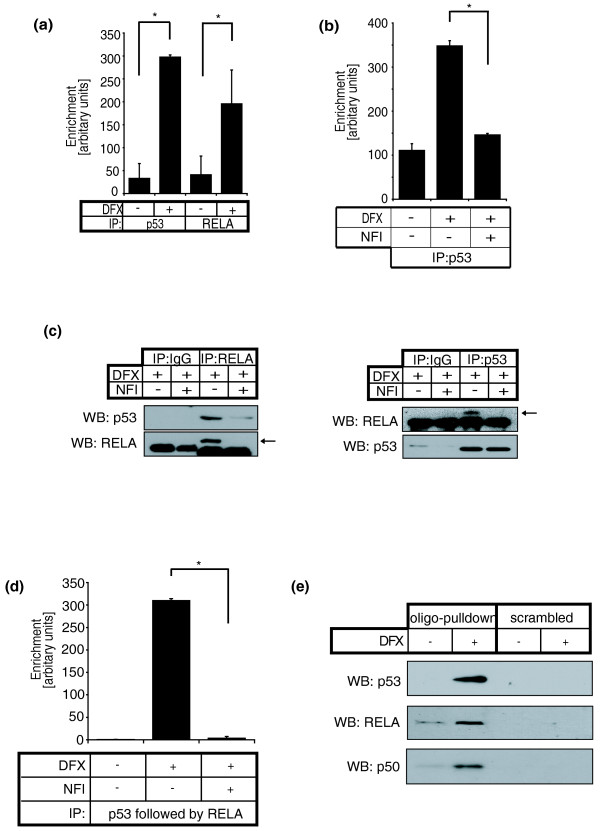
**p53 and NF-κB form a complex and occupy the putative GIS regulatory region simultaneously**. **(a) **ChIP was performed on cardiac fibroblasts with or without DFX using antibodies against either p53 or RELA. Results show fold enrichment of real-time qPCR for the putative regulatory sequence (GIS). **(b) **ChIP using a p53 antibody was performed on cardiac fibroblasts with or without DFX and NFI. ChIP results are presented as mean ± standard error for three independent experiments performed in triplicate. **(c) **Using cell lysates from cardiac fibroblasts treated with DFX with or without NFI, RELA or control IgG immunoprecipitation (IP) was performed followed by western blotting (WB) for p53 (left), and vice versa (right). Arrows indicate RELA. **(d) **Cardiac fibroblasts were treated with or without DFX and NFI, and p53 ChIP was performed followed by 'release' of the chromatin, and RELA re-ChIP. Results represent fold enrichment of real-time qPCR for GIS. Re-ChIP results are presented as mean ± standard error for two independent experiments performed in triplicate. **(e) **Lysates from cardiac fibroblasts treated with or without DFX were incubated with streptavidin-coated beads on which biotinylated GIS duplexes (oligo pulldown) or scrambled sequence duplexes (scrambled) were immobilized. Proteins bound to these duplexes were eluted and western blotted for p53, RELA and NF-κB subunit p50. Asterisks represent *P *< 0.05 (paired *t*-test).

Our results raise the possibility that RELA and p53 interact with the putative regulatory region GIS. Thus, we performed ChIP using anti-RELA and anti-p53 antibodies and found that the GIS region was occupied by both RELA and p53 *in vivo *(Figure 3a). Once again, NFI disrupted the GIS-p53 association, indicating that p53 binding required RELA (Figure 3b). To determine whether RELA and p53 co-exist in a single molecular complex, we first performed co-immunoprecipitation assays and found an interaction between endogenous RELA and p53 proteins that was disrupted by NFI (Figure 3c). The p53-RELA interaction was direct and dependent on the carboxy-terminal transactivation domain of RELA because a purified recombinant GST fusion protein of the RELA carboxy-terminal domain, but not the amino terminus DNA binding domain, was sufficient to interact with p53 (Additional file 5). Next we performed a sequential ChIP assay (re-ChIP) in which we initially performed ChIP with a p53 antibody, released the immunoprecipitated chromatin and then performed another ChIP using a RELA antibody. GIS was significantly enriched by p53-RELA re-ChIP and this association was disrupted by NFI, indicating that RELA and p53 were simultaneously residing at the GIS genomic location (Figure 3d). Furthermore, we performed oligonucleotide pulldown where the genomic sequence of GIS was synthesized, biotinylated, immobilized onto streptavidin-coated beads, and incubated with protein lysates from cells that had been treated with or without DFX. The GIS oligonucleotide, but not a scrambled control, effectively pulled down p53 and RELA in DFX-treated cells (Figure 3e). Similarly, we found that the GIS oligonucleotide also pulled down the NF-κB subunit p50 (Figure 3e) but not p52 (data not shown), suggesting that the p53-RELA complex included this subunit of NF-κB.

**Figure 4:**
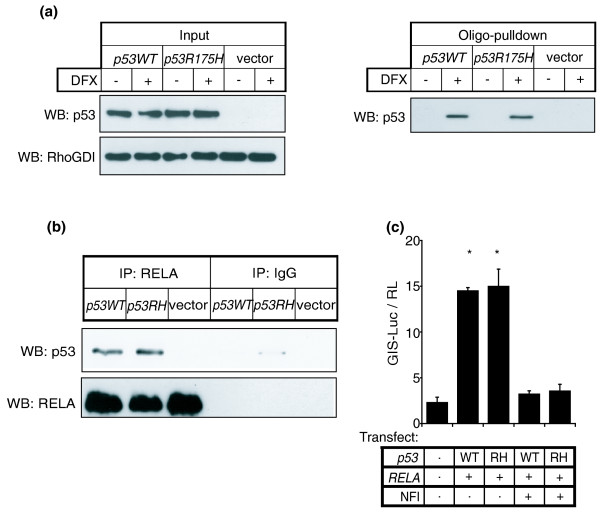
**NF-κB forms a complex at the GIS regulatory region with both wild-type p53 and p53 with a DNA-binding domain mutation**. **(a) **p53-deficient Soas2 cells were transfected with wild-type p53 (*p53WT*), DNA binding domain mutant p53 (*p53R175H*) or vector control, and cell lysates were incubated with GIS duplexes as in Figure 2e. Proteins bound to the GIS duplex were western blotted (WB) for p53 (left panel). The right panel shows input from transfected Soas2 cell lysates. **(b) **p53-deficent Soas2 cells were transfected with wild-type p53 (*p53WT*), mutant p53R175H (*p53RH*) or vector control. Cell lysates were co-immunoprecipitated with anti-RELA antibody or isotypic IgG control, and western blotting was performed for p53. **(c) **As in Figure 2c, GIS-luciferase and TK-renilla control were transfected into *p53*^-/- ^MEF cells with or without plasmids encoding *p53 *(WT and RH, respectively) and *RELA*, and incubated with or without NFI as indicated. Firefly luciferase gene reporter activity was normalized to renilla control. Asterisks represent *P *< 0.05 for treatment versus control, and with inhibitor versus without inhibitor. Luciferase reporter assays are presented as mean ± standard error for at least three independent replicates.

The presence of a κB motif instead of a p53 consensus sequence on GIS prompted us to consider if p53 was behaving as a co-factor and if the p53-GIS interaction was indirect and independent of the p53 DNA binding domain. We therefore performed another oligonucleotide-pull-down using lysates from p53-deficient cells (Soas2) pre-transfected with vector only, wild-type p53 or p53 bearing a mutation in the DNA binding domain (p53R175H). Both wild-type and mutant p53 associated with the GIS oligonucleotide (Figure 4a). Consistent with this, we also found that the p53-RELA interaction was independent of the p53 DNA binding domain (Figure 4b); moreover, mutant p53 was potentially capable of upregulating mir-21 expression (Figure 4c). Taken together, our data support the conclusion that p53 does not contribute to the GIS binding interface but instead behaves as a co-factor in this molecular complex and utilizes RELA for its association with GIS to transactivate mir-21 expression.

Sites of active chromatin at regulatory sequences are associated with the characteristic Histone-3 mark of lysine-4 tri-methylation (H3K4me3) [31]. We therefore performed ChIP using a specific H3K4me3 antibody and detected a marked enrichment of GIS compared to miPPR21 (Additional file 6). The association of the GIS genomic location with H3K4me3 has also been mapped by others in a genome-wide ChIP scan using human embryonic stem cells [32].

**Figure 5:**
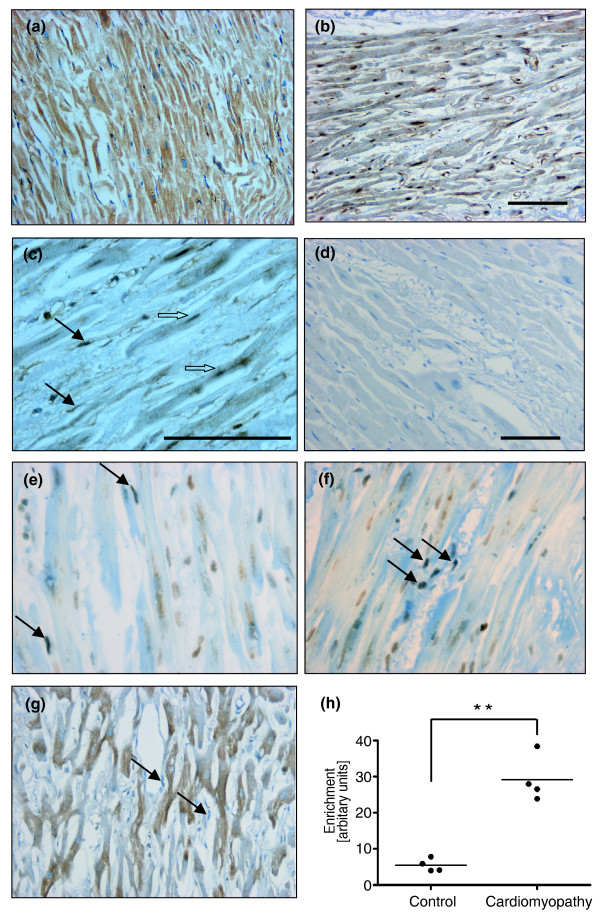
**The p53-NF-κB complex is present at the GIS regulatory region in human dilated cardiomyopathic hearts**. **(a-c) **Human left ventricular tissue sections immunostained for NF-κB/RELA showed that NF-κB/RELA (in brown) was predominantly cytoplasmic in control left ventricule (a) but nuclear in both myocytes (open arrows) and fibroblasts or non-myocytes (closed arrows) of cardiomyopathic left ventricule (b,c). **(d) **No primary antibody control. **(e,f) **Sections were also immunostained for both a marker of oxidative DNA damage (8-oxoG, in brown) and activated p53 (phospho-p53^ser15 ^(e); phospho-p53^ser20 ^(f); in black with closed arrows). **(g) **Myocytes were distinguished from non-myocytes and fibrotic tissue both by their characteristic striations and positive staining for ankyrin (in brown). Bar represents 100 μm. **(h) **Left ventricular tissues from normal hearts and cardiomyopathic hearts were used for p53-RELA re-ChIP. Results represent fold enrichment of real-time qPCR for GIS and are representative of two replicated experiments using the same eight left ventricular samples. **P < 0.0005. Patient details for these left ventricular samples are in Additional file 1.

Since levels of mir-21 are significantly elevated in dilated human hearts and murine hearts with decompensated hypertrophy [6,7,23], and Thum *et al. *[23] recently validated the therapeutic value of targeting mir-21 in a mouse model of heart failure, we undertook further analysis using human left ventricular tissues from patients who had undergone cardiac transplantation for end-stage dilated cardiomyopathy and age-matched normal control left ventricular tissues from individuals involved in road traffic accidents (Additional file 1). Despite different etiologies of heart failure (such as ischemic and non-ischemic), end-stage cardiomyopathy is collectively characterized by disease processes and molecular pathways such as apoptosis, dysregulated calcium signaling, decompensated contractility, G-protein coupled receptor down-regulation, maladaptive angiogenesis and fibrosis. Hence, as predicted for our heterogeneous series of end-stage cardiomyopathic hearts, we found significant nuclear accumulation of RELA in both myocytes and non-myocytes from cardiomyopathic hearts compared to control (Figure 5a-c). Significant p53 activation was detectable only in non-myocytes (Figure 5e-g). As a functionally significant output of the piggyback mechanism, we found that both p53 and RELA were simultaneously resident at the GIS site, and this association was significantly enriched in cardiomyopathic hearts compared to normal controls (Figure 5h).

**Figure 6:**
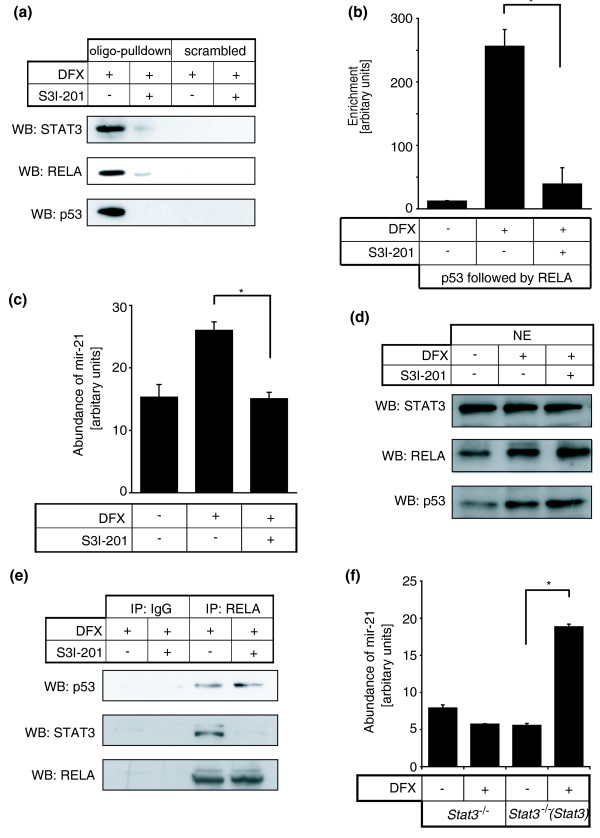
**p53-NF-κB mediated mir-21 expression is dependent on STAT3**. **(a) **Cardiac fibroblasts were treated with DFX with or without an inhibitor of STAT3 DNA binding (S3I-201), and cell lysates were incubated with streptavidin-coated beads on which biotinylated GIS duplex (oligo pulldown) or a scrambled sequence duplex (scrambled) was immobilized. Proteins bound to these duplexes were eluted and western blotted (WB) for STAT3, RELA and p53. **(b) **p53-RELA sequential ChIP was performed on cardiac fibroblasts with or without DFX and S3I-201. Results are presented as mean ± standard error for three independent experiments performed in triplicate. **(c) **Cardiac fibroblasts were treated with or without DFX and S3I-201, and mir-21 was quantified using the TaqMan miRNA assay. **(d) **Nuclear extracts (NE) from cardiac fibroblasts with or without DFX and S3I-201 were isolated and western blotted for STAT3, RELA and p53. **(e) **Using cell lysates from cardiac fibroblasts treated with DFX with or without S3I-201, RELA or control IgG immunoprecipitation (IP) was performed and western blotted for p53, STAT3 and RELA. **(f) ***Stat3*^*-/- *^MEF cells and *Stat3*^*-/- *^MEF cells that were re-constituted with wild-type *Stat3 *were treated with or without DFX and quantification for mir-21 was performed. All miRNA quantification results are presented as mean ± standard error, from three independent experiments and performed in triplicate. Asterisks represent *P *< 0.05.

We predicted that although different mechanisms may determine p53-RELA complex formation and its chromatin association, the specificity for this complex at some genomic locations such as mir-21 GIS may be assisted by additional factors. In order to investigate this, we performed parallel high-throughput sequencing with eight human cardiac sequential chromatin immunoprecipitates (four diseased and four controls; Additional file 1). We identified 26,628 genomic locations in normal hearts and 33,578 in diseased hearts (model fold = 100) aligned to the reference human genome (Gene Expression Omnibus [GES21356]). Among these, 12,311 tag locations, excluding repetitive elements, were unique to disease and had significant conservation across species (Additional files 7 and 8, and listed in Additional file 9). Of note, only 3% (381 out of 12,311) were identical to a previous global ChIP for RELA [22], although in the latter, ChIP was generated using a non-cardiac cell line and a stimulus unrelated to hypoxia. Including a location adjacent to mir-21, 1,344 out of 12,311 (10.9%) were identified to contain the *bona fide *κB consensus motif. This observation suggested that a diverse range of p53-RELA complexes may be involved in its chromatin association and most appear to be independent of the κB motif. Nonetheless, using CEAS [17], we analyzed these 1,344 genomic locations and the previous global RELA ChIP [22] in parallel. Several transcription factor motifs were overrepresented and common to both our subset of locations and the global RELA ChIP, except for STAT1, STAT3, STAT5 and STAT6 (Additional files 10, 11 and 12), with the STAT3 motif being the most prominent. The JAK/STAT3 pathway is particularly important for the secretory function and survival of cardiac fibroblasts [33]. Moreover, in multiple myeloma cancer cell lines, mir-21 expression is STAT3-mediated and two conserved STAT3 binding sites lie upstream of mir-21 [34]. We therefore examined whether the p53-RELA piggyback mechanism was STAT3-dependent. By using structure-based virtual screening, the cell-permeable compound S3I-201 was previously identified to bind to the STAT3 Src homology 2 (SH2) domain, and inhibit STAT3 dimerization, phosphorylation and DNA-binding [35]. We used S3I-201 and found that STAT3 inhibition disrupted p53-RELA- GIS association (Figure 6a, b), and inhibited mir-21 upregulation (Figure 6c), without altering p53 and RELA nuclear abundance (Figure 6d). Moreover, p53-RELA remained in complex, although STAT3 inhibition had blocked this complex from interacting with GIS and disrupted the interaction between STAT3 and p53-RELA complex (Figure 6e). Using *Stat3*^*-/- *^MEF cells, we found that STAT3 deficiency blocked DFX-induced mir-21 but this was recovered in *Stat3*^*-/- *^MEF cells that were reconstituted with wild-type *Stat3 *(Figure 6f), further demonstrating that STAT3 is required for p53-RELA-mediated mir-21 gene expression.

## Discussion

Part of the p53 response to cell death stimuli requires activation of NF-κB [36], whereas under other circumstances, NF-κB and p53 may instead be mutually repressive [37]. Likewise, the interaction between NF-κB/RELA and STAT3 may either transactivate [38] or inhibit [39] gene expression at different *cis*-regulatory elements; and the unphosphorylated STAT3-NF-κB/RELA complex has been shown to transactivate a subset of κB-dependent genes [40]. Our study links the activity of all three factors and demonstrates a specific cooperative mechanism by which p53 controls gene expression as a co-factor independent of its DNA binding domain. The p53-GIS interaction may be representative of other multiple transcription-factor loci where p53 is occupant and functional regardless of the mutation it bears. This may indeed represent a subset of genes that are transactivated by both mutant and wild-type p53.

Profiling studies of miRNA expression following p53 activating stimuli such as doxorubicin show a robust induction of up to ten-fold of another miRNA, mir-34 (reviewed in [12]). In those same studies, significant two- to four-fold induction of mir-21 was also detected. The difference in p53-dependent mir-34 and mir-21 upregulation may reflect the different molecular mechanisms: in the case of mir-34, p53 binds directly to a p53 consensus motif in the mir-34 regulatory element; for mir-21, p53 binds as a co-factor to RELA to control mir-21 expression. Our analysis of the genome-wide map of p53-NF-κB binding sites suggests that the molecular complex may regulate gene expression more widely, although the exact mechanism by which it operates in each location is not identical.

The central role of the transcription factor p53 in the transition from compensated cardiac hypertrophy to dilated cardiomyopathy was recently demonstrated [11]. Myocardial stress response through NF-κB activation and STAT3 signaling has also been described elsewhere [32,41,42]. Our findings here combine these results to suggest the role of a molecular complex comprising all three factors in regulating at least the expression of mir-21 in this disease context. The therapeutic benefit of inhibiting mir-21 has previously been shown [23] and our work adds to the understanding of how mir-21 is upregulated in heart failure.

On a wider level, our findings add to an emerging paradigm that revises our understanding of how transcription factors regulate gene expression at *cis*-regulatory elements. Genome-wide scans now show that, in the large majority of cases, where association between a transcription factor and genomic location is demonstrated, a classical consensus motif for the transcription factor cannot be identified. Instead, a transcription factor may utilize the consensus motif of a binding partner to regulate gene expression cooperatively. For example, we recently showed that the estrogen receptor-binding site of the *ERBB2 *gene contains a paired box 2 (PAX) consensus motif so that an estrogen receptor-PAX complex is recruited to repress *ERBB2 *gene expression following tamoxifen treatment [2]. The mechanism we have identified here may also underlie the ability of wild-type or mutant p53 to function as co-factors in order to regulate other target genes whose promoters lack a p53 consensus sequence.

## Conclusions

We have demonstrated that the cooperation between three transcription factors in a multi-protein complex may control gene expression program in heart failure. Understanding the way that different DNA binding proteins interact with DNA regulatory elements and modulate gene expression will provide information for drug therapy design for diseases such as heart failure.

## Abbreviations

bp: base pair; CEAS: the Cis-regulatory Element Annotation System; ChIP: chromatin immunoprecipitation; DFX: deferroxamine; MEF: mouse embryo fibroblast; miRNA: microRNA; NF: nuclear factor; NFI: NFκB activation inhibitor; qPCR: quantitative PCR; re-ChIP: sequential ChIP.

## Competing interests

The authors declare that they have no competing interests.

## Authors' contributions

MKC and RF carried out the cellular, genetic and immunoprecipitation experiments. MKC performed ChIP on human myocardial tissue, and analyzed the sequencing results. MM, LS and AV carried out quantitative PCR and western blot experiments. MG collected and dissected the human myocardial tissue. AS and NP provided essential RELA reagents, including retrovirally transfected *Rela*^-/- ^MEF cells. NF performed immunohistochemistry. MKC, MB, JC and RF designed the experiments. MKC, MB and RF drafted the manuscript. All authors read and approved the final manuscript.

## Supplementary Material

Additional file 1 **Details of human cardiomyopathic and normal control left ventricular explants**. Click here for file

Additional file 2 **(a) H9c2 cardiac cells were treated with or without doxorubicin (an activator of p53)**. Small RNAs were isolated and quantified using TaqMan miRNA assays. **(b) **Primary neonatal rat cardiac fibroblasts were incubated in normoxia or <1% hypoxia for 48 h and mir-21 quantification was performed. miRNA quantification is shown as mean ± standard error from three independent experiments performed in triplicate. Bottom panels: western blot of nuclear fraction demonstrating p53 accumulation with either doxorubicin or hypoxia stimuli. **(c) **Western blot demonstrating significant p53 accumulation in the nucleus, but not the cytosol, following DFX treatment (as in Figure 1b). Blots with anti-SF2 (nuclear marker) and anti-RhoGDI (cytosol marker) demonstrate effective cellular fractionation for the two compartments. Asterisks represent *P *< 0.05 for treatment versus control. Click here for file

Additional file 3 **Conservation between the human, rat and mouse GIS regulatory sequences is shown**. Box represents the putative NF-κB motif. Click here for file

Additional file 4 **The previously described mir-21 promoter **[30]**was cloned upstream of firefly luciferase (miPPR21-Luc) and transfected together with TK-renilla control, with or without plasmids encoding p53 and RELA, and incubated with or without NFI as indicated**. Assays are presented as mean ± standard error for four independent replicates. Click here for file

Additional file 5 **p53 binds RELA through the RELA transactivation domain**. Purified recombinant GST-RELA peptides (amino terminus, DNA binding domain; carboxyl terminus, transactivation domain; in the left panel, the arrow indicates the GST-RELA 428-551 amino acid peptide) were mixed with purified recombinant His-tagged full-length p53 (right lower panel), and analyzed by immunoprecipitation (IP) and western blotting (WB) (right upper panel). Click here for file

Additional file 6 **H3K4me3 and control IgG ChIP were performed on cardiac fibroblasts in the presence of DFX**. Results represent fold enrichment of real-time qPCR for the previously described mir-21 promoter (miPPR-21) and GIS. ChIP results are presented as mean ± standard error for two independent experiments performed in triplicate. Click here for file

Additional file 7 **Location of p53-RELA binding sites relative to genomic structures, as annotated using CEAS in Ji *et al. ***[17]. Proximal promoters, 1 kb upstream from RefSeq 5' start; immediate downstream, 1 kb from RefSeq 3' end. Click here for file

Additional file 8 **Average conservation plot from the analysis of the p53-RELA binding sites using CEAS demonstrating that the 12,311 tag locations of binding sites unique to disease were strongly conserved across species**. Click here for file

Additional file 9 **re-ChIP-seq p53-RELA binding sites**. Click here for file

Additional file 10 **(a-e) Motifs that were overrepresented in the subset of p53-RELA re-ChIP sites (total of 1,344 locations containing the *bona fide *κB motif (a)), compared to RELA alone ChIP sites: STAT3 (b), STAT6 (c), STAT1 (d), STAT5A (e)**. Click here for file

Additional file 11 **List of transcription factor motifs enriched in the subset of p53-RELA binding sites (total of 1,344 sites) containing the *bona fide *κB motif**. Click here for file

Additional file 12 **List of transcription factor motifs enriched in the previously published **[22]**genome-wide RELA binding sites dataset (PET2 and PET3 clusters)**. Click here for file
