# Supplementary material for: High-throughput sequencing identifies STAT3 as the DNA-associated factor for p53-NF-κB-complex-dependent gene expression in human heart failure
Source: Genome Med. 2010 Jun 14;2(6):37. doi: 10.1186/gm158 (PMC2905097; doi:10.1186/gm158)
Supplement: Additional file 1 — Details of human cardiomyopathic and normal control left ventricular explants. [file gm158-S1.DOC]

**Additional file 1**. Details of human cardiomyopathic and normal control left ventricular explants.

| Human LV samples | Age | Details |
| --- | --- | --- |
| Control | 47 | RTA |
|  | 50 | RTA |
|  | 41 | RTA |
|  | 52 | RTA |
| End-stage  cardiomyopathy | 53 | Non-ischaemic |
|  | 42 | Non-ischaemic |
|  | 61 | Ischaemic |
|  | 58 | Ischaemic |
