# Supplementary figures and images for: High-throughput sequencing identifies STAT3 as the DNA-associated factor for p53-NF-κB-complex-dependent gene expression in human heart failure
Source: Genome Med. 2010 Jun 14;2(6):37. doi: 10.1186/gm158 (PMC2905097; doi:10.1186/gm158)

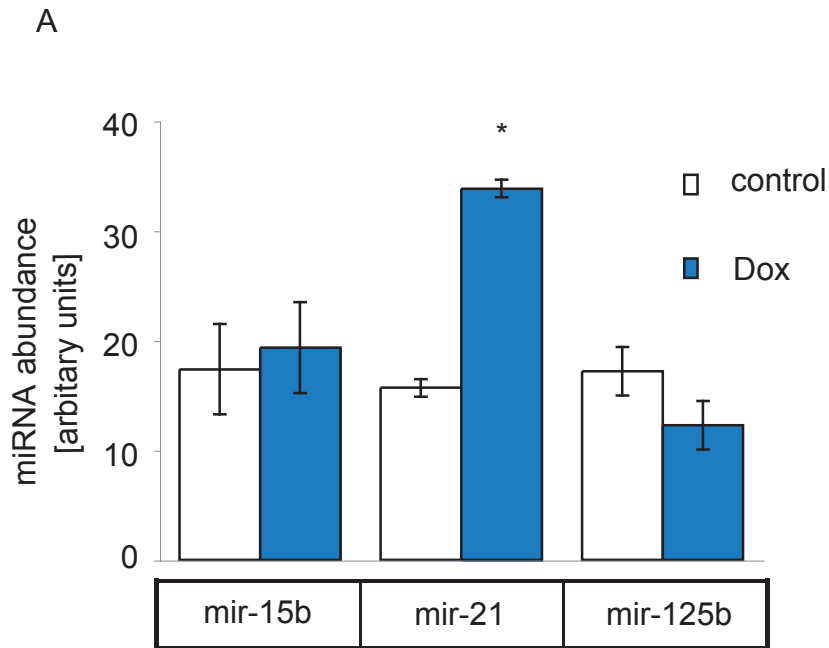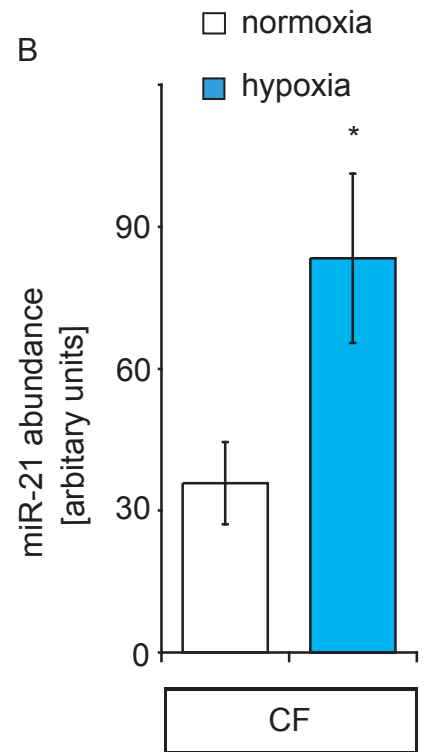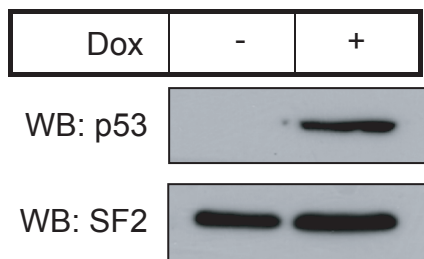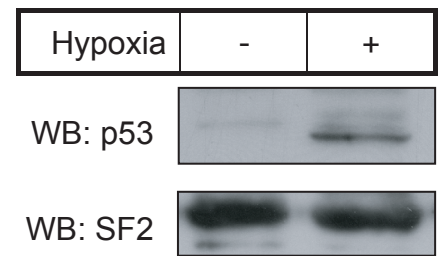

**C**

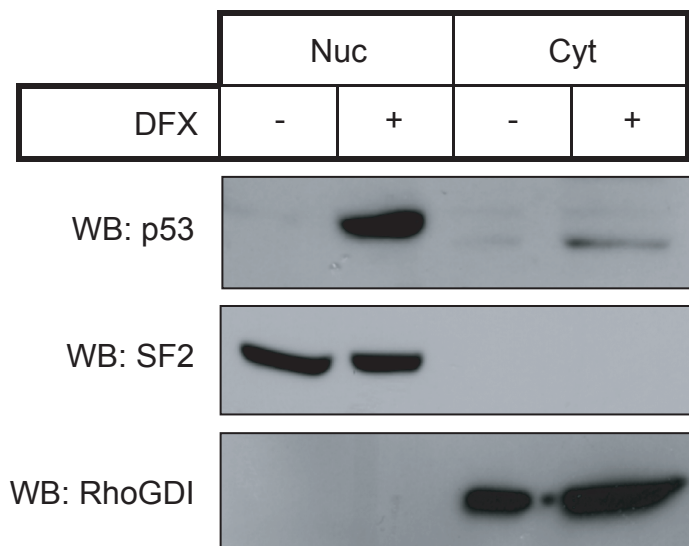

Supplement: Additional file 2 — (a) H9c2 cardiac cells were treated with or without doxorubicin (an activator of p53). Small RNAs were isolated and quantified using TaqMan miRNA assays. (b) Primary neonatal rat cardiac fibroblasts were incubated in normoxia or <1% hypoxia for 48 h and mir-21 quantification was performed. miRNA quantification is shown as mean ± standard error from three independent experiments performed in triplicate. Bottom panels: western blot of nuclear fraction demonstrating p53 accumulation with either doxorubicin or hypoxia stimuli. (c) Western blot demonstrating significant p53 accumulation in the nucleus, but not the cytosol, following DFX treatment (as in Figure 1b). Blots with anti-SF2 (nuclear marker) and anti-RhoGDI (cytosol marker) demonstrate effective cellular fractionation for the two compartments. Asterisks represent P < 0.05 for treatment versus control. [file gm158-S2.PDF]

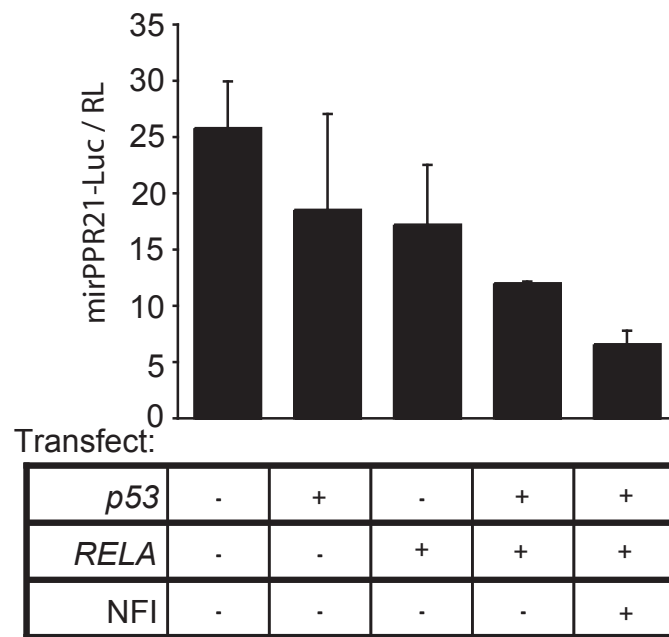

Supplement: Additional file 4 — The previously described mir-21 promoter [30]was cloned upstream of firefly luciferase (miPPR21-Luc) and transfected together with TK-renilla control, with or without plasmids encoding p53 and RELA, and incubated with or without NFI as indicated. Assays are presented as mean ± standard error for four independent replicates. [file gm158-S4.PDF]

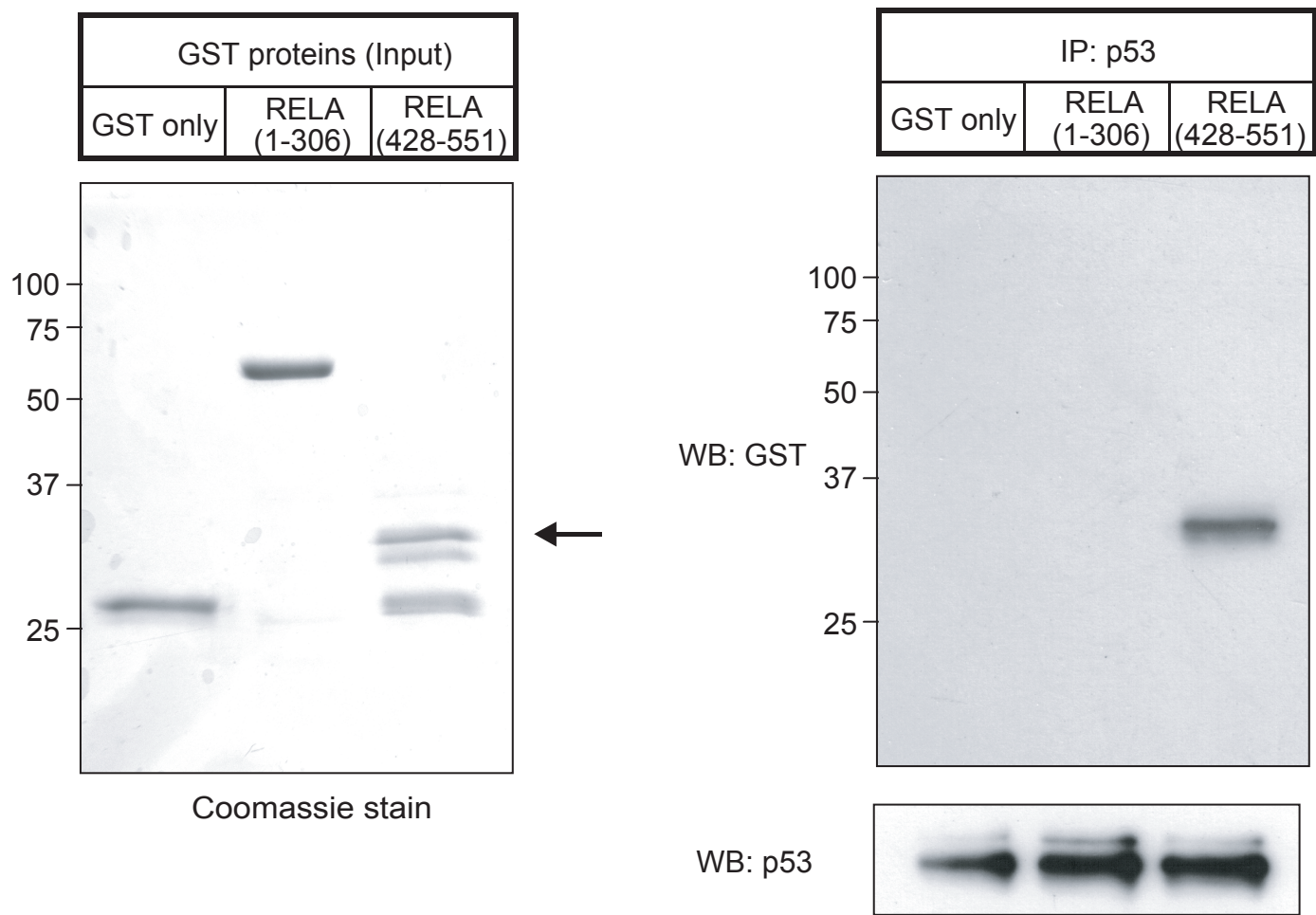

Supplement: Additional file 5 — p53 binds RELA through the RELA transactivation domain. Purified recombinant GST-RELA peptides (amino terminus, DNA binding domain; carboxyl terminus, transactivation domain; in the left panel, the arrow indicates the GST-RELA 428-551 amino acid peptide) were mixed with purified recombinant His-tagged full-length p53 (right lower panel), and analyzed by immunoprecipitation (IP) and western blotting (WB) (right upper panel). [file gm158-S5.PDF]

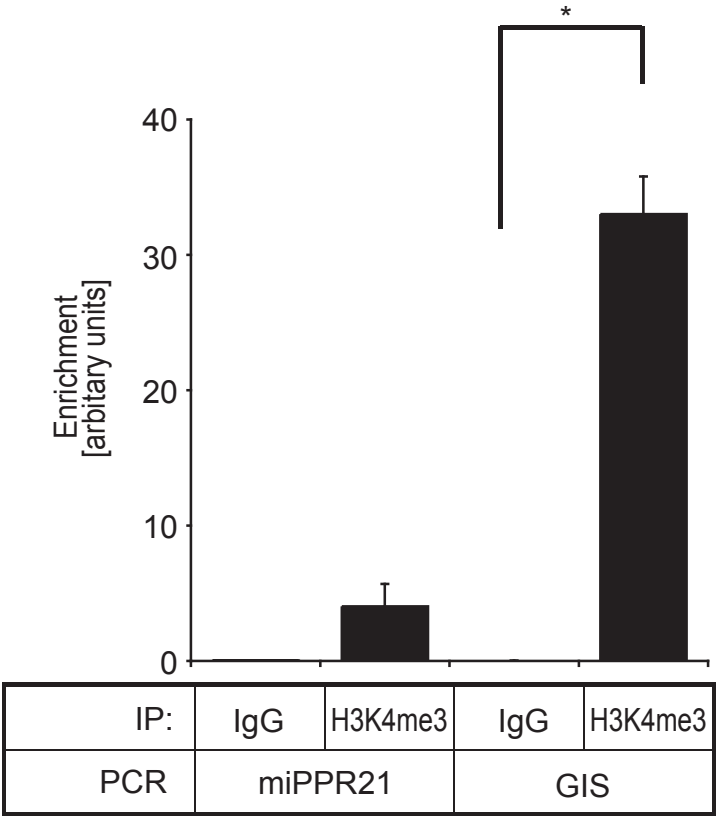

Supplement: Additional file 6 — H3K4me3 and control IgG ChIP were performed on cardiac fibroblasts in the presence of DFX. Results represent fold enrichment of real-time qPCR for the previously described mir-21 promoter (miPPR-21) and GIS. ChIP results are presented as mean ± standard error for two independent experiments performed in triplicate. [file gm158-S6.PDF]

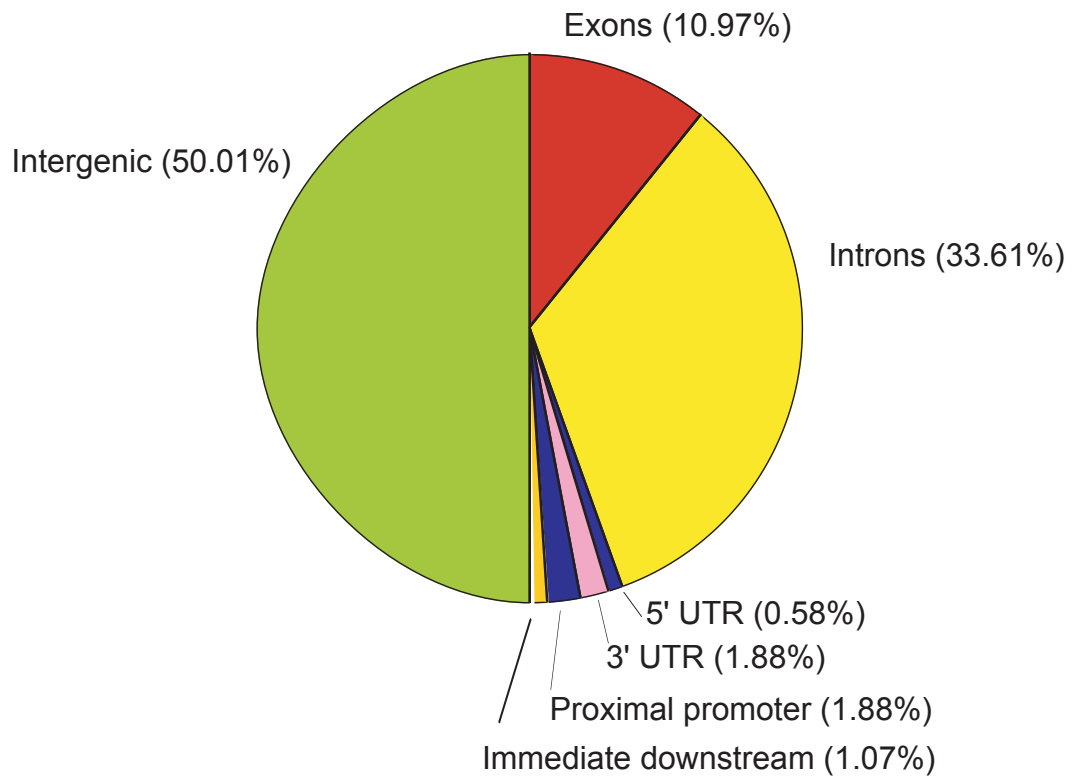

Supplement: Additional file 7 — Location of p53-RELA binding sites relative to genomic structures, as annotated using CEAS in Ji et al. [17]. Proximal promoters, 1 kb upstream from RefSeq 5' start; immediate downstream, 1 kb from RefSeq 3' end. [file gm158-S7.PDF]

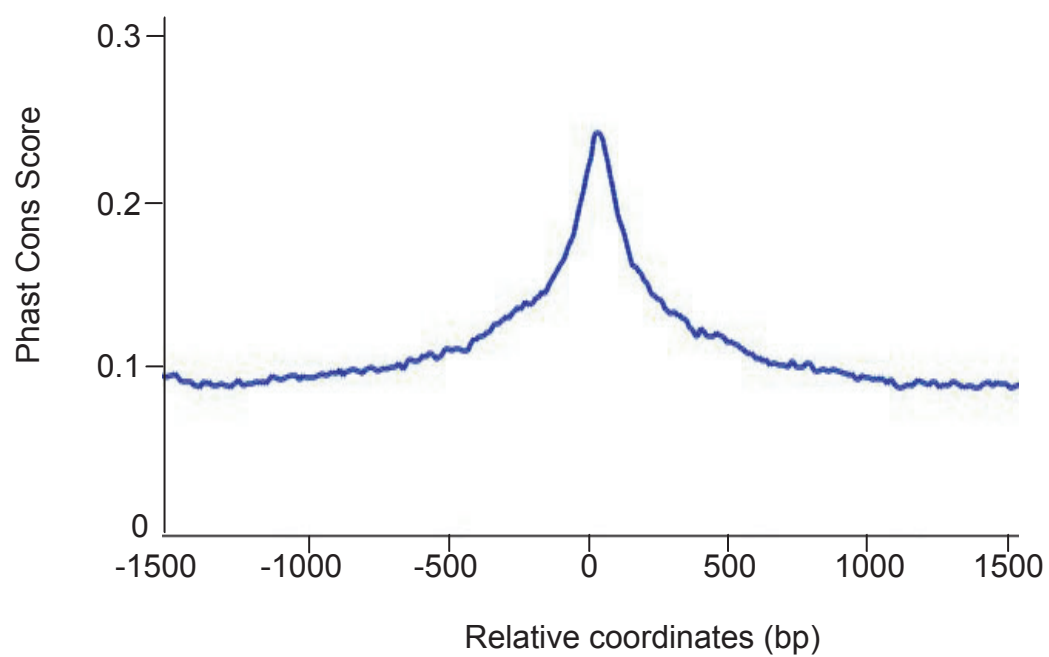

Supplement: Additional file 8 — Average conservation plot from the analysis of the p53-RELA binding sites using CEAS demonstrating that the 12,311 tag locations of binding sites unique to disease were strongly conserved across species. [file gm158-S8.PDF]

A

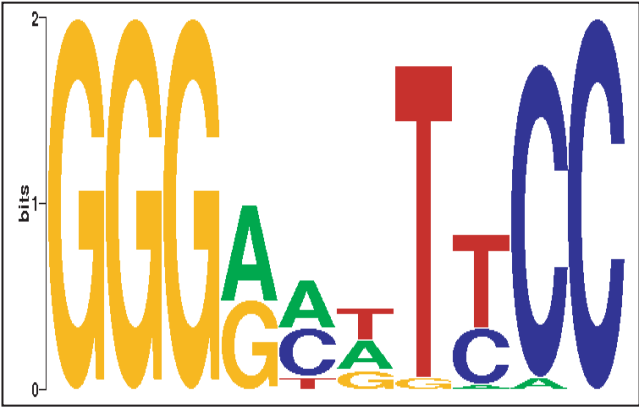

B

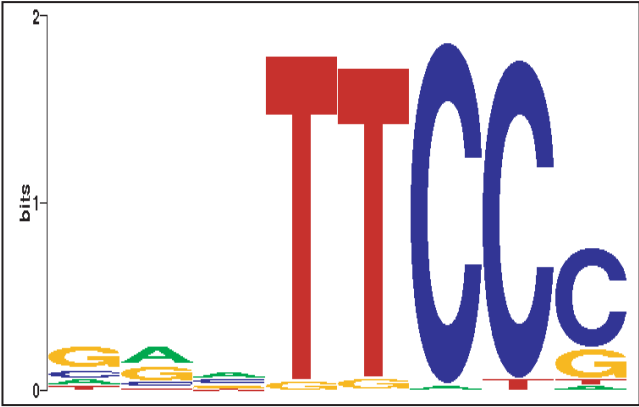

C

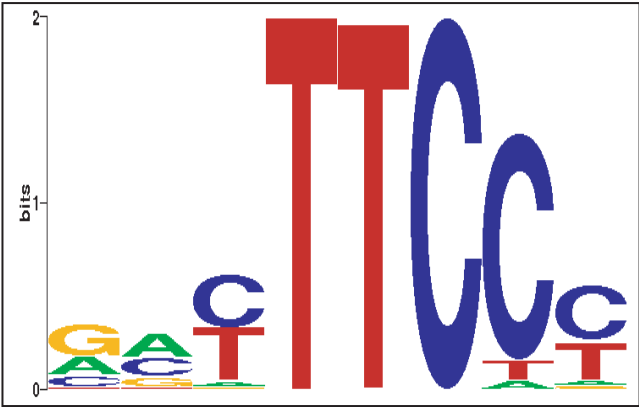

D

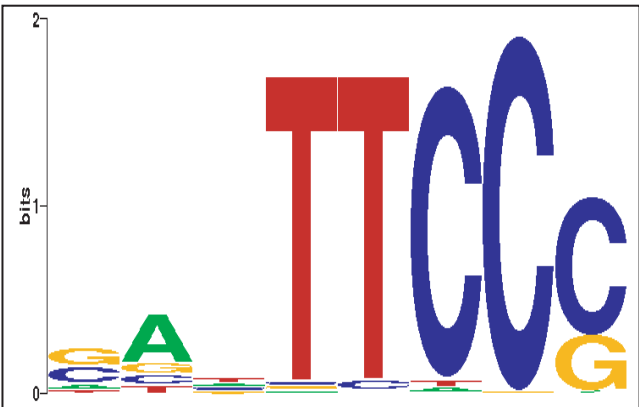

E

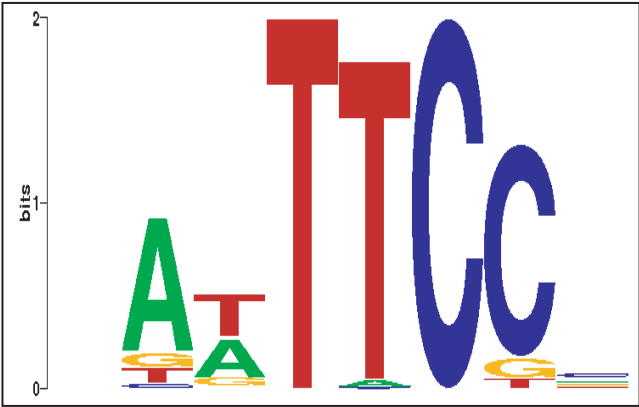

Supplement: Additional file 10 — (a-e) Motifs that were overrepresented in the subset of p53-RELA re-ChIP sites (total of 1,344 locations containing the bona fide κB motif (a)), compared to RELA alone ChIP sites: STAT3 (b), STAT6 (c), STAT1 (d), STAT5A (e). [file gm158-S10.PDF]
