# Supplementary material for: High-throughput sequencing identifies STAT3 as the DNA-associated factor for p53-NF-κB-complex-dependent gene expression in human heart failure
Source: Genome Med. 2010 Jun 14;2(6):37. doi: 10.1186/gm158 (PMC2905097; doi:10.1186/gm158)
Supplement: Additional file 3 — Conservation between the human, rat and mouse GIS regulatory sequences is shown. Box represents the putative NF-κB motif. [file gm158-S3.PDF]

Additional file 3 (Choy, *et al*)

```
                10         20         30         40         50         60         70         80
human  CCTTTTTCATTGCATTGTTCTGCATTTATTTCTACAGGGAGAAAACTGGTTGTCCTGGATGTTTGAAAAGTTGGTCGTTG 80
rat    -CGTTTTCCTTGAATTGTTCTGCTTG-TTCCCTCCAGGGAGAAAACTGGTTATCCTGGACGTTTGAGAAGCTGGTCGTTG 78
mouse  -CCTTTTTCTTGAATTGTTCTGCTTTCTTTCCTACAGGGAGAAAACTGGTTATCCTGGATGTTTGAGAAGCTGGTGGTTG 79

                90        100        110        120        130        140        150        160
       TCATGGTGTGTTACTTCATCCTATCTATCATTAACTCCATGGCACAAAGTTATGCCAAACGAATCCAGCAGCGGTTGAAC 160
       CCATGGTGTGTTACTTTATCCTGTCTATCATTAACTCCATGGCACAAAGCTATGCCAAACGAATCCAGCAGCGCTTGAAC 158
       CAATGGTGTGTTACTTTGTCCTGTCTATTATTAACTCCATGGCACAAAACTATGCCAAACGAATCCAGCAGCGCTTGAAC 159

               170        180        190        200        210        220        230        240
       TCAGAGGAGAAAACTAAATAAGTAGAGAAAGTTTTAAACTGCAGAAATTGGAGTGGATGGGTTCTGCCTTAAATTGGGAG 240
       TCAGAGGAGAAGACTAAATAAGCAGAGA--GTTTTTATCTGCAGAAGTTAGCGTGGTGGAG-CCTGCCTTACATCGGGAG 235
       TCAGAGGAGAAAACTAAATAAGCAGAGA--GTTTTTATCTGCAGAAGTTAACATGGTGGAGTCCTGCCTTACACTGGGAG 237

               250        260        270        280        290        300        310        320
       GACTCCAAGCCGGGAAGGAAAATTCCCTTTTCCAACCTGTATCAATTTTTACAACTTT-TTTCCTGAAAGCAGTTTAGTC 319
       GGCTCTCA-CCAGGAAGGAAGATCCCCATTTCCAACCTGTACTGATTTTTAAAACATTTCTCCCTGAAAGCAGTTTAGTC 314
       GGCTCTAA-CCAGGAAGGAAGGTCCCCGTTTCCAACCTGTGTTGATTTTTAAAACATT-CTTCCTGAAAGCAGTTTAGTC 315

               330        340        350        360        370        380        390        400
       CATACTTTGCACTGACATACTTTTTCCTTCTGTGCTAAGGTAAGGT--ATCCACCCTCGATGCAATCCACCTTGTGTTTT 397
       CACATTTTACACTGACATACTTTTGCTTTCTTTGTTAAGGTAAGGT--CTCCACCCTCGATTCAATCCACATTGTATTCC 392
       CATATGTTACACTGACATACTTTA------TTTGTTAAGGTAAGGTGTCTACACCCTCAGTTCAATCCACGTCGTATTCT 389

               410        420        430        440        450        460        470        480
       CTTAGGGTGGAATGTGATGTTCAGCAGCAAACTTGCAACAGACTGGCCTTCTGTTTGTTACTTTCAAAAGGCCCACATGA 477
       -TTAGGGTGGA-TATGATGTTCTGCTGCAAACTTAACAAAA-CTGGCCTTCTGAC----ACTTTCACAGGGCC-ACATGG 464
       -TTAGGGTGGA-TGTGATGTTCTGCTGCAAACTTAACAAAAACTGGCCTTCTGAT----ACCTTCACAGGGCC-ACA--- 459

               490        500        510        520        530        540        550        560
       TACAATTAGAGAATTCCCACCGCACAAAAAAAGTTCCTAAGTATGTTAAATATGTCAAGCTTTTTAGGCTTGTCACAAAT 557
       TCCAACTGGAGAACCTCGGCCACACAGAACC--TTCTGACGTATGTTAAATATGCCAGGCTTTTCAGGCTTGTCACAAAT 542
       ------------------------CAGAACC--TTCTGAAGTATGTTAAATATGTCAGGCTTTTTAGGCTTGTCACAAAT 513

               570        580        590        600        610        620        630        640
       GATTGCTTTGTTTTCCTAAGTCATCAAAATGTATATAAATTATCTAGATTGGATAACAGTCTTGCATGTTTATCATGTTA 637
       GATTGGTTT---TT-CTAAGTCACCAAA-TGTATATAAGTTATATATGTTGGATAGCAGTCTTGCATGCCTATCATGGAA 617
       GATTGTTTT---TTTCTAAGTCACCAAA-TGTATATAAGTTATATATATTGGATAGCAATCTTGCATGTCTATCATGGAA 589

               650        660        670        680        690        700        710        720
       CAATTTAATATTCCATCCTGCCCAACCCTTCCTCTCCCATCCTCAAAAAAGGGCCATTTTATGATGCATTGCACACCCTC 717
       CAATGTAATATGCC--------------TTCCTTTCCCACCCTCAAAAA--GGCCATTTTATGATGCATTGCACACCCTC 681
       CAATTTAATATGCC--------------TTCCTTTCCCACCCTCAAAAA--GGCCATTTTATGACGCATTGCACACCCTC 653

               730        740        750        760        770        780        790        800
       TGGGGAAATTGATCTTTAAATTTTGAGACAGTATAAGGAAAATCTGGTTGGTGTCTTACAAGTGAGCTGACACCATTTTT 797
       TGGGGAAATTGATCTTTAAATTTTGAGACAGTATAAGGAAAATCTGGTTGGTGTCTCACAAATGAGT--ACGCCATTTTT 759
       TGGGGAAATTGATCTTTAAATTTTGAAACAGTATAAGGAAAATCTGGTTGGTGTCTCACAAATGAGT--ACGCCATTTTT 731

               810        820        830        840        850        860        870        880
       TATTCTGTGTATTTAGAATGAAGTCTTGAAAAAAACTTTATAAAGACATCTTTAATCATTCCAAAATTGTGTCCGTTTTC 877
       TATTCTGTATATTTAGAATGAAGTCGTGAAAAA--CTTTATAAAGACATCTTTGATCATTCCAAAATTGTGTC        830
       TATTCTGTATATTTAGAATGAAGTCGTGAAAAA--CTTTATAAAGACATCTTTGATCATTCCGAAATT             797
```
