# Supplementary material for: High-throughput sequencing identifies STAT3 as the DNA-associated factor for p53-NF-κB-complex-dependent gene expression in human heart failure
Source: Genome Med. 2010 Jun 14;2(6):37. doi: 10.1186/gm158 (PMC2905097; doi:10.1186/gm158)
Supplement: Additional file 9 — re-ChIP-seq p53-RELA binding sites. [file gm158-S9.PDF]

chr1	106375556	106375648	1	0.032258065
chr1	93718380	93718487	2	0.037037037
chr1	246860374	246860469	3	0.041666667
chr1	112682086	112682180	4	0.042105263
chr1	145150953	145151045	5	0.043010753
chr1	116872377	116872456	6	0.05
chr1	200791489	200791548	7	0.05
chr1	171717127	171717203	8	0.051948052
chr1	10004527	10004564	9	0.052631579
chr1	100276988	100277025	10	0.052631579
chr1	100656012	100656049	11	0.052631579
chr1	101324997	101325034	12	0.052631579
chr1	10192844	10192881	13	0.052631579
chr1	101975354	101975391	14	0.052631579
chr1	102467952	102467989	15	0.052631579
chr1	102739001	102739038	16	0.052631579
chr1	102963809	102963846	17	0.052631579
chr1	105330260	105330297	18	0.052631579
chr1	105733823	105733860	19	0.052631579
chr1	105910157	105910194	20	0.052631579
chr1	105980457	105980494	21	0.052631579
chr1	106313787	106313824	22	0.052631579
chr1	10636781	10636818	23	0.052631579
chr1	106418174	106418211	24	0.052631579
chr1	106497405	106497442	25	0.052631579
chr1	10704012	10704049	26	0.052631579
chr1	107523261	107523298	27	0.052631579
chr1	107895699	107895736	28	0.052631579
chr1	108227225	108227262	29	0.052631579
chr1	108265403	108265440	30	0.052631579
chr1	108854400	108854437	31	0.052631579
chr1	109166704	109166741	32	0.052631579
chr1	109520199	109520236	33	0.052631579
chr1	109746158	109746195	34	0.052631579
chr1	109809039	109809076	35	0.052631579
chr1	109836760	109836797	36	0.052631579
chr1	109892775	109892812	37	0.052631579
chr1	110154203	110154240	38	0.052631579
chr1	11038681	11038718	39	0.052631579
chr1	110446564	110446601	40	0.052631579
chr1	110654316	110654353	41	0.052631579
chr1	11080527	11080564	42	0.052631579
chr1	110948347	110948384	43	0.052631579
chr1	111239722	111239759	44	0.052631579
chr1	11174573	11174610	45	0.052631579
chr1	111800229	111800266	46	0.052631579
chr1	112056867	112056904	47	0.052631579
chr1	112418644	112418681	48	0.052631579
chr1	112476936	112476973	49	0.052631579
chr1	112656776	112656813	50	0.052631579

chr1	113035765	113035802	51	0.052631579
chr1	113054420	113054457	52	0.052631579
chr1	113381430	113381467	53	0.052631579
chr1	114180528	114180565	54	0.052631579
chr1	114468905	114468942	55	0.052631579
chr1	114484423	114484460	56	0.052631579
chr1	114955568	114955605	57	0.052631579
chr1	115656036	115656073	58	0.052631579
chr1	115768227	115768264	59	0.052631579
chr1	115786331	115786368	60	0.052631579
chr1	115821036	115821073	61	0.052631579
chr1	115849950	115849987	62	0.052631579
chr1	116077887	116077924	63	0.052631579
chr1	116613912	116613949	64	0.052631579
chr1	11816200	11816237	65	0.052631579
chr1	119674141	119674178	66	0.052631579
chr1	119674810	119674847	67	0.052631579
chr1	120688971	120689008	68	0.052631579
chr1	120920253	120920290	69	0.052631579
chr1	12328371	12328408	70	0.052631579
chr1	1416111	1416148	71	0.052631579
chr1	143712092	143712129	72	0.052631579
chr1	143759056	143759093	73	0.052631579
chr1	143838121	143838158	74	0.052631579
chr1	14446469	14446506	75	0.052631579
chr1	14447232	14447269	76	0.052631579
chr1	14542325	14542362	77	0.052631579
chr1	145857991	145858028	78	0.052631579
chr1	147451538	147451575	79	0.052631579
chr1	147478644	147478681	80	0.052631579
chr1	14754450	14754487	81	0.052631579
chr1	147978270	147978307	82	0.052631579
chr1	148050350	148050387	83	0.052631579
chr1	148051474	148051511	84	0.052631579
chr1	148124662	148124699	85	0.052631579
chr1	148165030	148165067	86	0.052631579
chr1	148188597	148188634	87	0.052631579
chr1	148217269	148217306	88	0.052631579
chr1	148314901	148314938	89	0.052631579
chr1	148861799	148861836	90	0.052631579
chr1	149143000	149143037	91	0.052631579
chr1	149387063	149387100	92	0.052631579
chr1	149566456	149566493	93	0.052631579
chr1	14994431	14994468	94	0.052631579
chr1	149954269	149954306	95	0.052631579
chr1	150080991	150081028	96	0.052631579
chr1	150128297	150128334	97	0.052631579
chr1	150594053	150594090	98	0.052631579
chr1	150757847	150757884	99	0.052631579
chr1	150994692	150994729	100	0.052631579

chr1	151026572	151026609	101	0.052631579
chr1	151125681	151125718	102	0.052631579
chr1	151368751	151368788	103	0.052631579
chr1	151774365	151774402	104	0.052631579
chr1	151864405	151864442	105	0.052631579
chr1	152000728	152000765	106	0.052631579
chr1	152408417	152408454	107	0.052631579
chr1	152473602	152473639	108	0.052631579
chr1	152476129	152476166	109	0.052631579
chr1	15274937	15274974	110	0.052631579
chr1	152789416	152789453	111	0.052631579
chr1	153494174	153494211	112	0.052631579
chr1	153717105	153717142	113	0.052631579
chr1	153925069	153925106	114	0.052631579
chr1	154582695	154582732	115	0.052631579
chr1	154716155	154716192	116	0.052631579
chr1	154845453	154845490	117	0.052631579
chr1	154894646	154894683	118	0.052631579
chr1	155038163	155038200	119	0.052631579
chr1	1550868	1550905	120	0.052631579
chr1	155220433	155220470	121	0.052631579
chr1	155543115	155543152	122	0.052631579
chr1	15563306	15563343	123	0.052631579
chr1	156292047	156292084	124	0.052631579
chr1	156938049	156938086	125	0.052631579
chr1	158156896	158156933	126	0.052631579
chr1	158250718	158250755	127	0.052631579
chr1	158366440	158366477	128	0.052631579
chr1	158449897	158449934	129	0.052631579
chr1	158609932	158609969	130	0.052631579
chr1	158639286	158639323	131	0.052631579
chr1	159284409	159284446	132	0.052631579
chr1	159395804	159395841	133	0.052631579
chr1	159395888	159395925	134	0.052631579
chr1	159465445	159465482	135	0.052631579
chr1	159610993	159611030	136	0.052631579
chr1	159742153	159742190	137	0.052631579
chr1	159951085	159951122	138	0.052631579
chr1	160141996	160142033	139	0.052631579
chr1	160459133	160459170	140	0.052631579
chr1	160597279	160597316	141	0.052631579
chr1	160933029	160933066	142	0.052631579
chr1	161077728	161077765	143	0.052631579
chr1	161306128	161306165	144	0.052631579
chr1	161306224	161306261	145	0.052631579
chr1	16133354	16133391	146	0.052631579
chr1	161432997	161433034	147	0.052631579
chr1	16253933	16253970	148	0.052631579
chr1	162798457	162798494	149	0.052631579
chr1	163100672	163100709	150	0.052631579

chr1	163101150	163101187	151	0.052631579
chr1	16334745	16334782	152	0.052631579
chr1	163385640	163385677	153	0.052631579
chr1	163446594	163446631	154	0.052631579
chr1	163539274	163539311	155	0.052631579
chr1	163539372	163539409	156	0.052631579
chr1	163655556	163655593	157	0.052631579
chr1	164099612	164099649	158	0.052631579
chr1	164306456	164306493	159	0.052631579
chr1	16436048	16436085	160	0.052631579
chr1	164914466	164914503	161	0.052631579
chr1	165163163	165163200	162	0.052631579
chr1	166135481	166135518	163	0.052631579
chr1	166435825	166435862	164	0.052631579
chr1	166836824	166836861	165	0.052631579
chr1	166973519	166973556	166	0.052631579
chr1	166974353	166974390	167	0.052631579
chr1	167054259	167054296	168	0.052631579
chr1	16722497	16722534	169	0.052631579
chr1	167560438	167560475	170	0.052631579
chr1	167614350	167614387	171	0.052631579
chr1	167816944	167816981	172	0.052631579
chr1	1680591	1680628	173	0.052631579
chr1	168554814	168554851	174	0.052631579
chr1	168556267	168556304	175	0.052631579
chr1	168815922	168815959	176	0.052631579
chr1	170152621	170152658	177	0.052631579
chr1	170204690	170204727	178	0.052631579
chr1	170317627	170317664	179	0.052631579
chr1	17079441	17079478	180	0.052631579
chr1	1708268	1708305	181	0.052631579
chr1	171010014	171010051	182	0.052631579
chr1	171568645	171568682	183	0.052631579
chr1	171944809	171944846	184	0.052631579
chr1	172602207	172602244	185	0.052631579
chr1	173047694	173047731	186	0.052631579
chr1	173216746	173216783	187	0.052631579
chr1	173235366	173235403	188	0.052631579
chr1	173303302	173303339	189	0.052631579
chr1	1737066	1737103	190	0.052631579
chr1	174825004	174825041	191	0.052631579
chr1	174919096	174919133	192	0.052631579
chr1	174929631	174929668	193	0.052631579
chr1	176031969	176032006	194	0.052631579
chr1	176058073	176058110	195	0.052631579
chr1	176678490	176678527	196	0.052631579
chr1	176943505	176943542	197	0.052631579
chr1	177189515	177189552	198	0.052631579
chr1	177190237	177190274	199	0.052631579
chr1	177345067	177345104	200	0.052631579

chr1	178060572	178060609	201	0.052631579
chr1	178133155	178133192	202	0.052631579
chr1	179178543	179178580	203	0.052631579
chr1	17924987	17925024	204	0.052631579
chr1	179294203	179294240	205	0.052631579
chr1	179611187	179611224	206	0.052631579
chr1	179662610	179662647	207	0.052631579
chr1	179856860	179856897	208	0.052631579
chr1	180261597	180261634	209	0.052631579
chr1	180402647	180402684	210	0.052631579
chr1	180621573	180621610	211	0.052631579
chr1	180622127	180622164	212	0.052631579
chr1	18066488	18066525	213	0.052631579
chr1	18121075	18121112	214	0.052631579
chr1	181337738	181337775	215	0.052631579
chr1	181346358	181346395	216	0.052631579
chr1	181413178	181413215	217	0.052631579
chr1	181507963	181508000	218	0.052631579
chr1	181785303	181785340	219	0.052631579
chr1	181878499	181878536	220	0.052631579
chr1	181910524	181910561	221	0.052631579
chr1	182287948	182287985	222	0.052631579
chr1	182702267	182702304	223	0.052631579
chr1	182766736	182766773	224	0.052631579
chr1	182890854	182890891	225	0.052631579
chr1	183008792	183008829	226	0.052631579
chr1	1832049	1832086	227	0.052631579
chr1	183420541	183420578	228	0.052631579
chr1	183891195	183891232	229	0.052631579
chr1	18458545	18458582	230	0.052631579
chr1	185066855	185066892	231	0.052631579
chr1	185105185	185105222	232	0.052631579
chr1	185158971	185159008	233	0.052631579
chr1	18521842	18521879	234	0.052631579
chr1	185452208	185452245	235	0.052631579
chr1	187321296	187321333	236	0.052631579
chr1	18840013	18840050	237	0.052631579
chr1	188408277	188408314	238	0.052631579
chr1	188470162	188470199	239	0.052631579
chr1	188690566	188690603	240	0.052631579
chr1	190141752	190141789	241	0.052631579
chr1	19024052	19024089	242	0.052631579
chr1	19113270	19113307	243	0.052631579
chr1	191134252	191134289	244	0.052631579
chr1	191231482	191231519	245	0.052631579
chr1	191386097	191386134	246	0.052631579
chr1	191910166	191910203	247	0.052631579
chr1	192187139	192187176	248	0.052631579
chr1	192646402	192646439	249	0.052631579
chr1	19285804	19285841	250	0.052631579

chr1	192966260	192966297	251	0.052631579
chr1	193175488	193175525	252	0.052631579
chr1	193198815	193198852	253	0.052631579
chr1	193353491	193353528	254	0.052631579
chr1	19456978	19457015	255	0.052631579
chr1	194591112	194591149	256	0.052631579
chr1	194629866	194629903	257	0.052631579
chr1	196139019	196139056	258	0.052631579
chr1	196362420	196362457	259	0.052631579
chr1	196437801	196437838	260	0.052631579
chr1	196568363	196568400	261	0.052631579
chr1	196776804	196776841	262	0.052631579
chr1	196845456	196845493	263	0.052631579
chr1	197001187	197001224	264	0.052631579
chr1	197171521	197171558	265	0.052631579
chr1	197818879	197818916	266	0.052631579
chr1	198066416	198066453	267	0.052631579
chr1	198256775	198256812	268	0.052631579
chr1	198295924	198295961	269	0.052631579
chr1	198550882	198550919	270	0.052631579
chr1	199134238	199134275	271	0.052631579
chr1	199278519	199278556	272	0.052631579
chr1	199300965	199301002	273	0.052631579
chr1	199347934	199347971	274	0.052631579
chr1	199909072	199909109	275	0.052631579
chr1	199909781	199909818	276	0.052631579
chr1	199948616	199948653	277	0.052631579
chr1	200292590	200292627	278	0.052631579
chr1	200333215	200333252	279	0.052631579
chr1	200379348	200379385	280	0.052631579
chr1	200517473	200517510	281	0.052631579
chr1	200810783	200810820	282	0.052631579
chr1	201003917	201003954	283	0.052631579
chr1	201084373	201084410	284	0.052631579
chr1	201313318	201313355	285	0.052631579
chr1	201314165	201314202	286	0.052631579
chr1	201321387	201321424	287	0.052631579
chr1	201817771	201817808	288	0.052631579
chr1	201862954	201862991	289	0.052631579
chr1	201892608	201892645	290	0.052631579
chr1	201906624	201906661	291	0.052631579
chr1	201918706	201918743	292	0.052631579
chr1	202064344	202064381	293	0.052631579
chr1	202365436	202365473	294	0.052631579
chr1	202503627	202503664	295	0.052631579
chr1	202696939	202696976	296	0.052631579
chr1	203337412	203337449	297	0.052631579
chr1	203538714	203538751	298	0.052631579
chr1	203765290	203765327	299	0.052631579
chr1	204852605	204852642	300	0.052631579

chr1	204971817	204971854	301	0.052631579
chr1	205591532	205591569	302	0.052631579
chr1	205615244	205615281	303	0.052631579
chr1	206196500	206196537	304	0.052631579
chr1	206279601	206279638	305	0.052631579
chr1	206282246	206282283	306	0.052631579
chr1	206868943	206868980	307	0.052631579
chr1	206869193	206869230	308	0.052631579
chr1	20694523	20694560	309	0.052631579
chr1	207183889	207183926	310	0.052631579
chr1	20752124	20752161	311	0.052631579
chr1	208484945	208484982	312	0.052631579
chr1	208530997	208531034	313	0.052631579
chr1	208773601	208773638	314	0.052631579
chr1	208923056	208923093	315	0.052631579
chr1	20903920	20903957	316	0.052631579
chr1	209356851	209356888	317	0.052631579
chr1	209843881	209843918	318	0.052631579
chr1	210020226	210020263	319	0.052631579
chr1	210702874	210702911	320	0.052631579
chr1	210991787	210991824	321	0.052631579
chr1	210992346	210992383	322	0.052631579
chr1	211212860	211212897	323	0.052631579
chr1	211491916	211491953	324	0.052631579
chr1	211636947	211636984	325	0.052631579
chr1	212600432	212600469	326	0.052631579
chr1	212616380	212616417	327	0.052631579
chr1	212740429	212740466	328	0.052631579
chr1	213033560	213033597	329	0.052631579
chr1	213110728	213110765	330	0.052631579
chr1	21319865	21319902	331	0.052631579
chr1	213372127	213372164	332	0.052631579
chr1	213715948	213715985	333	0.052631579
chr1	214293879	214293916	334	0.052631579
chr1	214988390	214988427	335	0.052631579
chr1	215123794	215123831	336	0.052631579
chr1	215154525	215154562	337	0.052631579
chr1	215644101	215644138	338	0.052631579
chr1	215829822	215829859	339	0.052631579
chr1	215835315	215835352	340	0.052631579
chr1	216021125	216021162	341	0.052631579
chr1	216247201	216247238	342	0.052631579
chr1	216350112	216350149	343	0.052631579
chr1	217308610	217308647	344	0.052631579
chr1	217486152	217486189	345	0.052631579
chr1	217650042	217650079	346	0.052631579
chr1	218168141	218168178	347	0.052631579
chr1	218860099	218860136	348	0.052631579
chr1	218930832	218930869	349	0.052631579
chr1	219254195	219254232	350	0.052631579

chr1	219404553	219404590	351	0.052631579
chr1	219414561	219414598	352	0.052631579
chr1	219913157	219913194	353	0.052631579
chr1	219926105	219926142	354	0.052631579
chr1	220310098	220310135	355	0.052631579
chr1	22133339	22133376	356	0.052631579
chr1	221468623	221468660	357	0.052631579
chr1	221604924	221604961	358	0.052631579
chr1	2218955	2218992	359	0.052631579
chr1	221999691	221999728	360	0.052631579
chr1	222026131	222026168	361	0.052631579
chr1	222031428	222031465	362	0.052631579
chr1	2227362	2227399	363	0.052631579
chr1	22294150	22294187	364	0.052631579
chr1	22294924	22294961	365	0.052631579
chr1	223040599	223040636	366	0.052631579
chr1	223588104	223588141	367	0.052631579
chr1	224324411	224324448	368	0.052631579
chr1	224327431	224327468	369	0.052631579
chr1	224432506	224432543	370	0.052631579
chr1	22445069	22445106	371	0.052631579
chr1	224492577	224492614	372	0.052631579
chr1	224541736	224541773	373	0.052631579
chr1	224563008	224563045	374	0.052631579
chr1	224759444	224759481	375	0.052631579
chr1	224781872	224781909	376	0.052631579
chr1	224850762	224850799	377	0.052631579
chr1	224898009	224898046	378	0.052631579
chr1	225220357	225220394	379	0.052631579
chr1	225342911	225342948	380	0.052631579
chr1	225642855	225642892	381	0.052631579
chr1	225800585	225800622	382	0.052631579
chr1	225902331	225902368	383	0.052631579
chr1	226157499	226157536	384	0.052631579
chr1	226313559	226313596	385	0.052631579
chr1	226572757	226572794	386	0.052631579
chr1	226676025	226676062	387	0.052631579
chr1	226679168	226679205	388	0.052631579
chr1	226679388	226679425	389	0.052631579
chr1	226741921	226741958	390	0.052631579
chr1	227551810	227551847	391	0.052631579
chr1	227789896	227789933	392	0.052631579
chr1	22792405	22792442	393	0.052631579
chr1	228193777	228193814	394	0.052631579
chr1	228272140	228272177	395	0.052631579
chr1	228343034	228343071	396	0.052631579
chr1	228625864	228625901	397	0.052631579
chr1	228629270	228629307	398	0.052631579
chr1	228867290	228867327	399	0.052631579
chr1	229068368	229068405	400	0.052631579

chr1	229191061	229191098	401	0.052631579
chr1	229891410	229891447	402	0.052631579
chr1	229984037	229984074	403	0.052631579
chr1	229984862	229984899	404	0.052631579
chr1	230451683	230451720	405	0.052631579
chr1	230751435	230751472	406	0.052631579
chr1	230840648	230840685	407	0.052631579
chr1	23107040	23107077	408	0.052631579
chr1	23113579	23113616	409	0.052631579
chr1	231597000	231597037	410	0.052631579
chr1	231658471	231658508	411	0.052631579
chr1	232551935	232551972	412	0.052631579
chr1	233024725	233024762	413	0.052631579
chr1	233071635	233071672	414	0.052631579
chr1	23343061	23343098	415	0.052631579
chr1	233597669	233597706	416	0.052631579
chr1	234235349	234235386	417	0.052631579
chr1	234254516	234254553	418	0.052631579
chr1	234281840	234281877	419	0.052631579
chr1	234516919	234516956	420	0.052631579
chr1	234719142	234719179	421	0.052631579
chr1	234946477	234946514	422	0.052631579
chr1	234957922	234957959	423	0.052631579
chr1	235018573	235018610	424	0.052631579
chr1	235046755	235046792	425	0.052631579
chr1	235294765	235294802	426	0.052631579
chr1	235333998	235334035	427	0.052631579
chr1	235365895	235365932	428	0.052631579
chr1	235366997	235367034	429	0.052631579
chr1	235440400	235440437	430	0.052631579
chr1	23561444	23561481	431	0.052631579
chr1	235625219	235625256	432	0.052631579
chr1	235896020	235896057	433	0.052631579
chr1	235928998	235929035	434	0.052631579
chr1	236042562	236042599	435	0.052631579
chr1	236043301	236043338	436	0.052631579
chr1	236100175	236100212	437	0.052631579
chr1	236278941	236278978	438	0.052631579
chr1	236372563	236372600	439	0.052631579
chr1	236504853	236504890	440	0.052631579
chr1	23669922	23669959	441	0.052631579
chr1	236852328	236852365	442	0.052631579
chr1	237583606	237583643	443	0.052631579
chr1	238002960	238002997	444	0.052631579
chr1	238322380	238322417	445	0.052631579
chr1	238323174	238323211	446	0.052631579
chr1	238880895	238880932	447	0.052631579
chr1	239144382	239144419	448	0.052631579
chr1	239289615	239289652	449	0.052631579
chr1	239467034	239467071	450	0.052631579

chr1	239557542	239557579	451	0.052631579
chr1	239792335	239792372	452	0.052631579
chr1	239793203	239793240	453	0.052631579
chr1	23989504	23989541	454	0.052631579
chr1	240567750	240567787	455	0.052631579
chr1	24058302	24058339	456	0.052631579
chr1	24094892	24094929	457	0.052631579
chr1	240969020	240969057	458	0.052631579
chr1	241096166	241096203	459	0.052631579
chr1	241096261	241096298	460	0.052631579
chr1	2412775	2412812	461	0.052631579
chr1	241492386	241492423	462	0.052631579
chr1	241493087	241493124	463	0.052631579
chr1	241623440	241623477	464	0.052631579
chr1	241624216	241624253	465	0.052631579
chr1	241943711	241943748	466	0.052631579
chr1	241962670	241962707	467	0.052631579
chr1	241970749	241970786	468	0.052631579
chr1	242283891	242283928	469	0.052631579
chr1	242368798	242368835	470	0.052631579
chr1	242430737	242430774	471	0.052631579
chr1	242883259	242883296	472	0.052631579
chr1	243259836	243259873	473	0.052631579
chr1	243746700	243746737	474	0.052631579
chr1	243918436	243918473	475	0.052631579
chr1	243990323	243990360	476	0.052631579
chr1	244652673	244652710	477	0.052631579
chr1	244898218	244898255	478	0.052631579
chr1	24535841	24535878	479	0.052631579
chr1	245386517	245386554	480	0.052631579
chr1	245902586	245902623	481	0.052631579
chr1	246183261	246183298	482	0.052631579
chr1	246643184	246643221	483	0.052631579
chr1	247040678	247040715	484	0.052631579
chr1	247067921	247067958	485	0.052631579
chr1	25629981	25630018	486	0.052631579
chr1	2570415	2570452	487	0.052631579
chr1	25875885	25875922	488	0.052631579
chr1	26111232	26111269	489	0.052631579
chr1	26843618	26843655	490	0.052631579
chr1	26981149	26981186	491	0.052631579
chr1	27062804	27062841	492	0.052631579
chr1	27195356	27195393	493	0.052631579
chr1	2763023	2763060	494	0.052631579
chr1	27784833	27784870	495	0.052631579
chr1	27898592	27898629	496	0.052631579
chr1	27986775	27986812	497	0.052631579
chr1	28153658	28153695	498	0.052631579
chr1	28435281	28435318	499	0.052631579
chr1	28467981	28468018	500	0.052631579

chr1	28586005	28586042	501	0.052631579
chr1	28941697	28941734	502	0.052631579
chr1	30426704	30426741	503	0.052631579
chr1	30459525	30459562	504	0.052631579
chr1	30491864	30491901	505	0.052631579
chr1	30763620	30763657	506	0.052631579
chr1	31436166	31436203	507	0.052631579
chr1	31648419	31648456	508	0.052631579
chr1	32488222	32488259	509	0.052631579
chr1	33106528	33106565	510	0.052631579
chr1	33514561	33514598	511	0.052631579
chr1	33580602	33580639	512	0.052631579
chr1	33667291	33667328	513	0.052631579
chr1	33757760	33757797	514	0.052631579
chr1	3382476	3382513	515	0.052631579
chr1	3404022	3404059	516	0.052631579
chr1	34075443	34075480	517	0.052631579
chr1	34076251	34076288	518	0.052631579
chr1	3412879	3412916	519	0.052631579
chr1	34371320	34371357	520	0.052631579
chr1	34512980	34513017	521	0.052631579
chr1	34736259	34736296	522	0.052631579
chr1	34836073	34836110	523	0.052631579
chr1	35023168	35023205	524	0.052631579
chr1	35103628	35103665	525	0.052631579
chr1	35771552	35771589	526	0.052631579
chr1	35772275	35772312	527	0.052631579
chr1	36132526	36132563	528	0.052631579
chr1	36145126	36145163	529	0.052631579
chr1	36152040	36152077	530	0.052631579
chr1	36160065	36160102	531	0.052631579
chr1	36293305	36293342	532	0.052631579
chr1	36949411	36949448	533	0.052631579
chr1	37039439	37039476	534	0.052631579
chr1	37083545	37083582	535	0.052631579
chr1	37183416	37183453	536	0.052631579
chr1	37384445	37384482	537	0.052631579
chr1	3780529	3780566	538	0.052631579
chr1	38015209	38015246	539	0.052631579
chr1	38282124	38282161	540	0.052631579
chr1	38354031	38354068	541	0.052631579
chr1	38480428	38480465	542	0.052631579
chr1	38730824	38730861	543	0.052631579
chr1	39120675	39120712	544	0.052631579
chr1	39558094	39558131	545	0.052631579
chr1	40201375	40201412	546	0.052631579
chr1	40347667	40347704	547	0.052631579
chr1	40543064	40543101	548	0.052631579
chr1	40734017	40734054	549	0.052631579
chr1	41007159	41007196	550	0.052631579

chr1	41058433	41058470	551	0.052631579
chr1	41763078	41763115	552	0.052631579
chr1	42037908	42037945	553	0.052631579
chr1	42047701	42047738	554	0.052631579
chr1	42212056	42212093	555	0.052631579
chr1	42384157	42384194	556	0.052631579
chr1	42384885	42384922	557	0.052631579
chr1	42414874	42414911	558	0.052631579
chr1	42881336	42881373	559	0.052631579
chr1	4359640	4359677	560	0.052631579
chr1	43681208	43681245	561	0.052631579
chr1	4377287	4377324	562	0.052631579
chr1	43851047	43851084	563	0.052631579
chr1	44246835	44246872	564	0.052631579
chr1	44262821	44262858	565	0.052631579
chr1	44556715	44556752	566	0.052631579
chr1	44738952	44738989	567	0.052631579
chr1	44833666	44833703	568	0.052631579
chr1	44842958	44842995	569	0.052631579
chr1	44951778	44951815	570	0.052631579
chr1	45569582	45569619	571	0.052631579
chr1	4558325	4558362	572	0.052631579
chr1	45757653	45757690	573	0.052631579
chr1	45845637	45845674	574	0.052631579
chr1	45892951	45892988	575	0.052631579
chr1	46095613	46095650	576	0.052631579
chr1	4614712	4614749	577	0.052631579
chr1	46262074	46262111	578	0.052631579
chr1	46267164	46267201	579	0.052631579
chr1	47797186	47797223	580	0.052631579
chr1	48013518	48013555	581	0.052631579
chr1	48232655	48232692	582	0.052631579
chr1	49113827	49113864	583	0.052631579
chr1	49414270	49414307	584	0.052631579
chr1	50227074	50227111	585	0.052631579
chr1	50367589	50367626	586	0.052631579
chr1	50568733	50568770	587	0.052631579
chr1	50670011	50670048	588	0.052631579
chr1	51483028	51483065	589	0.052631579
chr1	5151495	5151532	590	0.052631579
chr1	5152311	5152348	591	0.052631579
chr1	51604703	51604740	592	0.052631579
chr1	51719765	51719802	593	0.052631579
chr1	51720452	51720489	594	0.052631579
chr1	52792071	52792108	595	0.052631579
chr1	52830201	52830238	596	0.052631579
chr1	53115405	53115442	597	0.052631579
chr1	53500408	53500445	598	0.052631579
chr1	53900205	53900242	599	0.052631579
chr1	53924601	53924638	600	0.052631579

chr1	55110828	55110865	601	0.052631579
chr1	55391301	55391338	602	0.052631579
chr1	55448232	55448269	603	0.052631579
chr1	55553362	55553399	604	0.052631579
chr1	56408200	56408237	605	0.052631579
chr1	56417017	56417054	606	0.052631579
chr1	56417713	56417750	607	0.052631579
chr1	56463061	56463098	608	0.052631579
chr1	56624773	56624810	609	0.052631579
chr1	57307650	57307687	610	0.052631579
chr1	57586210	57586247	611	0.052631579
chr1	57604468	57604505	612	0.052631579
chr1	57755743	57755780	613	0.052631579
chr1	57759686	57759723	614	0.052631579
chr1	5784684	5784721	615	0.052631579
chr1	58086822	58086859	616	0.052631579
chr1	58770394	58770431	617	0.052631579
chr1	58858384	58858421	618	0.052631579
chr1	58869264	58869301	619	0.052631579
chr1	59022448	59022485	620	0.052631579
chr1	59032925	59032962	621	0.052631579
chr1	59281896	59281933	622	0.052631579
chr1	5963787	5963824	623	0.052631579
chr1	60062190	60062227	624	0.052631579
chr1	60666883	60666920	625	0.052631579
chr1	60998511	60998548	626	0.052631579
chr1	61191170	61191207	627	0.052631579
chr1	61246071	61246108	628	0.052631579
chr1	6131997	6132034	629	0.052631579
chr1	61501479	61501516	630	0.052631579
chr1	62496823	62496860	631	0.052631579
chr1	62685746	62685783	632	0.052631579
chr1	62773784	62773821	633	0.052631579
chr1	63155477	63155514	634	0.052631579
chr1	63178502	63178539	635	0.052631579
chr1	63216992	63217029	636	0.052631579
chr1	63492914	63492951	637	0.052631579
chr1	63493661	63493698	638	0.052631579
chr1	63556265	63556302	639	0.052631579
chr1	63859129	63859166	640	0.052631579
chr1	6491640	6491677	641	0.052631579
chr1	65836230	65836267	642	0.052631579
chr1	6584917	6584954	643	0.052631579
chr1	66151971	66152008	644	0.052631579
chr1	66152523	66152560	645	0.052631579
chr1	66955505	66955542	646	0.052631579
chr1	67384898	67384935	647	0.052631579
chr1	67524142	67524179	648	0.052631579
chr1	6786157	6786194	649	0.052631579
chr1	67947292	67947329	650	0.052631579

chr1	6880435	6880472	651	0.052631579
chr1	69255199	69255236	652	0.052631579
chr1	69424768	69424805	653	0.052631579
chr1	69502417	69502454	654	0.052631579
chr1	69709158	69709195	655	0.052631579
chr1	69717960	69717997	656	0.052631579
chr1	70031225	70031262	657	0.052631579
chr1	70390159	70390196	658	0.052631579
chr1	71787088	71787125	659	0.052631579
chr1	73812198	73812235	660	0.052631579
chr1	74250347	74250384	661	0.052631579
chr1	74872271	74872308	662	0.052631579
chr1	75178421	75178458	663	0.052631579
chr1	75380544	75380581	664	0.052631579
chr1	7617575	7617612	665	0.052631579
chr1	76324240	76324277	666	0.052631579
chr1	76512366	76512403	667	0.052631579
chr1	77157118	77157155	668	0.052631579
chr1	77439297	77439334	669	0.052631579
chr1	78237497	78237534	670	0.052631579
chr1	7967663	7967700	671	0.052631579
chr1	80102841	80102878	672	0.052631579
chr1	80455129	80455166	673	0.052631579
chr1	80455297	80455334	674	0.052631579
chr1	80475374	80475411	675	0.052631579
chr1	80592142	80592179	676	0.052631579
chr1	80684533	80684570	677	0.052631579
chr1	81122380	81122417	678	0.052631579
chr1	81147401	81147438	679	0.052631579
chr1	81157226	81157263	680	0.052631579
chr1	81310599	81310636	681	0.052631579
chr1	81422542	81422579	682	0.052631579
chr1	82289093	82289130	683	0.052631579
chr1	82736929	82736966	684	0.052631579
chr1	83952737	83952774	685	0.052631579
chr1	84589933	84589970	686	0.052631579
chr1	84750872	84750909	687	0.052631579
chr1	84925612	84925649	688	0.052631579
chr1	84926681	84926718	689	0.052631579
chr1	85131961	85131998	690	0.052631579
chr1	85132538	85132575	691	0.052631579
chr1	85164948	85164985	692	0.052631579
chr1	85495746	85495783	693	0.052631579
chr1	85498820	85498857	694	0.052631579
chr1	86563537	86563574	695	0.052631579
chr1	86799707	86799744	696	0.052631579
chr1	8700653	8700690	697	0.052631579
chr1	87566442	87566479	698	0.052631579
chr1	87571896	87571933	699	0.052631579
chr1	87580076	87580113	700	0.052631579

chr1	87605935	87605972	701	0.052631579
chr1	87640541	87640578	702	0.052631579
chr1	87981495	87981532	703	0.052631579
chr1	88249073	88249110	704	0.052631579
chr1	88465228	88465265	705	0.052631579
chr1	88799790	88799827	706	0.052631579
chr1	89237191	89237228	707	0.052631579
chr1	89620848	89620885	708	0.052631579
chr1	89778429	89778466	709	0.052631579
chr1	89839099	89839136	710	0.052631579
chr1	90305667	90305704	711	0.052631579
chr1	90941806	90941843	712	0.052631579
chr1	91246802	91246839	713	0.052631579
chr1	92218001	92218038	714	0.052631579
chr1	92303891	92303928	715	0.052631579
chr1	92353293	92353330	716	0.052631579
chr1	92467510	92467547	717	0.052631579
chr1	92714286	92714323	718	0.052631579
chr1	9271896	9271933	719	0.052631579
chr1	9272131	9272168	720	0.052631579
chr1	92915608	92915645	721	0.052631579
chr1	93081619	93081656	722	0.052631579
chr1	93198430	93198467	723	0.052631579
chr1	93199237	93199274	724	0.052631579
chr1	93428804	93428841	725	0.052631579
chr1	93791300	93791337	726	0.052631579
chr1	93826038	93826075	727	0.052631579
chr1	94013311	94013348	728	0.052631579
chr1	9408000	9408037	729	0.052631579
chr1	95163896	95163933	730	0.052631579
chr1	95930902	95930939	731	0.052631579
chr1	96326010	96326047	732	0.052631579
chr1	96753673	96753710	733	0.052631579
chr1	96754366	96754403	734	0.052631579
chr1	9857693	9857730	735	0.052631579
chr1	9897552	9897589	736	0.052631579
chr1	99966285	99966322	737	0.052631579
chr1	99967120	99967157	738	0.052631579
chr1	115325493	115325555	739	0.063492063
chr1	142129159	142129219	740	0.06557377
chr1	197867868	197867926	741	0.06779661
chr1	33494672	33494712	742	0.073170732
chr1	100125271	100125310	743	0.075
chr1	102369063	102369102	744	0.075
chr1	103158486	103158525	745	0.075
chr1	107401010	107401049	746	0.075
chr1	10779360	10779399	747	0.075
chr1	109356284	109356323	748	0.075
chr1	10936000	10936039	749	0.075
chr1	109613763	109613802	750	0.075

chr1	11009421	11009460	751	0.075
chr1	110392113	110392152	752	0.075
chr1	110839160	110839199	753	0.075
chr1	111765567	111765606	754	0.075
chr1	112087543	112087582	755	0.075
chr1	112250579	112250618	756	0.075
chr1	11297303	11297342	757	0.075
chr1	113544013	113544052	758	0.075
chr1	113998235	113998274	759	0.075
chr1	114068998	114069037	760	0.075
chr1	114667602	114667641	761	0.075
chr1	115002553	115002592	762	0.075
chr1	115511224	115511263	763	0.075
chr1	115543741	115543780	764	0.075
chr1	116267097	116267136	765	0.075
chr1	116334512	116334551	766	0.075
chr1	116804800	116804839	767	0.075
chr1	117465607	117465646	768	0.075
chr1	117869491	117869530	769	0.075
chr1	118179299	118179338	770	0.075
chr1	118819192	118819231	771	0.075
chr1	119294428	119294467	772	0.075
chr1	119841074	119841113	773	0.075
chr1	119959079	119959118	774	0.075
chr1	1213310	1213349	775	0.075
chr1	12455794	12455833	776	0.075
chr1	12531090	12531129	777	0.075
chr1	13997818	13997857	778	0.075
chr1	14224935	14224974	779	0.075
chr1	143564639	143564678	780	0.075
chr1	143827107	143827146	781	0.075
chr1	144150956	144150995	782	0.075
chr1	144188278	144188317	783	0.075
chr1	146294215	146294254	784	0.075
chr1	14710679	14710718	785	0.075
chr1	14787378	14787417	786	0.075
chr1	148450447	148450486	787	0.075
chr1	150111211	150111250	788	0.075
chr1	150202041	150202080	789	0.075
chr1	150264146	150264185	790	0.075
chr1	151015618	151015657	791	0.075
chr1	151852171	151852210	792	0.075
chr1	151901327	151901366	793	0.075
chr1	151918903	151918942	794	0.075
chr1	15262005	15262044	795	0.075
chr1	15305632	15305671	796	0.075
chr1	153368342	153368381	797	0.075
chr1	153521115	153521154	798	0.075
chr1	153635720	153635759	799	0.075
chr1	154287097	154287136	800	0.075

chr1	154495447	154495486	801	0.075
chr1	154693360	154693399	802	0.075
chr1	154899808	154899847	803	0.075
chr1	155073226	155073265	804	0.075
chr1	155242735	155242774	805	0.075
chr1	15531963	15532002	806	0.075
chr1	155464765	155464804	807	0.075
chr1	157429031	157429070	808	0.075
chr1	158455270	158455309	809	0.075
chr1	158531648	158531687	810	0.075
chr1	158994412	158994451	811	0.075
chr1	15930610	15930649	812	0.075
chr1	163153183	163153222	813	0.075
chr1	163508844	163508883	814	0.075
chr1	16446652	16446691	815	0.075
chr1	164564573	164564612	816	0.075
chr1	164977758	164977797	817	0.075
chr1	165628405	165628444	818	0.075
chr1	166026427	166026466	819	0.075
chr1	166292974	166293013	820	0.075
chr1	16658461	16658500	821	0.075
chr1	166624831	166624870	822	0.075
chr1	166857435	166857474	823	0.075
chr1	167365918	167365957	824	0.075
chr1	167936351	167936390	825	0.075
chr1	16823527	16823566	826	0.075
chr1	168827094	168827133	827	0.075
chr1	169300027	169300066	828	0.075
chr1	169494118	169494157	829	0.075
chr1	169891223	169891262	830	0.075
chr1	170231809	170231848	831	0.075
chr1	170660101	170660140	832	0.075
chr1	171164854	171164893	833	0.075
chr1	1711792	1711831	834	0.075
chr1	173306879	173306918	835	0.075
chr1	173693040	173693079	836	0.075
chr1	173734580	173734619	837	0.075
chr1	173942693	173942732	838	0.075
chr1	175302792	175302831	839	0.075
chr1	176074907	176074946	840	0.075
chr1	176896650	176896689	841	0.075
chr1	177089288	177089327	842	0.075
chr1	177209717	177209756	843	0.075
chr1	178183317	178183356	844	0.075
chr1	178578546	178578585	845	0.075
chr1	180209043	180209082	846	0.075
chr1	180652126	180652165	847	0.075
chr1	180887877	180887916	848	0.075
chr1	181118989	181119028	849	0.075
chr1	181416409	181416448	850	0.075

chr1	181456629	181456668	851	0.075
chr1	181487491	181487530	852	0.075
chr1	181505163	181505202	853	0.075
chr1	182273558	182273597	854	0.075
chr1	186342198	186342237	855	0.075
chr1	187208270	187208309	856	0.075
chr1	190308835	190308874	857	0.075
chr1	192361442	192361481	858	0.075
chr1	194441349	194441388	859	0.075
chr1	19579154	19579193	860	0.075
chr1	19641114	19641153	861	0.075
chr1	198384294	198384333	862	0.075
chr1	199591901	199591940	863	0.075
chr1	199695880	199695919	864	0.075
chr1	199839204	199839243	865	0.075
chr1	199966581	199966620	866	0.075
chr1	200070774	200070813	867	0.075
chr1	201180835	201180874	868	0.075
chr1	201401501	201401540	869	0.075
chr1	201561461	201561500	870	0.075
chr1	201583447	201583486	871	0.075
chr1	201976792	201976831	872	0.075
chr1	202642026	202642065	873	0.075
chr1	202765506	202765545	874	0.075
chr1	202891966	202892005	875	0.075
chr1	203294880	203294919	876	0.075
chr1	203506942	203506981	877	0.075
chr1	203605884	203605923	878	0.075
chr1	204376523	204376562	879	0.075
chr1	205204120	205204159	880	0.075
chr1	205955121	205955160	881	0.075
chr1	206335081	206335120	882	0.075
chr1	207468067	207468106	883	0.075
chr1	207983362	207983401	884	0.075
chr1	208052095	208052134	885	0.075
chr1	20878030	20878069	886	0.075
chr1	208968246	208968285	887	0.075
chr1	210499528	210499567	888	0.075
chr1	210695646	210695685	889	0.075
chr1	210940765	210940804	890	0.075
chr1	21108252	21108291	891	0.075
chr1	213476460	213476499	892	0.075
chr1	213663997	213664036	893	0.075
chr1	214204500	214204539	894	0.075
chr1	214284292	214284331	895	0.075
chr1	214748525	214748564	896	0.075
chr1	214763911	214763950	897	0.075
chr1	216289754	216289793	898	0.075
chr1	216473074	216473113	899	0.075
chr1	216788148	216788187	900	0.075

chr1	217529978	217530017	901	0.075
chr1	217794281	217794320	902	0.075
chr1	2193717	2193756	903	0.075
chr1	220035126	220035165	904	0.075
chr1	22051944	22051983	905	0.075
chr1	220891799	220891838	906	0.075
chr1	221126198	221126237	907	0.075
chr1	221493198	221493237	908	0.075
chr1	221569180	221569219	909	0.075
chr1	222882552	222882591	910	0.075
chr1	223662748	223662787	911	0.075
chr1	224406746	224406785	912	0.075
chr1	224977834	224977873	913	0.075
chr1	225322973	225323012	914	0.075
chr1	225869903	225869942	915	0.075
chr1	226615593	226615632	916	0.075
chr1	227733505	227733544	917	0.075
chr1	228580618	228580657	918	0.075
chr1	229411534	229411573	919	0.075
chr1	230223819	230223858	920	0.075
chr1	230472980	230473019	921	0.075
chr1	231381628	231381667	922	0.075
chr1	231460332	231460371	923	0.075
chr1	231682763	231682802	924	0.075
chr1	234625293	234625332	925	0.075
chr1	234865672	234865711	926	0.075
chr1	238374083	238374122	927	0.075
chr1	240188225	240188264	928	0.075
chr1	241579088	241579127	929	0.075
chr1	242374779	242374818	930	0.075
chr1	242401765	242401804	931	0.075
chr1	243085485	243085524	932	0.075
chr1	244319840	244319879	933	0.075
chr1	244375422	244375461	934	0.075
chr1	244385105	244385144	935	0.075
chr1	244521080	244521119	936	0.075
chr1	244743683	244743722	937	0.075
chr1	246590268	246590307	938	0.075
chr1	246937251	246937290	939	0.075
chr1	246969623	246969662	940	0.075
chr1	25111316	25111355	941	0.075
chr1	25431221	25431260	942	0.075
chr1	26017213	26017252	943	0.075
chr1	26379977	26380016	944	0.075
chr1	26480521	26480560	945	0.075
chr1	26760171	26760210	946	0.075
chr1	27844278	27844317	947	0.075
chr1	28471414	28471453	948	0.075
chr1	29422309	29422348	949	0.075
chr1	29441380	29441419	950	0.075

chr1	29445522	29445561	951	0.075
chr1	30995617	30995656	952	0.075
chr1	31826956	31826995	953	0.075
chr1	31921387	31921426	954	0.075
chr1	32036184	32036223	955	0.075
chr1	32065450	32065489	956	0.075
chr1	32420872	32420911	957	0.075
chr1	33412318	33412357	958	0.075
chr1	3433712	3433751	959	0.075
chr1	34525210	34525249	960	0.075
chr1	34849041	34849080	961	0.075
chr1	34958045	34958084	962	0.075
chr1	3532227	3532266	963	0.075
chr1	35697196	35697235	964	0.075
chr1	3596568	3596607	965	0.075
chr1	35966096	35966135	966	0.075
chr1	36412108	36412147	967	0.075
chr1	36521660	36521699	968	0.075
chr1	36560685	36560724	969	0.075
chr1	36822597	36822636	970	0.075
chr1	36976100	36976139	971	0.075
chr1	37153114	37153153	972	0.075
chr1	37719990	37720029	973	0.075
chr1	37844364	37844403	974	0.075
chr1	38152683	38152722	975	0.075
chr1	3825545	3825584	976	0.075
chr1	38816873	38816912	977	0.075
chr1	38911163	38911202	978	0.075
chr1	39054366	39054405	979	0.075
chr1	39729999	39730038	980	0.075
chr1	40309179	40309218	981	0.075
chr1	4112343	4112382	982	0.075
chr1	41284794	41284833	983	0.075
chr1	41620004	41620043	984	0.075
chr1	4197771	4197810	985	0.075
chr1	4232133	4232172	986	0.075
chr1	42420445	42420484	987	0.075
chr1	42944238	42944277	988	0.075
chr1	42978979	42979018	989	0.075
chr1	43068839	43068878	990	0.075
chr1	44012835	44012874	991	0.075
chr1	44194652	44194691	992	0.075
chr1	44889368	44889407	993	0.075
chr1	45247636	45247675	994	0.075
chr1	4619107	4619146	995	0.075
chr1	46524726	46524765	996	0.075
chr1	47771659	47771698	997	0.075
chr1	48481736	48481775	998	0.075
chr1	48908930	48908969	999	0.075
chr1	49011083	49011122	1000	0.075

chr1	49073186	49073225	1001	0.075
chr1	52215286	52215325	1002	0.075
chr1	5237643	5237682	1003	0.075
chr1	52763916	52763955	1004	0.075
chr1	53135911	53135950	1005	0.075
chr1	54545439	54545478	1006	0.075
chr1	54953156	54953195	1007	0.075
chr1	556918	556957	1008	0.075
chr1	56542176	56542215	1009	0.075
chr1	56612802	56612841	1010	0.075
chr1	57282986	57283025	1011	0.075
chr1	58358279	58358318	1012	0.075
chr1	59685062	59685101	1013	0.075
chr1	59905253	59905292	1014	0.075
chr1	62521202	62521241	1015	0.075
chr1	63093472	63093511	1016	0.075
chr1	64049256	64049295	1017	0.075
chr1	64728104	64728143	1018	0.075
chr1	64953200	64953239	1019	0.075
chr1	65296723	65296762	1020	0.075
chr1	65306342	65306381	1021	0.075
chr1	65753369	65753408	1022	0.075
chr1	6636641	6636680	1023	0.075
chr1	6767713	6767752	1024	0.075
chr1	67847282	67847321	1025	0.075
chr1	70956973	70957012	1026	0.075
chr1	71834371	71834410	1027	0.075
chr1	73369714	73369753	1028	0.075
chr1	73718547	73718586	1029	0.075
chr1	75565477	75565516	1030	0.075
chr1	76024672	76024711	1031	0.075
chr1	76032372	76032411	1032	0.075
chr1	76507812	76507851	1033	0.075
chr1	76701116	76701155	1034	0.075
chr1	76709386	76709425	1035	0.075
chr1	77331574	77331613	1036	0.075
chr1	77852504	77852543	1037	0.075
chr1	78012367	78012406	1038	0.075
chr1	80603994	80604033	1039	0.075
chr1	81365504	81365543	1040	0.075
chr1	8187363	8187402	1041	0.075
chr1	8266786	8266825	1042	0.075
chr1	85045600	85045639	1043	0.075
chr1	85952323	85952362	1044	0.075
chr1	87363401	87363440	1045	0.075
chr1	87538476	87538515	1046	0.075
chr1	87549134	87549173	1047	0.075
chr1	87550167	87550206	1048	0.075
chr1	87743510	87743549	1049	0.075
chr1	89126959	89126998	1050	0.075

chr1	91073358	91073397	1051	0.075
chr1	91079301	91079340	1052	0.075
chr1	91300059	91300098	1053	0.075
chr1	93820935	93820974	1054	0.075
chr1	939681	939720	1055	0.075
chr1	94003043	94003082	1056	0.075
chr1	94733802	94733841	1057	0.075
chr1	95310896	95310935	1058	0.075
chr1	9559232	9559271	1059	0.075
chr1	9613835	9613874	1060	0.075
chr1	96762082	96762121	1061	0.075
chr1	9791736	9791775	1062	0.075
chr1	9806561	9806600	1063	0.075
chr1	91861391	91861443	1064	0.075471698
chr1	243765172	243765210	1065	0.076923077
chr1	93961469	93961518	1066	0.08
chr1	1258480	1258522	1067	0.093023256
chr1	180847694	180847734	1068	0.097560976
chr1	51945754	51945794	1069	0.097560976
chr1	92008345	92008385	1070	0.097560976
chr1	197541127	197541166	1071	0.1
chr1	158589510	158589556	1072	0.106382979
chr1	559781	559823	1073	0.11627907
chr10	14012278	14012393	1074	0.034482759
chr10	34308681	34308793	1075	0.03539823
chr10	72275421	72275530	1076	0.036363636
chr10	119620597	119620676	1077	0.0375
chr10	7881239	7881330	1078	0.043478261
chr10	88003089	88003156	1079	0.044117647
chr10	60428810	60428892	1080	0.048192771
chr10	402268	402307	1081	0.05
chr10	74247222	74247300	1082	0.050632911
chr10	28948506	28948563	1083	0.051724138
chr10	100002107	100002144	1084	0.052631579
chr10	100276179	100276216	1085	0.052631579
chr10	10047230	10047267	1086	0.052631579
chr10	10051509	10051546	1087	0.052631579
chr10	100642493	100642530	1088	0.052631579
chr10	101083925	101083962	1089	0.052631579
chr10	101182797	101182834	1090	0.052631579
chr10	101363415	101363452	1091	0.052631579
chr10	101534483	101534520	1092	0.052631579
chr10	101636130	101636167	1093	0.052631579
chr10	101934861	101934898	1094	0.052631579
chr10	102101086	102101123	1095	0.052631579
chr10	102101823	102101860	1096	0.052631579
chr10	102362740	102362777	1097	0.052631579
chr10	102432598	102432635	1098	0.052631579
chr10	102433418	102433455	1099	0.052631579
chr10	102437664	102437701	1100	0.052631579

chr10	102704355	102704392	1101	0.052631579
chr10	103307664	103307701	1102	0.052631579
chr10	103432932	103432969	1103	0.052631579
chr10	103557554	103557591	1104	0.052631579
chr10	103587590	103587627	1105	0.052631579
chr10	103588220	103588257	1106	0.052631579
chr10	103653338	103653375	1107	0.052631579
chr10	104060553	104060590	1108	0.052631579
chr10	104220569	104220606	1109	0.052631579
chr10	104416098	104416135	1110	0.052631579
chr10	104862957	104862994	1111	0.052631579
chr10	10489399	10489436	1112	0.052631579
chr10	104953014	104953051	1113	0.052631579
chr10	105457602	105457639	1114	0.052631579
chr10	105740517	105740554	1115	0.052631579
chr10	105840305	105840342	1116	0.052631579
chr10	105848434	105848471	1117	0.052631579
chr10	106058038	106058075	1118	0.052631579
chr10	106058796	106058833	1119	0.052631579
chr10	106062207	106062244	1120	0.052631579
chr10	106074200	106074237	1121	0.052631579
chr10	106079054	106079091	1122	0.052631579
chr10	106079815	106079852	1123	0.052631579
chr10	106891909	106891946	1124	0.052631579
chr10	106915728	106915765	1125	0.052631579
chr10	107402749	107402786	1126	0.052631579
chr10	107631084	107631121	1127	0.052631579
chr10	107652783	107652820	1128	0.052631579
chr10	108759541	108759578	1129	0.052631579
chr10	109285131	109285168	1130	0.052631579
chr10	10955375	10955412	1131	0.052631579
chr10	111763609	111763646	1132	0.052631579
chr10	111888760	111888797	1133	0.052631579
chr10	112208839	112208876	1134	0.052631579
chr10	112484115	112484152	1135	0.052631579
chr10	11301635	11301672	1136	0.052631579
chr10	113914329	113914366	1137	0.052631579
chr10	114287424	114287461	1138	0.052631579
chr10	114524314	114524351	1139	0.052631579
chr10	114890884	114890921	1140	0.052631579
chr10	115075584	115075621	1141	0.052631579
chr10	115098367	115098404	1142	0.052631579
chr10	115459309	115459346	1143	0.052631579
chr10	115724445	115724482	1144	0.052631579
chr10	116405307	116405344	1145	0.052631579
chr10	116718382	116718419	1146	0.052631579
chr10	11765594	11765631	1147	0.052631579
chr10	118090059	118090096	1148	0.052631579
chr10	118215416	118215453	1149	0.052631579
chr10	118374073	118374110	1150	0.052631579

chr10	118651444	118651481	1151	0.052631579
chr10	1197300	1197337	1152	0.052631579
chr10	1198071	1198108	1153	0.052631579
chr10	120606092	120606129	1154	0.052631579
chr10	120662433	120662470	1155	0.052631579
chr10	121814625	121814662	1156	0.052631579
chr10	122078840	122078877	1157	0.052631579
chr10	122417213	122417250	1158	0.052631579
chr10	12255793	12255830	1159	0.052631579
chr10	122905446	122905483	1160	0.052631579
chr10	123254459	123254496	1161	0.052631579
chr10	123314266	123314303	1162	0.052631579
chr10	12362076	12362113	1163	0.052631579
chr10	123674249	123674286	1164	0.052631579
chr10	124050537	124050574	1165	0.052631579
chr10	124123900	124123937	1166	0.052631579
chr10	125063341	125063378	1167	0.052631579
chr10	125064099	125064136	1168	0.052631579
chr10	125163179	125163216	1169	0.052631579
chr10	125195266	125195303	1170	0.052631579
chr10	125436553	125436590	1171	0.052631579
chr10	125606820	125606857	1172	0.052631579
chr10	125693889	125693926	1173	0.052631579
chr10	125807720	125807757	1174	0.052631579
chr10	126321637	126321674	1175	0.052631579
chr10	126621076	126621113	1176	0.052631579
chr10	126744166	126744203	1177	0.052631579
chr10	126746567	126746604	1178	0.052631579
chr10	127276831	127276868	1179	0.052631579
chr10	12771124	12771161	1180	0.052631579
chr10	12797570	12797607	1181	0.052631579
chr10	128172297	128172334	1182	0.052631579
chr10	128215699	128215736	1183	0.052631579
chr10	128411424	128411461	1184	0.052631579
chr10	128686344	128686381	1185	0.052631579
chr10	129060240	129060277	1186	0.052631579
chr10	129651958	129651995	1187	0.052631579
chr10	130003025	130003062	1188	0.052631579
chr10	130159150	130159187	1189	0.052631579
chr10	131090782	131090819	1190	0.052631579
chr10	131210764	131210801	1191	0.052631579
chr10	131542100	131542137	1192	0.052631579
chr10	131569851	131569888	1193	0.052631579
chr10	131593101	131593138	1194	0.052631579
chr10	13178566	13178603	1195	0.052631579
chr10	131847991	131848028	1196	0.052631579
chr10	132390769	132390806	1197	0.052631579
chr10	132807322	132807359	1198	0.052631579
chr10	13302170	13302207	1199	0.052631579
chr10	133117183	133117220	1200	0.052631579

chr10	133212903	133212940	1201	0.052631579
chr10	133863813	133863850	1202	0.052631579
chr10	133874225	133874262	1203	0.052631579
chr10	133889252	133889289	1204	0.052631579
chr10	133928228	133928265	1205	0.052631579
chr10	134167513	134167550	1206	0.052631579
chr10	134198530	134198567	1207	0.052631579
chr10	134354966	134355003	1208	0.052631579
chr10	13487735	13487772	1209	0.052631579
chr10	135202383	135202420	1210	0.052631579
chr10	13993230	13993267	1211	0.052631579
chr10	13993531	13993568	1212	0.052631579
chr10	15272030	15272067	1213	0.052631579
chr10	15433479	15433516	1214	0.052631579
chr10	15610505	15610542	1215	0.052631579
chr10	1578821	1578858	1216	0.052631579
chr10	16353608	16353645	1217	0.052631579
chr10	17311305	17311342	1218	0.052631579
chr10	17315626	17315663	1219	0.052631579
chr10	17647853	17647890	1220	0.052631579
chr10	1803972	1804009	1221	0.052631579
chr10	18451454	18451491	1222	0.052631579
chr10	19283901	19283938	1223	0.052631579
chr10	19657526	19657563	1224	0.052631579
chr10	20124591	20124628	1225	0.052631579
chr10	20672379	20672416	1226	0.052631579
chr10	21846679	21846716	1227	0.052631579
chr10	22371202	22371239	1228	0.052631579
chr10	22668098	22668135	1229	0.052631579
chr10	23610115	23610152	1230	0.052631579
chr10	23640323	23640360	1231	0.052631579
chr10	2403985	2404022	1232	0.052631579
chr10	24046074	24046111	1233	0.052631579
chr10	24704887	24704924	1234	0.052631579
chr10	25134882	25134919	1235	0.052631579
chr10	25307023	25307060	1236	0.052631579
chr10	25384181	25384218	1237	0.052631579
chr10	25433879	25433916	1238	0.052631579
chr10	26562123	26562160	1239	0.052631579
chr10	26562927	26562964	1240	0.052631579
chr10	26939485	26939522	1241	0.052631579
chr10	28758456	28758493	1242	0.052631579
chr10	29398790	29398827	1243	0.052631579
chr10	30002250	30002287	1244	0.052631579
chr10	30444791	30444828	1245	0.052631579
chr10	30445046	30445083	1246	0.052631579
chr10	30768921	30768958	1247	0.052631579
chr10	31114416	31114453	1248	0.052631579
chr10	31227044	31227081	1249	0.052631579
chr10	31462975	31463012	1250	0.052631579

chr10	3146867	3146904	1251	0.052631579
chr10	31759027	31759064	1252	0.052631579
chr10	31858072	31858109	1253	0.052631579
chr10	32027151	32027188	1254	0.052631579
chr10	32254151	32254188	1255	0.052631579
chr10	32517609	32517646	1256	0.052631579
chr10	32746765	32746802	1257	0.052631579
chr10	33708343	33708380	1258	0.052631579
chr10	34119879	34119916	1259	0.052631579
chr10	34500943	34500980	1260	0.052631579
chr10	35548399	35548436	1261	0.052631579
chr10	35967789	35967826	1262	0.052631579
chr10	36060128	36060165	1263	0.052631579
chr10	36060471	36060508	1264	0.052631579
chr10	36456985	36457022	1265	0.052631579
chr10	37045917	37045954	1266	0.052631579
chr10	37284212	37284249	1267	0.052631579
chr10	38161065	38161102	1268	0.052631579
chr10	38494770	38494807	1269	0.052631579
chr10	38681868	38681905	1270	0.052631579
chr10	38684949	38684986	1271	0.052631579
chr10	3894435	3894472	1272	0.052631579
chr10	3895075	3895112	1273	0.052631579
chr10	3986939	3986976	1274	0.052631579
chr10	404858	404895	1275	0.052631579
chr10	405147	405184	1276	0.052631579
chr10	42335048	42335085	1277	0.052631579
chr10	43011278	43011315	1278	0.052631579
chr10	43338239	43338276	1279	0.052631579
chr10	43379075	43379112	1280	0.052631579
chr10	4389212	4389249	1281	0.052631579
chr10	4389614	4389651	1282	0.052631579
chr10	44719326	44719363	1283	0.052631579
chr10	45076910	45076947	1284	0.052631579
chr10	45333443	45333480	1285	0.052631579
chr10	46420051	46420088	1286	0.052631579
chr10	46420718	46420755	1287	0.052631579
chr10	48275277	48275314	1288	0.052631579
chr10	48275940	48275977	1289	0.052631579
chr10	49926444	49926481	1290	0.052631579
chr10	50271660	50271697	1291	0.052631579
chr10	50377923	50377960	1292	0.052631579
chr10	50386447	50386484	1293	0.052631579
chr10	50573227	50573264	1294	0.052631579
chr10	51451271	51451308	1295	0.052631579
chr10	51789512	51789549	1296	0.052631579
chr10	52619812	52619849	1297	0.052631579
chr10	52705359	52705396	1298	0.052631579
chr10	52963864	52963901	1299	0.052631579
chr10	53862341	53862378	1300	0.052631579

chr10	54051731	54051768	1301	0.052631579
chr10	54311417	54311454	1302	0.052631579
chr10	55289129	55289166	1303	0.052631579
chr10	55939897	55939934	1304	0.052631579
chr10	56084148	56084185	1305	0.052631579
chr10	56345040	56345077	1306	0.052631579
chr10	57110158	57110195	1307	0.052631579
chr10	574792	574829	1308	0.052631579
chr10	57965850	57965887	1309	0.052631579
chr10	58100779	58100816	1310	0.052631579
chr10	58682900	58682937	1311	0.052631579
chr10	59290913	59290950	1312	0.052631579
chr10	59832464	59832501	1313	0.052631579
chr10	6007372	6007409	1314	0.052631579
chr10	60756865	60756902	1315	0.052631579
chr10	61234235	61234272	1316	0.052631579
chr10	61608426	61608463	1317	0.052631579
chr10	61635910	61635947	1318	0.052631579
chr10	61687983	61688020	1319	0.052631579
chr10	61910987	61911024	1320	0.052631579
chr10	62376409	62376446	1321	0.052631579
chr10	62431582	62431619	1322	0.052631579
chr10	63325507	63325544	1323	0.052631579
chr10	63476438	63476475	1324	0.052631579
chr10	6355387	6355424	1325	0.052631579
chr10	64435217	64435254	1326	0.052631579
chr10	6444767	6444804	1327	0.052631579
chr10	64615238	64615275	1328	0.052631579
chr10	64657002	64657039	1329	0.052631579
chr10	6508703	6508740	1330	0.052631579
chr10	6548217	6548254	1331	0.052631579
chr10	65620613	65620650	1332	0.052631579
chr10	65748829	65748866	1333	0.052631579
chr10	65749508	65749545	1334	0.052631579
chr10	6591349	6591386	1335	0.052631579
chr10	6657360	6657397	1336	0.052631579
chr10	67473616	67473653	1337	0.052631579
chr10	67494507	67494544	1338	0.052631579
chr10	67495024	67495061	1339	0.052631579
chr10	6822422	6822459	1340	0.052631579
chr10	68476044	68476081	1341	0.052631579
chr10	68980277	68980314	1342	0.052631579
chr10	69419255	69419292	1343	0.052631579
chr10	69807731	69807768	1344	0.052631579
chr10	70074300	70074337	1345	0.052631579
chr10	70924065	70924102	1346	0.052631579
chr10	71080213	71080250	1347	0.052631579
chr10	71251370	71251407	1348	0.052631579
chr10	71252153	71252190	1349	0.052631579
chr10	71553847	71553884	1350	0.052631579

chr10	71912790	71912827	1351	0.052631579
chr10	71913534	71913571	1352	0.052631579
chr10	72706804	72706841	1353	0.052631579
chr10	73276745	73276782	1354	0.052631579
chr10	73342204	73342241	1355	0.052631579
chr10	73545653	73545690	1356	0.052631579
chr10	73810715	73810752	1357	0.052631579
chr10	74158504	74158541	1358	0.052631579
chr10	74433288	74433325	1359	0.052631579
chr10	7444717	7444754	1360	0.052631579
chr10	74676836	74676873	1361	0.052631579
chr10	75732840	75732877	1362	0.052631579
chr10	76405311	76405348	1363	0.052631579
chr10	76519381	76519418	1364	0.052631579
chr10	76545119	76545156	1365	0.052631579
chr10	76640578	76640615	1366	0.052631579
chr10	76677915	76677952	1367	0.052631579
chr10	76808444	76808481	1368	0.052631579
chr10	76875722	76875759	1369	0.052631579
chr10	77318692	77318729	1370	0.052631579
chr10	77397212	77397249	1371	0.052631579
chr10	77455421	77455458	1372	0.052631579
chr10	78061277	78061314	1373	0.052631579
chr10	78171597	78171634	1374	0.052631579
chr10	78425271	78425308	1375	0.052631579
chr10	78683205	78683242	1376	0.052631579
chr10	78748967	78749004	1377	0.052631579
chr10	78749729	78749766	1378	0.052631579
chr10	78962358	78962395	1379	0.052631579
chr10	79348307	79348344	1380	0.052631579
chr10	79465302	79465339	1381	0.052631579
chr10	79485336	79485373	1382	0.052631579
chr10	79486045	79486082	1383	0.052631579
chr10	79627116	79627153	1384	0.052631579
chr10	79633372	79633409	1385	0.052631579
chr10	79998194	79998231	1386	0.052631579
chr10	80075246	80075283	1387	0.052631579
chr10	8062653	8062690	1388	0.052631579
chr10	80633716	80633753	1389	0.052631579
chr10	80674300	80674337	1390	0.052631579
chr10	8185545	8185582	1391	0.052631579
chr10	81921785	81921822	1392	0.052631579
chr10	82244223	82244260	1393	0.052631579
chr10	83069524	83069561	1394	0.052631579
chr10	83416613	83416650	1395	0.052631579
chr10	83542871	83542908	1396	0.052631579
chr10	83741344	83741381	1397	0.052631579
chr10	84090273	84090310	1398	0.052631579
chr10	84194886	84194923	1399	0.052631579
chr10	85361110	85361147	1400	0.052631579

chr10	85578548	85578585	1401	0.052631579
chr10	85618147	85618184	1402	0.052631579
chr10	85622203	85622240	1403	0.052631579
chr10	85700988	85701025	1404	0.052631579
chr10	85701442	85701479	1405	0.052631579
chr10	86112425	86112462	1406	0.052631579
chr10	8654857	8654894	1407	0.052631579
chr10	86629172	86629209	1408	0.052631579
chr10	87187575	87187612	1409	0.052631579
chr10	8742848	8742885	1410	0.052631579
chr10	87514226	87514263	1411	0.052631579
chr10	87660110	87660147	1412	0.052631579
chr10	8838682	8838719	1413	0.052631579
chr10	88714258	88714295	1414	0.052631579
chr10	88827523	88827560	1415	0.052631579
chr10	88963550	88963587	1416	0.052631579
chr10	8912270	8912307	1417	0.052631579
chr10	89296344	89296381	1418	0.052631579
chr10	89568231	89568268	1419	0.052631579
chr10	89753915	89753952	1420	0.052631579
chr10	89836745	89836782	1421	0.052631579
chr10	89877208	89877245	1422	0.052631579
chr10	89877902	89877939	1423	0.052631579
chr10	89988536	89988573	1424	0.052631579
chr10	90213881	90213918	1425	0.052631579
chr10	90214180	90214217	1426	0.052631579
chr10	90243299	90243336	1427	0.052631579
chr10	90260234	90260271	1428	0.052631579
chr10	90651454	90651491	1429	0.052631579
chr10	90651920	90651957	1430	0.052631579
chr10	90765653	90765690	1431	0.052631579
chr10	91671035	91671072	1432	0.052631579
chr10	91737422	91737459	1433	0.052631579
chr10	92332142	92332179	1434	0.052631579
chr10	92501705	92501742	1435	0.052631579
chr10	92789392	92789429	1436	0.052631579
chr10	9290987	9291024	1437	0.052631579
chr10	93268889	93268926	1438	0.052631579
chr10	93286983	93287020	1439	0.052631579
chr10	9339799	9339836	1440	0.052631579
chr10	94099553	94099590	1441	0.052631579
chr10	94225783	94225820	1442	0.052631579
chr10	94808001	94808038	1443	0.052631579
chr10	95080197	95080234	1444	0.052631579
chr10	95082956	95082993	1445	0.052631579
chr10	96484946	96484983	1446	0.052631579
chr10	96818449	96818486	1447	0.052631579
chr10	9705121	9705158	1448	0.052631579
chr10	97146080	97146117	1449	0.052631579
chr10	9719512	9719549	1450	0.052631579

chr10	98107371	98107408	1451	0.052631579
chr10	98423434	98423471	1452	0.052631579
chr10	98740014	98740051	1453	0.052631579
chr10	98803079	98803116	1454	0.052631579
chr10	98910067	98910104	1455	0.052631579
chr10	98913357	98913394	1456	0.052631579
chr10	98914141	98914178	1457	0.052631579
chr10	99134027	99134064	1458	0.052631579
chr10	99296578	99296615	1459	0.052631579
chr10	99555582	99555619	1460	0.052631579
chr10	99922719	99922756	1461	0.052631579
chr10	66037095	66037159	1462	0.061538462
chr10	75212112	75212181	1463	0.071428571
chr10	100048800	100048839	1464	0.075
chr10	100361298	100361337	1465	0.075
chr10	102112605	102112644	1466	0.075
chr10	102406349	102406388	1467	0.075
chr10	102473935	102473974	1468	0.075
chr10	102542571	102542610	1469	0.075
chr10	102737487	102737526	1470	0.075
chr10	102748561	102748600	1471	0.075
chr10	103374487	103374526	1472	0.075
chr10	103509612	103509651	1473	0.075
chr10	103544610	103544649	1474	0.075
chr10	104223518	104223557	1475	0.075
chr10	104238808	104238847	1476	0.075
chr10	104394398	104394437	1477	0.075
chr10	105716635	105716674	1478	0.075
chr10	106190570	106190609	1479	0.075
chr10	106702819	106702858	1480	0.075
chr10	106740188	106740227	1481	0.075
chr10	10770377	10770416	1482	0.075
chr10	10887317	10887356	1483	0.075
chr10	111957780	111957819	1484	0.075
chr10	113085704	113085743	1485	0.075
chr10	114154523	114154562	1486	0.075
chr10	11420602	11420641	1487	0.075
chr10	114853187	114853226	1488	0.075
chr10	114875476	114875515	1489	0.075
chr10	114901474	114901513	1490	0.075
chr10	114908193	114908232	1491	0.075
chr10	115586482	115586521	1492	0.075
chr10	115759561	115759600	1493	0.075
chr10	115852167	115852206	1494	0.075
chr10	11617674	11617713	1495	0.075
chr10	117745281	117745320	1496	0.075
chr10	118020426	118020465	1497	0.075
chr10	118556245	118556284	1498	0.075
chr10	118882598	118882637	1499	0.075
chr10	118949457	118949496	1500	0.075

chr10	119675620	119675659	1501	0.075
chr10	119750302	119750341	1502	0.075
chr10	120003148	120003187	1503	0.075
chr10	120731349	120731388	1504	0.075
chr10	121495793	121495832	1505	0.075
chr10	121542710	121542749	1506	0.075
chr10	122553474	122553513	1507	0.075
chr10	123158229	123158268	1508	0.075
chr10	123166874	123166913	1509	0.075
chr10	123437562	123437601	1510	0.075
chr10	123887290	123887329	1511	0.075
chr10	124098240	124098279	1512	0.075
chr10	124264199	124264238	1513	0.075
chr10	125141304	125141343	1514	0.075
chr10	125141537	125141576	1515	0.075
chr10	125416343	125416382	1516	0.075
chr10	126154961	126155000	1517	0.075
chr10	126421296	126421335	1518	0.075
chr10	126663506	126663545	1519	0.075
chr10	12681495	12681534	1520	0.075
chr10	127144578	127144617	1521	0.075
chr10	127185659	127185698	1522	0.075
chr10	127362470	127362509	1523	0.075
chr10	127363666	127363705	1524	0.075
chr10	127411990	127412029	1525	0.075
chr10	128192064	128192103	1526	0.075
chr10	128281021	128281060	1527	0.075
chr10	129042205	129042244	1528	0.075
chr10	129044348	129044387	1529	0.075
chr10	129777136	129777175	1530	0.075
chr10	1298854	1298893	1531	0.075
chr10	130547784	130547823	1532	0.075
chr10	130629902	130629941	1533	0.075
chr10	130734165	130734204	1534	0.075
chr10	131537634	131537673	1535	0.075
chr10	131538740	131538779	1536	0.075
chr10	132926304	132926343	1537	0.075
chr10	133969702	133969741	1538	0.075
chr10	134331232	134331271	1539	0.075
chr10	134773792	134773831	1540	0.075
chr10	135065173	135065212	1541	0.075
chr10	13830036	13830075	1542	0.075
chr10	15640800	15640839	1543	0.075
chr10	15807580	15807619	1544	0.075
chr10	1607613	1607652	1545	0.075
chr10	16385393	16385432	1546	0.075
chr10	16566172	16566211	1547	0.075
chr10	17221250	17221289	1548	0.075
chr10	18558300	18558339	1549	0.075
chr10	19465341	19465380	1550	0.075

chr10	21036697	21036736	1551	0.075
chr10	21054178	21054217	1552	0.075
chr10	21664481	21664520	1553	0.075
chr10	24606300	24606339	1554	0.075
chr10	24722106	24722145	1555	0.075
chr10	25277701	25277740	1556	0.075
chr10	25674671	25674710	1557	0.075
chr10	25914977	25915016	1558	0.075
chr10	27018083	27018122	1559	0.075
chr10	27685671	27685710	1560	0.075
chr10	27791837	27791876	1561	0.075
chr10	28260262	28260301	1562	0.075
chr10	28912180	28912219	1563	0.075
chr10	29703622	29703661	1564	0.075
chr10	30356356	30356395	1565	0.075
chr10	31455244	31455283	1566	0.075
chr10	31926925	31926964	1567	0.075
chr10	3207944	3207983	1568	0.075
chr10	33418327	33418366	1569	0.075
chr10	33788796	33788835	1570	0.075
chr10	34648543	34648582	1571	0.075
chr10	35204222	35204261	1572	0.075
chr10	35234175	35234214	1573	0.075
chr10	35661147	35661186	1574	0.075
chr10	35790649	35790688	1575	0.075
chr10	37308163	37308202	1576	0.075
chr10	37556952	37556991	1577	0.075
chr10	43297999	43298038	1578	0.075
chr10	44498212	44498251	1579	0.075
chr10	49460647	49460686	1580	0.075
chr10	49574208	49574247	1581	0.075
chr10	49590482	49590521	1582	0.075
chr10	49854879	49854918	1583	0.075
chr10	50274951	50274990	1584	0.075
chr10	50408224	50408263	1585	0.075
chr10	50560038	50560077	1586	0.075
chr10	53026952	53026991	1587	0.075
chr10	53759052	53759091	1588	0.075
chr10	54909436	54909475	1589	0.075
chr10	57057888	57057927	1590	0.075
chr10	58651109	58651148	1591	0.075
chr10	60826008	60826047	1592	0.075
chr10	6101697	6101736	1593	0.075
chr10	6190842	6190881	1594	0.075
chr10	62593226	62593265	1595	0.075
chr10	63135324	63135363	1596	0.075
chr10	65203139	65203178	1597	0.075
chr10	6798068	6798107	1598	0.075
chr10	68197555	68197594	1599	0.075
chr10	68724239	68724278	1600	0.075

chr10	69825397	69825436	1601	0.075
chr10	71570311	71570350	1602	0.075
chr10	71960551	71960590	1603	0.075
chr10	73078269	73078308	1604	0.075
chr10	73214723	73214762	1605	0.075
chr10	73237494	73237533	1606	0.075
chr10	74927739	74927778	1607	0.075
chr10	74964484	74964523	1608	0.075
chr10	75339444	75339483	1609	0.075
chr10	7571239	7571278	1610	0.075
chr10	76661581	76661620	1611	0.075
chr10	76774791	76774830	1612	0.075
chr10	76829357	76829396	1613	0.075
chr10	7752210	7752249	1614	0.075
chr10	78393524	78393563	1615	0.075
chr10	78520730	78520769	1616	0.075
chr10	78910221	78910260	1617	0.075
chr10	7917623	7917662	1618	0.075
chr10	79251406	79251445	1619	0.075
chr10	80013320	80013359	1620	0.075
chr10	80158441	80158480	1621	0.075
chr10	80191714	80191753	1622	0.075
chr10	80590070	80590109	1623	0.075
chr10	82646342	82646381	1624	0.075
chr10	84101170	84101209	1625	0.075
chr10	84220657	84220696	1626	0.075
chr10	85178364	85178403	1627	0.075
chr10	85750200	85750239	1628	0.075
chr10	85914651	85914690	1629	0.075
chr10	86992663	86992702	1630	0.075
chr10	87732762	87732801	1631	0.075
chr10	87947148	87947187	1632	0.075
chr10	88411781	88411820	1633	0.075
chr10	88508056	88508095	1634	0.075
chr10	88571195	88571234	1635	0.075
chr10	88621856	88621895	1636	0.075
chr10	91241525	91241564	1637	0.075
chr10	92478196	92478235	1638	0.075
chr10	93763710	93763749	1639	0.075
chr10	94395490	94395529	1640	0.075
chr10	95102520	95102559	1641	0.075
chr10	95396853	95396892	1642	0.075
chr10	95542255	95542294	1643	0.075
chr10	96042529	96042568	1644	0.075
chr10	98886749	98886788	1645	0.075
chr10	118519052	118519101	1646	0.08
chr10	114521079	114521125	1647	0.085106383
chr10	102405934	102405973	1648	0.1
chr11	86927281	86927369	1649	0.033707865
chr11	22956675	22956789	1650	0.034782609

chr11	61099051	61099160	1651	0.036363636
chr11	80219920	80219983	1652	0.046875
chr11	100001159	100001196	1653	0.052631579
chr11	100227985	100228022	1654	0.052631579
chr11	100385105	100385142	1655	0.052631579
chr11	100585307	100585344	1656	0.052631579
chr11	100651512	100651549	1657	0.052631579
chr11	100962341	100962378	1658	0.052631579
chr11	101593218	101593255	1659	0.052631579
chr11	101940213	101940250	1660	0.052631579
chr11	101940749	101940786	1661	0.052631579
chr11	102079141	102079178	1662	0.052631579
chr11	102266564	102266601	1663	0.052631579
chr11	10272126	10272163	1664	0.052631579
chr11	103084540	103084577	1665	0.052631579
chr11	103539761	103539798	1666	0.052631579
chr11	104138089	104138126	1667	0.052631579
chr11	104335989	104336026	1668	0.052631579
chr11	104606037	104606074	1669	0.052631579
chr11	104879559	104879596	1670	0.052631579
chr11	106039952	106039989	1671	0.052631579
chr11	106043066	106043103	1672	0.052631579
chr11	106934392	106934429	1673	0.052631579
chr11	106934687	106934724	1674	0.052631579
chr11	107079250	107079287	1675	0.052631579
chr11	107112359	107112396	1676	0.052631579
chr11	107185726	107185763	1677	0.052631579
chr11	107187586	107187623	1678	0.052631579
chr11	107557727	107557764	1679	0.052631579
chr11	10779851	10779888	1680	0.052631579
chr11	108765789	108765826	1681	0.052631579
chr11	108805661	108805698	1682	0.052631579
chr11	108806024	108806061	1683	0.052631579
chr11	109086413	109086450	1684	0.052631579
chr11	109425495	109425532	1685	0.052631579
chr11	109454394	109454431	1686	0.052631579
chr11	109597070	109597107	1687	0.052631579
chr11	110248151	110248188	1688	0.052631579
chr11	110659808	110659845	1689	0.052631579
chr11	111393468	111393505	1690	0.052631579
chr11	111435679	111435716	1691	0.052631579
chr11	111909737	111909774	1692	0.052631579
chr11	112402329	112402366	1693	0.052631579
chr11	112447110	112447147	1694	0.052631579
chr11	112769656	112769693	1695	0.052631579
chr11	112865900	112865937	1696	0.052631579
chr11	11319021	11319058	1697	0.052631579
chr11	113206845	113206882	1698	0.052631579
chr11	113454327	113454364	1699	0.052631579
chr11	113454427	113454464	1700	0.052631579

chr11	113807518	113807555	1701	0.052631579
chr11	114194349	114194386	1702	0.052631579
chr11	114692479	114692516	1703	0.052631579
chr11	114825155	114825192	1704	0.052631579
chr11	114895242	114895279	1705	0.052631579
chr11	114924854	114924891	1706	0.052631579
chr11	115097485	115097522	1707	0.052631579
chr11	115585948	115585985	1708	0.052631579
chr11	115619832	115619869	1709	0.052631579
chr11	115649411	115649448	1710	0.052631579
chr11	11623768	11623805	1711	0.052631579
chr11	116539726	116539763	1712	0.052631579
chr11	11677599	11677636	1713	0.052631579
chr11	116903372	116903409	1714	0.052631579
chr11	116991560	116991597	1715	0.052631579
chr11	117213637	117213674	1716	0.052631579
chr11	117214436	117214473	1717	0.052631579
chr11	117244987	117245024	1718	0.052631579
chr11	117409175	117409212	1719	0.052631579
chr11	117901943	117901980	1720	0.052631579
chr11	118287162	118287199	1721	0.052631579
chr11	118288001	118288038	1722	0.052631579
chr11	118501751	118501788	1723	0.052631579
chr11	118924078	118924115	1724	0.052631579
chr11	119230432	119230469	1725	0.052631579
chr11	119602765	119602802	1726	0.052631579
chr11	119944788	119944825	1727	0.052631579
chr11	120061772	120061809	1728	0.052631579
chr11	12025118	12025155	1729	0.052631579
chr11	12034302	12034339	1730	0.052631579
chr11	120890936	120890973	1731	0.052631579
chr11	121065216	121065253	1732	0.052631579
chr11	1213167	1213204	1733	0.052631579
chr11	121527680	121527717	1734	0.052631579
chr11	121528440	121528477	1735	0.052631579
chr11	121532107	121532144	1736	0.052631579
chr11	12201024	12201061	1737	0.052631579
chr11	12205274	12205311	1738	0.052631579
chr11	123579342	123579379	1739	0.052631579
chr11	123960860	123960897	1740	0.052631579
chr11	124220853	124220890	1741	0.052631579
chr11	124392260	124392297	1742	0.052631579
chr11	124443395	124443432	1743	0.052631579
chr11	124543114	124543151	1744	0.052631579
chr11	124774506	124774543	1745	0.052631579
chr11	124785724	124785761	1746	0.052631579
chr11	124831183	124831220	1747	0.052631579
chr11	124972281	124972318	1748	0.052631579
chr11	12506704	12506741	1749	0.052631579
chr11	12516192	12516229	1750	0.052631579

chr11	125638748	125638785	1751	0.052631579
chr11	125791268	125791305	1752	0.052631579
chr11	126033302	126033339	1753	0.052631579
chr11	126085600	126085637	1754	0.052631579
chr11	126412825	126412862	1755	0.052631579
chr11	126573597	126573634	1756	0.052631579
chr11	126898201	126898238	1757	0.052631579
chr11	128347488	128347525	1758	0.052631579
chr11	128507189	128507226	1759	0.052631579
chr11	128946505	128946542	1760	0.052631579
chr11	129295575	129295612	1761	0.052631579
chr11	129408163	129408200	1762	0.052631579
chr11	129810793	129810830	1763	0.052631579
chr11	130369150	130369187	1764	0.052631579
chr11	131134978	131135015	1765	0.052631579
chr11	131135701	131135738	1766	0.052631579
chr11	131282965	131283002	1767	0.052631579
chr11	131283069	131283106	1768	0.052631579
chr11	131689836	131689873	1769	0.052631579
chr11	132369262	132369299	1770	0.052631579
chr11	132573900	132573937	1771	0.052631579
chr11	132630202	132630239	1772	0.052631579
chr11	132982576	132982613	1773	0.052631579
chr11	133601131	133601168	1774	0.052631579
chr11	1379658	1379695	1775	0.052631579
chr11	13903905	13903942	1776	0.052631579
chr11	13929479	13929516	1777	0.052631579
chr11	14875863	14875900	1778	0.052631579
chr11	15518570	15518607	1779	0.052631579
chr11	15918497	15918534	1780	0.052631579
chr11	16193653	16193690	1781	0.052631579
chr11	16286986	16287023	1782	0.052631579
chr11	16350438	16350475	1783	0.052631579
chr11	16883646	16883683	1784	0.052631579
chr11	17373328	17373365	1785	0.052631579
chr11	17698490	17698527	1786	0.052631579
chr11	17950877	17950914	1787	0.052631579
chr11	18507284	18507321	1788	0.052631579
chr11	18680254	18680291	1789	0.052631579
chr11	18706442	18706479	1790	0.052631579
chr11	18845612	18845649	1791	0.052631579
chr11	19516802	19516839	1792	0.052631579
chr11	19799555	19799592	1793	0.052631579
chr11	1982441	1982478	1794	0.052631579
chr11	199484	199521	1795	0.052631579
chr11	21164920	21164957	1796	0.052631579
chr11	21853051	21853088	1797	0.052631579
chr11	22680594	22680631	1798	0.052631579
chr11	23074084	23074121	1799	0.052631579
chr11	23147920	23147957	1800	0.052631579

chr11	23808418	23808455	1801	0.052631579
chr11	2400718	2400755	1802	0.052631579
chr11	2418199	2418236	1803	0.052631579
chr11	2485798	2485835	1804	0.052631579
chr11	2537212	2537249	1805	0.052631579
chr11	2553035	2553072	1806	0.052631579
chr11	25818478	25818515	1807	0.052631579
chr11	26370830	26370867	1808	0.052631579
chr11	268134	268171	1809	0.052631579
chr11	27136309	27136346	1810	0.052631579
chr11	27274269	27274306	1811	0.052631579
chr11	28058010	28058047	1812	0.052631579
chr11	28718013	28718050	1813	0.052631579
chr11	29254309	29254346	1814	0.052631579
chr11	2941920	2941957	1815	0.052631579
chr11	29802901	29802938	1816	0.052631579
chr11	29803883	29803920	1817	0.052631579
chr11	2989631	2989668	1818	0.052631579
chr11	2990228	2990265	1819	0.052631579
chr11	30136615	30136652	1820	0.052631579
chr11	30137474	30137511	1821	0.052631579
chr11	305097	305134	1822	0.052631579
chr11	30521713	30521750	1823	0.052631579
chr11	31001110	31001147	1824	0.052631579
chr11	31393102	31393139	1825	0.052631579
chr11	31434415	31434452	1826	0.052631579
chr11	32449355	32449392	1827	0.052631579
chr11	33088596	33088633	1828	0.052631579
chr11	34894618	34894655	1829	0.052631579
chr11	35352887	35352924	1830	0.052631579
chr11	35387027	35387064	1831	0.052631579
chr11	36878516	36878553	1832	0.052631579
chr11	36955826	36955863	1833	0.052631579
chr11	36955940	36955977	1834	0.052631579
chr11	37318788	37318825	1835	0.052631579
chr11	37865677	37865714	1836	0.052631579
chr11	39082261	39082298	1837	0.052631579
chr11	39638863	39638900	1838	0.052631579
chr11	39657185	39657222	1839	0.052631579
chr11	40092992	40093029	1840	0.052631579
chr11	40267614	40267651	1841	0.052631579
chr11	40605827	40605864	1842	0.052631579
chr11	40668921	40668958	1843	0.052631579
chr11	40844397	40844434	1844	0.052631579
chr11	41011774	41011811	1845	0.052631579
chr11	41168926	41168963	1846	0.052631579
chr11	41928535	41928572	1847	0.052631579
chr11	41967772	41967809	1848	0.052631579
chr11	42238008	42238045	1849	0.052631579
chr11	42526705	42526742	1850	0.052631579

chr11	43023546	43023583	1851	0.052631579
chr11	43222281	43222318	1852	0.052631579
chr11	43568496	43568533	1853	0.052631579
chr11	43787622	43787659	1854	0.052631579
chr11	44206159	44206196	1855	0.052631579
chr11	44451349	44451386	1856	0.052631579
chr11	44452149	44452186	1857	0.052631579
chr11	45020473	45020510	1858	0.052631579
chr11	45088217	45088254	1859	0.052631579
chr11	45343502	45343539	1860	0.052631579
chr11	45344327	45344364	1861	0.052631579
chr11	45430810	45430847	1862	0.052631579
chr11	45932435	45932472	1863	0.052631579
chr11	4615002	4615039	1864	0.052631579
chr11	46366325	46366362	1865	0.052631579
chr11	46486356	46486393	1866	0.052631579
chr11	46569227	46569264	1867	0.052631579
chr11	46683956	46683993	1868	0.052631579
chr11	46737491	46737528	1869	0.052631579
chr11	46924201	46924238	1870	0.052631579
chr11	47289892	47289929	1871	0.052631579
chr11	47398401	47398438	1872	0.052631579
chr11	47510549	47510586	1873	0.052631579
chr11	47523669	47523706	1874	0.052631579
chr11	47620485	47620522	1875	0.052631579
chr11	47763113	47763150	1876	0.052631579
chr11	47826390	47826427	1877	0.052631579
chr11	47877925	47877962	1878	0.052631579
chr11	479288	479325	1879	0.052631579
chr11	479980	480017	1880	0.052631579
chr11	48047931	48047968	1881	0.052631579
chr11	48215850	48215887	1882	0.052631579
chr11	48344886	48344923	1883	0.052631579
chr11	4953306	4953343	1884	0.052631579
chr11	4961739	4961776	1885	0.052631579
chr11	526018	526055	1886	0.052631579
chr11	5411346	5411383	1887	0.052631579
chr11	5428963	5429000	1888	0.052631579
chr11	55250828	55250865	1889	0.052631579
chr11	55670005	55670042	1890	0.052631579
chr11	55670885	55670922	1891	0.052631579
chr11	56157911	56157948	1892	0.052631579
chr11	5644623	5644660	1893	0.052631579
chr11	56719220	56719257	1894	0.052631579
chr11	56729635	56729672	1895	0.052631579
chr11	56943274	56943311	1896	0.052631579
chr11	56987866	56987903	1897	0.052631579
chr11	57000783	57000820	1898	0.052631579
chr11	57070569	57070606	1899	0.052631579
chr11	58369853	58369890	1900	0.052631579

chr11	58611525	58611562	1901	0.052631579
chr11	58652499	58652536	1902	0.052631579
chr11	58653338	58653375	1903	0.052631579
chr11	58942935	58942972	1904	0.052631579
chr11	59300806	59300843	1905	0.052631579
chr11	60648098	60648173	1906	0.052631579
chr11	60826626	60826663	1907	0.052631579
chr11	61625770	61625807	1908	0.052631579
chr11	61691391	61691428	1909	0.052631579
chr11	61824887	61824924	1910	0.052631579
chr11	62044309	62044346	1911	0.052631579
chr11	62200612	62200649	1912	0.052631579
chr11	62277668	62277705	1913	0.052631579
chr11	62355676	62355713	1914	0.052631579
chr11	62404304	62404341	1915	0.052631579
chr11	62897326	62897363	1916	0.052631579
chr11	63416731	63416768	1917	0.052631579
chr11	63560719	63560756	1918	0.052631579
chr11	63631615	63631652	1919	0.052631579
chr11	63839088	63839125	1920	0.052631579
chr11	64064268	64064305	1921	0.052631579
chr11	64071517	64071554	1922	0.052631579
chr11	64145857	64145894	1923	0.052631579
chr11	64154516	64154553	1924	0.052631579
chr11	64384038	64384075	1925	0.052631579
chr11	64402266	64402303	1926	0.052631579
chr11	64519757	64519794	1927	0.052631579
chr11	64538253	64538290	1928	0.052631579
chr11	64800742	64800779	1929	0.052631579
chr11	6484092	6484129	1930	0.052631579
chr11	64905101	64905138	1931	0.052631579
chr11	65027137	65027174	1932	0.052631579
chr11	65064599	65064636	1933	0.052631579
chr11	65139829	65139866	1934	0.052631579
chr11	65397580	65397617	1935	0.052631579
chr11	65415159	65415196	1936	0.052631579
chr11	65501644	65501681	1937	0.052631579
chr11	65634074	65634111	1938	0.052631579
chr11	65733131	65733168	1939	0.052631579
chr11	65744805	65744842	1940	0.052631579
chr11	66043673	66043710	1941	0.052631579
chr11	6604833	6604870	1942	0.052631579
chr11	66067932	66067969	1943	0.052631579
chr11	66080197	66080234	1944	0.052631579
chr11	66086166	66086203	1945	0.052631579
chr11	6624579	6624616	1946	0.052631579
chr11	66367635	66367672	1947	0.052631579
chr11	66380697	66380734	1948	0.052631579
chr11	67889241	67889278	1949	0.052631579
chr11	68427371	68427408	1950	0.052631579

chr11	68434585	68434622	1951	0.052631579
chr11	68436625	68436662	1952	0.052631579
chr11	68484237	68484274	1953	0.052631579
chr11	68484998	68485035	1954	0.052631579
chr11	68595346	68595383	1955	0.052631579
chr11	68724201	68724238	1956	0.052631579
chr11	68809913	68809950	1957	0.052631579
chr11	69175636	69175673	1958	0.052631579
chr11	69229348	69229385	1959	0.052631579
chr11	6936009	6936046	1960	0.052631579
chr11	69464097	69464134	1961	0.052631579
chr11	69529175	69529212	1962	0.052631579
chr11	69924397	69924434	1963	0.052631579
chr11	69938346	69938383	1964	0.052631579
chr11	70403072	70403109	1965	0.052631579
chr11	70462316	70462353	1966	0.052631579
chr11	70875985	70876022	1967	0.052631579
chr11	710127	710164	1968	0.052631579
chr11	71110110	71110147	1969	0.052631579
chr11	71401887	71401924	1970	0.052631579
chr11	71639093	71639130	1971	0.052631579
chr11	71681969	71682006	1972	0.052631579
chr11	7198010	7198047	1973	0.052631579
chr11	72101230	72101267	1974	0.052631579
chr11	72284577	72284614	1975	0.052631579
chr11	72618963	72619000	1976	0.052631579
chr11	72676644	72676681	1977	0.052631579
chr11	72690827	72690864	1978	0.052631579
chr11	72722821	72722858	1979	0.052631579
chr11	72984711	72984748	1980	0.052631579
chr11	73992420	73992457	1981	0.052631579
chr11	7403676	7403713	1982	0.052631579
chr11	74182020	74182057	1983	0.052631579
chr11	74565313	74565350	1984	0.052631579
chr11	74649526	74649563	1985	0.052631579
chr11	74649753	74649790	1986	0.052631579
chr11	74774669	74774706	1987	0.052631579
chr11	74954373	74954410	1988	0.052631579
chr11	74955365	74955402	1989	0.052631579
chr11	75457784	75457821	1990	0.052631579
chr11	75691680	75691717	1991	0.052631579
chr11	76252094	76252131	1992	0.052631579
chr11	76593379	76593416	1993	0.052631579
chr11	76596801	76596838	1994	0.052631579
chr11	77102573	77102610	1995	0.052631579
chr11	77608010	77608047	1996	0.052631579
chr11	77681891	77681928	1997	0.052631579
chr11	77817355	77817392	1998	0.052631579
chr11	78063087	78063124	1999	0.052631579
chr11	78280025	78280062	2000	0.052631579

chr11	78656594	78656631	2001	0.052631579
chr11	79433591	79433628	2002	0.052631579
chr11	79927199	79927236	2003	0.052631579
chr11	7997429	7997466	2004	0.052631579
chr11	80045826	80045863	2005	0.052631579
chr11	80409790	80409827	2006	0.052631579
chr11	80605830	80605867	2007	0.052631579
chr11	80883298	80883335	2008	0.052631579
chr11	81022270	81022307	2009	0.052631579
chr11	8114358	8114395	2010	0.052631579
chr11	81405531	81405568	2011	0.052631579
chr11	81448976	81449013	2012	0.052631579
chr11	814705	814742	2013	0.052631579
chr11	82122119	82122156	2014	0.052631579
chr11	8215370	8215407	2015	0.052631579
chr11	82538112	82538149	2016	0.052631579
chr11	82597082	82597119	2017	0.052631579
chr11	82597694	82597731	2018	0.052631579
chr11	8308153	8308190	2019	0.052631579
chr11	83161625	83161662	2020	0.052631579
chr11	83924229	83924266	2021	0.052631579
chr11	84133388	84133425	2022	0.052631579
chr11	84829913	84829950	2023	0.052631579
chr11	85107092	85107129	2024	0.052631579
chr11	85307853	85307890	2025	0.052631579
chr11	854475	854512	2026	0.052631579
chr11	85673127	85673164	2027	0.052631579
chr11	85830871	85830908	2028	0.052631579
chr11	85860317	85860354	2029	0.052631579
chr11	8615983	8616020	2030	0.052631579
chr11	8687440	8687477	2031	0.052631579
chr11	87204227	87204264	2032	0.052631579
chr11	88070128	88070165	2033	0.052631579
chr11	88632436	88632473	2034	0.052631579
chr11	897840	897877	2035	0.052631579
chr11	90465316	90465353	2036	0.052631579
chr11	90969415	90969452	2037	0.052631579
chr11	91125564	91125601	2038	0.052631579
chr11	91637108	91637145	2039	0.052631579
chr11	91677022	91677059	2040	0.052631579
chr11	91808699	91808736	2041	0.052631579
chr11	91809440	91809477	2042	0.052631579
chr11	91874390	91874427	2043	0.052631579
chr11	92314746	92314783	2044	0.052631579
chr11	92458406	92458443	2045	0.052631579
chr11	9274978	9275015	2046	0.052631579
chr11	93014270	93014307	2047	0.052631579
chr11	93327471	93327508	2048	0.052631579
chr11	93328276	93328313	2049	0.052631579
chr11	9425462	9425499	2050	0.052631579

chr11	94483009	94483046	2051	0.052631579
chr11	94483463	94483500	2052	0.052631579
chr11	94523323	94523360	2053	0.052631579
chr11	95178632	95178669	2054	0.052631579
chr11	95207827	95207864	2055	0.052631579
chr11	95816606	95816643	2056	0.052631579
chr11	97065818	97065855	2057	0.052631579
chr11	97181039	97181076	2058	0.052631579
chr11	9718444	9718481	2059	0.052631579
chr11	97416820	97416857	2060	0.052631579
chr11	98224062	98224099	2061	0.052631579
chr11	98818025	98818062	2062	0.052631579
chr11	98818762	98818799	2063	0.052631579
chr11	99117925	99117962	2064	0.052631579
chr11	99347516	99347553	2065	0.052631579
chr11	99405835	99405872	2066	0.052631579
chr11	99487520	99487557	2067	0.052631579
chr11	99743244	99743281	2068	0.052631579
chr11	63434952	63435025	2069	0.054054054
chr11	100779342	100779381	2070	0.075
chr11	10140980	10141019	2071	0.075
chr11	102780347	102780386	2072	0.075
chr11	105388370	105388409	2073	0.075
chr11	106325292	106325331	2074	0.075
chr11	10637793	10637832	2075	0.075
chr11	10641812	10641851	2076	0.075
chr11	108389083	108389122	2077	0.075
chr11	109892781	109892820	2078	0.075
chr11	110303444	110303483	2079	0.075
chr11	110525678	110525717	2080	0.075
chr11	111111332	111111371	2081	0.075
chr11	113283312	113283351	2082	0.075
chr11	113309368	113309407	2083	0.075
chr11	113558446	113558485	2084	0.075
chr11	11406073	11406112	2085	0.075
chr11	114236839	114236878	2086	0.075
chr11	115956927	115956966	2087	0.075
chr11	116201555	116201594	2088	0.075
chr11	116210078	116210117	2089	0.075
chr11	116864026	116864065	2090	0.075
chr11	117250293	117250332	2091	0.075
chr11	117469415	117469454	2092	0.075
chr11	117794174	117794213	2093	0.075
chr11	117982963	117983002	2094	0.075
chr11	118694713	118694752	2095	0.075
chr11	119215526	119215565	2096	0.075
chr11	119843672	119843711	2097	0.075
chr11	120277882	120277921	2098	0.075
chr11	120350690	120350729	2099	0.075
chr11	121098817	121098856	2100	0.075

chr11	122394474	122394513	2101	0.075
chr11	122434261	122434300	2102	0.075
chr11	122460403	122460442	2103	0.075
chr11	123035583	123035622	2104	0.075
chr11	123129615	123129654	2105	0.075
chr11	124207073	124207112	2106	0.075
chr11	124660001	124660040	2107	0.075
chr11	125270756	125270795	2108	0.075
chr11	125425999	125426038	2109	0.075
chr11	125538270	125538309	2110	0.075
chr11	125589860	125589899	2111	0.075
chr11	125815631	125815670	2112	0.075
chr11	126141305	126141344	2113	0.075
chr11	126301653	126301692	2114	0.075
chr11	12669814	12669853	2115	0.075
chr11	126981308	126981347	2116	0.075
chr11	127050558	127050597	2117	0.075
chr11	127368936	127368975	2118	0.075
chr11	12825278	12825317	2119	0.075
chr11	128865336	128865375	2120	0.075
chr11	129380016	129380055	2121	0.075
chr11	13002809	13002848	2122	0.075
chr11	130366226	130366265	2123	0.075
chr11	130910768	130910807	2124	0.075
chr11	131639864	131639903	2125	0.075
chr11	13191878	13191917	2126	0.075
chr11	132382686	132382725	2127	0.075
chr11	132427024	132427063	2128	0.075
chr11	132570566	132570605	2129	0.075
chr11	133424473	133424512	2130	0.075
chr11	133786164	133786203	2131	0.075
chr11	134022166	134022205	2132	0.075
chr11	134120472	134120511	2133	0.075
chr11	13466994	13467033	2134	0.075
chr11	13816757	13816796	2135	0.075
chr11	14953670	14953709	2136	0.075
chr11	15467951	15467990	2137	0.075
chr11	15698380	15698419	2138	0.075
chr11	17364730	17364769	2139	0.075
chr11	18462296	18462335	2140	0.075
chr11	19386288	19386327	2141	0.075
chr11	19975959	19975998	2142	0.075
chr11	20117391	20117430	2143	0.075
chr11	20859220	20859259	2144	0.075
chr11	2111358	2111397	2145	0.075
chr11	21173482	21173521	2146	0.075
chr11	23286062	23286101	2147	0.075
chr11	27450372	27450411	2148	0.075
chr11	27679737	27679776	2149	0.075
chr11	2777492	2777531	2150	0.075

chr11	27867173	27867212	2151	0.075
chr11	29591616	29591655	2152	0.075
chr11	3079521	3079560	2153	0.075
chr11	31235157	31235196	2154	0.075
chr11	31747131	31747170	2155	0.075
chr11	31789179	31789218	2156	0.075
chr11	31858057	31858096	2157	0.075
chr11	32456957	32456996	2158	0.075
chr11	324873	324912	2159	0.075
chr11	32506154	32506193	2160	0.075
chr11	33358101	33358140	2161	0.075
chr11	34253418	34253457	2162	0.075
chr11	34309306	34309345	2163	0.075
chr11	35327359	35327398	2164	0.075
chr11	38677846	38677885	2165	0.075
chr11	40837150	40837189	2166	0.075
chr11	42948328	42948367	2167	0.075
chr11	42954649	42954688	2168	0.075
chr11	44316851	44316890	2169	0.075
chr11	45204932	45204971	2170	0.075
chr11	45546640	45546679	2171	0.075
chr11	45590681	45590720	2172	0.075
chr11	45845777	45845816	2173	0.075
chr11	46899259	46899298	2174	0.075
chr11	46912467	46912506	2175	0.075
chr11	46937427	46937466	2176	0.075
chr11	4699110	4699149	2177	0.075
chr11	47568363	47568402	2178	0.075
chr11	51319122	51319161	2179	0.075
chr11	55158476	55158515	2180	0.075
chr11	56020298	56020337	2181	0.075
chr11	57306072	57306111	2182	0.075
chr11	60366373	60366412	2183	0.075
chr11	60422642	60422681	2184	0.075
chr11	60424653	60424692	2185	0.075
chr11	60430781	60430820	2186	0.075
chr11	60891304	60891343	2187	0.075
chr11	60940306	60940345	2188	0.075
chr11	60971985	60972024	2189	0.075
chr11	61098910	61098949	2190	0.075
chr11	62057908	62057947	2191	0.075
chr11	62118406	62118445	2192	0.075
chr11	62528136	62528175	2193	0.075
chr11	6300579	6300618	2194	0.075
chr11	63263215	63263254	2195	0.075
chr11	6342064	6342103	2196	0.075
chr11	63431570	63431609	2197	0.075
chr11	64291625	64291664	2198	0.075
chr11	65159719	65159758	2199	0.075
chr11	6543754	6543793	2200	0.075

chr11	65942581	65942620	2201	0.075
chr11	66217940	66217979	2202	0.075
chr11	66436693	66436732	2203	0.075
chr11	66876821	66876860	2204	0.075
chr11	66889058	66889097	2205	0.075
chr11	66945194	66945233	2206	0.075
chr11	6694653	6694692	2207	0.075
chr11	66962448	66962487	2208	0.075
chr11	67561021	67561060	2209	0.075
chr11	6769139	6769178	2210	0.075
chr11	68977031	68977070	2211	0.075
chr11	69165183	69165222	2212	0.075
chr11	69867537	69867576	2213	0.075
chr11	72084499	72084538	2214	0.075
chr11	72610165	72610204	2215	0.075
chr11	72789509	72789548	2216	0.075
chr11	75114776	75114815	2217	0.075
chr11	75472381	75472420	2218	0.075
chr11	76405447	76405486	2219	0.075
chr11	77208835	77208874	2220	0.075
chr11	77743354	77743393	2221	0.075
chr11	78185632	78185671	2222	0.075
chr11	7836356	7836395	2223	0.075
chr11	78815659	78815698	2224	0.075
chr11	79084671	79084710	2225	0.075
chr11	79122916	79122955	2226	0.075
chr11	79326482	79326521	2227	0.075
chr11	79329438	79329477	2228	0.075
chr11	7962327	7962366	2229	0.075
chr11	8184693	8184732	2230	0.075
chr11	82917527	82917566	2231	0.075
chr11	8294296	8294335	2232	0.075
chr11	85323754	85323793	2233	0.075
chr11	87942777	87942816	2234	0.075
chr11	8995004	8995043	2235	0.075
chr11	92217343	92217382	2236	0.075
chr11	93029046	93029085	2237	0.075
chr11	93398669	93398708	2238	0.075
chr11	93598361	93598400	2239	0.075
chr11	93687328	93687367	2240	0.075
chr11	93770259	93770298	2241	0.075
chr11	93813484	93813523	2242	0.075
chr11	94131437	94131476	2243	0.075
chr11	94307086	94307125	2244	0.075
chr11	94967873	94967912	2245	0.075
chr11	97992030	97992069	2246	0.075
chr11	99846559	99846598	2247	0.075
chr11	21067839	21067891	2248	0.075471698
chr11	10785390	10785438	2249	0.081632653
chr11	57488585	57488632	2250	0.083333333

chr12	98072716	98072828	2251	0.03539823
chr12	59171672	59171783	2252	0.035714286
chr12	131672257	131672365	2253	0.036697248
chr12	88796930	88797035	2254	0.037735849
chr12	5024972	5025049	2255	0.038461538
chr12	53655972	53656071	2256	0.04
chr12	52147800	52147885	2257	0.046511628
chr12	46215253	46215332	2258	0.05
chr12	89023321	89023437	2259	0.051282051
chr12	63654960	63655036	2260	0.051948052
chr12	100328719	100328756	2261	0.052631579
chr12	100532134	100532171	2262	0.052631579
chr12	100665638	100665675	2263	0.052631579
chr12	100681806	100681843	2264	0.052631579
chr12	100804010	100804047	2265	0.052631579
chr12	101003989	101004026	2266	0.052631579
chr12	101004593	101004630	2267	0.052631579
chr12	102262431	102262468	2268	0.052631579
chr12	102651499	102651536	2269	0.052631579
chr12	102861701	102861738	2270	0.052631579
chr12	102883176	102883213	2271	0.052631579
chr12	10355368	10355405	2272	0.052631579
chr12	103976071	103976108	2273	0.052631579
chr12	104071373	104071410	2274	0.052631579
chr12	104194325	104194362	2275	0.052631579
chr12	104858245	104858282	2276	0.052631579
chr12	104873336	104873373	2277	0.052631579
chr12	105012083	105012120	2278	0.052631579
chr12	105155677	105155714	2279	0.052631579
chr12	105252519	105252556	2280	0.052631579
chr12	105507048	105507085	2281	0.052631579
chr12	105507803	105507840	2282	0.052631579
chr12	105875839	105875876	2283	0.052631579
chr12	106189902	106189939	2284	0.052631579
chr12	107386745	107386782	2285	0.052631579
chr12	10745324	10745361	2286	0.052631579
chr12	107454144	107454181	2287	0.052631579
chr12	107576653	107576690	2288	0.052631579
chr12	107629989	107630026	2289	0.052631579
chr12	108012340	108012377	2290	0.052631579
chr12	108203886	108203923	2291	0.052631579
chr12	108204678	108204715	2292	0.052631579
chr12	108989602	108989639	2293	0.052631579
chr12	109068322	109068359	2294	0.052631579
chr12	10931337	10931374	2295	0.052631579
chr12	110374991	110375028	2296	0.052631579
chr12	110408022	110408059	2297	0.052631579
chr12	110706617	110706654	2298	0.052631579
chr12	110707155	110707192	2299	0.052631579
chr12	111671731	111671768	2300	0.052631579

chr12	11170960	11170997	2301	0.052631579
chr12	11171068	11171105	2302	0.052631579
chr12	111724000	111724037	2303	0.052631579
chr12	111750520	111750557	2304	0.052631579
chr12	111811352	111811389	2305	0.052631579
chr12	111985001	111985038	2306	0.052631579
chr12	112294254	112294291	2307	0.052631579
chr12	112406358	112406395	2308	0.052631579
chr12	113585768	113585805	2309	0.052631579
chr12	113944132	113944169	2310	0.052631579
chr12	113974484	113974521	2311	0.052631579
chr12	11400782	11400819	2312	0.052631579
chr12	114114552	114114589	2313	0.052631579
chr12	114483437	114483474	2314	0.052631579
chr12	115693106	115693143	2315	0.052631579
chr12	115892986	115893023	2316	0.052631579
chr12	116601218	116601255	2317	0.052631579
chr12	117171251	117171288	2318	0.052631579
chr12	117903643	117903680	2319	0.052631579
chr12	11801337	11801374	2320	0.052631579
chr12	118161906	118161943	2321	0.052631579
chr12	118261099	118261136	2322	0.052631579
chr12	118264047	118264084	2323	0.052631579
chr12	118527990	118528027	2324	0.052631579
chr12	118585421	118585458	2325	0.052631579
chr12	11870392	11870429	2326	0.052631579
chr12	118762679	118762716	2327	0.052631579
chr12	119018302	119018339	2328	0.052631579
chr12	119094439	119094476	2329	0.052631579
chr12	119095093	119095130	2330	0.052631579
chr12	119184237	119184274	2331	0.052631579
chr12	119184981	119185018	2332	0.052631579
chr12	1192510	1192547	2333	0.052631579
chr12	11944808	11944845	2334	0.052631579
chr12	119609057	119609094	2335	0.052631579
chr12	119748135	119748172	2336	0.052631579
chr12	119918951	119918988	2337	0.052631579
chr12	120055216	120055253	2338	0.052631579
chr12	120250709	120250746	2339	0.052631579
chr12	120927493	120927530	2340	0.052631579
chr12	121191326	121191363	2341	0.052631579
chr12	121192121	121192158	2342	0.052631579
chr12	121314094	121314131	2343	0.052631579
chr12	121324579	121324616	2344	0.052631579
chr12	121633487	121633524	2345	0.052631579
chr12	122354156	122354193	2346	0.052631579
chr12	122396826	122396863	2347	0.052631579
chr12	122408925	122408962	2348	0.052631579
chr12	122434655	122434692	2349	0.052631579
chr12	122478456	122478493	2350	0.052631579

chr12	122672396	122672433	2351	0.052631579
chr12	122967015	122967052	2352	0.052631579
chr12	123172106	123172143	2353	0.052631579
chr12	123177775	123177812	2354	0.052631579
chr12	123314406	123314443	2355	0.052631579
chr12	123452184	123452221	2356	0.052631579
chr12	123538781	123538818	2357	0.052631579
chr12	124143074	124143111	2358	0.052631579
chr12	124143437	124143474	2359	0.052631579
chr12	124200814	124200851	2360	0.052631579
chr12	124533758	124533795	2361	0.052631579
chr12	124701857	124701894	2362	0.052631579
chr12	125600552	125600589	2363	0.052631579
chr12	125740204	125740241	2364	0.052631579
chr12	126240063	126240100	2365	0.052631579
chr12	126355765	126355802	2366	0.052631579
chr12	126378750	126378787	2367	0.052631579
chr12	126404149	126404186	2368	0.052631579
chr12	126947984	126948021	2369	0.052631579
chr12	127062097	127062134	2370	0.052631579
chr12	127208389	127208426	2371	0.052631579
chr12	127907815	127907852	2372	0.052631579
chr12	128661325	128661362	2373	0.052631579
chr12	128747070	128747107	2374	0.052631579
chr12	12899514	12899551	2375	0.052631579
chr12	129355231	129355268	2376	0.052631579
chr12	129382286	129382323	2377	0.052631579
chr12	130097784	130097821	2378	0.052631579
chr12	130159281	130159318	2379	0.052631579
chr12	130535740	130535777	2380	0.052631579
chr12	131015047	131015084	2381	0.052631579
chr12	131433176	131433213	2382	0.052631579
chr12	131443453	131443490	2383	0.052631579
chr12	131515706	131515743	2384	0.052631579
chr12	13256835	13256872	2385	0.052631579
chr12	14099161	14099198	2386	0.052631579
chr12	1434422	1434459	2387	0.052631579
chr12	1435157	1435194	2388	0.052631579
chr12	14605261	14605298	2389	0.052631579
chr12	14867617	14867654	2390	0.052631579
chr12	15053285	15053322	2391	0.052631579
chr12	1531396	1531433	2392	0.052631579
chr12	15449927	15449964	2393	0.052631579
chr12	15672133	15672170	2394	0.052631579
chr12	15740915	15740952	2395	0.052631579
chr12	15950793	15950830	2396	0.052631579
chr12	16363835	16363872	2397	0.052631579
chr12	16363979	16364016	2398	0.052631579
chr12	16634719	16634756	2399	0.052631579
chr12	16664796	16664833	2400	0.052631579

chr12	16676655	16676692	2401	0.052631579
chr12	1845077	1845114	2402	0.052631579
chr12	19174292	19174329	2403	0.052631579
chr12	19801610	19801647	2404	0.052631579
chr12	19902487	19902524	2405	0.052631579
chr12	19998877	19998914	2406	0.052631579
chr12	20043834	20043871	2407	0.052631579
chr12	2012989	2013026	2408	0.052631579
chr12	20184954	20184991	2409	0.052631579
chr12	202613	202650	2410	0.052631579
chr12	20442234	20442271	2411	0.052631579
chr12	20510635	20510672	2412	0.052631579
chr12	21574207	21574244	2413	0.052631579
chr12	21679691	21679728	2414	0.052631579
chr12	22365924	22365961	2415	0.052631579
chr12	22535748	22535785	2416	0.052631579
chr12	22710149	22710186	2417	0.052631579
chr12	24459617	24459654	2418	0.052631579
chr12	24494298	24494335	2419	0.052631579
chr12	2562330	2562367	2420	0.052631579
chr12	26241563	26241600	2421	0.052631579
chr12	26565938	26565975	2422	0.052631579
chr12	26803069	26803106	2423	0.052631579
chr12	2715440	2715477	2424	0.052631579
chr12	27304499	27304536	2425	0.052631579
chr12	27359297	27359334	2426	0.052631579
chr12	27477880	27477917	2427	0.052631579
chr12	27696651	27696688	2428	0.052631579
chr12	28077668	28077705	2429	0.052631579
chr12	28097621	28097658	2430	0.052631579
chr12	2836513	2836550	2431	0.052631579
chr12	28738078	28738115	2432	0.052631579
chr12	28765430	28765467	2433	0.052631579
chr12	28795389	28795426	2434	0.052631579
chr12	28827200	28827237	2435	0.052631579
chr12	29022753	29022790	2436	0.052631579
chr12	29210794	29210831	2437	0.052631579
chr12	29286480	29286517	2438	0.052631579
chr12	29499480	29499517	2439	0.052631579
chr12	2950747	2950784	2440	0.052631579
chr12	29809040	29809077	2441	0.052631579
chr12	29872696	29872733	2442	0.052631579
chr12	30004568	30004605	2443	0.052631579
chr12	30088285	30088322	2444	0.052631579
chr12	30088615	30088652	2445	0.052631579
chr12	30106815	30106852	2446	0.052631579
chr12	30233894	30233931	2447	0.052631579
chr12	30251079	30251116	2448	0.052631579
chr12	30375279	30375316	2449	0.052631579
chr12	30376156	30376193	2450	0.052631579

chr12	3057351	3057388	2451	0.052631579
chr12	30714774	30714811	2452	0.052631579
chr12	30898085	30898122	2453	0.052631579
chr12	31140191	31140228	2454	0.052631579
chr12	31817898	31817935	2455	0.052631579
chr12	31993752	31993789	2456	0.052631579
chr12	3211171	3211208	2457	0.052631579
chr12	32329181	32329218	2458	0.052631579
chr12	32464454	32464491	2459	0.052631579
chr12	33756650	33756687	2460	0.052631579
chr12	34196296	34196333	2461	0.052631579
chr12	34217453	34217490	2462	0.052631579
chr12	37308683	37308720	2463	0.052631579
chr12	37568820	37568857	2464	0.052631579
chr12	37957755	37957792	2465	0.052631579
chr12	37987284	37987321	2466	0.052631579
chr12	38030272	38030309	2467	0.052631579
chr12	38967545	38967582	2468	0.052631579
chr12	39657679	39657716	2469	0.052631579
chr12	4064174	4064211	2470	0.052631579
chr12	40692269	40692306	2471	0.052631579
chr12	4258545	4258582	2472	0.052631579
chr12	42601010	42601047	2473	0.052631579
chr12	43396853	43396890	2474	0.052631579
chr12	43949316	43949353	2475	0.052631579
chr12	44945990	44946027	2476	0.052631579
chr12	45550331	45550368	2477	0.052631579
chr12	45794845	45794882	2478	0.052631579
chr12	45873216	45873253	2479	0.052631579
chr12	46153892	46153929	2480	0.052631579
chr12	46704057	46704094	2481	0.052631579
chr12	47167656	47167693	2482	0.052631579
chr12	47444366	47444403	2483	0.052631579
chr12	47507047	47507084	2484	0.052631579
chr12	47507835	47507872	2485	0.052631579
chr12	47620102	47620139	2486	0.052631579
chr12	47772165	47772202	2487	0.052631579
chr12	47780704	47780741	2488	0.052631579
chr12	47811468	47811505	2489	0.052631579
chr12	47865759	47865796	2490	0.052631579
chr12	48072410	48072447	2491	0.052631579
chr12	48221072	48221109	2492	0.052631579
chr12	48319156	48319193	2493	0.052631579
chr12	4833275	4833312	2494	0.052631579
chr12	48342010	48342047	2495	0.052631579
chr12	48685230	48685267	2496	0.052631579
chr12	48696680	48696717	2497	0.052631579
chr12	48939208	48939245	2498	0.052631579
chr12	49165074	49165111	2499	0.052631579
chr12	49414057	49414132	2500	0.052631579

chr12	4946524	4946561	2501	0.052631579
chr12	49523541	49523578	2502	0.052631579
chr12	49636517	49636554	2503	0.052631579
chr12	49790990	49791027	2504	0.052631579
chr12	50070785	50070822	2505	0.052631579
chr12	50255160	50255197	2506	0.052631579
chr12	50486485	50486522	2507	0.052631579
chr12	50722446	50722483	2508	0.052631579
chr12	50844247	50844284	2509	0.052631579
chr12	50868722	50868759	2510	0.052631579
chr12	51002267	51002304	2511	0.052631579
chr12	51056150	51056187	2512	0.052631579
chr12	51270981	51271018	2513	0.052631579
chr12	51891316	51891353	2514	0.052631579
chr12	51892099	51892136	2515	0.052631579
chr12	52167241	52167278	2516	0.052631579
chr12	52393826	52393863	2517	0.052631579
chr12	52469381	52469418	2518	0.052631579
chr12	52489998	52490035	2519	0.052631579
chr12	52522811	52522848	2520	0.052631579
chr12	53157168	53157205	2521	0.052631579
chr12	53222382	53222419	2522	0.052631579
chr12	53230123	53230160	2523	0.052631579
chr12	53337342	53337379	2524	0.052631579
chr12	53361271	53361308	2525	0.052631579
chr12	5420319	5420356	2526	0.052631579
chr12	54519675	54519712	2527	0.052631579
chr12	54527874	54527911	2528	0.052631579
chr12	54631642	54631679	2529	0.052631579
chr12	54833792	54833829	2530	0.052631579
chr12	54840045	54840082	2531	0.052631579
chr12	54861588	54861625	2532	0.052631579
chr12	5500335	5500372	2533	0.052631579
chr12	55128906	55128943	2534	0.052631579
chr12	55129743	55129780	2535	0.052631579
chr12	55151117	55151154	2536	0.052631579
chr12	55324956	55324993	2537	0.052631579
chr12	55744796	55744833	2538	0.052631579
chr12	55832508	55832545	2539	0.052631579
chr12	55834555	55834592	2540	0.052631579
chr12	56468019	56468056	2541	0.052631579
chr12	56502619	56502656	2542	0.052631579
chr12	56695452	56695489	2543	0.052631579
chr12	56916349	56916386	2544	0.052631579
chr12	57323226	57323263	2545	0.052631579
chr12	57601771	57601808	2546	0.052631579
chr12	57780249	57780286	2547	0.052631579
chr12	57781657	57781694	2548	0.052631579
chr12	59029513	59029550	2549	0.052631579
chr12	59077925	59077962	2550	0.052631579

chr12	59372860	59372897	2551	0.052631579
chr12	60205477	60205514	2552	0.052631579
chr12	6033819	6033856	2553	0.052631579
chr12	60397690	60397727	2554	0.052631579
chr12	60702686	60702723	2555	0.052631579
chr12	60803657	60803694	2556	0.052631579
chr12	60931999	60932036	2557	0.052631579
chr12	61073203	61073240	2558	0.052631579
chr12	61244482	61244519	2559	0.052631579
chr12	61283783	61283820	2560	0.052631579
chr12	61435620	61435657	2561	0.052631579
chr12	6179905	6179942	2562	0.052631579
chr12	62226629	62226666	2563	0.052631579
chr12	63104771	63104808	2564	0.052631579
chr12	63177527	63177564	2565	0.052631579
chr12	64783840	64783877	2566	0.052631579
chr12	64906225	64906262	2567	0.052631579
chr12	64907001	64907038	2568	0.052631579
chr12	65029022	65029059	2569	0.052631579
chr12	65832814	65832851	2570	0.052631579
chr12	65868083	65868120	2571	0.052631579
chr12	66842162	66842199	2572	0.052631579
chr12	66916163	66916200	2573	0.052631579
chr12	68490736	68490773	2574	0.052631579
chr12	6849995	6850032	2575	0.052631579
chr12	69201096	69201133	2576	0.052631579
chr12	69242627	69242664	2577	0.052631579
chr12	69340892	69340929	2578	0.052631579
chr12	69915083	69915120	2579	0.052631579
chr12	70264353	70264390	2580	0.052631579
chr12	70264602	70264639	2581	0.052631579
chr12	71649651	71649688	2582	0.052631579
chr12	71803739	71803776	2583	0.052631579
chr12	72475657	72475694	2584	0.052631579
chr12	735535	735572	2585	0.052631579
chr12	73741950	73741987	2586	0.052631579
chr12	73954781	73954818	2587	0.052631579
chr12	74708918	74708955	2588	0.052631579
chr12	77278478	77278515	2589	0.052631579
chr12	79332638	79332675	2590	0.052631579
chr12	79567476	79567513	2591	0.052631579
chr12	811449	811486	2592	0.052631579
chr12	82032508	82032545	2593	0.052631579
chr12	82197578	82197615	2594	0.052631579
chr12	83357948	83357985	2595	0.052631579
chr12	83697411	83697448	2596	0.052631579
chr12	83699282	83699319	2597	0.052631579
chr12	84249090	84249127	2598	0.052631579
chr12	86300133	86300170	2599	0.052631579
chr12	86619621	86619658	2600	0.052631579

chr12	86763289	86763326	2601	0.052631579
chr12	87244580	87244617	2602	0.052631579
chr12	87720240	87720277	2603	0.052631579
chr12	87720352	87720389	2604	0.052631579
chr12	887414	887451	2605	0.052631579
chr12	89029960	89029997	2606	0.052631579
chr12	89312249	89312286	2607	0.052631579
chr12	8987681	8987718	2608	0.052631579
chr12	89914484	89914521	2609	0.052631579
chr12	90134456	90134493	2610	0.052631579
chr12	9038807	9038844	2611	0.052631579
chr12	90480788	90480825	2612	0.052631579
chr12	90534512	90534549	2613	0.052631579
chr12	90721681	90721718	2614	0.052631579
chr12	91265167	91265204	2615	0.052631579
chr12	91385791	91385828	2616	0.052631579
chr12	91436064	91436101	2617	0.052631579
chr12	91663270	91663307	2618	0.052631579
chr12	92555887	92555924	2619	0.052631579
chr12	92891941	92891978	2620	0.052631579
chr12	93110481	93110518	2621	0.052631579
chr12	93717833	93717870	2622	0.052631579
chr12	94187312	94187349	2623	0.052631579
chr12	94433787	94433824	2624	0.052631579
chr12	94803699	94803736	2625	0.052631579
chr12	94811839	94811876	2626	0.052631579
chr12	94980387	94980424	2627	0.052631579
chr12	95891065	95891102	2628	0.052631579
chr12	95903503	95903540	2629	0.052631579
chr12	96547784	96547821	2630	0.052631579
chr12	96675071	96675108	2631	0.052631579
chr12	969527	969564	2632	0.052631579
chr12	97099452	97099489	2633	0.052631579
chr12	97437541	97437578	2634	0.052631579
chr12	97513433	97513470	2635	0.052631579
chr12	97572115	97572152	2636	0.052631579
chr12	97724998	97725035	2637	0.052631579
chr12	97773128	97773165	2638	0.052631579
chr12	98417020	98417057	2639	0.052631579
chr12	9875860	9875897	2640	0.052631579
chr12	98863596	98863633	2641	0.052631579
chr12	99319463	99319500	2642	0.052631579
chr12	99391370	99391407	2643	0.052631579
chr12	99483180	99483217	2644	0.052631579
chr12	99747032	99747069	2645	0.052631579
chr12	99904639	99904676	2646	0.052631579
chr12	45343696	45343770	2647	0.053333333
chr12	131595601	131595668	2648	0.058823529
chr12	49396842	49396907	2649	0.060606061
chr12	17309572	17309631	2650	0.066666667

chr12	63772990	63773034	2651	0.066666667
chr12	78362490	78362543	2652	0.074074074
chr12	104706781	104706820	2653	0.075
chr12	104812997	104813036	2654	0.075
chr12	104940714	104940753	2655	0.075
chr12	105052765	105052804	2656	0.075
chr12	105166362	105166401	2657	0.075
chr12	106540160	106540199	2658	0.075
chr12	106567496	106567535	2659	0.075
chr12	107140149	107140188	2660	0.075
chr12	107185799	107185838	2661	0.075
chr12	108825537	108825576	2662	0.075
chr12	108872720	108872759	2663	0.075
chr12	108979393	108979432	2664	0.075
chr12	109013388	109013427	2665	0.075
chr12	109275414	109275453	2666	0.075
chr12	109775408	109775447	2667	0.075
chr12	109990405	109990444	2668	0.075
chr12	110812624	110812663	2669	0.075
chr12	110865428	110865467	2670	0.075
chr12	111075413	111075452	2671	0.075
chr12	112168408	112168447	2672	0.075
chr12	112792925	112792964	2673	0.075
chr12	112948244	112948283	2674	0.075
chr12	114497195	114497234	2675	0.075
chr12	115120792	115120831	2676	0.075
chr12	115635125	115635164	2677	0.075
chr12	116730807	116730846	2678	0.075
chr12	117026776	117026815	2679	0.075
chr12	117168528	117168567	2680	0.075
chr12	117207217	117207256	2681	0.075
chr12	118661349	118661388	2682	0.075
chr12	11873236	11873275	2683	0.075
chr12	118756454	118756493	2684	0.075
chr12	119144980	119145019	2685	0.075
chr12	119368511	119368550	2686	0.075
chr12	119832050	119832089	2687	0.075
chr12	120274885	120274924	2688	0.075
chr12	120471778	120471817	2689	0.075
chr12	120820329	120820368	2690	0.075
chr12	12136976	12137015	2691	0.075
chr12	122085011	122085050	2692	0.075
chr12	123043661	123043700	2693	0.075
chr12	123963140	123963179	2694	0.075
chr12	124162217	124162256	2695	0.075
chr12	124411048	124411087	2696	0.075
chr12	125274098	125274137	2697	0.075
chr12	128928090	128928129	2698	0.075
chr12	129489163	129489202	2699	0.075
chr12	129830854	129830893	2700	0.075

chr12	130010918	130010957	2701	0.075
chr12	13212009	13212048	2702	0.075
chr12	17138252	17138291	2703	0.075
chr12	19553336	19553375	2704	0.075
chr12	2186251	2186290	2705	0.075
chr12	22588814	22588853	2706	0.075
chr12	22963700	22963739	2707	0.075
chr12	2359513	2359552	2708	0.075
chr12	23670692	23670731	2709	0.075
chr12	24132906	24132945	2710	0.075
chr12	24562711	24562750	2711	0.075
chr12	25105785	25105824	2712	0.075
chr12	25114004	25114043	2713	0.075
chr12	25271492	25271531	2714	0.075
chr12	25689673	25689712	2715	0.075
chr12	2644376	2644415	2716	0.075
chr12	27150189	27150228	2717	0.075
chr12	27430544	27430583	2718	0.075
chr12	2750042	2750081	2719	0.075
chr12	27959458	27959497	2720	0.075
chr12	29366040	29366079	2721	0.075
chr12	30794524	30794563	2722	0.075
chr12	32928932	32928971	2723	0.075
chr12	36842699	36842738	2724	0.075
chr12	37315262	37315301	2725	0.075
chr12	38300307	38300346	2726	0.075
chr12	38547041	38547080	2727	0.075
chr12	38818058	38818097	2728	0.075
chr12	4097170	4097209	2729	0.075
chr12	41458064	41458103	2730	0.075
chr12	4210554	4210593	2731	0.075
chr12	42516758	42516797	2732	0.075
chr12	43173454	43173493	2733	0.075
chr12	43235850	43235889	2734	0.075
chr12	43290923	43290962	2735	0.075
chr12	43966497	43966536	2736	0.075
chr12	44006991	44007030	2737	0.075
chr12	44585407	44585446	2738	0.075
chr12	46829945	46829984	2739	0.075
chr12	47662328	47662367	2740	0.075
chr12	47755666	47755705	2741	0.075
chr12	47868497	47868536	2742	0.075
chr12	48016512	48016551	2743	0.075
chr12	48880878	48880917	2744	0.075
chr12	4916562	4916601	2745	0.075
chr12	49920432	49920471	2746	0.075
chr12	50006847	50006886	2747	0.075
chr12	50084193	50084232	2748	0.075
chr12	50386741	50386780	2749	0.075
chr12	50861566	50861605	2750	0.075

chr12	52063290	52063329	2751	0.075
chr12	52713493	52713532	2752	0.075
chr12	52735226	52735265	2753	0.075
chr12	52875997	52876036	2754	0.075
chr12	53253227	53253266	2755	0.075
chr12	54080950	54080989	2756	0.075
chr12	54824430	54824469	2757	0.075
chr12	54937277	54937316	2758	0.075
chr12	55768659	55768698	2759	0.075
chr12	55859396	55859435	2760	0.075
chr12	55886745	55886784	2761	0.075
chr12	56210417	56210456	2762	0.075
chr12	56509954	56509993	2763	0.075
chr12	5666223	5666262	2764	0.075
chr12	57217120	57217159	2765	0.075
chr12	59406404	59406443	2766	0.075
chr12	62306719	62306758	2767	0.075
chr12	63298751	63298790	2768	0.075
chr12	63354949	63354988	2769	0.075
chr12	63371570	63371609	2770	0.075
chr12	6516130	6516169	2771	0.075
chr12	6517739	6517778	2772	0.075
chr12	652930	652969	2773	0.075
chr12	6567308	6567347	2774	0.075
chr12	66781756	66781795	2775	0.075
chr12	67601119	67601158	2776	0.075
chr12	67769626	67769665	2777	0.075
chr12	67921200	67921239	2778	0.075
chr12	67940110	67940149	2779	0.075
chr12	69842604	69842643	2780	0.075
chr12	69928693	69928732	2781	0.075
chr12	73578207	73578246	2782	0.075
chr12	73662452	73662491	2783	0.075
chr12	73887941	73887980	2784	0.075
chr12	73975383	73975422	2785	0.075
chr12	74244675	74244714	2786	0.075
chr12	75377758	75377797	2787	0.075
chr12	77715806	77715845	2788	0.075
chr12	78061408	78061447	2789	0.075
chr12	78332731	78332770	2790	0.075
chr12	79765202	79765241	2791	0.075
chr12	8025983	8026022	2792	0.075
chr12	80337464	80337503	2793	0.075
chr12	80919939	80919978	2794	0.075
chr12	8103715	8103754	2795	0.075
chr12	81801850	81801889	2796	0.075
chr12	83901217	83901256	2797	0.075
chr12	84002860	84002899	2798	0.075
chr12	85088024	85088063	2799	0.075
chr12	85666163	85666202	2800	0.075

chr12	8904836	8904875	2801	0.075
chr12	89268119	89268158	2802	0.075
chr12	90075035	90075074	2803	0.075
chr12	92045980	92046019	2804	0.075
chr12	92649537	92649576	2805	0.075
chr12	94184316	94184355	2806	0.075
chr12	95015736	95015775	2807	0.075
chr12	95145486	95145525	2808	0.075
chr12	96418098	96418137	2809	0.075
chr12	97096145	97096184	2810	0.075
chr12	97718723	97718762	2811	0.075
chr13	34787768	34787860	2812	0.043010753
chr13	18187285	18187324	2813	0.05
chr13	100634882	100634919	2814	0.052631579
chr13	100953234	100953271	2815	0.052631579
chr13	101217402	101217439	2816	0.052631579
chr13	101500998	101501035	2817	0.052631579
chr13	101714697	101714734	2818	0.052631579
chr13	101799718	101799755	2819	0.052631579
chr13	102108797	102108834	2820	0.052631579
chr13	102400159	102400196	2821	0.052631579
chr13	102739191	102739228	2822	0.052631579
chr13	102835605	102835642	2823	0.052631579
chr13	103257073	103257110	2824	0.052631579
chr13	103333608	103333645	2825	0.052631579
chr13	104633226	104633263	2826	0.052631579
chr13	104721690	104721727	2827	0.052631579
chr13	105152571	105152608	2828	0.052631579
chr13	105176172	105176209	2829	0.052631579
chr13	105261750	105261787	2830	0.052631579
chr13	105973550	105973587	2831	0.052631579
chr13	106017641	106017678	2832	0.052631579
chr13	106018398	106018435	2833	0.052631579
chr13	106266370	106266407	2834	0.052631579
chr13	106386970	106387007	2835	0.052631579
chr13	106405298	106405335	2836	0.052631579
chr13	107148057	107148094	2837	0.052631579
chr13	109061228	109061265	2838	0.052631579
chr13	109556949	109556986	2839	0.052631579
chr13	109917405	109917442	2840	0.052631579
chr13	110519279	110519316	2841	0.052631579
chr13	110596407	110596444	2842	0.052631579
chr13	110597118	110597155	2843	0.052631579
chr13	110621059	110621096	2844	0.052631579
chr13	110719765	110719802	2845	0.052631579
chr13	110982441	110982478	2846	0.052631579
chr13	111817608	111817645	2847	0.052631579
chr13	112027817	112027854	2848	0.052631579
chr13	112667071	112667108	2849	0.052631579
chr13	112676880	112676917	2850	0.052631579

chr13	112910558	112910595	2851	0.052631579
chr13	112980236	112980273	2852	0.052631579
chr13	113025332	113025369	2853	0.052631579
chr13	113277717	113277754	2854	0.052631579
chr13	113495204	113495241	2855	0.052631579
chr13	18425492	18425529	2856	0.052631579
chr13	18426219	18426256	2857	0.052631579
chr13	19064289	19064326	2858	0.052631579
chr13	19598885	19598922	2859	0.052631579
chr13	20280703	20280740	2860	0.052631579
chr13	21002536	21002573	2861	0.052631579
chr13	21317033	21317070	2862	0.052631579
chr13	21317147	21317184	2863	0.052631579
chr13	21356798	21356835	2864	0.052631579
chr13	22569795	22569832	2865	0.052631579
chr13	22569879	22569916	2866	0.052631579
chr13	23116062	23116099	2867	0.052631579
chr13	23418959	23418996	2868	0.052631579
chr13	23669415	23669452	2869	0.052631579
chr13	24164628	24164665	2870	0.052631579
chr13	24569641	24569678	2871	0.052631579
chr13	24738501	24738538	2872	0.052631579
chr13	25002205	25002242	2873	0.052631579
chr13	25096333	25096370	2874	0.052631579
chr13	26074939	26074976	2875	0.052631579
chr13	26158790	26158827	2876	0.052631579
chr13	26808067	26808104	2877	0.052631579
chr13	26940537	26940574	2878	0.052631579
chr13	27104679	27104716	2879	0.052631579
chr13	27168594	27168631	2880	0.052631579
chr13	27168723	27168760	2881	0.052631579
chr13	27191036	27191073	2882	0.052631579
chr13	27200845	27200882	2883	0.052631579
chr13	27660965	27661002	2884	0.052631579
chr13	27938723	27938760	2885	0.052631579
chr13	27939258	27939295	2886	0.052631579
chr13	28016414	28016451	2887	0.052631579
chr13	28964706	28964743	2888	0.052631579
chr13	28965450	28965487	2889	0.052631579
chr13	29040360	29040397	2890	0.052631579
chr13	29167810	29167847	2891	0.052631579
chr13	29184502	29184539	2892	0.052631579
chr13	29583025	29583062	2893	0.052631579
chr13	29749275	29749312	2894	0.052631579
chr13	29931474	29931511	2895	0.052631579
chr13	31244627	31244664	2896	0.052631579
chr13	32630660	32630697	2897	0.052631579
chr13	32843569	32843606	2898	0.052631579
chr13	32907346	32907383	2899	0.052631579
chr13	33100727	33100764	2900	0.052631579

chr13	33184041	33184078	2901	0.052631579
chr13	33184192	33184229	2902	0.052631579
chr13	33416349	33416386	2903	0.052631579
chr13	34772140	34772177	2904	0.052631579
chr13	34772239	34772276	2905	0.052631579
chr13	34942030	34942067	2906	0.052631579
chr13	35303788	35303825	2907	0.052631579
chr13	35304599	35304636	2908	0.052631579
chr13	35630591	35630628	2909	0.052631579
chr13	35672543	35672580	2910	0.052631579
chr13	36179348	36179385	2911	0.052631579
chr13	36237978	36238015	2912	0.052631579
chr13	36238833	36238870	2913	0.052631579
chr13	36634622	36634659	2914	0.052631579
chr13	37195125	37195162	2915	0.052631579
chr13	37343507	37343544	2916	0.052631579
chr13	37904681	37904718	2917	0.052631579
chr13	38206573	38206610	2918	0.052631579
chr13	38258726	38258763	2919	0.052631579
chr13	38596562	38596599	2920	0.052631579
chr13	38985266	38985303	2921	0.052631579
chr13	39561520	39561557	2922	0.052631579
chr13	39858294	39858331	2923	0.052631579
chr13	39887147	39887184	2924	0.052631579
chr13	40219439	40219476	2925	0.052631579
chr13	40930502	40930539	2926	0.052631579
chr13	41398292	41398329	2927	0.052631579
chr13	41688623	41688660	2928	0.052631579
chr13	42074956	42074993	2929	0.052631579
chr13	42257615	42257652	2930	0.052631579
chr13	42446962	42446999	2931	0.052631579
chr13	42770439	42770476	2932	0.052631579
chr13	42921861	42921898	2933	0.052631579
chr13	43463489	43463526	2934	0.052631579
chr13	44801836	44801873	2935	0.052631579
chr13	45291394	45291431	2936	0.052631579
chr13	46194944	46194981	2937	0.052631579
chr13	46213290	46213327	2938	0.052631579
chr13	47607670	47607707	2939	0.052631579
chr13	48781494	48781531	2940	0.052631579
chr13	49510845	49510882	2941	0.052631579
chr13	49733417	49733454	2942	0.052631579
chr13	50438066	50438103	2943	0.052631579
chr13	50723773	50723810	2944	0.052631579
chr13	50765280	50765317	2945	0.052631579
chr13	50895692	50895729	2946	0.052631579
chr13	50896407	50896444	2947	0.052631579
chr13	50972765	50972802	2948	0.052631579
chr13	51413118	51413155	2949	0.052631579
chr13	51937822	51937859	2950	0.052631579

chr13	52902223	52902260	2951	0.052631579
chr13	52902946	52902983	2952	0.052631579
chr13	53653256	53653293	2953	0.052631579
chr13	54956120	54956157	2954	0.052631579
chr13	55531785	55531822	2955	0.052631579
chr13	55885753	55885790	2956	0.052631579
chr13	56620451	56620488	2957	0.052631579
chr13	57906817	57906854	2958	0.052631579
chr13	58708505	58708542	2959	0.052631579
chr13	59036722	59036759	2960	0.052631579
chr13	59138894	59138931	2961	0.052631579
chr13	59409954	59409991	2962	0.052631579
chr13	59651956	59651993	2963	0.052631579
chr13	60088464	60088501	2964	0.052631579
chr13	60639514	60639551	2965	0.052631579
chr13	60655908	60655945	2966	0.052631579
chr13	61614432	61614469	2967	0.052631579
chr13	62178977	62179014	2968	0.052631579
chr13	62546221	62546258	2969	0.052631579
chr13	62546380	62546417	2970	0.052631579
chr13	63211228	63211265	2971	0.052631579
chr13	63340862	63340899	2972	0.052631579
chr13	63903964	63904001	2973	0.052631579
chr13	64672749	64672786	2974	0.052631579
chr13	65851750	65851787	2975	0.052631579
chr13	66791929	66791966	2976	0.052631579
chr13	67012036	67012073	2977	0.052631579
chr13	67654672	67654709	2978	0.052631579
chr13	67773589	67773626	2979	0.052631579
chr13	67842410	67842447	2980	0.052631579
chr13	69135911	69135948	2981	0.052631579
chr13	70377277	70377314	2982	0.052631579
chr13	71176293	71176330	2983	0.052631579
chr13	71217451	71217488	2984	0.052631579
chr13	71624568	71624605	2985	0.052631579
chr13	72135286	72135323	2986	0.052631579
chr13	72330707	72330744	2987	0.052631579
chr13	72378250	72378287	2988	0.052631579
chr13	72378966	72379003	2989	0.052631579
chr13	72464534	72464571	2990	0.052631579
chr13	72501512	72501549	2991	0.052631579
chr13	72534640	72534677	2992	0.052631579
chr13	73365035	73365072	2993	0.052631579
chr13	73504973	73505010	2994	0.052631579
chr13	73889489	73889526	2995	0.052631579
chr13	75858366	75858403	2996	0.052631579
chr13	76636620	76636657	2997	0.052631579
chr13	76949187	76949224	2998	0.052631579
chr13	77018188	77018225	2999	0.052631579
chr13	77019003	77019040	3000	0.052631579

chr13	77030476	77030513	3001	0.052631579
chr13	77557776	77557813	3002	0.052631579
chr13	77826134	77826171	3003	0.052631579
chr13	78039729	78039766	3004	0.052631579
chr13	78515957	78515994	3005	0.052631579
chr13	78622900	78622937	3006	0.052631579
chr13	78699604	78699641	3007	0.052631579
chr13	78739548	78739585	3008	0.052631579
chr13	79408786	79408823	3009	0.052631579
chr13	79740585	79740622	3010	0.052631579
chr13	79769547	79769584	3011	0.052631579
chr13	80033147	80033184	3012	0.052631579
chr13	81002194	81002231	3013	0.052631579
chr13	81199465	81199502	3014	0.052631579
chr13	81628999	81629036	3015	0.052631579
chr13	81989103	81989140	3016	0.052631579
chr13	82878045	82878082	3017	0.052631579
chr13	83490664	83490701	3018	0.052631579
chr13	84006790	84006827	3019	0.052631579
chr13	84272533	84272570	3020	0.052631579
chr13	84449790	84449827	3021	0.052631579
chr13	84900042	84900079	3022	0.052631579
chr13	85025213	85025250	3023	0.052631579
chr13	85047095	85047132	3024	0.052631579
chr13	85607354	85607391	3025	0.052631579
chr13	86669569	86669606	3026	0.052631579
chr13	87126557	87126594	3027	0.052631579
chr13	87441557	87441594	3028	0.052631579
chr13	88963789	88963826	3029	0.052631579
chr13	89819045	89819082	3030	0.052631579
chr13	90043708	90043745	3031	0.052631579
chr13	90376582	90376619	3032	0.052631579
chr13	90377352	90377389	3033	0.052631579
chr13	90478695	90478732	3034	0.052631579
chr13	90808051	90808088	3035	0.052631579
chr13	91003348	91003385	3036	0.052631579
chr13	91262260	91262297	3037	0.052631579
chr13	91385119	91385156	3038	0.052631579
chr13	91762881	91762918	3039	0.052631579
chr13	92676454	92676491	3040	0.052631579
chr13	92799481	92799518	3041	0.052631579
chr13	93094080	93094117	3042	0.052631579
chr13	93227536	93227573	3043	0.052631579
chr13	93240714	93240751	3044	0.052631579
chr13	93315656	93315693	3045	0.052631579
chr13	93458243	93458280	3046	0.052631579
chr13	93733239	93733276	3047	0.052631579
chr13	93781026	93781063	3048	0.052631579
chr13	94156920	94156957	3049	0.052631579
chr13	94550805	94550842	3050	0.052631579

chr13	94589535	94589572	3051	0.052631579
chr13	94838335	94838372	3052	0.052631579
chr13	95004174	95004211	3053	0.052631579
chr13	95004712	95004749	3054	0.052631579
chr13	96313336	96313373	3055	0.052631579
chr13	96359721	96359758	3056	0.052631579
chr13	96393337	96393374	3057	0.052631579
chr13	96793917	96793954	3058	0.052631579
chr13	96977473	96977510	3059	0.052631579
chr13	97156651	97156688	3060	0.052631579
chr13	97421429	97421466	3061	0.052631579
chr13	97440697	97440734	3062	0.052631579
chr13	97846314	97846351	3063	0.052631579
chr13	97854490	97854527	3064	0.052631579
chr13	97889398	97889435	3065	0.052631579
chr13	99064082	99064119	3066	0.052631579
chr13	99260999	99261036	3067	0.052631579
chr13	99261830	99261867	3068	0.052631579
chr13	99312280	99312317	3069	0.052631579
chr13	99987706	99987743	3070	0.052631579
chr13	38065108	38065178	3071	0.056338028
chr13	110421300	110421351	3072	0.057692308
chr13	72884931	72884989	3073	0.06779661
chr13	58615519	58615589	3074	0.070422535
chr13	100166839	100166878	3075	0.075
chr13	100354288	100354327	3076	0.075
chr13	102875774	102875813	3077	0.075
chr13	104325135	104325174	3078	0.075
chr13	104728462	104728501	3079	0.075
chr13	105462686	105462725	3080	0.075
chr13	105564078	105564117	3081	0.075
chr13	105891656	105891695	3082	0.075
chr13	106953344	106953383	3083	0.075
chr13	108261750	108261789	3084	0.075
chr13	109250925	109250964	3085	0.075
chr13	109289559	109289598	3086	0.075
chr13	109467957	109467996	3087	0.075
chr13	109625651	109625690	3088	0.075
chr13	109930737	109930776	3089	0.075
chr13	109958417	109958456	3090	0.075
chr13	110656809	110656848	3091	0.075
chr13	112400906	112400945	3092	0.075
chr13	113544728	113544767	3093	0.075
chr13	19638581	19638620	3094	0.075
chr13	19940567	19940606	3095	0.075
chr13	20552363	20552402	3096	0.075
chr13	20626625	20626664	3097	0.075
chr13	20786468	20786507	3098	0.075
chr13	23143063	23143102	3099	0.075
chr13	23205738	23205777	3100	0.075

chr13	24361944	24361983	3101	0.075
chr13	24703815	24703854	3102	0.075
chr13	25364957	25364996	3103	0.075
chr13	25496424	25496463	3104	0.075
chr13	26260545	26260584	3105	0.075
chr13	26842986	26843025	3106	0.075
chr13	27274036	27274075	3107	0.075
chr13	27518314	27518353	3108	0.075
chr13	28973890	28973929	3109	0.075
chr13	30380155	30380194	3110	0.075
chr13	30584422	30584461	3111	0.075
chr13	31598102	31598141	3112	0.075
chr13	33252485	33252524	3113	0.075
chr13	33890178	33890217	3114	0.075
chr13	34424473	34424512	3115	0.075
chr13	35335876	35335915	3116	0.075
chr13	37114169	37114208	3117	0.075
chr13	38079743	38079782	3118	0.075
chr13	38968595	38968634	3119	0.075
chr13	39550431	39550470	3120	0.075
chr13	39602884	39602923	3121	0.075
chr13	40857128	40857167	3122	0.075
chr13	41653541	41653580	3123	0.075
chr13	41912054	41912093	3124	0.075
chr13	42003052	42003091	3125	0.075
chr13	44515023	44515062	3126	0.075
chr13	46152273	46152312	3127	0.075
chr13	46609988	46610027	3128	0.075
chr13	48243353	48243392	3129	0.075
chr13	48695904	48695943	3130	0.075
chr13	49279114	49279153	3131	0.075
chr13	51501006	51501045	3132	0.075
chr13	52220792	52220831	3133	0.075
chr13	52641523	52641562	3134	0.075
chr13	52912929	52912968	3135	0.075
chr13	54064569	54064608	3136	0.075
chr13	56437126	56437165	3137	0.075
chr13	56542093	56542132	3138	0.075
chr13	56988741	56988780	3139	0.075
chr13	58924826	58924865	3140	0.075
chr13	60020246	60020285	3141	0.075
chr13	64661168	64661207	3142	0.075
chr13	64837459	64837498	3143	0.075
chr13	66622915	66622954	3144	0.075
chr13	68799483	68799522	3145	0.075
chr13	70540441	70540480	3146	0.075
chr13	71913320	71913359	3147	0.075
chr13	73458171	73458210	3148	0.075
chr13	73994718	73994757	3149	0.075
chr13	74026981	74027020	3150	0.075

chr13	74188053	74188092	3151	0.075
chr13	74247341	74247380	3152	0.075
chr13	76357327	76357366	3153	0.075
chr13	76471445	76471484	3154	0.075
chr13	76758409	76758448	3155	0.075
chr13	79592321	79592360	3156	0.075
chr13	80218303	80218342	3157	0.075
chr13	81639401	81639440	3158	0.075
chr13	82611393	82611432	3159	0.075
chr13	83828294	83828333	3160	0.075
chr13	85917735	85917774	3161	0.075
chr13	88013026	88013065	3162	0.075
chr13	89663619	89663658	3163	0.075
chr13	90402107	90402146	3164	0.075
chr13	92959659	92959698	3165	0.075
chr13	93509115	93509154	3166	0.075
chr13	93684555	93684594	3167	0.075
chr13	94065880	94065919	3168	0.075
chr13	94112729	94112768	3169	0.075
chr13	94397982	94398021	3170	0.075
chr13	94416903	94416942	3171	0.075
chr13	95027327	95027366	3172	0.075
chr13	96488927	96488966	3173	0.075
chr13	96916696	96916735	3174	0.075
chr13	97912470	97912509	3175	0.075
chr13	99044328	99044367	3176	0.075
chr13	52565750	52565801	3177	0.076923077
chr13	44812854	44812893	3178	0.1
chr14	78810819	78810932	3179	0.035087719
chr14	60051825	60051935	3180	0.036036036
chr14	100111221	100111258	3181	0.052631579
chr14	100195212	100195249	3182	0.052631579
chr14	100350874	100350911	3183	0.052631579
chr14	100378285	100378322	3184	0.052631579
chr14	100387352	100387389	3185	0.052631579
chr14	100388149	100388186	3186	0.052631579
chr14	100564368	100564405	3187	0.052631579
chr14	100791114	100791151	3188	0.052631579
chr14	101083539	101083576	3189	0.052631579
chr14	101859106	101859143	3190	0.052631579
chr14	101899120	101899157	3191	0.052631579
chr14	102027811	102027848	3192	0.052631579
chr14	102872858	102872895	3193	0.052631579
chr14	103215535	103215572	3194	0.052631579
chr14	103427315	103427352	3195	0.052631579
chr14	103466905	103466942	3196	0.052631579
chr14	103984049	103984086	3197	0.052631579
chr14	103984854	103984891	3198	0.052631579
chr14	104248270	104248307	3199	0.052631579
chr14	104261751	104261788	3200	0.052631579

chr14	104312558	104312595	3201	0.052631579
chr14	104850210	104850247	3202	0.052631579
chr14	104905466	104905503	3203	0.052631579
chr14	105164860	105164897	3204	0.052631579
chr14	105313052	105313089	3205	0.052631579
chr14	105524765	105524802	3206	0.052631579
chr14	105845314	105845351	3207	0.052631579
chr14	18407152	18407189	3208	0.052631579
chr14	18463382	18463419	3209	0.052631579
chr14	18514190	18514227	3210	0.052631579
chr14	18719692	18719729	3211	0.052631579
chr14	19207523	19207560	3212	0.052631579
chr14	19435395	19435432	3213	0.052631579
chr14	19837086	19837123	3214	0.052631579
chr14	19854429	19854466	3215	0.052631579
chr14	19864610	19864647	3216	0.052631579
chr14	20214105	20214142	3217	0.052631579
chr14	20566767	20566804	3218	0.052631579
chr14	20776546	20776583	3219	0.052631579
chr14	21194007	21194044	3220	0.052631579
chr14	21406021	21406058	3221	0.052631579
chr14	21645742	21645779	3222	0.052631579
chr14	21919566	21919603	3223	0.052631579
chr14	22058825	22058862	3224	0.052631579
chr14	22067248	22067285	3225	0.052631579
chr14	22080389	22080426	3226	0.052631579
chr14	22080554	22080591	3227	0.052631579
chr14	22382884	22382921	3228	0.052631579
chr14	22654495	22654532	3229	0.052631579
chr14	22708997	22709034	3230	0.052631579
chr14	22710987	22711024	3231	0.052631579
chr14	22847526	22847563	3232	0.052631579
chr14	22848528	22848565	3233	0.052631579
chr14	22917399	22917436	3234	0.052631579
chr14	22932479	22932516	3235	0.052631579
chr14	22954471	22954508	3236	0.052631579
chr14	22962684	22962721	3237	0.052631579
chr14	23200134	23200171	3238	0.052631579
chr14	23476897	23476934	3239	0.052631579
chr14	23639093	23639130	3240	0.052631579
chr14	23839928	23839965	3241	0.052631579
chr14	23840692	23840729	3242	0.052631579
chr14	24395798	24395835	3243	0.052631579
chr14	24591691	24591728	3244	0.052631579
chr14	24777616	24777653	3245	0.052631579
chr14	25167596	25167633	3246	0.052631579
chr14	25168247	25168284	3247	0.052631579
chr14	25236140	25236177	3248	0.052631579
chr14	25674275	25674312	3249	0.052631579
chr14	25754153	25754190	3250	0.052631579

chr14	26480587	26480624	3251	0.052631579
chr14	26751940	26751977	3252	0.052631579
chr14	27625384	27625421	3253	0.052631579
chr14	27628255	27628292	3254	0.052631579
chr14	28297728	28297765	3255	0.052631579
chr14	28759502	28759539	3256	0.052631579
chr14	29375130	29375167	3257	0.052631579
chr14	30097753	30097790	3258	0.052631579
chr14	30230415	30230452	3259	0.052631579
chr14	30381155	30381192	3260	0.052631579
chr14	30649425	30649462	3261	0.052631579
chr14	30735881	30735918	3262	0.052631579
chr14	30861232	30861269	3263	0.052631579
chr14	31225307	31225344	3264	0.052631579
chr14	31744893	31744930	3265	0.052631579
chr14	32068944	32068981	3266	0.052631579
chr14	32069716	32069753	3267	0.052631579
chr14	32407356	32407393	3268	0.052631579
chr14	32526300	32526337	3269	0.052631579
chr14	32982402	32982439	3270	0.052631579
chr14	32988882	32988919	3271	0.052631579
chr14	33313021	33313058	3272	0.052631579
chr14	33313803	33313840	3273	0.052631579
chr14	33316973	33317010	3274	0.052631579
chr14	33397666	33397703	3275	0.052631579
chr14	34561990	34562027	3276	0.052631579
chr14	34562127	34562164	3277	0.052631579
chr14	34638554	34638591	3278	0.052631579
chr14	34664628	34664665	3279	0.052631579
chr14	35144638	35144675	3280	0.052631579
chr14	35806165	35806202	3281	0.052631579
chr14	36206675	36206712	3282	0.052631579
chr14	36670590	36670627	3283	0.052631579
chr14	36673671	36673708	3284	0.052631579
chr14	37140482	37140519	3285	0.052631579
chr14	37686203	37686240	3286	0.052631579
chr14	38197017	38197054	3287	0.052631579
chr14	38229392	38229429	3288	0.052631579
chr14	38874052	38874089	3289	0.052631579
chr14	39300445	39300482	3290	0.052631579
chr14	39912082	39912119	3291	0.052631579
chr14	40128644	40128681	3292	0.052631579
chr14	40596171	40596208	3293	0.052631579
chr14	40606462	40606499	3294	0.052631579
chr14	41091698	41091735	3295	0.052631579
chr14	41721639	41721676	3296	0.052631579
chr14	43333323	43333360	3297	0.052631579
chr14	43588365	43588402	3298	0.052631579
chr14	44292110	44292147	3299	0.052631579
chr14	44304608	44304645	3300	0.052631579

chr14	44771392	44771429	3301	0.052631579
chr14	46456229	46456266	3302	0.052631579
chr14	47451104	47451141	3303	0.052631579
chr14	47894610	47894647	3304	0.052631579
chr14	48205653	48205690	3305	0.052631579
chr14	48442564	48442601	3306	0.052631579
chr14	48596480	48596517	3307	0.052631579
chr14	48907932	48907969	3308	0.052631579
chr14	49013910	49013947	3309	0.052631579
chr14	49175482	49175519	3310	0.052631579
chr14	49696201	49696238	3311	0.052631579
chr14	51044925	51044962	3312	0.052631579
chr14	51551634	51551671	3313	0.052631579
chr14	51826400	51826437	3314	0.052631579
chr14	51863769	51863806	3315	0.052631579
chr14	52274579	52274616	3316	0.052631579
chr14	52580416	52580453	3317	0.052631579
chr14	52583359	52583396	3318	0.052631579
chr14	52777679	52777716	3319	0.052631579
chr14	53387729	53387766	3320	0.052631579
chr14	53886700	53886737	3321	0.052631579
chr14	53886914	53886951	3322	0.052631579
chr14	54138581	54138618	3323	0.052631579
chr14	54271061	54271098	3324	0.052631579
chr14	54271819	54271856	3325	0.052631579
chr14	54297423	54297460	3326	0.052631579
chr14	54663887	54663924	3327	0.052631579
chr14	54863120	54863157	3328	0.052631579
chr14	54887699	54887736	3329	0.052631579
chr14	55640182	55640219	3330	0.052631579
chr14	55640646	55640683	3331	0.052631579
chr14	55775192	55775229	3332	0.052631579
chr14	55948627	55948664	3333	0.052631579
chr14	56127118	56127155	3334	0.052631579
chr14	56184711	56184748	3335	0.052631579
chr14	56305121	56305158	3336	0.052631579
chr14	56305304	56305341	3337	0.052631579
chr14	56398401	56398438	3338	0.052631579
chr14	56409828	56409865	3339	0.052631579
chr14	56515795	56515832	3340	0.052631579
chr14	58088338	58088375	3341	0.052631579
chr14	58137400	58137437	3342	0.052631579
chr14	58212544	58212581	3343	0.052631579
chr14	58892047	58892084	3344	0.052631579
chr14	59523348	59523385	3345	0.052631579
chr14	59688474	59688511	3346	0.052631579
chr14	59914717	59914754	3347	0.052631579
chr14	60078067	60078104	3348	0.052631579
chr14	60648973	60649010	3349	0.052631579
chr14	61145347	61145384	3350	0.052631579

chr14	61831667	61831704	3351	0.052631579
chr14	62260080	62260117	3352	0.052631579
chr14	62305723	62305760	3353	0.052631579
chr14	62384871	62384908	3354	0.052631579
chr14	63478341	63478378	3355	0.052631579
chr14	63761908	63761945	3356	0.052631579
chr14	63942086	63942123	3357	0.052631579
chr14	64051535	64051572	3358	0.052631579
chr14	64295753	64295790	3359	0.052631579
chr14	64296642	64296679	3360	0.052631579
chr14	64316204	64316241	3361	0.052631579
chr14	64354447	64354484	3362	0.052631579
chr14	64476175	64476212	3363	0.052631579
chr14	64866860	64866897	3364	0.052631579
chr14	65441862	65441899	3365	0.052631579
chr14	65442123	65442160	3366	0.052631579
chr14	65456920	65456957	3367	0.052631579
chr14	65609027	65609064	3368	0.052631579
chr14	65941885	65941922	3369	0.052631579
chr14	66646654	66646691	3370	0.052631579
chr14	66936744	66936781	3371	0.052631579
chr14	67054506	67054543	3372	0.052631579
chr14	67115059	67115096	3373	0.052631579
chr14	67197100	67197137	3374	0.052631579
chr14	67527095	67527132	3375	0.052631579
chr14	67578877	67578914	3376	0.052631579
chr14	68228811	68228848	3377	0.052631579
chr14	68326548	68326585	3378	0.052631579
chr14	68420542	68420579	3379	0.052631579
chr14	68489709	68489746	3380	0.052631579
chr14	68570716	68570753	3381	0.052631579
chr14	68983606	68983643	3382	0.052631579
chr14	69206636	69206673	3383	0.052631579
chr14	69784111	69784148	3384	0.052631579
chr14	70462104	70462141	3385	0.052631579
chr14	71198373	71198410	3386	0.052631579
chr14	71267251	71267288	3387	0.052631579
chr14	71315739	71315776	3388	0.052631579
chr14	71443462	71443499	3389	0.052631579
chr14	71537461	71537498	3390	0.052631579
chr14	71865637	71865674	3391	0.052631579
chr14	72006495	72006532	3392	0.052631579
chr14	72131924	72131961	3393	0.052631579
chr14	72156975	72157012	3394	0.052631579
chr14	72190245	72190282	3395	0.052631579
chr14	72251531	72251568	3396	0.052631579
chr14	72289778	72289815	3397	0.052631579
chr14	72429952	72429989	3398	0.052631579
chr14	72495778	72495815	3399	0.052631579
chr14	72806243	72806280	3400	0.052631579

chr14	74039376	74039413	3401	0.052631579
chr14	74206444	74206481	3402	0.052631579
chr14	74449658	74449695	3403	0.052631579
chr14	74553473	74553510	3404	0.052631579
chr14	75015686	75015723	3405	0.052631579
chr14	75416331	75416368	3406	0.052631579
chr14	75733727	75733764	3407	0.052631579
chr14	76430898	76430935	3408	0.052631579
chr14	76562215	76562252	3409	0.052631579
chr14	76573751	76573788	3410	0.052631579
chr14	76624402	76624439	3411	0.052631579
chr14	77012171	77012208	3412	0.052631579
chr14	77047523	77047560	3413	0.052631579
chr14	77291206	77291243	3414	0.052631579
chr14	77795922	77795959	3415	0.052631579
chr14	78100498	78100535	3416	0.052631579
chr14	78118474	78118511	3417	0.052631579
chr14	78459216	78459253	3418	0.052631579
chr14	79367370	79367407	3419	0.052631579
chr14	79393045	79393082	3420	0.052631579
chr14	79755447	79755484	3421	0.052631579
chr14	80196388	80196425	3422	0.052631579
chr14	80712453	80712490	3423	0.052631579
chr14	80749029	80749066	3424	0.052631579
chr14	81064793	81064830	3425	0.052631579
chr14	81421535	81421572	3426	0.052631579
chr14	82209944	82209981	3427	0.052631579
chr14	82295545	82295582	3428	0.052631579
chr14	82381521	82381558	3429	0.052631579
chr14	82451625	82451662	3430	0.052631579
chr14	82847439	82847476	3431	0.052631579
chr14	83124214	83124251	3432	0.052631579
chr14	83148215	83148252	3433	0.052631579
chr14	83403171	83403208	3434	0.052631579
chr14	84930573	84930610	3435	0.052631579
chr14	85015413	85015450	3436	0.052631579
chr14	85116421	85116458	3437	0.052631579
chr14	85886875	85886912	3438	0.052631579
chr14	85887589	85887626	3439	0.052631579
chr14	86600836	86600873	3440	0.052631579
chr14	87033013	87033050	3441	0.052631579
chr14	87371488	87371525	3442	0.052631579
chr14	88090374	88090411	3443	0.052631579
chr14	88414332	88414369	3444	0.052631579
chr14	88414454	88414491	3445	0.052631579
chr14	88597710	88597747	3446	0.052631579
chr14	88739251	88739288	3447	0.052631579
chr14	89826394	89826431	3448	0.052631579
chr14	90001136	90001173	3449	0.052631579
chr14	90059333	90059370	3450	0.052631579

chr14	90060146	90060183	3451	0.052631579
chr14	90207720	90207757	3452	0.052631579
chr14	92423575	92423612	3453	0.052631579
chr14	92972442	92972479	3454	0.052631579
chr14	92972549	92972586	3455	0.052631579
chr14	93153023	93153060	3456	0.052631579
chr14	93221878	93221915	3457	0.052631579
chr14	93673043	93673080	3458	0.052631579
chr14	94207414	94207451	3459	0.052631579
chr14	94301959	94301996	3460	0.052631579
chr14	94395727	94395764	3461	0.052631579
chr14	94653814	94653851	3462	0.052631579
chr14	94950735	94950772	3463	0.052631579
chr14	94962094	94962131	3464	0.052631579
chr14	95742180	95742217	3465	0.052631579
chr14	96101089	96101126	3466	0.052631579
chr14	96367379	96367416	3467	0.052631579
chr14	97249211	97249248	3468	0.052631579
chr14	97376498	97376535	3469	0.052631579
chr14	97438158	97438195	3470	0.052631579
chr14	97884017	97884054	3471	0.052631579
chr14	97905019	97905056	3472	0.052631579
chr14	98325748	98325785	3473	0.052631579
chr14	98399532	98399569	3474	0.052631579
chr14	98440176	98440213	3475	0.052631579
chr14	98616831	98616868	3476	0.052631579
chr14	99160800	99160837	3477	0.052631579
chr14	99668329	99668366	3478	0.052631579
chr14	99669036	99669073	3479	0.052631579
chr14	99869801	99869838	3480	0.052631579
chr14	99959163	99959200	3481	0.052631579
chr14	80966981	80967037	3482	0.070175439
chr14	100761445	100761484	3483	0.075
chr14	101676057	101676096	3484	0.075
chr14	102666218	102666257	3485	0.075
chr14	103056041	103056080	3486	0.075
chr14	103056571	103056610	3487	0.075
chr14	103064658	103064697	3488	0.075
chr14	104490741	104490780	3489	0.075
chr14	105027468	105027507	3490	0.075
chr14	105119120	105119159	3491	0.075
chr14	106106291	106106330	3492	0.075
chr14	18517546	18517585	3493	0.075
chr14	20640373	20640412	3494	0.075
chr14	22385418	22385457	3495	0.075
chr14	22610393	22610432	3496	0.075
chr14	22812025	22812064	3497	0.075
chr14	22863605	22863644	3498	0.075
chr14	23066666	23066705	3499	0.075
chr14	23085070	23085109	3500	0.075

chr14	23269089	23269128	3501	0.075
chr14	23599712	23599751	3502	0.075
chr14	23915087	23915126	3503	0.075
chr14	25344573	25344612	3504	0.075
chr14	26195535	26195574	3505	0.075
chr14	28970776	28970815	3506	0.075
chr14	32164337	32164376	3507	0.075
chr14	32471860	32471899	3508	0.075
chr14	32592168	32592207	3509	0.075
chr14	34523358	34523397	3510	0.075
chr14	34570675	34570714	3511	0.075
chr14	36224212	36224251	3512	0.075
chr14	36683221	36683260	3513	0.075
chr14	41144914	41144953	3514	0.075
chr14	41314620	41314659	3515	0.075
chr14	41737845	41737884	3516	0.075
chr14	44660488	44660527	3517	0.075
chr14	45390850	45390889	3518	0.075
chr14	46379835	46379874	3519	0.075
chr14	50250600	50250639	3520	0.075
chr14	51605474	51605513	3521	0.075
chr14	55508696	55508735	3522	0.075
chr14	57517570	57517609	3523	0.075
chr14	58425445	58425484	3524	0.075
chr14	59851884	59851923	3525	0.075
chr14	60617380	60617419	3526	0.075
chr14	60763228	60763267	3527	0.075
chr14	61046484	61046523	3528	0.075
chr14	61193915	61193954	3529	0.075
chr14	63898742	63898781	3530	0.075
chr14	64078360	64078399	3531	0.075
chr14	64191728	64191767	3532	0.075
chr14	64404969	64405008	3533	0.075
chr14	67051933	67051972	3534	0.075
chr14	67108666	67108705	3535	0.075
chr14	67323099	67323138	3536	0.075
chr14	68012877	68012916	3537	0.075
chr14	68148483	68148522	3538	0.075
chr14	69231702	69231741	3539	0.075
chr14	71154975	71155014	3540	0.075
chr14	71977214	71977253	3541	0.075
chr14	72254621	72254660	3542	0.075
chr14	72269734	72269773	3543	0.075
chr14	72364544	72364583	3544	0.075
chr14	72905110	72905149	3545	0.075
chr14	73027652	73027691	3546	0.075
chr14	73045688	73045727	3547	0.075
chr14	73601747	73601786	3548	0.075
chr14	73784343	73784382	3549	0.075
chr14	73863173	73863212	3550	0.075

chr14	74498085	74498124	3551	0.075
chr14	74946619	74946658	3552	0.075
chr14	75668556	75668595	3553	0.075
chr14	75764807	75764846	3554	0.075
chr14	75870675	75870714	3555	0.075
chr14	76034510	76034549	3556	0.075
chr14	77768355	77768394	3557	0.075
chr14	77857999	77858038	3558	0.075
chr14	77884441	77884480	3559	0.075
chr14	78563395	78563434	3560	0.075
chr14	78742779	78742818	3561	0.075
chr14	81006511	81006550	3562	0.075
chr14	82785498	82785537	3563	0.075
chr14	84830550	84830589	3564	0.075
chr14	88253108	88253147	3565	0.075
chr14	90597125	90597164	3566	0.075
chr14	92448955	92448994	3567	0.075
chr14	92780330	92780369	3568	0.075
chr14	93212672	93212711	3569	0.075
chr14	95418911	95418950	3570	0.075
chr14	95624049	95624088	3571	0.075
chr14	96515996	96516035	3572	0.075
chr14	97087279	97087318	3573	0.075
chr14	97289503	97289542	3574	0.075
chr14	97380398	97380437	3575	0.075
chr14	98109418	98109457	3576	0.075
chr14	98169662	98169701	3577	0.075
chr14	98600847	98600886	3578	0.075
chr14	98926281	98926320	3579	0.075
chr14	99062482	99062521	3580	0.075
chr14	99863677	99863716	3581	0.075
chr15	97813666	97813781	3582	0.034482759
chr15	24151074	24151184	3583	0.036036036
chr15	41873302	41873382	3584	0.037037037
chr15	70903890	70903979	3585	0.044444444
chr15	18676327	18676366	3586	0.05
chr15	100148992	100149029	3587	0.052631579
chr15	18737755	18737792	3588	0.052631579
chr15	19283490	19283527	3589	0.052631579
chr15	19878795	19878832	3590	0.052631579
chr15	20412094	20412131	3591	0.052631579
chr15	20414207	20414244	3592	0.052631579
chr15	21238780	21238817	3593	0.052631579
chr15	21766969	21767006	3594	0.052631579
chr15	22771693	22771730	3595	0.052631579
chr15	22790556	22790593	3596	0.052631579
chr15	22866815	22866852	3597	0.052631579
chr15	22981655	22981692	3598	0.052631579
chr15	23171959	23171996	3599	0.052631579
chr15	23261822	23261859	3600	0.052631579

chr15	23570980	23571017	3601	0.052631579
chr15	23655274	23655311	3602	0.052631579
chr15	24353930	24353967	3603	0.052631579
chr15	24482635	24482672	3604	0.052631579
chr15	24966147	24966184	3605	0.052631579
chr15	25439933	25439970	3606	0.052631579
chr15	25877542	25877579	3607	0.052631579
chr15	26064977	26065014	3608	0.052631579
chr15	26184619	26184656	3609	0.052631579
chr15	27666461	27666498	3610	0.052631579
chr15	27788204	27788241	3611	0.052631579
chr15	27885754	27885791	3612	0.052631579
chr15	28043464	28043501	3613	0.052631579
chr15	28047267	28047304	3614	0.052631579
chr15	28047648	28047685	3615	0.052631579
chr15	28731316	28731353	3616	0.052631579
chr15	28747749	28747786	3617	0.052631579
chr15	28791920	28791957	3618	0.052631579
chr15	28866774	28866811	3619	0.052631579
chr15	29157842	29157879	3620	0.052631579
chr15	29432296	29432333	3621	0.052631579
chr15	29433026	29433063	3622	0.052631579
chr15	29451627	29451664	3623	0.052631579
chr15	30110657	30110694	3624	0.052631579
chr15	30382011	30382048	3625	0.052631579
chr15	30392510	30392547	3626	0.052631579
chr15	30940862	30940899	3627	0.052631579
chr15	30941636	30941673	3628	0.052631579
chr15	31293901	31293938	3629	0.052631579
chr15	31315759	31315796	3630	0.052631579
chr15	31928149	31928186	3631	0.052631579
chr15	31978349	31978386	3632	0.052631579
chr15	32427807	32427844	3633	0.052631579
chr15	32431237	32431274	3634	0.052631579
chr15	32801494	32801531	3635	0.052631579
chr15	32837703	32837740	3636	0.052631579
chr15	33896006	33896043	3637	0.052631579
chr15	34679540	34679577	3638	0.052631579
chr15	34754534	34754571	3639	0.052631579
chr15	34865065	34865102	3640	0.052631579
chr15	34957482	34957519	3641	0.052631579
chr15	35052690	35052727	3642	0.052631579
chr15	35226916	35226953	3643	0.052631579
chr15	35425871	35425908	3644	0.052631579
chr15	35464425	35464462	3645	0.052631579
chr15	35711338	35711375	3646	0.052631579
chr15	35959625	35959662	3647	0.052631579
chr15	36012268	36012305	3648	0.052631579
chr15	36897506	36897543	3649	0.052631579
chr15	37041243	37041280	3650	0.052631579

chr15	37118792	37118829	3651	0.052631579
chr15	37236511	37236548	3652	0.052631579
chr15	37729272	37729309	3653	0.052631579
chr15	38089173	38089210	3654	0.052631579
chr15	38178809	38178846	3655	0.052631579
chr15	38191268	38191305	3656	0.052631579
chr15	38373890	38373927	3657	0.052631579
chr15	38444744	38444781	3658	0.052631579
chr15	38731483	38731520	3659	0.052631579
chr15	38865380	38865417	3660	0.052631579
chr15	39240545	39240582	3661	0.052631579
chr15	39902181	39902218	3662	0.052631579
chr15	39942994	39943031	3663	0.052631579
chr15	41233837	41233874	3664	0.052631579
chr15	41825911	41825948	3665	0.052631579
chr15	41897395	41897432	3666	0.052631579
chr15	41953880	41953917	3667	0.052631579
chr15	42577564	42577601	3668	0.052631579
chr15	42650610	42650647	3669	0.052631579
chr15	42923709	42923746	3670	0.052631579
chr15	43261432	43261469	3671	0.052631579
chr15	44076646	44076683	3672	0.052631579
chr15	44124666	44124703	3673	0.052631579
chr15	45079642	45079679	3674	0.052631579
chr15	45294718	45294755	3675	0.052631579
chr15	46567866	46567903	3676	0.052631579
chr15	46601077	46601114	3677	0.052631579
chr15	46616900	46616937	3678	0.052631579
chr15	47445623	47445660	3679	0.052631579
chr15	47494474	47494511	3680	0.052631579
chr15	47766235	47766272	3681	0.052631579
chr15	47943601	47943638	3682	0.052631579
chr15	48074960	48074997	3683	0.052631579
chr15	49160903	49160940	3684	0.052631579
chr15	50273241	50273278	3685	0.052631579
chr15	50442211	50442248	3686	0.052631579
chr15	50444159	50444196	3687	0.052631579
chr15	50545970	50546007	3688	0.052631579
chr15	50867036	50867073	3689	0.052631579
chr15	50949231	50949268	3690	0.052631579
chr15	51253405	51253442	3691	0.052631579
chr15	51253547	51253584	3692	0.052631579
chr15	51418883	51418920	3693	0.052631579
chr15	51521778	51521815	3694	0.052631579
chr15	51629912	51629949	3695	0.052631579
chr15	52004805	52004842	3696	0.052631579
chr15	52067460	52067497	3697	0.052631579
chr15	52068213	52068250	3698	0.052631579
chr15	52400016	52400053	3699	0.052631579
chr15	52568371	52568408	3700	0.052631579

chr15	52580318	52580355	3701	0.052631579
chr15	52649308	52649345	3702	0.052631579
chr15	53035115	53035152	3703	0.052631579
chr15	53450686	53450723	3704	0.052631579
chr15	54834837	54834874	3705	0.052631579
chr15	55298888	55298925	3706	0.052631579
chr15	55411506	55411543	3707	0.052631579
chr15	55715902	55715939	3708	0.052631579
chr15	55782992	55783029	3709	0.052631579
chr15	56344445	56344482	3710	0.052631579
chr15	56500170	56500207	3711	0.052631579
chr15	56500898	56500935	3712	0.052631579
chr15	56800654	56800691	3713	0.052631579
chr15	57274575	57274612	3714	0.052631579
chr15	57722185	57722222	3715	0.052631579
chr15	57984896	57984933	3716	0.052631579
chr15	58012124	58012161	3717	0.052631579
chr15	58085238	58085275	3718	0.052631579
chr15	58304149	58304186	3719	0.052631579
chr15	58754217	58754254	3720	0.052631579
chr15	58776234	58776271	3721	0.052631579
chr15	58776820	58776857	3722	0.052631579
chr15	58877810	58877847	3723	0.052631579
chr15	59107203	59107240	3724	0.052631579
chr15	59588953	59588990	3725	0.052631579
chr15	59735748	59735785	3726	0.052631579
chr15	59780203	59780240	3727	0.052631579
chr15	60215380	60215417	3728	0.052631579
chr15	60608943	60608980	3729	0.052631579
chr15	61036645	61036682	3730	0.052631579
chr15	61359884	61359921	3731	0.052631579
chr15	61494284	61494321	3732	0.052631579
chr15	61573424	61573461	3733	0.052631579
chr15	61633271	61633308	3734	0.052631579
chr15	61714000	61714037	3735	0.052631579
chr15	62540413	62540450	3736	0.052631579
chr15	62567345	62567382	3737	0.052631579
chr15	62587333	62587370	3738	0.052631579
chr15	62757629	62757666	3739	0.052631579
chr15	63307903	63307940	3740	0.052631579
chr15	63393734	63393771	3741	0.052631579
chr15	63519028	63519065	3742	0.052631579
chr15	64556198	64556235	3743	0.052631579
chr15	65221588	65221625	3744	0.052631579
chr15	65627501	65627538	3745	0.052631579
chr15	65658687	65658724	3746	0.052631579
chr15	65720863	65720900	3747	0.052631579
chr15	65745880	65745917	3748	0.052631579
chr15	65864951	65864988	3749	0.052631579
chr15	66164192	66164229	3750	0.052631579

chr15	66378607	66378644	3751	0.052631579
chr15	67022975	67023012	3752	0.052631579
chr15	67592702	67592739	3753	0.052631579
chr15	68155524	68155561	3754	0.052631579
chr15	69089355	69089392	3755	0.052631579
chr15	69738130	69738167	3756	0.052631579
chr15	70242449	70242486	3757	0.052631579
chr15	70363922	70363959	3758	0.052631579
chr15	70638317	70638354	3759	0.052631579
chr15	70648266	70648303	3760	0.052631579
chr15	70829702	70829739	3761	0.052631579
chr15	70830086	70830123	3762	0.052631579
chr15	71356755	71356792	3763	0.052631579
chr15	71409138	71409175	3764	0.052631579
chr15	71643812	71643849	3765	0.052631579
chr15	72398931	72398968	3766	0.052631579
chr15	72448474	72448511	3767	0.052631579
chr15	72714645	72714682	3768	0.052631579
chr15	72772129	72772166	3769	0.052631579
chr15	73045054	73045091	3770	0.052631579
chr15	73686748	73686785	3771	0.052631579
chr15	74211106	74211143	3772	0.052631579
chr15	74417270	74417307	3773	0.052631579
chr15	75096431	75096468	3774	0.052631579
chr15	75848017	75848054	3775	0.052631579
chr15	75876403	75876440	3776	0.052631579
chr15	75877059	75877096	3777	0.052631579
chr15	76188631	76188668	3778	0.052631579
chr15	76888482	76888519	3779	0.052631579
chr15	76894488	76894525	3780	0.052631579
chr15	76991132	76991169	3781	0.052631579
chr15	76991910	76991947	3782	0.052631579
chr15	77378650	77378687	3783	0.052631579
chr15	77401615	77401652	3784	0.052631579
chr15	78395972	78396009	3785	0.052631579
chr15	79047327	79047364	3786	0.052631579
chr15	79058817	79058854	3787	0.052631579
chr15	79253806	79253843	3788	0.052631579
chr15	81019357	81019394	3789	0.052631579
chr15	81019693	81019730	3790	0.052631579
chr15	81351387	81351424	3791	0.052631579
chr15	81478744	81478781	3792	0.052631579
chr15	83327230	83327267	3793	0.052631579
chr15	83726294	83726331	3794	0.052631579
chr15	84221116	84221153	3795	0.052631579
chr15	84483159	84483196	3796	0.052631579
chr15	85152687	85152724	3797	0.052631579
chr15	85428559	85428596	3798	0.052631579
chr15	85843037	85843074	3799	0.052631579
chr15	86336625	86336662	3800	0.052631579

chr15	86372773	86372810	3801	0.052631579
chr15	86605083	86605120	3802	0.052631579
chr15	86843275	86843312	3803	0.052631579
chr15	87038642	87038679	3804	0.052631579
chr15	87249727	87249764	3805	0.052631579
chr15	87566314	87566351	3806	0.052631579
chr15	87714800	87714837	3807	0.052631579
chr15	87715581	87715618	3808	0.052631579
chr15	87734199	87734236	3809	0.052631579
chr15	87756525	87756562	3810	0.052631579
chr15	88143946	88143983	3811	0.052631579
chr15	88776724	88776761	3812	0.052631579
chr15	89158812	89158849	3813	0.052631579
chr15	89159470	89159507	3814	0.052631579
chr15	89293561	89293598	3815	0.052631579
chr15	89686063	89686100	3816	0.052631579
chr15	90420960	90420997	3817	0.052631579
chr15	90746976	90747013	3818	0.052631579
chr15	90881549	90881586	3819	0.052631579
chr15	91070283	91070320	3820	0.052631579
chr15	91141541	91141578	3821	0.052631579
chr15	91311724	91311761	3822	0.052631579
chr15	91380980	91381017	3823	0.052631579
chr15	91434213	91434250	3824	0.052631579
chr15	91839677	91839714	3825	0.052631579
chr15	92117650	92117687	3826	0.052631579
chr15	92397331	92397368	3827	0.052631579
chr15	92446539	92446576	3828	0.052631579
chr15	92446743	92446780	3829	0.052631579
chr15	92467899	92467936	3830	0.052631579
chr15	92676096	92676133	3831	0.052631579
chr15	93620168	93620205	3832	0.052631579
chr15	94349207	94349244	3833	0.052631579
chr15	94485413	94485450	3834	0.052631579
chr15	94500158	94500195	3835	0.052631579
chr15	94676463	94676500	3836	0.052631579
chr15	94676702	94676739	3837	0.052631579
chr15	94881470	94881507	3838	0.052631579
chr15	94963326	94963363	3839	0.052631579
chr15	95394025	95394062	3840	0.052631579
chr15	95920655	95920692	3841	0.052631579
chr15	96713985	96714022	3842	0.052631579
chr15	97011463	97011500	3843	0.052631579
chr15	97149842	97149879	3844	0.052631579
chr15	97191117	97191154	3845	0.052631579
chr15	98086395	98086432	3846	0.052631579
chr15	98401795	98401832	3847	0.052631579
chr15	99008597	99008634	3848	0.052631579
chr15	51151617	51151685	3849	0.057971014
chr15	36748186	36748247	3850	0.064516129

chr15	100135978	100136017	3851	0.075
chr15	19884593	19884632	3852	0.075
chr15	19952976	19953015	3853	0.075
chr15	20585329	20585368	3854	0.075
chr15	23641770	23641809	3855	0.075
chr15	24136824	24136863	3856	0.075
chr15	24229301	24229340	3857	0.075
chr15	24741481	24741520	3858	0.075
chr15	24882643	24882682	3859	0.075
chr15	25076862	25076901	3860	0.075
chr15	25081922	25081961	3861	0.075
chr15	27133668	27133707	3862	0.075
chr15	27186136	27186175	3863	0.075
chr15	29517485	29517524	3864	0.075
chr15	30199523	30199562	3865	0.075
chr15	31284171	31284210	3866	0.075
chr15	32453174	32453213	3867	0.075
chr15	35140520	35140559	3868	0.075
chr15	37417283	37417322	3869	0.075
chr15	37672574	37672613	3870	0.075
chr15	39500025	39500064	3871	0.075
chr15	39641501	39641540	3872	0.075
chr15	39677411	39677450	3873	0.075
chr15	40741902	40741941	3874	0.075
chr15	41398112	41398151	3875	0.075
chr15	42861449	42861488	3876	0.075
chr15	43235350	43235389	3877	0.075
chr15	43840693	43840732	3878	0.075
chr15	45844903	45844942	3879	0.075
chr15	45850899	45850938	3880	0.075
chr15	46529817	46529856	3881	0.075
chr15	46725309	46725348	3882	0.075
chr15	48341147	48341186	3883	0.075
chr15	51547989	51548028	3884	0.075
chr15	52058329	52058368	3885	0.075
chr15	52315398	52315437	3886	0.075
chr15	53056465	53056504	3887	0.075
chr15	56616532	56616571	3888	0.075
chr15	57185315	57185354	3889	0.075
chr15	57347691	57347730	3890	0.075
chr15	57856672	57856711	3891	0.075
chr15	58590544	58590583	3892	0.075
chr15	59005459	59005498	3893	0.075
chr15	59889016	59889055	3894	0.075
chr15	60085553	60085592	3895	0.075
chr15	61241247	61241286	3896	0.075
chr15	61397534	61397573	3897	0.075
chr15	62754617	62754656	3898	0.075
chr15	62946398	62946437	3899	0.075
chr15	63288240	63288279	3900	0.075

chr15	63465985	63466024	3901	0.075
chr15	63684582	63684621	3902	0.075
chr15	63996787	63996826	3903	0.075
chr15	64179197	64179236	3904	0.075
chr15	64230283	64230322	3905	0.075
chr15	64402908	64402947	3906	0.075
chr15	64791750	64791789	3907	0.075
chr15	66147770	66147809	3908	0.075
chr15	66649418	66649457	3909	0.075
chr15	66731473	66731512	3910	0.075
chr15	66814364	66814403	3911	0.075
chr15	68053278	68053317	3912	0.075
chr15	68159915	68159954	3913	0.075
chr15	68553143	68553182	3914	0.075
chr15	69311651	69311690	3915	0.075
chr15	70246156	70246195	3916	0.075
chr15	70642884	70642923	3917	0.075
chr15	71692183	71692222	3918	0.075
chr15	71712543	71712582	3919	0.075
chr15	72483077	72483116	3920	0.075
chr15	72699123	72699162	3921	0.075
chr15	73547947	73547986	3922	0.075
chr15	73955579	73955618	3923	0.075
chr15	74011683	74011722	3924	0.075
chr15	74192018	74192057	3925	0.075
chr15	75500612	75500651	3926	0.075
chr15	77124280	77124319	3927	0.075
chr15	77294158	77294197	3928	0.075
chr15	77995215	77995254	3929	0.075
chr15	78525065	78525104	3930	0.075
chr15	78692183	78692222	3931	0.075
chr15	78715193	78715232	3932	0.075
chr15	79346131	79346170	3933	0.075
chr15	79363058	79363097	3934	0.075
chr15	81732840	81732879	3935	0.075
chr15	84112554	84112593	3936	0.075
chr15	84537799	84537838	3937	0.075
chr15	85160580	85160619	3938	0.075
chr15	85778761	85778800	3939	0.075
chr15	85848379	85848418	3940	0.075
chr15	87544243	87544282	3941	0.075
chr15	87664121	87664160	3942	0.075
chr15	87978092	87978131	3943	0.075
chr15	88047730	88047769	3944	0.075
chr15	88121393	88121432	3945	0.075
chr15	88425664	88425703	3946	0.075
chr15	88659947	88659986	3947	0.075
chr15	89180757	89180796	3948	0.075
chr15	89587885	89587924	3949	0.075
chr15	91463644	91463683	3950	0.075

chr15	91544905	91544944	3951	0.075
chr15	91672245	91672284	3952	0.075
chr15	92839469	92839508	3953	0.075
chr15	95436363	95436402	3954	0.075
chr15	95526719	95526758	3955	0.075
chr15	97105424	97105463	3956	0.075
chr15	97482774	97482813	3957	0.075
chr15	98140269	98140308	3958	0.075
chr15	98156505	98156544	3959	0.075
chr15	98406164	98406203	3960	0.075
chr15	98542597	98542636	3961	0.075
chr15	98559845	98559884	3962	0.075
chr15	98579172	98579211	3963	0.075
chr15	99386829	99386868	3964	0.075
chr15	99446741	99446780	3965	0.075
chr15	99533696	99533735	3966	0.075
chr15	99683178	99683217	3967	0.075
chr15	58552742	58552780	3968	0.076923077
chr15	98156405	98156443	3969	0.076923077
chr15	62239375	62239416	3970	0.095238095
chr15	22677871	22677910	3971	0.1
chr16	30843001	30843114	3972	0.035087719
chr16	14349256	14349367	3973	0.035714286
chr16	17424467	17424575	3974	0.036697248
chr16	65586971	65587067	3975	0.041237113
chr16	47991084	47991164	3976	0.049382716
chr16	83892317	83892396	3977	0.05
chr16	87995	88034	3978	0.05
chr16	10207637	10207674	3979	0.052631579
chr16	10597457	10597494	3980	0.052631579
chr16	11304442	11304479	3981	0.052631579
chr16	11314211	11314248	3982	0.052631579
chr16	11382375	11382412	3983	0.052631579
chr16	11388778	11388815	3984	0.052631579
chr16	11389591	11389628	3985	0.052631579
chr16	11753128	11753165	3986	0.052631579
chr16	12520662	12520699	3987	0.052631579
chr16	12685714	12685751	3988	0.052631579
chr16	12740209	12740246	3989	0.052631579
chr16	13603701	13603738	3990	0.052631579
chr16	14264057	14264094	3991	0.052631579
chr16	15010421	15010458	3992	0.052631579
chr16	15047447	15047484	3993	0.052631579
chr16	15716327	15716364	3994	0.052631579
chr16	1666295	1666332	3995	0.052631579
chr16	16792838	16792875	3996	0.052631579
chr16	17092539	17092576	3997	0.052631579
chr16	17226993	17227030	3998	0.052631579
chr16	17293829	17293866	3999	0.052631579
chr16	17498612	17498649	4000	0.052631579

chr16	18304853	18304890	4001	0.052631579
chr16	18819786	18819823	4002	0.052631579
chr16	19102876	19102913	4003	0.052631579
chr16	19339871	19339908	4004	0.052631579
chr16	19806083	19806120	4005	0.052631579
chr16	19882076	19882113	4006	0.052631579
chr16	20153685	20153722	4007	0.052631579
chr16	2038370	2038407	4008	0.052631579
chr16	20592484	20592521	4009	0.052631579
chr16	20789314	20789351	4010	0.052631579
chr16	20916214	20916251	4011	0.052631579
chr16	21119643	21119680	4012	0.052631579
chr16	21887463	21887500	4013	0.052631579
chr16	2196158	2196195	4014	0.052631579
chr16	22138550	22138587	4015	0.052631579
chr16	23369168	23369205	4016	0.052631579
chr16	24744500	24744537	4017	0.052631579
chr16	24892249	24892286	4018	0.052631579
chr16	2502249	2502286	4019	0.052631579
chr16	25505390	25505427	4020	0.052631579
chr16	25506183	25506220	4021	0.052631579
chr16	25563439	25563476	4022	0.052631579
chr16	26445061	26445098	4023	0.052631579
chr16	2692404	2692441	4024	0.052631579
chr16	27462597	27462634	4025	0.052631579
chr16	27509655	27509692	4026	0.052631579
chr16	27705667	27705704	4027	0.052631579
chr16	27902630	27902667	4028	0.052631579
chr16	28023565	28023602	4029	0.052631579
chr16	28025209	28025246	4030	0.052631579
chr16	28159437	28159474	4031	0.052631579
chr16	28802102	28802139	4032	0.052631579
chr16	29752564	29752601	4033	0.052631579
chr16	29781461	29781498	4034	0.052631579
chr16	29901632	29901669	4035	0.052631579
chr16	29982076	29982113	4036	0.052631579
chr16	30035794	30035831	4037	0.052631579
chr16	30697767	30697804	4038	0.052631579
chr16	30698617	30698654	4039	0.052631579
chr16	30861027	30861064	4040	0.052631579
chr16	30875877	30875914	4041	0.052631579
chr16	30880267	30880304	4042	0.052631579
chr16	31281861	31281898	4043	0.052631579
chr16	31326318	31326355	4044	0.052631579
chr16	32298006	32298043	4045	0.052631579
chr16	3280374	3280411	4046	0.052631579
chr16	33287345	33287382	4047	0.052631579
chr16	33485860	33485897	4048	0.052631579
chr16	3354565	3354602	4049	0.052631579
chr16	33771505	33771542	4050	0.052631579

chr16	34063507	34063544	4051	0.052631579
chr16	34222977	34223014	4052	0.052631579
chr16	34547341	34547378	4053	0.052631579
chr16	3644351	3644388	4054	0.052631579
chr16	3719628	3719665	4055	0.052631579
chr16	3768855	3768892	4056	0.052631579
chr16	3775773	3775810	4057	0.052631579
chr16	3956477	3956514	4058	0.052631579
chr16	3996501	3996538	4059	0.052631579
chr16	4518403	4518440	4060	0.052631579
chr16	45640186	45640223	4061	0.052631579
chr16	45728241	45728278	4062	0.052631579
chr16	46044524	46044561	4063	0.052631579
chr16	46049056	46049093	4064	0.052631579
chr16	46296499	46296536	4065	0.052631579
chr16	46688744	46688781	4066	0.052631579
chr16	46953369	46953406	4067	0.052631579
chr16	47488254	47488291	4068	0.052631579
chr16	47615133	47615170	4069	0.052631579
chr16	47753995	47754032	4070	0.052631579
chr16	47875860	47875897	4071	0.052631579
chr16	47884947	47884984	4072	0.052631579
chr16	48293276	48293313	4073	0.052631579
chr16	4847417	4847454	4074	0.052631579
chr16	48485580	48485617	4075	0.052631579
chr16	48586163	48586200	4076	0.052631579
chr16	48604770	48604807	4077	0.052631579
chr16	48646696	48646733	4078	0.052631579
chr16	48650038	48650075	4079	0.052631579
chr16	49178341	49178378	4080	0.052631579
chr16	49369260	49369297	4081	0.052631579
chr16	49660369	49660406	4082	0.052631579
chr16	49774528	49774565	4083	0.052631579
chr16	50620275	50620312	4084	0.052631579
chr16	50680778	50680815	4085	0.052631579
chr16	50717716	50717753	4086	0.052631579
chr16	50718517	50718554	4087	0.052631579
chr16	51133017	51133054	4088	0.052631579
chr16	51136494	51136531	4089	0.052631579
chr16	52196964	52197001	4090	0.052631579
chr16	52253320	52253357	4091	0.052631579
chr16	52280305	52280342	4092	0.052631579
chr16	52530692	52530729	4093	0.052631579
chr16	52677531	52677568	4094	0.052631579
chr16	53458053	53458090	4095	0.052631579
chr16	53677448	53677485	4096	0.052631579
chr16	54587563	54587600	4097	0.052631579
chr16	54599919	54599956	4098	0.052631579
chr16	54603506	54603543	4099	0.052631579
chr16	54713600	54713637	4100	0.052631579

chr16	54804565	54804602	4101	0.052631579
chr16	55006447	55006484	4102	0.052631579
chr16	55349787	55349824	4103	0.052631579
chr16	55665822	55665859	4104	0.052631579
chr16	5599026	5599063	4105	0.052631579
chr16	56245591	56245628	4106	0.052631579
chr16	56271813	56271850	4107	0.052631579
chr16	56324955	56324992	4108	0.052631579
chr16	57100383	57100420	4109	0.052631579
chr16	57143031	57143068	4110	0.052631579
chr16	572409	572446	4111	0.052631579
chr16	57624190	57624227	4112	0.052631579
chr16	57781669	57781706	4113	0.052631579
chr16	5810636	5810673	4114	0.052631579
chr16	59379127	59379164	4115	0.052631579
chr16	59434204	59434241	4116	0.052631579
chr16	5955695	5955732	4117	0.052631579
chr16	60277599	60277636	4118	0.052631579
chr16	60526205	60526242	4119	0.052631579
chr16	60723092	60723129	4120	0.052631579
chr16	61755336	61755373	4121	0.052631579
chr16	61755413	61755450	4122	0.052631579
chr16	62147104	62147141	4123	0.052631579
chr16	62216601	62216638	4124	0.052631579
chr16	62516652	62516689	4125	0.052631579
chr16	63277029	63277066	4126	0.052631579
chr16	6342873	6342910	4127	0.052631579
chr16	63440574	63440611	4128	0.052631579
chr16	63444271	63444308	4129	0.052631579
chr16	63590030	63590067	4130	0.052631579
chr16	6395194	6395231	4131	0.052631579
chr16	64465920	64465957	4132	0.052631579
chr16	64574777	64574814	4133	0.052631579
chr16	6488498	6488535	4134	0.052631579
chr16	6512285	6512322	4135	0.052631579
chr16	65243530	65243567	4136	0.052631579
chr16	65525520	65525557	4137	0.052631579
chr16	65877878	65877915	4138	0.052631579
chr16	65997819	65997856	4139	0.052631579
chr16	66047329	66047366	4140	0.052631579
chr16	66140838	66140875	4141	0.052631579
chr16	66236929	66236966	4142	0.052631579
chr16	66709525	66709562	4143	0.052631579
chr16	66775666	66775703	4144	0.052631579
chr16	66947560	66947597	4145	0.052631579
chr16	67369985	67370022	4146	0.052631579
chr16	67553012	67553049	4147	0.052631579
chr16	67705814	67705851	4148	0.052631579
chr16	67828711	67828748	4149	0.052631579
chr16	67943691	67943728	4150	0.052631579

chr16	68528998	68529035	4151	0.052631579
chr16	6882196	6882233	4152	0.052631579
chr16	68921353	68921390	4153	0.052631579
chr16	69159743	69159780	4154	0.052631579
chr16	69255206	69255243	4155	0.052631579
chr16	69305023	69305060	4156	0.052631579
chr16	69425247	69425284	4157	0.052631579
chr16	69877982	69878019	4158	0.052631579
chr16	69895052	69895089	4159	0.052631579
chr16	70475105	70475142	4160	0.052631579
chr16	70724341	70724378	4161	0.052631579
chr16	70761737	70761774	4162	0.052631579
chr16	7094213	7094250	4163	0.052631579
chr16	71681158	71681195	4164	0.052631579
chr16	71775634	71775671	4165	0.052631579
chr16	72335559	72335596	4166	0.052631579
chr16	72341183	72341220	4167	0.052631579
chr16	72341744	72341781	4168	0.052631579
chr16	7248566	7248603	4169	0.052631579
chr16	72568306	72568343	4170	0.052631579
chr16	72767640	72767677	4171	0.052631579
chr16	73071701	73071738	4172	0.052631579
chr16	73328060	73328097	4173	0.052631579
chr16	73834337	73834374	4174	0.052631579
chr16	7385412	7385449	4175	0.052631579
chr16	74111610	74111647	4176	0.052631579
chr16	74225611	74225648	4177	0.052631579
chr16	74364156	74364193	4178	0.052631579
chr16	74550597	74550634	4179	0.052631579
chr16	75625961	75625998	4180	0.052631579
chr16	76581236	76581273	4181	0.052631579
chr16	76878863	76878900	4182	0.052631579
chr16	76920945	76920982	4183	0.052631579
chr16	77053456	77053493	4184	0.052631579
chr16	77068835	77068872	4185	0.052631579
chr16	77165171	77165208	4186	0.052631579
chr16	77844650	77844687	4187	0.052631579
chr16	78530415	78530452	4188	0.052631579
chr16	78981454	78981491	4189	0.052631579
chr16	79050340	79050377	4190	0.052631579
chr16	7912270	7912307	4191	0.052631579
chr16	79300428	79300465	4192	0.052631579
chr16	79301187	79301224	4193	0.052631579
chr16	79426927	79426964	4194	0.052631579
chr16	80034747	80034784	4195	0.052631579
chr16	80263121	80263158	4196	0.052631579
chr16	80674410	80674447	4197	0.052631579
chr16	80917616	80917653	4198	0.052631579
chr16	80955109	80955146	4199	0.052631579
chr16	81265030	81265067	4200	0.052631579

chr16	81725230	81725267	4201	0.052631579
chr16	81819841	81819878	4202	0.052631579
chr16	81833671	81833708	4203	0.052631579
chr16	82023802	82023839	4204	0.052631579
chr16	82083942	82083979	4205	0.052631579
chr16	82345233	82345270	4206	0.052631579
chr16	82981594	82981631	4207	0.052631579
chr16	83581503	83581540	4208	0.052631579
chr16	83830790	83830827	4209	0.052631579
chr16	8409404	8409441	4210	0.052631579
chr16	84168959	84168996	4211	0.052631579
chr16	84363349	84363386	4212	0.052631579
chr16	84708649	84708686	4213	0.052631579
chr16	85047176	85047213	4214	0.052631579
chr16	86548171	86548208	4215	0.052631579
chr16	86635462	86635499	4216	0.052631579
chr16	87050187	87050224	4217	0.052631579
chr16	87292900	87292937	4218	0.052631579
chr16	87313492	87313529	4219	0.052631579
chr16	87572132	87572169	4220	0.052631579
chr16	87875449	87875486	4221	0.052631579
chr16	88072660	88072697	4222	0.052631579
chr16	88182612	88182649	4223	0.052631579
chr16	9461718	9461755	4224	0.052631579
chr16	45553616	45553707	4225	0.054347826
chr16	30342830	30342895	4226	0.060606061
chr16	57190696	57190750	4227	0.072727273
chr16	10962966	10963005	4228	0.075
chr16	11051037	11051076	4229	0.075
chr16	11206988	11207027	4230	0.075
chr16	12035664	12035703	4231	0.075
chr16	12126226	12126265	4232	0.075
chr16	14302511	14302550	4233	0.075
chr16	14582725	14582764	4234	0.075
chr16	1556298	1556337	4235	0.075
chr16	15978673	15978712	4236	0.075
chr16	1680757	1680796	4237	0.075
chr16	16920668	16920707	4238	0.075
chr16	16939142	16939181	4239	0.075
chr16	1705355	1705394	4240	0.075
chr16	17163115	17163154	4241	0.075
chr16	17248576	17248615	4242	0.075
chr16	17523880	17523919	4243	0.075
chr16	1763350	1763389	4244	0.075
chr16	17927906	17927945	4245	0.075
chr16	18734213	18734252	4246	0.075
chr16	18748503	18748542	4247	0.075
chr16	1954559	1954598	4248	0.075
chr16	2005845	2005884	4249	0.075
chr16	20534452	20534491	4250	0.075

chr16	20804734	20804773	4251	0.075
chr16	21617684	21617723	4252	0.075
chr16	2168574	2168613	4253	0.075
chr16	218293	218332	4254	0.075
chr16	21974203	21974242	4255	0.075
chr16	22539710	22539749	4256	0.075
chr16	22855153	22855192	4257	0.075
chr16	23325344	23325383	4258	0.075
chr16	24459163	24459202	4259	0.075
chr16	27502239	27502278	4260	0.075
chr16	2956180	2956219	4261	0.075
chr16	2977395	2977434	4262	0.075
chr16	29974052	29974091	4263	0.075
chr16	29986284	29986323	4264	0.075
chr16	30319650	30319689	4265	0.075
chr16	30523616	30523655	4266	0.075
chr16	31082675	31082714	4267	0.075
chr16	31381022	31381061	4268	0.075
chr16	32515448	32515487	4269	0.075
chr16	34299627	34299666	4270	0.075
chr16	3579593	3579632	4271	0.075
chr16	3633478	3633517	4272	0.075
chr16	3731231	3731270	4273	0.075
chr16	4403266	4403305	4274	0.075
chr16	45320491	45320530	4275	0.075
chr16	4596572	4596611	4276	0.075
chr16	46595626	46595665	4277	0.075
chr16	4684555	4684594	4278	0.075
chr16	47325721	47325760	4279	0.075
chr16	47609205	47609244	4280	0.075
chr16	47728960	47728999	4281	0.075
chr16	4786767	4786806	4282	0.075
chr16	48030094	48030133	4283	0.075
chr16	48691149	48691188	4284	0.075
chr16	49057764	49057803	4285	0.075
chr16	4908897	4908936	4286	0.075
chr16	49293758	49293797	4287	0.075
chr16	49614466	49614505	4288	0.075
chr16	5010928	5010967	4289	0.075
chr16	50302352	50302391	4290	0.075
chr16	50606028	50606067	4291	0.075
chr16	51035799	51035838	4292	0.075
chr16	5247805	5247844	4293	0.075
chr16	52713778	52713817	4294	0.075
chr16	52791916	52791955	4295	0.075
chr16	53177032	53177071	4296	0.075
chr16	53363913	53363952	4297	0.075
chr16	53745624	53745663	4298	0.075
chr16	54089814	54089853	4299	0.075
chr16	54506486	54506525	4300	0.075

chr16	55077031	55077070	4301	0.075
chr16	55139144	55139183	4302	0.075
chr16	55161483	55161522	4303	0.075
chr16	55200419	55200458	4304	0.075
chr16	55430420	55430459	4305	0.075
chr16	56361996	56362035	4306	0.075
chr16	5667224	5667263	4307	0.075
chr16	56785821	56785860	4308	0.075
chr16	576445	576484	4309	0.075
chr16	58508012	58508051	4310	0.075
chr16	59654466	59654505	4311	0.075
chr16	5988488	5988527	4312	0.075
chr16	60245027	60245066	4313	0.075
chr16	6103642	6103681	4314	0.075
chr16	6123276	6123315	4315	0.075
chr16	62156088	62156127	4316	0.075
chr16	63282711	63282750	4317	0.075
chr16	64884464	64884503	4318	0.075
chr16	64994833	64994872	4319	0.075
chr16	65251834	65251873	4320	0.075
chr16	65360955	65360994	4321	0.075
chr16	65731448	65731487	4322	0.075
chr16	66717926	66717965	4323	0.075
chr16	66880766	66880805	4324	0.075
chr16	66902292	66902331	4325	0.075
chr16	67407181	67407220	4326	0.075
chr16	67907641	67907680	4327	0.075
chr16	68193555	68193594	4328	0.075
chr16	68284666	68284705	4329	0.075
chr16	68309875	68309914	4330	0.075
chr16	69115521	69115560	4331	0.075
chr16	69286302	69286341	4332	0.075
chr16	69576068	69576107	4333	0.075
chr16	69964895	69964934	4334	0.075
chr16	70219336	70219375	4335	0.075
chr16	70687660	70687699	4336	0.075
chr16	71393479	71393518	4337	0.075
chr16	71542048	71542087	4338	0.075
chr16	71951182	71951221	4339	0.075
chr16	72390050	72390089	4340	0.075
chr16	72508618	72508657	4341	0.075
chr16	72846803	72846842	4342	0.075
chr16	72856464	72856503	4343	0.075
chr16	73043646	73043685	4344	0.075
chr16	73077211	73077250	4345	0.075
chr16	73259492	73259531	4346	0.075
chr16	736159	736198	4347	0.075
chr16	75174885	75174924	4348	0.075
chr16	76749725	76749764	4349	0.075
chr16	77878978	77879017	4350	0.075

chr16	77894049	77894088	4351	0.075
chr16	78248139	78248178	4352	0.075
chr16	79094977	79095016	4353	0.075
chr16	79204161	79204200	4354	0.075
chr16	79253609	79253648	4355	0.075
chr16	80291774	80291813	4356	0.075
chr16	80739908	80739947	4357	0.075
chr16	80977158	80977197	4358	0.075
chr16	81225899	81225938	4359	0.075
chr16	81422390	81422429	4360	0.075
chr16	81728835	81728874	4361	0.075
chr16	81911701	81911740	4362	0.075
chr16	81993189	81993228	4363	0.075
chr16	82629644	82629683	4364	0.075
chr16	83481614	83481653	4365	0.075
chr16	83532555	83532594	4366	0.075
chr16	84390773	84390812	4367	0.075
chr16	85651625	85651664	4368	0.075
chr16	85842612	85842651	4369	0.075
chr16	86026534	86026573	4370	0.075
chr16	86338013	86338052	4371	0.075
chr16	86662564	86662603	4372	0.075
chr16	86684009	86684048	4373	0.075
chr16	86738918	86738957	4374	0.075
chr16	86831387	86831426	4375	0.075
chr16	8714319	8714358	4376	0.075
chr16	87268855	87268894	4377	0.075
chr16	87495274	87495313	4378	0.075
chr16	88239147	88239186	4379	0.075
chr16	88529106	88529145	4380	0.075
chr16	57990655	57990704	4381	0.08
chr16	63601358	63601415	4382	0.086206897
chr17	45618174	45618271	4383	0.040816327
chr17	84711	84750	4384	0.05
chr17	7581284	7581360	4385	0.051948052
chr17	10071209	10071246	4386	0.052631579
chr17	10150993	10151030	4387	0.052631579
chr17	10244492	10244529	4388	0.052631579
chr17	10474470	10474507	4389	0.052631579
chr17	115367	115404	4390	0.052631579
chr17	11570110	11570147	4391	0.052631579
chr17	12251020	12251057	4392	0.052631579
chr17	12639338	12639375	4393	0.052631579
chr17	13268608	13268645	4394	0.052631579
chr17	13497535	13497572	4395	0.052631579
chr17	13569765	13569802	4396	0.052631579
chr17	14146164	14146201	4397	0.052631579
chr17	14505871	14505908	4398	0.052631579
chr17	14683424	14683461	4399	0.052631579
chr17	1483820	1483857	4400	0.052631579

chr17	15073919	15073956	4401	0.052631579
chr17	15869125	15869162	4402	0.052631579
chr17	15990825	15990862	4403	0.052631579
chr17	16155091	16155128	4404	0.052631579
chr17	16161916	16161953	4405	0.052631579
chr17	16292550	16292587	4406	0.052631579
chr17	16814046	16814083	4407	0.052631579
chr17	1734495	1734532	4408	0.052631579
chr17	17650630	17650667	4409	0.052631579
chr17	17855699	17855736	4410	0.052631579
chr17	1787606	1787643	4411	0.052631579
chr17	17909408	17909445	4412	0.052631579
chr17	1816345	1816382	4413	0.052631579
chr17	1846157	1846194	4414	0.052631579
chr17	18823666	18823703	4415	0.052631579
chr17	19949490	19949527	4416	0.052631579
chr17	20135139	20135176	4417	0.052631579
chr17	20479985	20480022	4418	0.052631579
chr17	2086687	2086724	4419	0.052631579
chr17	21131656	21131693	4420	0.052631579
chr17	21161817	21161854	4421	0.052631579
chr17	21469512	21469549	4422	0.052631579
chr17	21493523	21493560	4423	0.052631579
chr17	2184857	2184894	4424	0.052631579
chr17	22130840	22130877	4425	0.052631579
chr17	22989158	22989195	4426	0.052631579
chr17	23122019	23122056	4427	0.052631579
chr17	23155333	23155370	4428	0.052631579
chr17	23633769	23633806	4429	0.052631579
chr17	23634539	23634576	4430	0.052631579
chr17	23706964	23707001	4431	0.052631579
chr17	23707346	23707383	4432	0.052631579
chr17	24039305	24039342	4433	0.052631579
chr17	24233348	24233385	4434	0.052631579
chr17	24361806	24361843	4435	0.052631579
chr17	24519845	24519882	4436	0.052631579
chr17	24601433	24601470	4437	0.052631579
chr17	24964333	24964370	4438	0.052631579
chr17	24964881	24964918	4439	0.052631579
chr17	25050687	25050724	4440	0.052631579
chr17	25271605	25271642	4441	0.052631579
chr17	25676588	25676625	4442	0.052631579
chr17	26411056	26411093	4443	0.052631579
chr17	26445439	26445476	4444	0.052631579
chr17	26574584	26574621	4445	0.052631579
chr17	26743082	26743119	4446	0.052631579
chr17	26908677	26908714	4447	0.052631579
chr17	27274789	27274826	4448	0.052631579
chr17	2746595	2746632	4449	0.052631579
chr17	27961384	27961421	4450	0.052631579

chr17	28749556	28749593	4451	0.052631579
chr17	2966695	2966732	4452	0.052631579
chr17	29804527	29804564	4453	0.052631579
chr17	30161289	30161326	4454	0.052631579
chr17	3063518	3063555	4455	0.052631579
chr17	30941628	30941665	4456	0.052631579
chr17	32028751	32028788	4457	0.052631579
chr17	32096586	32096623	4458	0.052631579
chr17	32380968	32381005	4459	0.052631579
chr17	32542616	32542653	4460	0.052631579
chr17	32563198	32563235	4461	0.052631579
chr17	32623153	32623190	4462	0.052631579
chr17	32680685	32680722	4463	0.052631579
chr17	32708373	32708410	4464	0.052631579
chr17	32940137	32940174	4465	0.052631579
chr17	33167738	33167775	4466	0.052631579
chr17	3353610	3353647	4467	0.052631579
chr17	33774752	33774789	4468	0.052631579
chr17	33920749	33920786	4469	0.052631579
chr17	33958305	33958342	4470	0.052631579
chr17	33960976	33961013	4471	0.052631579
chr17	33961758	33961795	4472	0.052631579
chr17	34251407	34251444	4473	0.052631579
chr17	34880761	34880798	4474	0.052631579
chr17	34972430	34972467	4475	0.052631579
chr17	3519371	3519408	4476	0.052631579
chr17	35338918	35338955	4477	0.052631579
chr17	35480538	35480575	4478	0.052631579
chr17	35484228	35484265	4479	0.052631579
chr17	35524315	35524352	4480	0.052631579
chr17	35716990	35717027	4481	0.052631579
chr17	35759564	35759601	4482	0.052631579
chr17	35954201	35954238	4483	0.052631579
chr17	3627649	3627686	4484	0.052631579
chr17	36570139	36570176	4485	0.052631579
chr17	36888711	36888748	4486	0.052631579
chr17	37073516	37073553	4487	0.052631579
chr17	37229348	37229385	4488	0.052631579
chr17	37277424	37277461	4489	0.052631579
chr17	37531021	37531058	4490	0.052631579
chr17	37734948	37734985	4491	0.052631579
chr17	37794346	37794383	4492	0.052631579
chr17	3792737	3792774	4493	0.052631579
chr17	38117854	38117891	4494	0.052631579
chr17	38246310	38246347	4495	0.052631579
chr17	38324961	38324998	4496	0.052631579
chr17	38401055	38401092	4497	0.052631579
chr17	38451328	38451365	4498	0.052631579
chr17	38515209	38515246	4499	0.052631579
chr17	38832738	38832775	4500	0.052631579

chr17	39006692	39006729	4501	0.052631579
chr17	39644857	39644894	4502	0.052631579
chr17	39654209	39654246	4503	0.052631579
chr17	39831052	39831089	4504	0.052631579
chr17	39890084	39890121	4505	0.052631579
chr17	40200377	40200414	4506	0.052631579
chr17	40200565	40200602	4507	0.052631579
chr17	40262957	40262994	4508	0.052631579
chr17	40318306	40318343	4509	0.052631579
chr17	40395752	40395789	4510	0.052631579
chr17	40678781	40678818	4511	0.052631579
chr17	41549913	41549950	4512	0.052631579
chr17	4218294	4218331	4513	0.052631579
chr17	42550219	42550256	4514	0.052631579
chr17	42734884	42734921	4515	0.052631579
chr17	42735803	42735840	4516	0.052631579
chr17	42828927	42828964	4517	0.052631579
chr17	43272559	43272596	4518	0.052631579
chr17	43289791	43289828	4519	0.052631579
chr17	43374134	43374171	4520	0.052631579
chr17	43716324	43716361	4521	0.052631579
chr17	43723890	43723927	4522	0.052631579
chr17	43883551	43883588	4523	0.052631579
chr17	43884083	43884120	4524	0.052631579
chr17	44006199	44006236	4525	0.052631579
chr17	44007333	44007370	4526	0.052631579
chr17	44485991	44486028	4527	0.052631579
chr17	4502877	4502914	4528	0.052631579
chr17	45482932	45482969	4529	0.052631579
chr17	45483714	45483751	4530	0.052631579
chr17	45582404	45582441	4531	0.052631579
chr17	45817707	45817744	4532	0.052631579
chr17	46294993	46295030	4533	0.052631579
chr17	4634432	4634469	4534	0.052631579
chr17	4658332	4658369	4535	0.052631579
chr17	46652326	46652363	4536	0.052631579
chr17	46683954	46683991	4537	0.052631579
chr17	47590279	47590316	4538	0.052631579
chr17	47614387	47614424	4539	0.052631579
chr17	47699211	47699248	4540	0.052631579
chr17	4848958	4848995	4541	0.052631579
chr17	48885217	48885254	4542	0.052631579
chr17	4939893	4939930	4543	0.052631579
chr17	49497928	49497965	4544	0.052631579
chr17	49834228	49834265	4545	0.052631579
chr17	50313801	50313838	4546	0.052631579
chr17	51128512	51128549	4547	0.052631579
chr17	51135775	51135812	4548	0.052631579
chr17	51153021	51153058	4549	0.052631579
chr17	51558520	51558557	4550	0.052631579

chr17	52146854	52146891	4551	0.052631579
chr17	5217381	5217418	4552	0.052631579
chr17	52241414	52241451	4553	0.052631579
chr17	52290105	52290142	4554	0.052631579
chr17	52386202	52386239	4555	0.052631579
chr17	52393142	52393179	4556	0.052631579
chr17	52470872	52470909	4557	0.052631579
chr17	52689527	52689564	4558	0.052631579
chr17	52689698	52689735	4559	0.052631579
chr17	52843718	52843755	4560	0.052631579
chr17	52883465	52883502	4561	0.052631579
chr17	5306463	5306500	4562	0.052631579
chr17	53242386	53242423	4563	0.052631579
chr17	53433414	53433451	4564	0.052631579
chr17	53757902	53757939	4565	0.052631579
chr17	53939618	53939655	4566	0.052631579
chr17	54073398	54073435	4567	0.052631579
chr17	55097002	55097039	4568	0.052631579
chr17	55125947	55125984	4569	0.052631579
chr17	55240584	55240621	4570	0.052631579
chr17	55269942	55269979	4571	0.052631579
chr17	55328719	55328756	4572	0.052631579
chr17	56454104	56454141	4573	0.052631579
chr17	56628670	56628707	4574	0.052631579
chr17	56833912	56833949	4575	0.052631579
chr17	56884266	56884303	4576	0.052631579
chr17	56949925	56949962	4577	0.052631579
chr17	56950454	56950491	4578	0.052631579
chr17	57343687	57343724	4579	0.052631579
chr17	57384331	57384368	4580	0.052631579
chr17	58103110	58103147	4581	0.052631579
chr17	58170990	58171027	4582	0.052631579
chr17	58178470	58178507	4583	0.052631579
chr17	58679082	58679119	4584	0.052631579
chr17	58786291	58786328	4585	0.052631579
chr17	58786458	58786495	4586	0.052631579
chr17	58851712	58851749	4587	0.052631579
chr17	5885531	5885568	4588	0.052631579
chr17	5952327	5952364	4589	0.052631579
chr17	603989	604026	4590	0.052631579
chr17	60467672	60467709	4591	0.052631579
chr17	60598306	60598343	4592	0.052631579
chr17	60620163	60620200	4593	0.052631579
chr17	60637977	60638014	4594	0.052631579
chr17	61496240	61496277	4595	0.052631579
chr17	62140243	62140280	4596	0.052631579
chr17	62274634	62274671	4597	0.052631579
chr17	62533866	62533903	4598	0.052631579
chr17	62850585	62850622	4599	0.052631579
chr17	63328295	63328332	4600	0.052631579

chr17	63328529	63328566	4601	0.052631579
chr17	63341009	63341046	4602	0.052631579
chr17	63374756	63374793	4603	0.052631579
chr17	63587494	63587531	4604	0.052631579
chr17	64784744	64784781	4605	0.052631579
chr17	6483650	6483687	4606	0.052631579
chr17	64915055	64915092	4607	0.052631579
chr17	6494063	6494100	4608	0.052631579
chr17	65505058	65505095	4609	0.052631579
chr17	66007752	66007789	4610	0.052631579
chr17	66079397	66079434	4611	0.052631579
chr17	66371655	66371692	4612	0.052631579
chr17	66715782	66715819	4613	0.052631579
chr17	66733190	66733227	4614	0.052631579
chr17	66847322	66847359	4615	0.052631579
chr17	67055395	67055432	4616	0.052631579
chr17	67129126	67129163	4617	0.052631579
chr17	67633723	67633760	4618	0.052631579
chr17	67768726	67768763	4619	0.052631579
chr17	67844249	67844286	4620	0.052631579
chr17	67979218	67979255	4621	0.052631579
chr17	68270906	68270943	4622	0.052631579
chr17	68342856	68342893	4623	0.052631579
chr17	68755401	68755438	4624	0.052631579
chr17	68865814	68865851	4625	0.052631579
chr17	68901416	68901453	4626	0.052631579
chr17	69023809	69023846	4627	0.052631579
chr17	69293697	69293734	4628	0.052631579
chr17	69323273	69323310	4629	0.052631579
chr17	69448605	69448642	4630	0.052631579
chr17	69514314	69514351	4631	0.052631579
chr17	69792484	69792521	4632	0.052631579
chr17	69949687	69949724	4633	0.052631579
chr17	70257482	70257519	4634	0.052631579
chr17	70595817	70595854	4635	0.052631579
chr17	70723475	70723512	4636	0.052631579
chr17	71003220	71003257	4637	0.052631579
chr17	71013545	71013582	4638	0.052631579
chr17	71039606	71039643	4639	0.052631579
chr17	71102100	71102137	4640	0.052631579
chr17	71149335	71149372	4641	0.052631579
chr17	71192880	71192917	4642	0.052631579
chr17	71292750	71292787	4643	0.052631579
chr17	71293552	71293589	4644	0.052631579
chr17	71352678	71352715	4645	0.052631579
chr17	71395369	71395406	4646	0.052631579
chr17	7155436	7155473	4647	0.052631579
chr17	71647653	71647690	4648	0.052631579
chr17	71669279	71669316	4649	0.052631579
chr17	71819076	71819113	4650	0.052631579

chr17	71919227	71919264	4651	0.052631579
chr17	71970693	71970730	4652	0.052631579
chr17	71971328	71971365	4653	0.052631579
chr17	71980452	71980489	4654	0.052631579
chr17	71982464	71982501	4655	0.052631579
chr17	72051391	72051428	4656	0.052631579
chr17	72242203	72242240	4657	0.052631579
chr17	72423705	72423742	4658	0.052631579
chr17	72477232	72477269	4659	0.052631579
chr17	72899986	72900023	4660	0.052631579
chr17	7292773	7292810	4661	0.052631579
chr17	7311506	7311543	4662	0.052631579
chr17	73398353	73398390	4663	0.052631579
chr17	7341377	7341414	4664	0.052631579
chr17	73455037	73455074	4665	0.052631579
chr17	73646617	73646654	4666	0.052631579
chr17	7402431	7402468	4667	0.052631579
chr17	74129684	74129721	4668	0.052631579
chr17	74177458	74177495	4669	0.052631579
chr17	74178171	74178208	4670	0.052631579
chr17	74206356	74206393	4671	0.052631579
chr17	7430040	7430077	4672	0.052631579
chr17	74382172	74382209	4673	0.052631579
chr17	74721277	74721314	4674	0.052631579
chr17	74765803	74765840	4675	0.052631579
chr17	74766261	74766298	4676	0.052631579
chr17	74973038	74973075	4677	0.052631579
chr17	75400073	75400110	4678	0.052631579
chr17	75423488	75423525	4679	0.052631579
chr17	75647028	75647065	4680	0.052631579
chr17	76058954	76058991	4681	0.052631579
chr17	76114561	76114598	4682	0.052631579
chr17	76325325	76325362	4683	0.052631579
chr17	76325868	76325905	4684	0.052631579
chr17	76527976	76528013	4685	0.052631579
chr17	76578925	76578962	4686	0.052631579
chr17	76615528	76615565	4687	0.052631579
chr17	76698434	76698471	4688	0.052631579
chr17	77026900	77026937	4689	0.052631579
chr17	77151967	77152004	4690	0.052631579
chr17	77485418	77485455	4691	0.052631579
chr17	77486168	77486205	4692	0.052631579
chr17	7750599	7750636	4693	0.052631579
chr17	7752824	7752861	4694	0.052631579
chr17	77610927	77610964	4695	0.052631579
chr17	77767757	77767794	4696	0.052631579
chr17	77834254	77834291	4697	0.052631579
chr17	77834762	77834799	4698	0.052631579
chr17	77868138	77868175	4699	0.052631579
chr17	78356712	78356749	4700	0.052631579

chr17	78481742	78481779	4701	0.052631579
chr17	78496267	78496304	4702	0.052631579
chr17	7910705	7910742	4703	0.052631579
chr17	8067340	8067377	4704	0.052631579
chr17	8467143	8467180	4705	0.052631579
chr17	8501628	8501665	4706	0.052631579
chr17	8866293	8866330	4707	0.052631579
chr17	9064861	9064898	4708	0.052631579
chr17	9180151	9180188	4709	0.052631579
chr17	9276747	9276784	4710	0.052631579
chr17	45626331	45626399	4711	0.057971014
chr17	68281797	68281863	4712	0.059701493
chr17	10367662	10367705	4713	0.068181818
chr17	10549133	10549172	4714	0.075
chr17	11990394	11990433	4715	0.075
chr17	12811401	12811440	4716	0.075
chr17	1286340	1286379	4717	0.075
chr17	1317887	1317926	4718	0.075
chr17	1318064	1318103	4719	0.075
chr17	13238685	13238724	4720	0.075
chr17	1327552	1327591	4721	0.075
chr17	1419424	1419463	4722	0.075
chr17	1425726	1425765	4723	0.075
chr17	17017223	17017262	4724	0.075
chr17	17643460	17643499	4725	0.075
chr17	17656398	17656437	4726	0.075
chr17	17945514	17945553	4727	0.075
chr17	18094757	18094796	4728	0.075
chr17	19290365	19290404	4729	0.075
chr17	19384683	19384722	4730	0.075
chr17	19522587	19522626	4731	0.075
chr17	1991291	1991330	4732	0.075
chr17	21245870	21245909	4733	0.075
chr17	23625407	23625446	4734	0.075
chr17	23967246	23967285	4735	0.075
chr17	2443313	2443352	4736	0.075
chr17	24982246	24982285	4737	0.075
chr17	25500533	25500572	4738	0.075
chr17	26777254	26777293	4739	0.075
chr17	27172207	27172246	4740	0.075
chr17	27948706	27948745	4741	0.075
chr17	28231601	28231640	4742	0.075
chr17	28288415	28288454	4743	0.075
chr17	29145042	29145081	4744	0.075
chr17	29324410	29324449	4745	0.075
chr17	29401432	29401471	4746	0.075
chr17	32020191	32020230	4747	0.075
chr17	3264757	3264796	4748	0.075
chr17	34134414	34134453	4749	0.075
chr17	34329596	34329635	4750	0.075

chr17	35039194	35039233	4751	0.075
chr17	35137289	35137328	4752	0.075
chr17	35140250	35140289	4753	0.075
chr17	35464137	35464176	4754	0.075
chr17	35510791	35510830	4755	0.075
chr17	35579144	35579183	4756	0.075
chr17	35853522	35853561	4757	0.075
chr17	35906126	35906165	4758	0.075
chr17	36160226	36160265	4759	0.075
chr17	36338875	36338914	4760	0.075
chr17	36847251	36847290	4761	0.075
chr17	36885901	36885940	4762	0.075
chr17	37053340	37053379	4763	0.075
chr17	37306674	37306713	4764	0.075
chr17	37906816	37906855	4765	0.075
chr17	37972843	37972882	4766	0.075
chr17	38204184	38204223	4767	0.075
chr17	39279968	39280007	4768	0.075
chr17	39390821	39390860	4769	0.075
chr17	39511199	39511238	4770	0.075
chr17	39624878	39624917	4771	0.075
chr17	39886843	39886882	4772	0.075
chr17	40122106	40122145	4773	0.075
chr17	40519398	40519437	4774	0.075
chr17	42411014	42411053	4775	0.075
chr17	42569679	42569718	4776	0.075
chr17	42719513	42719552	4777	0.075
chr17	43512215	43512254	4778	0.075
chr17	44032090	44032129	4779	0.075
chr17	4447233	4447272	4780	0.075
chr17	45621750	45621789	4781	0.075
chr17	45621856	45621895	4782	0.075
chr17	45633917	45633956	4783	0.075
chr17	45708076	45708115	4784	0.075
chr17	46049824	46049863	4785	0.075
chr17	46182996	46183035	4786	0.075
chr17	46888592	46888631	4787	0.075
chr17	470945	470984	4788	0.075
chr17	4739204	4739243	4789	0.075
chr17	4788613	4788652	4790	0.075
chr17	4866369	4866408	4791	0.075
chr17	49122948	49122987	4792	0.075
chr17	518823	518862	4793	0.075
chr17	5263605	5263644	4794	0.075
chr17	52697274	52697313	4795	0.075
chr17	53010599	53010638	4796	0.075
chr17	53349399	53349438	4797	0.075
chr17	55438333	55438372	4798	0.075
chr17	55911531	55911570	4799	0.075
chr17	56081501	56081540	4800	0.075

chr17	56358700	56358739	4801	0.075
chr17	56798933	56798972	4802	0.075
chr17	56828984	56829023	4803	0.075
chr17	57339361	57339400	4804	0.075
chr17	57909697	57909736	4805	0.075
chr17	59237646	59237685	4806	0.075
chr17	60008816	60008855	4807	0.075
chr17	61624690	61624729	4808	0.075
chr17	62803790	62803829	4809	0.075
chr17	62984821	62984860	4810	0.075
chr17	63868478	63868517	4811	0.075
chr17	64130835	64130874	4812	0.075
chr17	64931393	64931432	4813	0.075
chr17	65163331	65163370	4814	0.075
chr17	66169229	66169268	4815	0.075
chr17	66914775	66914814	4816	0.075
chr17	67648286	67648325	4817	0.075
chr17	67876841	67876880	4818	0.075
chr17	67891090	67891129	4819	0.075
chr17	67900993	67901032	4820	0.075
chr17	6860736	6860775	4821	0.075
chr17	68673566	68673605	4822	0.075
chr17	69347052	69347091	4823	0.075
chr17	6990871	6990910	4824	0.075
chr17	69943972	69944011	4825	0.075
chr17	70773228	70773267	4826	0.075
chr17	70991734	70991773	4827	0.075
chr17	71127070	71127109	4828	0.075
chr17	71237955	71237994	4829	0.075
chr17	71265647	71265686	4830	0.075
chr17	7173146	7173185	4831	0.075
chr17	71757548	71757587	4832	0.075
chr17	71822517	71822556	4833	0.075
chr17	71862880	71862919	4834	0.075
chr17	72134399	72134438	4835	0.075
chr17	7252654	7252693	4836	0.075
chr17	72564006	72564045	4837	0.075
chr17	7269559	7269598	4838	0.075
chr17	72805984	72806023	4839	0.075
chr17	73006204	73006243	4840	0.075
chr17	73341994	73342033	4841	0.075
chr17	7346560	7346599	4842	0.075
chr17	73578774	73578813	4843	0.075
chr17	73820712	73820751	4844	0.075
chr17	7393216	7393255	4845	0.075
chr17	7420853	7420892	4846	0.075
chr17	74502831	74502870	4847	0.075
chr17	7556352	7556391	4848	0.075
chr17	77136842	77136881	4849	0.075
chr17	77293165	77293204	4850	0.075

chr17	77420103	77420142	4851	0.075
chr17	77509899	77509938	4852	0.075
chr17	77634115	77634154	4853	0.075
chr17	78046767	78046806	4854	0.075
chr17	78127878	78127917	4855	0.075
chr17	8199371	8199410	4856	0.075
chr17	8639327	8639366	4857	0.075
chr17	9546402	9546441	4858	0.075
chr17	9633862	9633901	4859	0.075
chr17	2437600	2437638	4860	0.076923077
chr17	45626942	45626991	4861	0.08
chr17	38405541	38405591	4862	0.098039216
chr18	37300900	37301005	4863	0.037735849
chr18	10430918	10431020	4864	0.038834951
chr18	10108024	10108061	4865	0.052631579
chr18	10177936	10177973	4866	0.052631579
chr18	10521808	10521845	4867	0.052631579
chr18	10909837	10909874	4868	0.052631579
chr18	11055960	11055997	4869	0.052631579
chr18	11139529	11139566	4870	0.052631579
chr18	11267087	11267124	4871	0.052631579
chr18	11314549	11314586	4872	0.052631579
chr18	11573678	11573715	4873	0.052631579
chr18	11702566	11702603	4874	0.052631579
chr18	11871216	11871253	4875	0.052631579
chr18	11938803	11938840	4876	0.052631579
chr18	11991966	11992003	4877	0.052631579
chr18	12018093	12018130	4878	0.052631579
chr18	12100123	12100160	4879	0.052631579
chr18	12212951	12212988	4880	0.052631579
chr18	12315089	12315126	4881	0.052631579
chr18	13343981	13344018	4882	0.052631579
chr18	13440313	13440350	4883	0.052631579
chr18	13483301	13483338	4884	0.052631579
chr18	14561516	14561553	4885	0.052631579
chr18	167578	167615	4886	0.052631579
chr18	16825904	16825941	4887	0.052631579
chr18	17324073	17324110	4888	0.052631579
chr18	17399307	17399344	4889	0.052631579
chr18	17659705	17659742	4890	0.052631579
chr18	18013891	18013928	4891	0.052631579
chr18	18177117	18177154	4892	0.052631579
chr18	18610483	18610520	4893	0.052631579
chr18	18672227	18672264	4894	0.052631579
chr18	18885968	18886005	4895	0.052631579
chr18	19128263	19128300	4896	0.052631579
chr18	20388163	20388200	4897	0.052631579
chr18	20654910	20654947	4898	0.052631579
chr18	20785826	20785863	4899	0.052631579
chr18	21324503	21324540	4900	0.052631579

chr18	21749963	21750000	4901	0.052631579
chr18	22390020	22390057	4902	0.052631579
chr18	22639202	22639239	4903	0.052631579
chr18	2294604	2294641	4904	0.052631579
chr18	23070422	23070459	4905	0.052631579
chr18	23071214	23071251	4906	0.052631579
chr18	23123173	23123210	4907	0.052631579
chr18	23724392	23724429	4908	0.052631579
chr18	23845863	23845900	4909	0.052631579
chr18	23927132	23927169	4910	0.052631579
chr18	23927835	23927872	4911	0.052631579
chr18	23957764	23957801	4912	0.052631579
chr18	24343755	24343792	4913	0.052631579
chr18	24556827	24556864	4914	0.052631579
chr18	24632282	24632319	4915	0.052631579
chr18	24807371	24807408	4916	0.052631579
chr18	25143368	25143405	4917	0.052631579
chr18	25479066	25479103	4918	0.052631579
chr18	25820039	25820076	4919	0.052631579
chr18	26292637	26292674	4920	0.052631579
chr18	2718552	2718589	4921	0.052631579
chr18	2754196	2754233	4922	0.052631579
chr18	28607113	28607150	4923	0.052631579
chr18	28775455	28775492	4924	0.052631579
chr18	28776174	28776211	4925	0.052631579
chr18	29095924	29095961	4926	0.052631579
chr18	29305054	29305091	4927	0.052631579
chr18	30810990	30811027	4928	0.052631579
chr18	30825727	30825764	4929	0.052631579
chr18	30910727	30910764	4930	0.052631579
chr18	3092561	3092598	4931	0.052631579
chr18	31331592	31331629	4932	0.052631579
chr18	31417874	31417911	4933	0.052631579
chr18	3184398	3184435	4934	0.052631579
chr18	31970689	31970726	4935	0.052631579
chr18	32135362	32135399	4936	0.052631579
chr18	32399002	32399039	4937	0.052631579
chr18	32617060	32617097	4938	0.052631579
chr18	33113646	33113683	4939	0.052631579
chr18	33294418	33294455	4940	0.052631579
chr18	33416731	33416768	4941	0.052631579
chr18	33417430	33417467	4942	0.052631579
chr18	33504799	33504836	4943	0.052631579
chr18	33508867	33508904	4944	0.052631579
chr18	33788599	33788636	4945	0.052631579
chr18	34355037	34355074	4946	0.052631579
chr18	34446678	34446715	4947	0.052631579
chr18	34545842	34545879	4948	0.052631579
chr18	355951	355988	4949	0.052631579
chr18	35634811	35634848	4950	0.052631579

chr18	35635552	35635589	4951	0.052631579
chr18	356761	356798	4952	0.052631579
chr18	3599728	3599765	4953	0.052631579
chr18	37033536	37033573	4954	0.052631579
chr18	37600474	37600511	4955	0.052631579
chr18	37726574	37726611	4956	0.052631579
chr18	3792618	3792655	4957	0.052631579
chr18	38749483	38749520	4958	0.052631579
chr18	39156970	39157007	4959	0.052631579
chr18	39367201	39367238	4960	0.052631579
chr18	39613035	39613072	4961	0.052631579
chr18	40257273	40257310	4962	0.052631579
chr18	40393302	40393339	4963	0.052631579
chr18	40494060	40494097	4964	0.052631579
chr18	4060968	4061005	4965	0.052631579
chr18	40645637	40645674	4966	0.052631579
chr18	40770575	40770612	4967	0.052631579
chr18	40808492	40808529	4968	0.052631579
chr18	40831874	40831911	4969	0.052631579
chr18	40852494	40852531	4970	0.052631579
chr18	40902001	40902038	4971	0.052631579
chr18	40932711	40932748	4972	0.052631579
chr18	41126071	41126108	4973	0.052631579
chr18	41126344	41126381	4974	0.052631579
chr18	41183441	41183478	4975	0.052631579
chr18	41362128	41362165	4976	0.052631579
chr18	41657648	41657685	4977	0.052631579
chr18	41850051	41850088	4978	0.052631579
chr18	42365352	42365389	4979	0.052631579
chr18	42801158	42801195	4980	0.052631579
chr18	42812224	42812261	4981	0.052631579
chr18	43056167	43056204	4982	0.052631579
chr18	43631301	43631338	4983	0.052631579
chr18	44365452	44365489	4984	0.052631579
chr18	44655010	44655047	4985	0.052631579
chr18	45346057	45346094	4986	0.052631579
chr18	45421975	45422012	4987	0.052631579
chr18	45685784	45685821	4988	0.052631579
chr18	45860559	45860596	4989	0.052631579
chr18	46064425	46064462	4990	0.052631579
chr18	46078863	46078900	4991	0.052631579
chr18	46541001	46541038	4992	0.052631579
chr18	46561175	46561212	4993	0.052631579
chr18	47131900	47131937	4994	0.052631579
chr18	47449331	47449368	4995	0.052631579
chr18	47449764	47449801	4996	0.052631579
chr18	47503139	47503176	4997	0.052631579
chr18	48475450	48475487	4998	0.052631579
chr18	49382611	49382648	4999	0.052631579
chr18	49393891	49393928	5000	0.052631579

chr18	49540802	49540839	5001	0.052631579
chr18	49541447	49541484	5002	0.052631579
chr18	49878238	49878275	5003	0.052631579
chr18	50084057	50084094	5004	0.052631579
chr18	50200336	50200373	5005	0.052631579
chr18	50238132	50238169	5006	0.052631579
chr18	50376002	50376039	5007	0.052631579
chr18	50381162	50381199	5008	0.052631579
chr18	50942124	50942161	5009	0.052631579
chr18	50948713	50948750	5010	0.052631579
chr18	51071546	51071583	5011	0.052631579
chr18	51360888	51360925	5012	0.052631579
chr18	51756771	51756808	5013	0.052631579
chr18	51808821	51808858	5014	0.052631579
chr18	51914525	51914562	5015	0.052631579
chr18	52788371	52788408	5016	0.052631579
chr18	52978859	52978896	5017	0.052631579
chr18	53728134	53728171	5018	0.052631579
chr18	54077321	54077358	5019	0.052631579
chr18	54173926	54173963	5020	0.052631579
chr18	54222568	54222605	5021	0.052631579
chr18	5425864	5425901	5022	0.052631579
chr18	54656430	54656467	5023	0.052631579
chr18	55282151	55282188	5024	0.052631579
chr18	56659114	56659151	5025	0.052631579
chr18	56879721	56879758	5026	0.052631579
chr18	56950816	56950853	5027	0.052631579
chr18	56970358	56970395	5028	0.052631579
chr18	58964407	58964444	5029	0.052631579
chr18	59094341	59094378	5030	0.052631579
chr18	59111511	59111548	5031	0.052631579
chr18	59737002	59737039	5032	0.052631579
chr18	59737693	59737730	5033	0.052631579
chr18	59851156	59851193	5034	0.052631579
chr18	60091877	60091914	5035	0.052631579
chr18	61008632	61008669	5036	0.052631579
chr18	61036070	61036107	5037	0.052631579
chr18	61127623	61127660	5038	0.052631579
chr18	61184680	61184717	5039	0.052631579
chr18	61502251	61502288	5040	0.052631579
chr18	61638279	61638316	5041	0.052631579
chr18	62315059	62315096	5042	0.052631579
chr18	63187675	63187712	5043	0.052631579
chr18	63297368	63297405	5044	0.052631579
chr18	65228337	65228374	5045	0.052631579
chr18	65312737	65312774	5046	0.052631579
chr18	65529078	65529115	5047	0.052631579
chr18	65716856	65716893	5048	0.052631579
chr18	65717284	65717321	5049	0.052631579
chr18	66143445	66143482	5050	0.052631579

chr18	66258067	66258104	5051	0.052631579
chr18	66464118	66464155	5052	0.052631579
chr18	6665316	6665353	5053	0.052631579
chr18	66719790	66719827	5054	0.052631579
chr18	67600921	67600958	5055	0.052631579
chr18	68919609	68919646	5056	0.052631579
chr18	69158184	69158221	5057	0.052631579
chr18	69158326	69158363	5058	0.052631579
chr18	6940701	6940738	5059	0.052631579
chr18	69537460	69537497	5060	0.052631579
chr18	69569414	69569451	5061	0.052631579
chr18	69726669	69726706	5062	0.052631579
chr18	69976557	69976594	5063	0.052631579
chr18	70414296	70414333	5064	0.052631579
chr18	70645880	70645917	5065	0.052631579
chr18	7078197	7078234	5066	0.052631579
chr18	70870476	70870513	5067	0.052631579
chr18	70913628	70913665	5068	0.052631579
chr18	71068355	71068392	5069	0.052631579
chr18	71316265	71316302	5070	0.052631579
chr18	71526882	71526919	5071	0.052631579
chr18	71556248	71556285	5072	0.052631579
chr18	71699725	71699762	5073	0.052631579
chr18	71699853	71699890	5074	0.052631579
chr18	72124445	72124482	5075	0.052631579
chr18	72158413	72158450	5076	0.052631579
chr18	72285314	72285351	5077	0.052631579
chr18	72736302	72736339	5078	0.052631579
chr18	72974113	72974150	5079	0.052631579
chr18	73195918	73195955	5080	0.052631579
chr18	73932942	73932979	5081	0.052631579
chr18	73933707	73933744	5082	0.052631579
chr18	73989649	73989686	5083	0.052631579
chr18	74108476	74108513	5084	0.052631579
chr18	74370076	74370113	5085	0.052631579
chr18	74439069	74439106	5086	0.052631579
chr18	74563345	74563382	5087	0.052631579
chr18	74854955	74854992	5088	0.052631579
chr18	75137090	75137127	5089	0.052631579
chr18	7517925	7517962	5090	0.052631579
chr18	75231610	75231647	5091	0.052631579
chr18	75734618	75734655	5092	0.052631579
chr18	75738606	75738643	5093	0.052631579
chr18	8017632	8017669	5094	0.052631579
chr18	8952228	8952265	5095	0.052631579
chr18	9059819	9059856	5096	0.052631579
chr18	9506470	9506507	5097	0.052631579
chr18	9535167	9535204	5098	0.052631579
chr18	9553094	9553131	5099	0.052631579
chr18	9651018	9651055	5100	0.052631579

chr18	2897238	2897306	5101	0.057971014
chr18	43366628	43366694	5102	0.059701493
chr18	10202041	10202094	5103	0.074074074
chr18	10160180	10160219	5104	0.075
chr18	11165223	11165262	5105	0.075
chr18	11749748	11749787	5106	0.075
chr18	12088245	12088284	5107	0.075
chr18	12961218	12961257	5108	0.075
chr18	12999467	12999506	5109	0.075
chr18	13311456	13311495	5110	0.075
chr18	13318595	13318634	5111	0.075
chr18	13353457	13353496	5112	0.075
chr18	17328661	17328700	5113	0.075
chr18	17376477	17376516	5114	0.075
chr18	17448760	17448799	5115	0.075
chr18	17680958	17680997	5116	0.075
chr18	18997698	18997737	5117	0.075
chr18	19070599	19070638	5118	0.075
chr18	19834084	19834123	5119	0.075
chr18	20635714	20635753	5120	0.075
chr18	20772621	20772660	5121	0.075
chr18	20853720	20853759	5122	0.075
chr18	21119853	21119892	5123	0.075
chr18	22537802	22537841	5124	0.075
chr18	22623530	22623569	5125	0.075
chr18	24935898	24935937	5126	0.075
chr18	26221469	26221508	5127	0.075
chr18	28121555	28121594	5128	0.075
chr18	2941359	2941398	5129	0.075
chr18	30672734	30672773	5130	0.075
chr18	31171341	31171380	5131	0.075
chr18	31861754	31861793	5132	0.075
chr18	31975350	31975389	5133	0.075
chr18	32522978	32523017	5134	0.075
chr18	33101297	33101336	5135	0.075
chr18	33534582	33534621	5136	0.075
chr18	38285766	38285805	5137	0.075
chr18	39469377	39469416	5138	0.075
chr18	4031420	4031459	5139	0.075
chr18	40510597	40510636	5140	0.075
chr18	41608809	41608848	5141	0.075
chr18	42108251	42108290	5142	0.075
chr18	42226789	42226828	5143	0.075
chr18	42293659	42293698	5144	0.075
chr18	42340027	42340066	5145	0.075
chr18	42896915	42896954	5146	0.075
chr18	43414384	43414423	5147	0.075
chr18	44549687	44549726	5148	0.075
chr18	44772065	44772104	5149	0.075
chr18	44847753	44847792	5150	0.075

chr18	45079145	45079184	5151	0.075
chr18	46071005	46071044	5152	0.075
chr18	46175039	46175078	5153	0.075
chr18	46533520	46533559	5154	0.075
chr18	46647100	46647139	5155	0.075
chr18	46956973	46957012	5156	0.075
chr18	46976081	46976120	5157	0.075
chr18	4841721	4841760	5158	0.075
chr18	48668613	48668652	5159	0.075
chr18	48680693	48680732	5160	0.075
chr18	49024262	49024301	5161	0.075
chr18	50004915	50004954	5162	0.075
chr18	50518858	50518897	5163	0.075
chr18	54208955	54208994	5164	0.075
chr18	54766349	54766388	5165	0.075
chr18	57693525	57693564	5166	0.075
chr18	5879307	5879346	5167	0.075
chr18	60149121	60149160	5168	0.075
chr18	62965241	62965280	5169	0.075
chr18	64576140	64576179	5170	0.075
chr18	64933732	64933771	5171	0.075
chr18	64985703	64985742	5172	0.075
chr18	65005800	65005839	5173	0.075
chr18	66271985	66272024	5174	0.075
chr18	66522610	66522649	5175	0.075
chr18	67754977	67755016	5176	0.075
chr18	68109326	68109365	5177	0.075
chr18	68674474	68674513	5178	0.075
chr18	69881639	69881678	5179	0.075
chr18	70109505	70109544	5180	0.075
chr18	70117276	70117315	5181	0.075
chr18	71835703	71835742	5182	0.075
chr18	72187474	72187513	5183	0.075
chr18	72251144	72251183	5184	0.075
chr18	72938676	72938715	5185	0.075
chr18	73048777	73048816	5186	0.075
chr18	73234150	73234189	5187	0.075
chr18	73896639	73896678	5188	0.075
chr18	75408384	75408423	5189	0.075
chr18	75647970	75648009	5190	0.075
chr18	75679601	75679640	5191	0.075
chr18	9234144	9234183	5192	0.075
chr18	9550341	9550380	5193	0.075
chr18	9904163	9904202	5194	0.075
chr18	55308121	55308159	5195	0.076923077
chr18	59241152	59241200	5196	0.081632653
chr18	10965628	10965674	5197	0.085106383
chr18	58409410	58409450	5198	0.097560976
chr19	1175308	1175386	5199	0.050632911
chr19	2080631	2080707	5200	0.051948052

chr19	10091325	10091362	5201	0.052631579
chr19	10422545	10422582	5202	0.052631579
chr19	10486656	10486693	5203	0.052631579
chr19	10885661	10885698	5204	0.052631579
chr19	11136035	11136072	5205	0.052631579
chr19	1115336	1115373	5206	0.052631579
chr19	11430069	11430106	5207	0.052631579
chr19	11848117	11848154	5208	0.052631579
chr19	11901934	11901971	5209	0.052631579
chr19	1202053	1202090	5210	0.052631579
chr19	12373161	12373198	5211	0.052631579
chr19	12687510	12687547	5212	0.052631579
chr19	12869139	12869176	5213	0.052631579
chr19	13787780	13787817	5214	0.052631579
chr19	13884165	13884202	5215	0.052631579
chr19	14108989	14109026	5216	0.052631579
chr19	14381339	14381376	5217	0.052631579
chr19	14943575	14943612	5218	0.052631579
chr19	1496738	1496775	5219	0.052631579
chr19	15146150	15146187	5220	0.052631579
chr19	15340980	15341017	5221	0.052631579
chr19	15369016	15369053	5222	0.052631579
chr19	15586632	15586669	5223	0.052631579
chr19	15635867	15635904	5224	0.052631579
chr19	16343584	16343621	5225	0.052631579
chr19	17656439	17656476	5226	0.052631579
chr19	18110892	18110929	5227	0.052631579
chr19	18140982	18141019	5228	0.052631579
chr19	18622497	18622534	5229	0.052631579
chr19	18832372	18832409	5230	0.052631579
chr19	18918644	18918681	5231	0.052631579
chr19	18988142	18988179	5232	0.052631579
chr19	18994672	18994709	5233	0.052631579
chr19	19121141	19121178	5234	0.052631579
chr19	19122581	19122618	5235	0.052631579
chr19	19249596	19249633	5236	0.052631579
chr19	19377043	19377080	5237	0.052631579
chr19	19510010	19510047	5238	0.052631579
chr19	19606977	19607014	5239	0.052631579
chr19	19618996	19619033	5240	0.052631579
chr19	19880810	19880847	5241	0.052631579
chr19	20296039	20296076	5242	0.052631579
chr19	20418226	20418263	5243	0.052631579
chr19	208437	208474	5244	0.052631579
chr19	2171081	2171118	5245	0.052631579
chr19	2191699	2191736	5246	0.052631579
chr19	2200547	2200584	5247	0.052631579
chr19	22407771	22407808	5248	0.052631579
chr19	23083096	23083133	5249	0.052631579
chr19	23254788	23254825	5250	0.052631579

chr19	24074144	24074181	5251	0.052631579
chr19	261217	261254	5252	0.052631579
chr19	2668564	2668601	5253	0.052631579
chr19	2851682	2851719	5254	0.052631579
chr19	2979084	2979121	5255	0.052631579
chr19	3004203	3004240	5256	0.052631579
chr19	33071738	33071775	5257	0.052631579
chr19	33093662	33093699	5258	0.052631579
chr19	33328143	33328180	5259	0.052631579
chr19	33392728	33392765	5260	0.052631579
chr19	34182713	34182750	5261	0.052631579
chr19	34426273	34426310	5262	0.052631579
chr19	34520935	34520972	5263	0.052631579
chr19	35337354	35337391	5264	0.052631579
chr19	35439985	35440022	5265	0.052631579
chr19	35459779	35459816	5266	0.052631579
chr19	35721858	35721895	5267	0.052631579
chr19	35771721	35771758	5268	0.052631579
chr19	36226504	36226541	5269	0.052631579
chr19	36455383	36455420	5270	0.052631579
chr19	36497155	36497192	5271	0.052631579
chr19	36509290	36509327	5272	0.052631579
chr19	36521742	36521779	5273	0.052631579
chr19	36660656	36660693	5274	0.052631579
chr19	36728546	36728583	5275	0.052631579
chr19	3702248	3702285	5276	0.052631579
chr19	37089104	37089141	5277	0.052631579
chr19	37279547	37279584	5278	0.052631579
chr19	37335083	37335120	5279	0.052631579
chr19	37411902	37411939	5280	0.052631579
chr19	37468665	37468702	5281	0.052631579
chr19	37928732	37928769	5282	0.052631579
chr19	38923784	38923821	5283	0.052631579
chr19	3935153	3935190	5284	0.052631579
chr19	3958381	3958418	5285	0.052631579
chr19	39926967	39927004	5286	0.052631579
chr19	40596395	40596432	5287	0.052631579
chr19	4061494	4061531	5288	0.052631579
chr19	40812303	40812340	5289	0.052631579
chr19	41062734	41062771	5290	0.052631579
chr19	41408168	41408205	5291	0.052631579
chr19	41419459	41419496	5292	0.052631579
chr19	42392332	42392369	5293	0.052631579
chr19	42800356	42800393	5294	0.052631579
chr19	43404719	43404756	5295	0.052631579
chr19	43628518	43628555	5296	0.052631579
chr19	4371556	4371593	5297	0.052631579
chr19	43755699	43755736	5298	0.052631579
chr19	45027345	45027382	5299	0.052631579
chr19	45027636	45027673	5300	0.052631579

chr19	45390181	45390218	5301	0.052631579
chr19	45809697	45809734	5302	0.052631579
chr19	45809824	45809861	5303	0.052631579
chr19	45931043	45931080	5304	0.052631579
chr19	46472534	46472571	5305	0.052631579
chr19	46611796	46611833	5306	0.052631579
chr19	46634086	46634123	5307	0.052631579
chr19	47118467	47118504	5308	0.052631579
chr19	47283197	47283234	5309	0.052631579
chr19	47305418	47305455	5310	0.052631579
chr19	47512475	47512512	5311	0.052631579
chr19	47720005	47720042	5312	0.052631579
chr19	47773388	47773425	5313	0.052631579
chr19	47891961	47891998	5314	0.052631579
chr19	4790424	4790461	5315	0.052631579
chr19	4819449	4819486	5316	0.052631579
chr19	48205295	48205332	5317	0.052631579
chr19	48379170	48379207	5318	0.052631579
chr19	50016264	50016301	5319	0.052631579
chr19	50036365	50036402	5320	0.052631579
chr19	50187436	50187473	5321	0.052631579
chr19	5057254	5057291	5322	0.052631579
chr19	50595217	50595254	5323	0.052631579
chr19	50713110	50713147	5324	0.052631579
chr19	51067194	51067231	5325	0.052631579
chr19	51101733	51101770	5326	0.052631579
chr19	51306139	51306176	5327	0.052631579
chr19	51585525	51585562	5328	0.052631579
chr19	5162685	5162722	5329	0.052631579
chr19	51804338	51804375	5330	0.052631579
chr19	51908057	51908094	5331	0.052631579
chr19	51982661	51982698	5332	0.052631579
chr19	52188190	52188227	5333	0.052631579
chr19	52398323	52398360	5334	0.052631579
chr19	52660948	52660985	5335	0.052631579
chr19	52676963	52677000	5336	0.052631579
chr19	53506766	53506803	5337	0.052631579
chr19	53614749	53614786	5338	0.052631579
chr19	53636997	53637034	5339	0.052631579
chr19	53669948	53669985	5340	0.052631579
chr19	53824870	53824907	5341	0.052631579
chr19	53825334	53825371	5342	0.052631579
chr19	5398357	5398394	5343	0.052631579
chr19	54293780	54293817	5344	0.052631579
chr19	54697561	54697598	5345	0.052631579
chr19	54709309	54709346	5346	0.052631579
chr19	54719964	54720001	5347	0.052631579
chr19	54977690	54977727	5348	0.052631579
chr19	55504529	55504566	5349	0.052631579
chr19	5645813	5645850	5350	0.052631579

chr19	56466089	56466126	5351	0.052631579
chr19	58867262	58867299	5352	0.052631579
chr19	5891153	5891190	5353	0.052631579
chr19	59050002	59050039	5354	0.052631579
chr19	59310441	59310478	5355	0.052631579
chr19	60858259	60858296	5356	0.052631579
chr19	60894906	60894943	5357	0.052631579
chr19	61163578	61163615	5358	0.052631579
chr19	61330253	61330290	5359	0.052631579
chr19	61411153	61411190	5360	0.052631579
chr19	61521626	61521663	5361	0.052631579
chr19	62202885	62202922	5362	0.052631579
chr19	62638460	62638497	5363	0.052631579
chr19	632524	632561	5364	0.052631579
chr19	6367497	6367534	5365	0.052631579
chr19	7074613	7074650	5366	0.052631579
chr19	7287014	7287051	5367	0.052631579
chr19	7786072	7786109	5368	0.052631579
chr19	7899023	7899060	5369	0.052631579
chr19	7934494	7934531	5370	0.052631579
chr19	8937464	8937501	5371	0.052631579
chr19	9390587	9390624	5372	0.052631579
chr19	9764581	9764618	5373	0.052631579
chr19	55712943	55713010	5374	0.058823529
chr19	3928461	3928504	5375	0.068181818
chr19	1005290	1005329	5376	0.075
chr19	10369834	10369873	5377	0.075
chr19	10608547	10608586	5378	0.075
chr19	10660311	10660350	5379	0.075
chr19	1224029	1224068	5380	0.075
chr19	12615109	12615148	5381	0.075
chr19	12675556	12675595	5382	0.075
chr19	12758429	12758468	5383	0.075
chr19	12919684	12919723	5384	0.075
chr19	12958114	12958153	5385	0.075
chr19	13110001	13110040	5386	0.075
chr19	13652194	13652233	5387	0.075
chr19	14094510	14094549	5388	0.075
chr19	15451163	15451202	5389	0.075
chr19	16205361	16205400	5390	0.075
chr19	17377928	17377967	5391	0.075
chr19	18545506	18545545	5392	0.075
chr19	18546707	18546746	5393	0.075
chr19	18563885	18563924	5394	0.075
chr19	20208934	20208973	5395	0.075
chr19	24006112	24006151	5396	0.075
chr19	2970356	2970395	5397	0.075
chr19	3327130	3327169	5398	0.075
chr19	3431345	3431384	5399	0.075
chr19	3447616	3447655	5400	0.075

chr19	3486503	3486542	5401	0.075
chr19	35832830	35832869	5402	0.075
chr19	36083949	36083988	5403	0.075
chr19	36985485	36985524	5404	0.075
chr19	37065059	37065098	5405	0.075
chr19	37447785	37447824	5406	0.075
chr19	37842453	37842492	5407	0.075
chr19	39098284	39098323	5408	0.075
chr19	3928250	3928289	5409	0.075
chr19	3928866	3928905	5410	0.075
chr19	3979565	3979604	5411	0.075
chr19	40050485	40050524	5412	0.075
chr19	41411500	41411539	5413	0.075
chr19	4339700	4339739	5414	0.075
chr19	43475891	43475930	5415	0.075
chr19	43806717	43806756	5416	0.075
chr19	44114991	44115030	5417	0.075
chr19	44356364	44356403	5418	0.075
chr19	44618350	44618389	5419	0.075
chr19	44653928	44653967	5420	0.075
chr19	45141444	45141483	5421	0.075
chr19	45974747	45974786	5422	0.075
chr19	47057052	47057091	5423	0.075
chr19	47078240	47078279	5424	0.075
chr19	47144518	47144557	5425	0.075
chr19	47483660	47483699	5426	0.075
chr19	47572399	47572438	5427	0.075
chr19	47602146	47602185	5428	0.075
chr19	47743803	47743842	5429	0.075
chr19	486597	486636	5430	0.075
chr19	49113805	49113844	5431	0.075
chr19	49189714	49189753	5432	0.075
chr19	49483365	49483404	5433	0.075
chr19	49702869	49702908	5434	0.075
chr19	50336472	50336511	5435	0.075
chr19	50414870	50414909	5436	0.075
chr19	50459961	50460000	5437	0.075
chr19	50498701	50498740	5438	0.075
chr19	50973249	50973288	5439	0.075
chr19	51149489	51149528	5440	0.075
chr19	51156205	51156244	5441	0.075
chr19	52115357	52115396	5442	0.075
chr19	530584	530623	5443	0.075
chr19	53966081	53966120	5444	0.075
chr19	54160843	54160882	5445	0.075
chr19	54826255	54826294	5446	0.075
chr19	54831993	54832032	5447	0.075
chr19	54848366	54848405	5448	0.075
chr19	55063620	55063659	5449	0.075
chr19	55132385	55132424	5450	0.075

chr19	55713464	55713503	5451	0.075
chr19	5574334	5574373	5452	0.075
chr19	56797836	56797875	5453	0.075
chr19	56923854	56923893	5454	0.075
chr19	5736073	5736112	5455	0.075
chr19	5884261	5884300	5456	0.075
chr19	61035492	61035531	5457	0.075
chr19	61101337	61101376	5458	0.075
chr19	6319854	6319893	5459	0.075
chr19	63213079	63213118	5460	0.075
chr19	7071718	7071757	5461	0.075
chr19	7510802	7510841	5462	0.075
chr19	7875937	7875976	5463	0.075
chr19	8401711	8401750	5464	0.075
chr19	9132420	9132459	5465	0.075
chr19	9793583	9793622	5466	0.075
chr19	13935669	13935707	5467	0.076923077
chr19	21004685	21004736	5468	0.076923077
chr19	22831379	22831429	5469	0.078431373
chr19	7098742	7098781	5470	0.1
chr2	68337794	68337908	5471	0.034782609
chr2	70663963	70664076	5472	0.035087719
chr2	26057243	26057353	5473	0.036036036
chr2	55461007	55461086	5474	0.0375
chr2	28489233	28489335	5475	0.038834951
chr2	173069050	173069150	5476	0.03960396
chr2	42573802	42573870	5477	0.043478261
chr2	134728488	134728555	5478	0.044117647
chr2	226597322	226597405	5479	0.047619048
chr2	215945104	215945165	5480	0.048387097
chr2	80420328	80420409	5481	0.048780488
chr2	14300648	14300728	5482	0.049382716
chr2	237941998	237942076	5483	0.050632911
chr2	100145397	100145434	5484	0.052631579
chr2	100646874	100646911	5485	0.052631579
chr2	100807048	100807085	5486	0.052631579
chr2	100912989	100913026	5487	0.052631579
chr2	101061741	101061778	5488	0.052631579
chr2	101560570	101560607	5489	0.052631579
chr2	101942532	101942569	5490	0.052631579
chr2	102035846	102035883	5491	0.052631579
chr2	102161378	102161415	5492	0.052631579
chr2	102314335	102314372	5493	0.052631579
chr2	102637494	102637531	5494	0.052631579
chr2	102942413	102942450	5495	0.052631579
chr2	103130320	103130357	5496	0.052631579
chr2	103131090	103131127	5497	0.052631579
chr2	103411492	103411529	5498	0.052631579
chr2	104138899	104138936	5499	0.052631579
chr2	104216565	104216602	5500	0.052631579

chr2	104375717	104375754	5501	0.052631579
chr2	104501415	104501452	5502	0.052631579
chr2	104502046	104502083	5503	0.052631579
chr2	104782675	104782712	5504	0.052631579
chr2	10480621	10480658	5505	0.052631579
chr2	10572948	10572985	5506	0.052631579
chr2	105848670	105848707	5507	0.052631579
chr2	106266943	106266980	5508	0.052631579
chr2	106540563	106540600	5509	0.052631579
chr2	106968419	106968456	5510	0.052631579
chr2	106976694	106976731	5511	0.052631579
chr2	108010903	108010940	5512	0.052631579
chr2	108132902	108132939	5513	0.052631579
chr2	10850709	10850746	5514	0.052631579
chr2	108568750	108568787	5515	0.052631579
chr2	108979198	108979235	5516	0.052631579
chr2	109156800	109156837	5517	0.052631579
chr2	109264953	109264990	5518	0.052631579
chr2	109324852	109324889	5519	0.052631579
chr2	109325385	109325422	5520	0.052631579
chr2	10953101	10953138	5521	0.052631579
chr2	109760694	109760731	5522	0.052631579
chr2	111468034	111468071	5523	0.052631579
chr2	111625960	111625997	5524	0.052631579
chr2	11162601	11162638	5525	0.052631579
chr2	111635783	111635820	5526	0.052631579
chr2	111637057	111637094	5527	0.052631579
chr2	112129710	112129747	5528	0.052631579
chr2	11259193	11259230	5529	0.052631579
chr2	112669757	112669794	5530	0.052631579
chr2	112788206	112788243	5531	0.052631579
chr2	113195383	113195420	5532	0.052631579
chr2	113694393	113694430	5533	0.052631579
chr2	114517994	114518031	5534	0.052631579
chr2	115258903	115258940	5535	0.052631579
chr2	115264434	115264471	5536	0.052631579
chr2	115997732	115997769	5537	0.052631579
chr2	11601405	11601442	5538	0.052631579
chr2	116190883	116190920	5539	0.052631579
chr2	117243068	117243105	5540	0.052631579
chr2	117254673	117254710	5541	0.052631579
chr2	118096106	118096143	5542	0.052631579
chr2	11823145	11823182	5543	0.052631579
chr2	118603592	118603629	5544	0.052631579
chr2	118657176	118657213	5545	0.052631579
chr2	119256397	119256434	5546	0.052631579
chr2	119598562	119598599	5547	0.052631579
chr2	119599080	119599117	5548	0.052631579
chr2	119756663	119756700	5549	0.052631579
chr2	119757179	119757216	5550	0.052631579

chr2	119998609	119998646	5551	0.052631579
chr2	120198055	120198092	5552	0.052631579
chr2	120495958	120495995	5553	0.052631579
chr2	120741525	120741562	5554	0.052631579
chr2	120824348	120824385	5555	0.052631579
chr2	12093495	12093532	5556	0.052631579
chr2	121451060	121451097	5557	0.052631579
chr2	122723187	122723224	5558	0.052631579
chr2	122938229	122938266	5559	0.052631579
chr2	12356497	12356534	5560	0.052631579
chr2	123644100	123644137	5561	0.052631579
chr2	123907022	123907059	5562	0.052631579
chr2	123938612	123938649	5563	0.052631579
chr2	124236383	124236420	5564	0.052631579
chr2	124347213	124347250	5565	0.052631579
chr2	124444401	124444438	5566	0.052631579
chr2	124958856	124958893	5567	0.052631579
chr2	12511032	12511069	5568	0.052631579
chr2	127376572	127376609	5569	0.052631579
chr2	127538036	127538073	5570	0.052631579
chr2	127638855	127638892	5571	0.052631579
chr2	127745211	127745248	5572	0.052631579
chr2	127747061	127747098	5573	0.052631579
chr2	127761064	127761101	5574	0.052631579
chr2	127791822	127791859	5575	0.052631579
chr2	128285290	128285327	5576	0.052631579
chr2	128747537	128747574	5577	0.052631579
chr2	129354500	129354537	5578	0.052631579
chr2	129756445	129756482	5579	0.052631579
chr2	130016093	130016130	5580	0.052631579
chr2	13110098	13110135	5581	0.052631579
chr2	131439990	131440027	5582	0.052631579
chr2	131974005	131974042	5583	0.052631579
chr2	132555038	132555075	5584	0.052631579
chr2	132887991	132888028	5585	0.052631579
chr2	132953541	132953578	5586	0.052631579
chr2	133344609	133344646	5587	0.052631579
chr2	133583659	133583696	5588	0.052631579
chr2	133632275	133632312	5589	0.052631579
chr2	134038882	134038919	5590	0.052631579
chr2	134129433	134129470	5591	0.052631579
chr2	134266155	134266192	5592	0.052631579
chr2	134411957	134411994	5593	0.052631579
chr2	134492055	134492092	5594	0.052631579
chr2	134565281	134565318	5595	0.052631579
chr2	134607387	134607424	5596	0.052631579
chr2	134649020	134649057	5597	0.052631579
chr2	134928402	134928439	5598	0.052631579
chr2	135055825	135055862	5599	0.052631579
chr2	135339452	135339489	5600	0.052631579

chr2	136108155	136108192	5601	0.052631579
chr2	136705087	136705124	5602	0.052631579
chr2	136713712	136713749	5603	0.052631579
chr2	136764335	136764372	5604	0.052631579
chr2	136849780	136849817	5605	0.052631579
chr2	136849956	136849993	5606	0.052631579
chr2	138071055	138071092	5607	0.052631579
chr2	138428825	138428862	5608	0.052631579
chr2	138539003	138539040	5609	0.052631579
chr2	139477450	139477487	5610	0.052631579
chr2	139849576	139849613	5611	0.052631579
chr2	140426112	140426149	5612	0.052631579
chr2	140782428	140782465	5613	0.052631579
chr2	140917981	140918018	5614	0.052631579
chr2	140949304	140949341	5615	0.052631579
chr2	141381966	141382003	5616	0.052631579
chr2	141491172	141491209	5617	0.052631579
chr2	142317391	142317428	5618	0.052631579
chr2	14270445	14270482	5619	0.052631579
chr2	14286122	14286159	5620	0.052631579
chr2	142895116	142895153	5621	0.052631579
chr2	143017188	143017225	5622	0.052631579
chr2	143066706	143066743	5623	0.052631579
chr2	14328242	14328279	5624	0.052631579
chr2	143435808	143435845	5625	0.052631579
chr2	144047430	144047467	5626	0.052631579
chr2	144124109	144124146	5627	0.052631579
chr2	144279964	144280001	5628	0.052631579
chr2	144285333	144285370	5629	0.052631579
chr2	144377152	144377189	5630	0.052631579
chr2	144431615	144431652	5631	0.052631579
chr2	144523246	144523283	5632	0.052631579
chr2	144542543	144542580	5633	0.052631579
chr2	144542625	144542662	5634	0.052631579
chr2	144662620	144662657	5635	0.052631579
chr2	144863973	144864010	5636	0.052631579
chr2	144964896	144964933	5637	0.052631579
chr2	144984833	144984870	5638	0.052631579
chr2	145070818	145070855	5639	0.052631579
chr2	145450251	145450288	5640	0.052631579
chr2	145533666	145533703	5641	0.052631579
chr2	145678083	145678120	5642	0.052631579
chr2	146162875	146162912	5643	0.052631579
chr2	147235190	147235227	5644	0.052631579
chr2	147301038	147301075	5645	0.052631579
chr2	147369534	147369571	5646	0.052631579
chr2	147570763	147570800	5647	0.052631579
chr2	147735810	147735847	5648	0.052631579
chr2	147934388	147934425	5649	0.052631579
chr2	148124733	148124770	5650	0.052631579

chr2	148541463	148541500	5651	0.052631579
chr2	148952106	148952143	5652	0.052631579
chr2	149106968	149107005	5653	0.052631579
chr2	149119477	149119514	5654	0.052631579
chr2	149549957	149549994	5655	0.052631579
chr2	150268646	150268683	5656	0.052631579
chr2	150289509	150289546	5657	0.052631579
chr2	150559001	150559038	5658	0.052631579
chr2	150929451	150929488	5659	0.052631579
chr2	150977893	150977930	5660	0.052631579
chr2	15119456	15119493	5661	0.052631579
chr2	151262074	151262111	5662	0.052631579
chr2	151842003	151842040	5663	0.052631579
chr2	152179114	152179151	5664	0.052631579
chr2	15220229	15220266	5665	0.052631579
chr2	152239293	152239330	5666	0.052631579
chr2	152411827	152411864	5667	0.052631579
chr2	152609282	152609319	5668	0.052631579
chr2	15272957	15272994	5669	0.052631579
chr2	152866523	152866560	5670	0.052631579
chr2	152867298	152867335	5671	0.052631579
chr2	153281971	153282008	5672	0.052631579
chr2	153543643	153543680	5673	0.052631579
chr2	153713608	153713645	5674	0.052631579
chr2	153911179	153911216	5675	0.052631579
chr2	154041594	154041631	5676	0.052631579
chr2	154042369	154042406	5677	0.052631579
chr2	154705060	154705097	5678	0.052631579
chr2	155211181	155211218	5679	0.052631579
chr2	155265968	155266005	5680	0.052631579
chr2	155859973	155860010	5681	0.052631579
chr2	156366091	156366128	5682	0.052631579
chr2	156366885	156366922	5683	0.052631579
chr2	157216734	157216771	5684	0.052631579
chr2	157342697	157342734	5685	0.052631579
chr2	157371592	157371629	5686	0.052631579
chr2	157570275	157570312	5687	0.052631579
chr2	157726334	157726371	5688	0.052631579
chr2	157915885	157915922	5689	0.052631579
chr2	15861604	15861641	5690	0.052631579
chr2	159019895	159019932	5691	0.052631579
chr2	159219342	159219379	5692	0.052631579
chr2	159220055	159220092	5693	0.052631579
chr2	159533323	159533360	5694	0.052631579
chr2	159619488	159619525	5695	0.052631579
chr2	159619592	159619629	5696	0.052631579
chr2	160090948	160090985	5697	0.052631579
chr2	160200033	160200070	5698	0.052631579
chr2	160218624	160218661	5699	0.052631579
chr2	160616841	160616878	5700	0.052631579

chr2	160974273	160974310	5701	0.052631579
chr2	161076536	161076573	5702	0.052631579
chr2	161081577	161081614	5703	0.052631579
chr2	161162709	161162746	5704	0.052631579
chr2	161279486	161279523	5705	0.052631579
chr2	161280295	161280332	5706	0.052631579
chr2	161499416	161499453	5707	0.052631579
chr2	16156706	16156743	5708	0.052631579
chr2	161845395	161845432	5709	0.052631579
chr2	1621506	1621543	5710	0.052631579
chr2	162511140	162511177	5711	0.052631579
chr2	162521825	162521862	5712	0.052631579
chr2	162761622	162761659	5713	0.052631579
chr2	162803163	162803200	5714	0.052631579
chr2	163393096	163393133	5715	0.052631579
chr2	163453931	163453968	5716	0.052631579
chr2	163668432	163668469	5717	0.052631579
chr2	163759443	163759480	5718	0.052631579
chr2	164030420	164030457	5719	0.052631579
chr2	164034461	164034498	5720	0.052631579
chr2	164184540	164184577	5721	0.052631579
chr2	164286732	164286769	5722	0.052631579
chr2	164304947	164304984	5723	0.052631579
chr2	164305578	164305615	5724	0.052631579
chr2	16454587	16454624	5725	0.052631579
chr2	164995862	164995899	5726	0.052631579
chr2	165004985	165005022	5727	0.052631579
chr2	165059169	165059206	5728	0.052631579
chr2	165236934	165236971	5729	0.052631579
chr2	165519503	165519540	5730	0.052631579
chr2	165681480	165681517	5731	0.052631579
chr2	165972152	165972189	5732	0.052631579
chr2	166012170	166012207	5733	0.052631579
chr2	166012723	166012760	5734	0.052631579
chr2	166609937	166609974	5735	0.052631579
chr2	166632416	166632453	5736	0.052631579
chr2	166761391	166761428	5737	0.052631579
chr2	166806109	166806146	5738	0.052631579
chr2	167569130	167569167	5739	0.052631579
chr2	167766932	167766969	5740	0.052631579
chr2	167822912	167822949	5741	0.052631579
chr2	167828468	167828505	5742	0.052631579
chr2	167887911	167887948	5743	0.052631579
chr2	167888701	167888738	5744	0.052631579
chr2	16825674	16825711	5745	0.052631579
chr2	168608846	168608883	5746	0.052631579
chr2	168664631	168664668	5747	0.052631579
chr2	168998351	168998388	5748	0.052631579
chr2	169334844	169334881	5749	0.052631579
chr2	169378935	169378972	5750	0.052631579

chr2	170676240	170676277	5751	0.052631579
chr2	170901670	170901707	5752	0.052631579
chr2	171033056	171033093	5753	0.052631579
chr2	171279916	171279953	5754	0.052631579
chr2	171658652	171658689	5755	0.052631579
chr2	171698852	171698889	5756	0.052631579
chr2	172467760	172467797	5757	0.052631579
chr2	173021929	173021966	5758	0.052631579
chr2	17329213	17329250	5759	0.052631579
chr2	173672579	173672616	5760	0.052631579
chr2	173682480	173682517	5761	0.052631579
chr2	174086511	174086548	5762	0.052631579
chr2	175119844	175119881	5763	0.052631579
chr2	175197604	175197641	5764	0.052631579
chr2	175844777	175844814	5765	0.052631579
chr2	175888833	175888870	5766	0.052631579
chr2	176174770	176174807	5767	0.052631579
chr2	176438037	176438074	5768	0.052631579
chr2	177163205	177163242	5769	0.052631579
chr2	177171723	177171760	5770	0.052631579
chr2	177235488	177235525	5771	0.052631579
chr2	178201679	178201716	5772	0.052631579
chr2	179130006	179130043	5773	0.052631579
chr2	179360888	179360925	5774	0.052631579
chr2	179395419	179395456	5775	0.052631579
chr2	179604961	179604998	5776	0.052631579
chr2	180114439	180114476	5777	0.052631579
chr2	180319375	180319412	5778	0.052631579
chr2	180319470	180319507	5779	0.052631579
chr2	180661912	180661949	5780	0.052631579
chr2	18110351	18110388	5781	0.052631579
chr2	181167806	181167843	5782	0.052631579
chr2	181281116	181281153	5783	0.052631579
chr2	18142991	18143028	5784	0.052631579
chr2	181804968	181805005	5785	0.052631579
chr2	181856504	181856541	5786	0.052631579
chr2	182132976	182133013	5787	0.052631579
chr2	182227113	182227150	5788	0.052631579
chr2	182505976	182506013	5789	0.052631579
chr2	182916202	182916239	5790	0.052631579
chr2	183290183	183290220	5791	0.052631579
chr2	183301583	183301620	5792	0.052631579
chr2	183305475	183305512	5793	0.052631579
chr2	183564215	183564252	5794	0.052631579
chr2	183566432	183566469	5795	0.052631579
chr2	183762232	183762269	5796	0.052631579
chr2	184431627	184431664	5797	0.052631579
chr2	184435669	184435706	5798	0.052631579
chr2	184435976	184436013	5799	0.052631579
chr2	184680074	184680111	5800	0.052631579

chr2	184831268	184831305	5801	0.052631579
chr2	185286189	185286226	5802	0.052631579
chr2	185308250	185308287	5803	0.052631579
chr2	185793150	185793187	5804	0.052631579
chr2	186294740	186294777	5805	0.052631579
chr2	186387317	186387354	5806	0.052631579
chr2	187056030	187056067	5807	0.052631579
chr2	187056409	187056446	5808	0.052631579
chr2	187438777	187438814	5809	0.052631579
chr2	187977247	187977284	5810	0.052631579
chr2	187988638	187988675	5811	0.052631579
chr2	18833183	18833220	5812	0.052631579
chr2	18960218	18960255	5813	0.052631579
chr2	189635862	189635899	5814	0.052631579
chr2	189771492	189771529	5815	0.052631579
chr2	189771576	189771613	5816	0.052631579
chr2	190106252	190106289	5817	0.052631579
chr2	190518936	190518973	5818	0.052631579
chr2	190897634	190897671	5819	0.052631579
chr2	19096476	19096513	5820	0.052631579
chr2	191062741	191062778	5821	0.052631579
chr2	191244308	191244345	5822	0.052631579
chr2	191316852	191316889	5823	0.052631579
chr2	191430877	191430914	5824	0.052631579
chr2	191430966	191431003	5825	0.052631579
chr2	191982583	191982620	5826	0.052631579
chr2	191987762	191987799	5827	0.052631579
chr2	192440888	192440925	5828	0.052631579
chr2	19319092	19319129	5829	0.052631579
chr2	193432744	193432781	5830	0.052631579
chr2	19418292	19418329	5831	0.052631579
chr2	194652928	194652965	5832	0.052631579
chr2	194702872	194702909	5833	0.052631579
chr2	194842789	194842826	5834	0.052631579
chr2	195160985	195161022	5835	0.052631579
chr2	195161197	195161234	5836	0.052631579
chr2	195283251	195283288	5837	0.052631579
chr2	195575710	195575747	5838	0.052631579
chr2	195577920	195577957	5839	0.052631579
chr2	195721153	195721190	5840	0.052631579
chr2	195721823	195721860	5841	0.052631579
chr2	196379895	196379932	5842	0.052631579
chr2	196437272	196437309	5843	0.052631579
chr2	19674613	19674650	5844	0.052631579
chr2	196934170	196934207	5845	0.052631579
chr2	197212683	197212720	5846	0.052631579
chr2	197741423	197741460	5847	0.052631579
chr2	197976692	197976729	5848	0.052631579
chr2	198486053	198486090	5849	0.052631579
chr2	198879430	198879467	5850	0.052631579

chr2	198947067	198947104	5851	0.052631579
chr2	199098532	199098569	5852	0.052631579
chr2	199226594	199226631	5853	0.052631579
chr2	199775949	199775986	5854	0.052631579
chr2	199843197	199843234	5855	0.052631579
chr2	199872487	199872524	5856	0.052631579
chr2	199880759	199880796	5857	0.052631579
chr2	199883311	199883348	5858	0.052631579
chr2	200144021	200144058	5859	0.052631579
chr2	200154902	200154939	5860	0.052631579
chr2	200695332	200695369	5861	0.052631579
chr2	200785925	200785962	5862	0.052631579
chr2	200926160	200926197	5863	0.052631579
chr2	201099788	201099825	5864	0.052631579
chr2	201204799	201204836	5865	0.052631579
chr2	201396787	201396824	5866	0.052631579
chr2	201959493	201959530	5867	0.052631579
chr2	20240064	20240101	5868	0.052631579
chr2	20240756	20240793	5869	0.052631579
chr2	202411843	202411880	5870	0.052631579
chr2	202601468	202601505	5871	0.052631579
chr2	202658004	202658041	5872	0.052631579
chr2	202663695	202663732	5873	0.052631579
chr2	202686696	202686733	5874	0.052631579
chr2	203016910	203016947	5875	0.052631579
chr2	203267525	203267562	5876	0.052631579
chr2	203824944	203824981	5877	0.052631579
chr2	203883970	203884007	5878	0.052631579
chr2	204223704	204223741	5879	0.052631579
chr2	204384870	204384907	5880	0.052631579
chr2	204471326	204471363	5881	0.052631579
chr2	204695226	204695263	5882	0.052631579
chr2	204806675	204806712	5883	0.052631579
chr2	205027165	205027202	5884	0.052631579
chr2	205073080	205073117	5885	0.052631579
chr2	20511028	20511065	5886	0.052631579
chr2	205124526	205124563	5887	0.052631579
chr2	20533403	20533440	5888	0.052631579
chr2	205598088	205598125	5889	0.052631579
chr2	206048434	206048471	5890	0.052631579
chr2	206300955	206300992	5891	0.052631579
chr2	206485941	206485978	5892	0.052631579
chr2	206582331	206582368	5893	0.052631579
chr2	206632546	206632583	5894	0.052631579
chr2	206778298	206778335	5895	0.052631579
chr2	207213776	207213813	5896	0.052631579
chr2	207313746	207313783	5897	0.052631579
chr2	207363005	207363042	5898	0.052631579
chr2	207824737	207824774	5899	0.052631579
chr2	208122474	208122511	5900	0.052631579

chr2	208178750	208178787	5901	0.052631579
chr2	208227917	208227954	5902	0.052631579
chr2	208304625	208304662	5903	0.052631579
chr2	208340546	208340583	5904	0.052631579
chr2	208871676	208871713	5905	0.052631579
chr2	209051462	209051499	5906	0.052631579
chr2	209240170	209240207	5907	0.052631579
chr2	209463298	209463335	5908	0.052631579
chr2	209607013	209607050	5909	0.052631579
chr2	210213701	210213738	5910	0.052631579
chr2	210506926	210506963	5911	0.052631579
chr2	210533142	210533179	5912	0.052631579
chr2	21101855	21101892	5913	0.052631579
chr2	211305067	211305104	5914	0.052631579
chr2	212564632	212564669	5915	0.052631579
chr2	212564762	212564799	5916	0.052631579
chr2	21269644	21269681	5917	0.052631579
chr2	212879439	212879476	5918	0.052631579
chr2	213042696	213042733	5919	0.052631579
chr2	213043291	213043328	5920	0.052631579
chr2	213723604	213723641	5921	0.052631579
chr2	214855534	214855571	5922	0.052631579
chr2	215148364	215148401	5923	0.052631579
chr2	215217832	215217869	5924	0.052631579
chr2	215899892	215899929	5925	0.052631579
chr2	215949511	215949548	5926	0.052631579
chr2	215991033	215991070	5927	0.052631579
chr2	215991129	215991166	5928	0.052631579
chr2	216213928	216213965	5929	0.052631579
chr2	21656579	21656616	5930	0.052631579
chr2	216624529	216624566	5931	0.052631579
chr2	216625211	216625248	5932	0.052631579
chr2	216777967	216778004	5933	0.052631579
chr2	216823551	216823588	5934	0.052631579
chr2	216870299	216870336	5935	0.052631579
chr2	217798586	217798623	5936	0.052631579
chr2	217944430	217944467	5937	0.052631579
chr2	218107241	218107278	5938	0.052631579
chr2	2183888	2183925	5939	0.052631579
chr2	219251258	219251295	5940	0.052631579
chr2	219252037	219252074	5941	0.052631579
chr2	219638486	219638523	5942	0.052631579
chr2	219823872	219823909	5943	0.052631579
chr2	220596647	220596684	5944	0.052631579
chr2	220597373	220597410	5945	0.052631579
chr2	221482786	221482823	5946	0.052631579
chr2	221712812	221712849	5947	0.052631579
chr2	222005421	222005458	5948	0.052631579
chr2	222005567	222005604	5949	0.052631579
chr2	222060667	222060704	5950	0.052631579

chr2	222656334	222656371	5951	0.052631579
chr2	222686767	222686804	5952	0.052631579
chr2	223385570	223385607	5953	0.052631579
chr2	223465464	223465501	5954	0.052631579
chr2	22362621	22362658	5955	0.052631579
chr2	223836488	223836525	5956	0.052631579
chr2	223884088	223884125	5957	0.052631579
chr2	223894237	223894274	5958	0.052631579
chr2	224009479	224009516	5959	0.052631579
chr2	224508893	224508930	5960	0.052631579
chr2	224747368	224747405	5961	0.052631579
chr2	224885024	224885061	5962	0.052631579
chr2	224943606	224943643	5963	0.052631579
chr2	226086567	226086604	5964	0.052631579
chr2	226365690	226365727	5965	0.052631579
chr2	226367838	226367875	5966	0.052631579
chr2	226411807	226411844	5967	0.052631579
chr2	226627226	226627263	5968	0.052631579
chr2	227020310	227020347	5969	0.052631579
chr2	227170779	227170816	5970	0.052631579
chr2	227369387	227369424	5971	0.052631579
chr2	227370956	227370993	5972	0.052631579
chr2	227413307	227413344	5973	0.052631579
chr2	227652398	227652435	5974	0.052631579
chr2	227653156	227653193	5975	0.052631579
chr2	227806525	227806562	5976	0.052631579
chr2	227836695	227836732	5977	0.052631579
chr2	227906189	227906226	5978	0.052631579
chr2	228028033	228028070	5979	0.052631579
chr2	228544489	228544526	5980	0.052631579
chr2	228902288	228902325	5981	0.052631579
chr2	229362880	229362917	5982	0.052631579
chr2	229990634	229990671	5983	0.052631579
chr2	229991207	229991244	5984	0.052631579
chr2	230049884	230049921	5985	0.052631579
chr2	230365997	230366034	5986	0.052631579
chr2	230785932	230785969	5987	0.052631579
chr2	230969179	230969216	5988	0.052631579
chr2	231040076	231040113	5989	0.052631579
chr2	232025636	232025673	5990	0.052631579
chr2	232215687	232215724	5991	0.052631579
chr2	232643293	232643330	5992	0.052631579
chr2	233106289	233106326	5993	0.052631579
chr2	233407123	233407160	5994	0.052631579
chr2	234024536	234024573	5995	0.052631579
chr2	234130308	234130345	5996	0.052631579
chr2	23426952	23426989	5997	0.052631579
chr2	23433406	23433443	5998	0.052631579
chr2	234657800	234657837	5999	0.052631579
chr2	234797002	234797039	6000	0.052631579

chr2	234803185	234803222	6001	0.052631579
chr2	235007395	235007432	6002	0.052631579
chr2	23513078	23513115	6003	0.052631579
chr2	236087594	236087631	6004	0.052631579
chr2	236108322	236108359	6005	0.052631579
chr2	236294346	236294383	6006	0.052631579
chr2	236353949	236353986	6007	0.052631579
chr2	236692111	236692148	6008	0.052631579
chr2	236728958	236728995	6009	0.052631579
chr2	236907236	236907273	6010	0.052631579
chr2	236951417	236951454	6011	0.052631579
chr2	237031871	237031908	6012	0.052631579
chr2	237154247	237154284	6013	0.052631579
chr2	23715585	23715622	6014	0.052631579
chr2	23718786	23718823	6015	0.052631579
chr2	237250023	237250060	6016	0.052631579
chr2	237579784	237579821	6017	0.052631579
chr2	23760253	23760290	6018	0.052631579
chr2	237909513	237909550	6019	0.052631579
chr2	237945338	237945375	6020	0.052631579
chr2	23828693	23828730	6021	0.052631579
chr2	238375155	238375192	6022	0.052631579
chr2	23841796	23841833	6023	0.052631579
chr2	23848820	23848857	6024	0.052631579
chr2	238635539	238635576	6025	0.052631579
chr2	238888461	238888498	6026	0.052631579
chr2	238985695	238985732	6027	0.052631579
chr2	239098784	239098821	6028	0.052631579
chr2	239118334	239118371	6029	0.052631579
chr2	239600682	239600719	6030	0.052631579
chr2	239677914	239677951	6031	0.052631579
chr2	239915147	239915184	6032	0.052631579
chr2	239933516	239933553	6033	0.052631579
chr2	240073485	240073522	6034	0.052631579
chr2	240074298	240074335	6035	0.052631579
chr2	240938281	240938318	6036	0.052631579
chr2	241383972	241384009	6037	0.052631579
chr2	241384791	241384828	6038	0.052631579
chr2	24144776	24144813	6039	0.052631579
chr2	241576184	241576221	6040	0.052631579
chr2	241759113	241759150	6041	0.052631579
chr2	241827704	241827741	6042	0.052631579
chr2	241890945	241890982	6043	0.052631579
chr2	241901901	241901938	6044	0.052631579
chr2	24585748	24585785	6045	0.052631579
chr2	24695826	24695863	6046	0.052631579
chr2	25672451	25672488	6047	0.052631579
chr2	2590129	2590166	6048	0.052631579
chr2	25904278	25904315	6049	0.052631579
chr2	26110725	26110762	6050	0.052631579

chr2	26571321	26571358	6051	0.052631579
chr2	27162345	27162382	6052	0.052631579
chr2	27348063	27348100	6053	0.052631579
chr2	27511559	27511596	6054	0.052631579
chr2	27569119	27569156	6055	0.052631579
chr2	27662284	27662321	6056	0.052631579
chr2	2789898	2789935	6057	0.052631579
chr2	28314007	28314044	6058	0.052631579
chr2	28685111	28685148	6059	0.052631579
chr2	28945909	28945946	6060	0.052631579
chr2	28974243	28974280	6061	0.052631579
chr2	29015024	29015061	6062	0.052631579
chr2	29648580	29648617	6063	0.052631579
chr2	29649148	29649185	6064	0.052631579
chr2	29785685	29785722	6065	0.052631579
chr2	29851315	29851352	6066	0.052631579
chr2	30044284	30044321	6067	0.052631579
chr2	30115972	30116009	6068	0.052631579
chr2	30447564	30447601	6069	0.052631579
chr2	30479698	30479735	6070	0.052631579
chr2	30554726	30554763	6071	0.052631579
chr2	30653972	30654009	6072	0.052631579
chr2	30966501	30966538	6073	0.052631579
chr2	31506163	31506200	6074	0.052631579
chr2	31525	31562	6075	0.052631579
chr2	31733047	31733084	6076	0.052631579
chr2	31994683	31994720	6077	0.052631579
chr2	32260097	32260134	6078	0.052631579
chr2	32376813	32376850	6079	0.052631579
chr2	32534279	32534316	6080	0.052631579
chr2	32534478	32534515	6081	0.052631579
chr2	32667283	32667320	6082	0.052631579
chr2	33151604	33151641	6083	0.052631579
chr2	33517924	33517961	6084	0.052631579
chr2	34041645	34041682	6085	0.052631579
chr2	34216813	34216850	6086	0.052631579
chr2	34318264	34318301	6087	0.052631579
chr2	34439414	34439451	6088	0.052631579
chr2	35555	35592	6089	0.052631579
chr2	35938247	35938284	6090	0.052631579
chr2	36661983	36662020	6091	0.052631579
chr2	36844570	36844607	6092	0.052631579
chr2	36928857	36928894	6093	0.052631579
chr2	37149473	37149510	6094	0.052631579
chr2	37475095	37475132	6095	0.052631579
chr2	37495387	37495424	6096	0.052631579
chr2	37641962	37641999	6097	0.052631579
chr2	37855105	37855142	6098	0.052631579
chr2	37875298	37875335	6099	0.052631579
chr2	38653101	38653138	6100	0.052631579

chr2	39057328	39057365	6101	0.052631579
chr2	39462883	39462920	6102	0.052631579
chr2	40208103	40208140	6103	0.052631579
chr2	40250191	40250228	6104	0.052631579
chr2	40367822	40367859	6105	0.052631579
chr2	40433596	40433633	6106	0.052631579
chr2	40434341	40434378	6107	0.052631579
chr2	40995961	40995998	6108	0.052631579
chr2	41002492	41002529	6109	0.052631579
chr2	41602979	41603016	6110	0.052631579
chr2	41628322	41628359	6111	0.052631579
chr2	41629090	41629127	6112	0.052631579
chr2	41645137	41645174	6113	0.052631579
chr2	42196553	42196590	6114	0.052631579
chr2	42361468	42361505	6115	0.052631579
chr2	43040706	43040743	6116	0.052631579
chr2	43156026	43156063	6117	0.052631579
chr2	43478626	43478663	6118	0.052631579
chr2	43497366	43497403	6119	0.052631579
chr2	43565946	43565983	6120	0.052631579
chr2	43652480	43652517	6121	0.052631579
chr2	43775290	43775327	6122	0.052631579
chr2	44184312	44184349	6123	0.052631579
chr2	44289843	44289880	6124	0.052631579
chr2	45023350	45023387	6125	0.052631579
chr2	45266532	45266569	6126	0.052631579
chr2	45400262	45400299	6127	0.052631579
chr2	45402499	45402536	6128	0.052631579
chr2	45574882	45574919	6129	0.052631579
chr2	46062873	46062910	6130	0.052631579
chr2	46063694	46063731	6131	0.052631579
chr2	46084683	46084720	6132	0.052631579
chr2	46333234	46333271	6133	0.052631579
chr2	46417693	46417730	6134	0.052631579
chr2	46427521	46427558	6135	0.052631579
chr2	4652665	4652702	6136	0.052631579
chr2	46543959	46543996	6137	0.052631579
chr2	46779508	46779545	6138	0.052631579
chr2	46894069	46894106	6139	0.052631579
chr2	4692819	4692856	6140	0.052631579
chr2	47069913	47069950	6141	0.052631579
chr2	47232821	47232858	6142	0.052631579
chr2	47279449	47279486	6143	0.052631579
chr2	47452783	47452820	6144	0.052631579
chr2	48033091	48033128	6145	0.052631579
chr2	48485459	48485496	6146	0.052631579
chr2	48542138	48542175	6147	0.052631579
chr2	4867991	4868028	6148	0.052631579
chr2	4904766	4904803	6149	0.052631579
chr2	49194483	49194520	6150	0.052631579

chr2	4930183	4930220	6151	0.052631579
chr2	50155625	50155662	6152	0.052631579
chr2	50317521	50317558	6153	0.052631579
chr2	50684186	50684223	6154	0.052631579
chr2	50970091	50970128	6155	0.052631579
chr2	51833829	51833866	6156	0.052631579
chr2	51833928	51833965	6157	0.052631579
chr2	51860038	51860075	6158	0.052631579
chr2	52547461	52547498	6159	0.052631579
chr2	52662856	52662893	6160	0.052631579
chr2	52949854	52949891	6161	0.052631579
chr2	53306471	53306508	6162	0.052631579
chr2	53517534	53517571	6163	0.052631579
chr2	53618305	53618342	6164	0.052631579
chr2	53704434	53704471	6165	0.052631579
chr2	53705211	53705248	6166	0.052631579
chr2	53797349	53797386	6167	0.052631579
chr2	53944909	53944946	6168	0.052631579
chr2	5432317	5432354	6169	0.052631579
chr2	5432467	5432504	6170	0.052631579
chr2	54353241	54353278	6171	0.052631579
chr2	54411122	54411159	6172	0.052631579
chr2	54449089	54449126	6173	0.052631579
chr2	54492086	54492123	6174	0.052631579
chr2	54646535	54646572	6175	0.052631579
chr2	5467582	5467619	6176	0.052631579
chr2	54723628	54723665	6177	0.052631579
chr2	54784429	54784466	6178	0.052631579
chr2	5526218	5526255	6179	0.052631579
chr2	55317645	55317682	6180	0.052631579
chr2	55376535	55376572	6181	0.052631579
chr2	55511148	55511185	6182	0.052631579
chr2	56405551	56405588	6183	0.052631579
chr2	56464029	56464066	6184	0.052631579
chr2	56990864	56990901	6185	0.052631579
chr2	56991435	56991472	6186	0.052631579
chr2	57295636	57295673	6187	0.052631579
chr2	5799666	5799703	6188	0.052631579
chr2	5799758	5799795	6189	0.052631579
chr2	58134341	58134378	6190	0.052631579
chr2	58593301	58593338	6191	0.052631579
chr2	58594049	58594086	6192	0.052631579
chr2	59034969	59035006	6193	0.052631579
chr2	59394830	59394867	6194	0.052631579
chr2	5951679	5951716	6195	0.052631579
chr2	59851408	59851445	6196	0.052631579
chr2	59852226	59852263	6197	0.052631579
chr2	59900883	59900920	6198	0.052631579
chr2	5990772	5990809	6199	0.052631579
chr2	60203485	60203522	6200	0.052631579

chr2	60354028	60354065	6201	0.052631579
chr2	6039610	6039647	6202	0.052631579
chr2	61346096	61346133	6203	0.052631579
chr2	61958060	61958097	6204	0.052631579
chr2	61998964	61999001	6205	0.052631579
chr2	6213043	6213080	6206	0.052631579
chr2	62243165	62243202	6207	0.052631579
chr2	62504362	62504399	6208	0.052631579
chr2	62743350	62743387	6209	0.052631579
chr2	63368640	63368677	6210	0.052631579
chr2	64730357	64730394	6211	0.052631579
chr2	64893268	64893305	6212	0.052631579
chr2	6503194	6503231	6213	0.052631579
chr2	65169054	65169091	6214	0.052631579
chr2	65757819	65757856	6215	0.052631579
chr2	66381704	66381741	6216	0.052631579
chr2	6663349	6663386	6217	0.052631579
chr2	67317438	67317475	6218	0.052631579
chr2	67910149	67910186	6219	0.052631579
chr2	68157536	68157573	6220	0.052631579
chr2	69208099	69208136	6221	0.052631579
chr2	69227347	69227384	6222	0.052631579
chr2	69372289	69372326	6223	0.052631579
chr2	6953260	6953297	6224	0.052631579
chr2	7063079	7063116	6225	0.052631579
chr2	71667943	71667980	6226	0.052631579
chr2	71786044	71786081	6227	0.052631579
chr2	71871147	71871184	6228	0.052631579
chr2	71871433	71871470	6229	0.052631579
chr2	7239296	7239333	6230	0.052631579
chr2	7239912	7239949	6231	0.052631579
chr2	72570600	72570637	6232	0.052631579
chr2	73125102	73125139	6233	0.052631579
chr2	73125999	73126036	6234	0.052631579
chr2	7314640	7314677	6235	0.052631579
chr2	73364537	73364574	6236	0.052631579
chr2	73965903	73965940	6237	0.052631579
chr2	74030861	74030898	6238	0.052631579
chr2	74155824	74155861	6239	0.052631579
chr2	74451992	74452029	6240	0.052631579
chr2	74539279	74539316	6241	0.052631579
chr2	74540272	74540309	6242	0.052631579
chr2	745417	745454	6243	0.052631579
chr2	74759637	74759674	6244	0.052631579
chr2	74772521	74772558	6245	0.052631579
chr2	75742965	75743002	6246	0.052631579
chr2	76349378	76349415	6247	0.052631579
chr2	76349580	76349617	6248	0.052631579
chr2	76666602	76666639	6249	0.052631579
chr2	76997559	76997596	6250	0.052631579

chr2	7700547	7700584	6251	0.052631579
chr2	77223213	77223250	6252	0.052631579
chr2	77535240	77535277	6253	0.052631579
chr2	7792008	7792045	6254	0.052631579
chr2	7792742	7792779	6255	0.052631579
chr2	77973608	77973645	6256	0.052631579
chr2	78551416	78551453	6257	0.052631579
chr2	7860367	7860404	6258	0.052631579
chr2	79168684	79168721	6259	0.052631579
chr2	81057962	81057999	6260	0.052631579
chr2	81628210	81628247	6261	0.052631579
chr2	81694300	81694337	6262	0.052631579
chr2	81809442	81809479	6263	0.052631579
chr2	82258193	82258230	6264	0.052631579
chr2	82619874	82619911	6265	0.052631579
chr2	83217260	83217297	6266	0.052631579
chr2	83612725	83612762	6267	0.052631579
chr2	83940065	83940102	6268	0.052631579
chr2	84034853	84034890	6269	0.052631579
chr2	8408307	8408344	6270	0.052631579
chr2	84223493	84223530	6271	0.052631579
chr2	84498338	84498375	6272	0.052631579
chr2	85321982	85322019	6273	0.052631579
chr2	85384598	85384635	6274	0.052631579
chr2	86117805	86117842	6275	0.052631579
chr2	86313288	86313325	6276	0.052631579
chr2	86418779	86418816	6277	0.052631579
chr2	87827405	87827442	6278	0.052631579
chr2	88377024	88377061	6279	0.052631579
chr2	89032857	89032894	6280	0.052631579
chr2	89885916	89885953	6281	0.052631579
chr2	89886710	89886747	6282	0.052631579
chr2	91042396	91042433	6283	0.052631579
chr2	91332827	91332864	6284	0.052631579
chr2	91395064	91395101	6285	0.052631579
chr2	9413785	9413822	6286	0.052631579
chr2	9463271	9463308	6287	0.052631579
chr2	95312327	95312364	6288	0.052631579
chr2	95464275	95464312	6289	0.052631579
chr2	9559082	9559119	6290	0.052631579
chr2	96308776	96308813	6291	0.052631579
chr2	96313025	96313062	6292	0.052631579
chr2	96695429	96695466	6293	0.052631579
chr2	96841666	96841703	6294	0.052631579
chr2	96870132	96870169	6295	0.052631579
chr2	97014939	97014976	6296	0.052631579
chr2	97194934	97194971	6297	0.052631579
chr2	98616503	98616540	6298	0.052631579
chr2	99328057	99328094	6299	0.052631579
chr2	99361160	99361197	6300	0.052631579

chr2	99457340	99457377	6301	0.052631579
chr2	99462759	99462796	6302	0.052631579
chr2	100226515	100226554	6303	0.075
chr2	100993016	100993055	6304	0.075
chr2	10100311	10100350	6305	0.075
chr2	101469498	101469537	6306	0.075
chr2	101541736	101541775	6307	0.075
chr2	103654191	103654230	6308	0.075
chr2	103807080	103807119	6309	0.075
chr2	104232830	104232869	6310	0.075
chr2	10517231	10517270	6311	0.075
chr2	109399882	109399921	6312	0.075
chr2	109420998	109421037	6313	0.075
chr2	11107975	11108014	6314	0.075
chr2	111542946	111542985	6315	0.075
chr2	111613560	111613599	6316	0.075
chr2	111699802	111699841	6317	0.075
chr2	111721574	111721613	6318	0.075
chr2	112011235	112011274	6319	0.075
chr2	112961352	112961391	6320	0.075
chr2	115738361	115738400	6321	0.075
chr2	11606877	11606916	6322	0.075
chr2	117534220	117534259	6323	0.075
chr2	119911746	119911785	6324	0.075
chr2	120457399	120457438	6325	0.075
chr2	121729414	121729453	6326	0.075
chr2	122121677	122121716	6327	0.075
chr2	122445185	122445224	6328	0.075
chr2	123343440	123343479	6329	0.075
chr2	124169983	124170022	6330	0.075
chr2	124353140	124353179	6331	0.075
chr2	125113852	125113891	6332	0.075
chr2	125276734	125276773	6333	0.075
chr2	126704349	126704388	6334	0.075
chr2	128135765	128135804	6335	0.075
chr2	128238876	128238915	6336	0.075
chr2	128859484	128859523	6337	0.075
chr2	1295482	1295521	6338	0.075
chr2	130811547	130811586	6339	0.075
chr2	131930036	131930075	6340	0.075
chr2	132969058	132969097	6341	0.075
chr2	134258062	134258101	6342	0.075
chr2	134329657	134329696	6343	0.075
chr2	135161626	135161665	6344	0.075
chr2	13570133	13570172	6345	0.075
chr2	136286638	136286677	6346	0.075
chr2	136718420	136718459	6347	0.075
chr2	136835451	136835490	6348	0.075
chr2	137248729	137248768	6349	0.075
chr2	137528673	137528712	6350	0.075

chr2	138667635	138667674	6351	0.075
chr2	139646104	139646143	6352	0.075
chr2	141996907	141996946	6353	0.075
chr2	14210685	14210724	6354	0.075
chr2	144347543	144347582	6355	0.075
chr2	144861278	144861317	6356	0.075
chr2	144952362	144952401	6357	0.075
chr2	145620780	145620819	6358	0.075
chr2	145660639	145660678	6359	0.075
chr2	147898628	147898667	6360	0.075
chr2	1481459	1481498	6361	0.075
chr2	149728489	149728528	6362	0.075
chr2	152195217	152195256	6363	0.075
chr2	152220492	152220531	6364	0.075
chr2	152711969	152712008	6365	0.075
chr2	152800649	152800688	6366	0.075
chr2	155977743	155977782	6367	0.075
chr2	157315456	157315495	6368	0.075
chr2	157621005	157621044	6369	0.075
chr2	157786870	157786909	6370	0.075
chr2	158194602	158194641	6371	0.075
chr2	158288554	158288593	6372	0.075
chr2	158590054	158590093	6373	0.075
chr2	159409536	159409575	6374	0.075
chr2	159744813	159744852	6375	0.075
chr2	160244434	160244473	6376	0.075
chr2	160497510	160497549	6377	0.075
chr2	160621863	160621902	6378	0.075
chr2	161473251	161473290	6379	0.075
chr2	163300950	163300989	6380	0.075
chr2	164329859	164329898	6381	0.075
chr2	164555510	164555549	6382	0.075
chr2	164915979	164916018	6383	0.075
chr2	16548425	16548464	6384	0.075
chr2	16597482	16597521	6385	0.075
chr2	166058979	166059018	6386	0.075
chr2	166141296	166141335	6387	0.075
chr2	166385617	166385656	6388	0.075
chr2	168503861	168503900	6389	0.075
chr2	170201707	170201746	6390	0.075
chr2	170939022	170939061	6391	0.075
chr2	17151374	17151413	6392	0.075
chr2	17169157	17169196	6393	0.075
chr2	171892412	171892451	6394	0.075
chr2	172143551	172143590	6395	0.075
chr2	172666976	172667015	6396	0.075
chr2	173516892	173516931	6397	0.075
chr2	174316243	174316282	6398	0.075
chr2	175332523	175332562	6399	0.075
chr2	175374543	175374582	6400	0.075

chr2	175410810	175410849	6401	0.075
chr2	175754911	175754950	6402	0.075
chr2	176110854	176110893	6403	0.075
chr2	176288340	176288379	6404	0.075
chr2	176520640	176520679	6405	0.075
chr2	176663310	176663349	6406	0.075
chr2	176774141	176774180	6407	0.075
chr2	177064590	177064629	6408	0.075
chr2	177147466	177147505	6409	0.075
chr2	177792383	177792422	6410	0.075
chr2	178191250	178191289	6411	0.075
chr2	178466479	178466518	6412	0.075
chr2	17895374	17895413	6413	0.075
chr2	179372661	179372700	6414	0.075
chr2	179686118	179686157	6415	0.075
chr2	18355034	18355073	6416	0.075
chr2	18687095	18687134	6417	0.075
chr2	188794460	188794499	6418	0.075
chr2	190245637	190245676	6419	0.075
chr2	19024690	19024729	6420	0.075
chr2	1908489	1908528	6421	0.075
chr2	191042995	191043034	6422	0.075
chr2	195886613	195886652	6423	0.075
chr2	196813455	196813494	6424	0.075
chr2	197930508	197930547	6425	0.075
chr2	198781992	198782031	6426	0.075
chr2	200903658	200903697	6427	0.075
chr2	201272350	201272389	6428	0.075
chr2	20176517	20176556	6429	0.075
chr2	20288269	20288308	6430	0.075
chr2	203684652	203684691	6431	0.075
chr2	204361533	204361572	6432	0.075
chr2	204363206	204363245	6433	0.075
chr2	204718712	204718751	6434	0.075
chr2	204983764	204983803	6435	0.075
chr2	205586176	205586215	6436	0.075
chr2	206164071	206164110	6437	0.075
chr2	206186223	206186262	6438	0.075
chr2	206340753	206340792	6439	0.075
chr2	206351059	206351098	6440	0.075
chr2	206407083	206407122	6441	0.075
chr2	206430707	206430746	6442	0.075
chr2	207618566	207618605	6443	0.075
chr2	207754485	207754524	6444	0.075
chr2	208619099	208619138	6445	0.075
chr2	208902120	208902159	6446	0.075
chr2	211652840	211652879	6447	0.075
chr2	212169573	212169612	6448	0.075
chr2	212352321	212352360	6449	0.075
chr2	212375884	212375923	6450	0.075

chr2	213204467	213204506	6451	0.075
chr2	214073170	214073209	6452	0.075
chr2	21566691	21566730	6453	0.075
chr2	215934462	215934501	6454	0.075
chr2	215938543	215938582	6455	0.075
chr2	215959657	215959696	6456	0.075
chr2	215982916	215982955	6457	0.075
chr2	216757237	216757276	6458	0.075
chr2	216946297	216946336	6459	0.075
chr2	217206830	217206869	6460	0.075
chr2	218284444	218284483	6461	0.075
chr2	218501966	218502005	6462	0.075
chr2	218847209	218847248	6463	0.075
chr2	219120638	219120677	6464	0.075
chr2	219183119	219183158	6465	0.075
chr2	219535103	219535142	6466	0.075
chr2	219631027	219631066	6467	0.075
chr2	219870857	219870896	6468	0.075
chr2	219871209	219871248	6469	0.075
chr2	220112501	220112540	6470	0.075
chr2	220112748	220112787	6471	0.075
chr2	22012186	22012225	6472	0.075
chr2	22130629	22130668	6473	0.075
chr2	222633590	222633629	6474	0.075
chr2	222870038	222870077	6475	0.075
chr2	223140684	223140723	6476	0.075
chr2	225361343	225361382	6477	0.075
chr2	227920245	227920284	6478	0.075
chr2	22918977	22919016	6479	0.075
chr2	229761908	229761947	6480	0.075
chr2	230374357	230374396	6481	0.075
chr2	230898544	230898583	6482	0.075
chr2	231744425	231744464	6483	0.075
chr2	23180914	23180953	6484	0.075
chr2	231910817	231910856	6485	0.075
chr2	232257767	232257806	6486	0.075
chr2	234009038	234009077	6487	0.075
chr2	234625128	234625167	6488	0.075
chr2	235624788	235624827	6489	0.075
chr2	23588310	23588349	6490	0.075
chr2	236111955	236111994	6491	0.075
chr2	236732258	236732297	6492	0.075
chr2	237805363	237805402	6493	0.075
chr2	238305923	238305962	6494	0.075
chr2	238498971	238499010	6495	0.075
chr2	239147627	239147666	6496	0.075
chr2	239240169	239240208	6497	0.075
chr2	239305275	239305314	6498	0.075
chr2	240767573	240767612	6499	0.075
chr2	241114352	241114391	6500	0.075

chr2	241160171	241160210	6501	0.075
chr2	24295477	24295516	6502	0.075
chr2	24842636	24842675	6503	0.075
chr2	25140806	25140845	6504	0.075
chr2	25237194	25237233	6505	0.075
chr2	26539891	26539930	6506	0.075
chr2	26603894	26603933	6507	0.075
chr2	27225768	27225807	6508	0.075
chr2	27331735	27331774	6509	0.075
chr2	27462593	27462632	6510	0.075
chr2	27505257	27505296	6511	0.075
chr2	27557588	27557627	6512	0.075
chr2	2865225	2865264	6513	0.075
chr2	28828478	28828517	6514	0.075
chr2	29344972	29345011	6515	0.075
chr2	29476343	29476382	6516	0.075
chr2	29657866	29657905	6517	0.075
chr2	29795437	29795476	6518	0.075
chr2	30439462	30439501	6519	0.075
chr2	30813720	30813759	6520	0.075
chr2	31020051	31020090	6521	0.075
chr2	32889712	32889751	6522	0.075
chr2	3336959	3336998	6523	0.075
chr2	33658339	33658378	6524	0.075
chr2	3390756	3390795	6525	0.075
chr2	35573564	35573603	6526	0.075
chr2	36105456	36105495	6527	0.075
chr2	396078	396117	6528	0.075
chr2	3988927	3988966	6529	0.075
chr2	40470881	40470920	6530	0.075
chr2	41949542	41949581	6531	0.075
chr2	42082598	42082637	6532	0.075
chr2	42296341	42296380	6533	0.075
chr2	42984771	42984810	6534	0.075
chr2	43081815	43081854	6535	0.075
chr2	43236836	43236875	6536	0.075
chr2	43305998	43306037	6537	0.075
chr2	43307241	43307280	6538	0.075
chr2	44194787	44194826	6539	0.075
chr2	44761189	44761228	6540	0.075
chr2	45028719	45028758	6541	0.075
chr2	46338422	46338461	6542	0.075
chr2	46539818	46539857	6543	0.075
chr2	46989865	46989904	6544	0.075
chr2	47996368	47996407	6545	0.075
chr2	48362588	48362627	6546	0.075
chr2	48455761	48455800	6547	0.075
chr2	48459835	48459874	6548	0.075
chr2	49115708	49115747	6549	0.075
chr2	49190543	49190582	6550	0.075

chr2	49735984	49736023	6551	0.075
chr2	51016872	51016911	6552	0.075
chr2	51494875	51494914	6553	0.075
chr2	51730012	51730051	6554	0.075
chr2	53751674	53751713	6555	0.075
chr2	53893617	53893656	6556	0.075
chr2	54038548	54038587	6557	0.075
chr2	54615519	54615558	6558	0.075
chr2	54711640	54711679	6559	0.075
chr2	55012986	55013025	6560	0.075
chr2	55389543	55389582	6561	0.075
chr2	58188748	58188787	6562	0.075
chr2	58775489	58775528	6563	0.075
chr2	58797873	58797912	6564	0.075
chr2	5927135	5927174	6565	0.075
chr2	59534218	59534257	6566	0.075
chr2	59670222	59670261	6567	0.075
chr2	59989651	59989690	6568	0.075
chr2	60445198	60445237	6569	0.075
chr2	61617312	61617351	6570	0.075
chr2	63076810	63076849	6571	0.075
chr2	63147904	63147943	6572	0.075
chr2	63567629	63567668	6573	0.075
chr2	65332388	65332427	6574	0.075
chr2	65350145	65350184	6575	0.075
chr2	65374283	65374322	6576	0.075
chr2	65644845	65644884	6577	0.075
chr2	6578462	6578501	6578	0.075
chr2	66648178	66648217	6579	0.075
chr2	66957884	66957923	6580	0.075
chr2	67529758	67529797	6581	0.075
chr2	68399909	68399948	6582	0.075
chr2	68553302	68553341	6583	0.075
chr2	69185942	69185981	6584	0.075
chr2	6921038	6921077	6585	0.075
chr2	70109863	70109902	6586	0.075
chr2	70860479	70860518	6587	0.075
chr2	70917339	70917378	6588	0.075
chr2	7118992	7119031	6589	0.075
chr2	71258165	71258204	6590	0.075
chr2	71586508	71586547	6591	0.075
chr2	71789197	71789236	6592	0.075
chr2	72474114	72474153	6593	0.075
chr2	73584433	73584472	6594	0.075
chr2	74253236	74253275	6595	0.075
chr2	74508511	74508550	6596	0.075
chr2	7514446	7514485	6597	0.075
chr2	76168883	76168922	6598	0.075
chr2	76913493	76913532	6599	0.075
chr2	7987383	7987422	6600	0.075

chr2	80100285	80100324	6601	0.075
chr2	80140154	80140193	6602	0.075
chr2	80669962	80670001	6603	0.075
chr2	8069643	8069682	6604	0.075
chr2	8097056	8097095	6605	0.075
chr2	81328971	81329010	6606	0.075
chr2	84164629	84164668	6607	0.075
chr2	84511101	84511140	6608	0.075
chr2	84996676	84996715	6609	0.075
chr2	85072352	85072391	6610	0.075
chr2	85256747	85256786	6611	0.075
chr2	85562151	85562190	6612	0.075
chr2	85624388	85624427	6613	0.075
chr2	86121064	86121103	6614	0.075
chr2	86609860	86609899	6615	0.075
chr2	8949565	8949604	6616	0.075
chr2	91155239	91155278	6617	0.075
chr2	91237560	91237599	6618	0.075
chr2	9520205	9520244	6619	0.075
chr2	95417929	95417968	6620	0.075
chr2	96797602	96797641	6621	0.075
chr2	97721397	97721436	6622	0.075
chr2	9849158	9849197	6623	0.075
chr2	98892381	98892420	6624	0.075
chr2	213652516	213652554	6625	0.076923077
chr2	136564111	136564159	6626	0.081632653
chr2	200751449	200751493	6627	0.088888889
chr2	227620759	227620799	6628	0.097560976
chr20	44376287	44376399	6629	0.03539823
chr20	39941675	39941779	6630	0.038095238
chr20	60063717	60063791	6631	0.04
chr20	56771549	56771647	6632	0.04040404
chr20	44292078	44292170	6633	0.043010753
chr20	56410080	56410163	6634	0.047619048
chr20	10018070	10018107	6635	0.052631579
chr20	1008506	1008543	6636	0.052631579
chr20	10267408	10267445	6637	0.052631579
chr20	10366854	10366891	6638	0.052631579
chr20	10574970	10575007	6639	0.052631579
chr20	10626875	10626912	6640	0.052631579
chr20	11176038	11176075	6641	0.052631579
chr20	11471953	11471990	6642	0.052631579
chr20	11901186	11901223	6643	0.052631579
chr20	12522975	12523012	6644	0.052631579
chr20	13775715	13775752	6645	0.052631579
chr20	14046856	14046893	6646	0.052631579
chr20	14256844	14256881	6647	0.052631579
chr20	14257451	14257488	6648	0.052631579
chr20	14608266	14608303	6649	0.052631579
chr20	15265149	15265186	6650	0.052631579

chr20	16266637	16266674	6651	0.052631579
chr20	16423048	16423085	6652	0.052631579
chr20	16598434	16598471	6653	0.052631579
chr20	17132876	17132913	6654	0.052631579
chr20	17285051	17285088	6655	0.052631579
chr20	17357186	17357223	6656	0.052631579
chr20	17487779	17487816	6657	0.052631579
chr20	17985609	17985646	6658	0.052631579
chr20	18584019	18584056	6659	0.052631579
chr20	18941738	18941775	6660	0.052631579
chr20	19737527	19737564	6661	0.052631579
chr20	19903608	19903645	6662	0.052631579
chr20	19943361	19943398	6663	0.052631579
chr20	19996650	19996687	6664	0.052631579
chr20	20322673	20322710	6665	0.052631579
chr20	20729686	20729723	6666	0.052631579
chr20	21006959	21006996	6667	0.052631579
chr20	21055476	21055513	6668	0.052631579
chr20	21103271	21103308	6669	0.052631579
chr20	21261286	21261323	6670	0.052631579
chr20	21324937	21324974	6671	0.052631579
chr20	21401608	21401645	6672	0.052631579
chr20	21561250	21561287	6673	0.052631579
chr20	2226596	2226633	6674	0.052631579
chr20	22360649	22360686	6675	0.052631579
chr20	2239491	2239528	6676	0.052631579
chr20	2332292	2332329	6677	0.052631579
chr20	23331657	23331694	6678	0.052631579
chr20	23416851	23416888	6679	0.052631579
chr20	23820605	23820642	6680	0.052631579
chr20	23940292	23940329	6681	0.052631579
chr20	24036895	24036932	6682	0.052631579
chr20	24565841	24565878	6683	0.052631579
chr20	24966594	24966631	6684	0.052631579
chr20	25395773	25395810	6685	0.052631579
chr20	25605046	25605083	6686	0.052631579
chr20	25796146	25796183	6687	0.052631579
chr20	2580759	2580796	6688	0.052631579
chr20	2585054	2585091	6689	0.052631579
chr20	26136868	26136905	6690	0.052631579
chr20	26138021	26138058	6691	0.052631579
chr20	26161599	26161636	6692	0.052631579
chr20	28041504	28041541	6693	0.052631579
chr20	28061774	28061811	6694	0.052631579
chr20	28252420	28252457	6695	0.052631579
chr20	2915429	2915466	6696	0.052631579
chr20	29524552	29524589	6697	0.052631579
chr20	29566046	29566083	6698	0.052631579
chr20	30525267	30525304	6699	0.052631579
chr20	31069822	31069859	6700	0.052631579

chr20	3158250	3158287	6701	0.052631579
chr20	31630218	31630255	6702	0.052631579
chr20	31818460	31818497	6703	0.052631579
chr20	31916359	31916396	6704	0.052631579
chr20	32559558	32559595	6705	0.052631579
chr20	32667440	32667477	6706	0.052631579
chr20	33046937	33046974	6707	0.052631579
chr20	33269483	33269520	6708	0.052631579
chr20	33287277	33287314	6709	0.052631579
chr20	33365602	33365639	6710	0.052631579
chr20	33740340	33740377	6711	0.052631579
chr20	33755039	33755076	6712	0.052631579
chr20	34078825	34078862	6713	0.052631579
chr20	34494008	34494045	6714	0.052631579
chr20	34494259	34494296	6715	0.052631579
chr20	34718192	34718229	6716	0.052631579
chr20	35425303	35425340	6717	0.052631579
chr20	35778737	35778774	6718	0.052631579
chr20	36095887	36095924	6719	0.052631579
chr20	36131375	36131412	6720	0.052631579
chr20	36137593	36137630	6721	0.052631579
chr20	36151772	36151809	6722	0.052631579
chr20	36192102	36192139	6723	0.052631579
chr20	36308301	36308338	6724	0.052631579
chr20	367459	367496	6725	0.052631579
chr20	37191494	37191531	6726	0.052631579
chr20	37376411	37376448	6727	0.052631579
chr20	38048983	38049020	6728	0.052631579
chr20	38937622	38937659	6729	0.052631579
chr20	39230873	39230910	6730	0.052631579
chr20	39456157	39456194	6731	0.052631579
chr20	40292089	40292126	6732	0.052631579
chr20	40547649	40547686	6733	0.052631579
chr20	40612389	40612426	6734	0.052631579
chr20	42071469	42071506	6735	0.052631579
chr20	42241146	42241183	6736	0.052631579
chr20	42492431	42492468	6737	0.052631579
chr20	43878320	43878357	6738	0.052631579
chr20	43890886	43890923	6739	0.052631579
chr20	43945783	43945820	6740	0.052631579
chr20	44426913	44426950	6741	0.052631579
chr20	44877190	44877227	6742	0.052631579
chr20	45881726	45881763	6743	0.052631579
chr20	46861655	46861692	6744	0.052631579
chr20	47021068	47021105	6745	0.052631579
chr20	47100078	47100115	6746	0.052631579
chr20	47549702	47549739	6747	0.052631579
chr20	47932113	47932150	6748	0.052631579
chr20	48104197	48104234	6749	0.052631579
chr20	48123529	48123566	6750	0.052631579

chr20	48241672	48241709	6751	0.052631579
chr20	48613155	48613192	6752	0.052631579
chr20	48625363	48625400	6753	0.052631579
chr20	49104547	49104584	6754	0.052631579
chr20	49157011	49157048	6755	0.052631579
chr20	49157731	49157768	6756	0.052631579
chr20	49466410	49466447	6757	0.052631579
chr20	49520554	49520591	6758	0.052631579
chr20	49671552	49671589	6759	0.052631579
chr20	4971875	4971912	6760	0.052631579
chr20	50578503	50578540	6761	0.052631579
chr20	50654176	50654213	6762	0.052631579
chr20	52597678	52597715	6763	0.052631579
chr20	52640259	52640296	6764	0.052631579
chr20	5265179	5265216	6765	0.052631579
chr20	52737162	52737199	6766	0.052631579
chr20	5280238	5280275	6767	0.052631579
chr20	53474987	53475024	6768	0.052631579
chr20	53943326	53943363	6769	0.052631579
chr20	54013808	54013845	6770	0.052631579
chr20	54039024	54039061	6771	0.052631579
chr20	54044582	54044619	6772	0.052631579
chr20	54104795	54104832	6773	0.052631579
chr20	54374592	54374629	6774	0.052631579
chr20	54378459	54378496	6775	0.052631579
chr20	54525847	54525884	6776	0.052631579
chr20	55385465	55385502	6777	0.052631579
chr20	5574523	5574560	6778	0.052631579
chr20	56226385	56226422	6779	0.052631579
chr20	5624755	5624792	6780	0.052631579
chr20	56469868	56469905	6781	0.052631579
chr20	56866208	56866245	6782	0.052631579
chr20	56884299	56884336	6783	0.052631579
chr20	56899340	56899377	6784	0.052631579
chr20	57064322	57064359	6785	0.052631579
chr20	57317920	57317957	6786	0.052631579
chr20	57443932	57443969	6787	0.052631579
chr20	57923653	57923690	6788	0.052631579
chr20	58034267	58034304	6789	0.052631579
chr20	58258854	58258891	6790	0.052631579
chr20	59199559	59199596	6791	0.052631579
chr20	59850708	59850745	6792	0.052631579
chr20	60142984	60143021	6793	0.052631579
chr20	603178	603215	6794	0.052631579
chr20	60374591	60374628	6795	0.052631579
chr20	60926643	60926680	6796	0.052631579
chr20	61017619	61017656	6797	0.052631579
chr20	61254510	61254547	6798	0.052631579
chr20	61266501	61266538	6799	0.052631579
chr20	61322628	61322665	6800	0.052631579

chr20	61524962	61524999	6801	0.052631579
chr20	62031279	62031316	6802	0.052631579
chr20	62169840	62169877	6803	0.052631579
chr20	62341557	62341594	6804	0.052631579
chr20	6275207	6275244	6805	0.052631579
chr20	6379185	6379222	6806	0.052631579
chr20	7866522	7866559	6807	0.052631579
chr20	8455941	8455978	6808	0.052631579
chr20	906423	906460	6809	0.052631579
chr20	9829006	9829043	6810	0.052631579
chr20	9910994	9911031	6811	0.052631579
chr20	54258148	54258216	6812	0.072463768
chr20	1018218	1018257	6813	0.075
chr20	10228023	10228062	6814	0.075
chr20	10257513	10257552	6815	0.075
chr20	11514602	11514641	6816	0.075
chr20	12654118	12654157	6817	0.075
chr20	13399003	13399042	6818	0.075
chr20	14381696	14381735	6819	0.075
chr20	14592671	14592710	6820	0.075
chr20	15196298	15196337	6821	0.075
chr20	15841357	15841396	6822	0.075
chr20	15904880	15904919	6823	0.075
chr20	17495774	17495813	6824	0.075
chr20	1753484	1753523	6825	0.075
chr20	17571824	17571863	6826	0.075
chr20	17579803	17579842	6827	0.075
chr20	18323100	18323139	6828	0.075
chr20	18537747	18537786	6829	0.075
chr20	19818150	19818189	6830	0.075
chr20	20128434	20128473	6831	0.075
chr20	20392885	20392924	6832	0.075
chr20	21605493	21605532	6833	0.075
chr20	21637893	21637932	6834	0.075
chr20	21812629	21812668	6835	0.075
chr20	22213888	22213927	6836	0.075
chr20	22381067	22381106	6837	0.075
chr20	22605273	22605312	6838	0.075
chr20	22623563	22623602	6839	0.075
chr20	23129769	23129808	6840	0.075
chr20	23632761	23632800	6841	0.075
chr20	2390489	2390528	6842	0.075
chr20	24048849	24048888	6843	0.075
chr20	24408810	24408849	6844	0.075
chr20	24735782	24735821	6845	0.075
chr20	25937441	25937480	6846	0.075
chr20	2767350	2767389	6847	0.075
chr20	2944502	2944541	6848	0.075
chr20	29897004	29897043	6849	0.075
chr20	3093573	3093612	6850	0.075

chr20	31422279	31422318	6851	0.075
chr20	31745368	31745407	6852	0.075
chr20	34611635	34611674	6853	0.075
chr20	34729425	34729464	6854	0.075
chr20	3511682 3511721 6855 0.075			
chr20	36110165	36110204	6856	0.075
chr20	36175303	36175342	6857	0.075
chr20	37260797	37260836	6858	0.075
chr20	37344907	37344946	6859	0.075
chr20	37821801	37821840	6860	0.075
chr20	38267290	38267329	6861	0.075
chr20	38760215	38760254	6862	0.075
chr20	40201241	40201280	6863	0.075
chr20	4103664 4103703 6864 0.075			
chr20	41364759	41364798	6865	0.075
chr20	41557024	41557063	6866	0.075
chr20	42468146	42468185	6867	0.075
chr20	42684083	42684122	6868	0.075
chr20	43365916	43365955	6869	0.075
chr20	43429138	43429177	6870	0.075
chr20	43479174	43479213	6871	0.075
chr20	44238827	44238866	6872	0.075
chr20	44387071	44387110	6873	0.075
chr20	44686202	44686241	6874	0.075
chr20	44788069	44788108	6875	0.075
chr20	44994295	44994334	6876	0.075
chr20	46627814	46627853	6877	0.075
chr20	47063812	47063851	6878	0.075
chr20	47078490	47078529	6879	0.075
chr20	47078570	47078609	6880	0.075
chr20	47292587	47292626	6881	0.075
chr20	49316966	49317005	6882	0.075
chr20	49596498	49596537	6883	0.075
chr20	49724502	49724541	6884	0.075
chr20	50174965	50175004	6885	0.075
chr20	51930332	51930371	6886	0.075
chr20	53625510	53625549	6887	0.075
chr20	54141827	54141866	6888	0.075
chr20	54870413	54870452	6889	0.075
chr20	55560891	55560930	6890	0.075
chr20	55695367	55695406	6891	0.075
chr20	56770966	56771005	6892	0.075
chr20	57032168	57032207	6893	0.075
chr20	57839889	57839928	6894	0.075
chr20	58005320	58005359	6895	0.075
chr20	60502054	60502093	6896	0.075
chr20	60513298	60513337	6897	0.075
chr20	61167082	61167121	6898	0.075
chr20	61351814	61351853	6899	0.075
chr20	61396895	61396934	6900	0.075

chr20	61836394	61836433	6901	0.075
chr20	61846640	61846679	6902	0.075
chr20	62131237	62131276	6903	0.075
chr20	62143405	62143444	6904	0.075
chr20	6565771	6565810	6905	0.075
chr20	6631512	6631551	6906	0.075
chr20	6811491	6811530	6907	0.075
chr20	7196644	7196683	6908	0.075
chr20	8233597	8233636	6909	0.075
chr20	8325369	8325408	6910	0.075
chr20	8664493	8664532	6911	0.075
chr20	9561752	9561791	6912	0.075
chr21	20426745	20426856	6913	0.035714286
chr21	23349678	23349769	6914	0.043478261
chr21	10059255	10059292	6915	0.052631579
chr21	10062466	10062503	6916	0.052631579
chr21	10068039	10068076	6917	0.052631579
chr21	15893953	15893990	6918	0.052631579
chr21	15932305	15932342	6919	0.052631579
chr21	15969323	15969360	6920	0.052631579
chr21	16374586	16374623	6921	0.052631579
chr21	16407139	16407176	6922	0.052631579
chr21	16462946	16462983	6923	0.052631579
chr21	17439440	17439477	6924	0.052631579
chr21	17964336	17964373	6925	0.052631579
chr21	19530327	19530364	6926	0.052631579
chr21	19586782	19586819	6927	0.052631579
chr21	20633789	20633826	6928	0.052631579
chr21	20688664	20688701	6929	0.052631579
chr21	21117223	21117260	6930	0.052631579
chr21	21167421	21167458	6931	0.052631579
chr21	21705420	21705457	6932	0.052631579
chr21	22207992	22208029	6933	0.052631579
chr21	22262445	22262482	6934	0.052631579
chr21	22316003	22316040	6935	0.052631579
chr21	23018539	23018576	6936	0.052631579
chr21	23124853	23124890	6937	0.052631579
chr21	23720082	23720119	6938	0.052631579
chr21	23885813	23885850	6939	0.052631579
chr21	24003649	24003686	6940	0.052631579
chr21	24016684	24016721	6941	0.052631579
chr21	24722503	24722540	6942	0.052631579
chr21	25726940	25726977	6943	0.052631579
chr21	26058477	26058514	6944	0.052631579
chr21	26127365	26127402	6945	0.052631579
chr21	26248834	26248871	6946	0.052631579
chr21	26431432	26431469	6947	0.052631579
chr21	26672026	26672063	6948	0.052631579
chr21	26977625	26977662	6949	0.052631579
chr21	27049120	27049157	6950	0.052631579

chr21	29357594	29357631	6951	0.052631579
chr21	29771385	29771422	6952	0.052631579
chr21	30172424	30172461	6953	0.052631579
chr21	32128327	32128364	6954	0.052631579
chr21	32247993	32248030	6955	0.052631579
chr21	32248546	32248583	6956	0.052631579
chr21	32293407	32293444	6957	0.052631579
chr21	32411545	32411582	6958	0.052631579
chr21	32665523	32665560	6959	0.052631579
chr21	32767527	32767564	6960	0.052631579
chr21	32893322	32893359	6961	0.052631579
chr21	32895913	32895950	6962	0.052631579
chr21	33143827	33143864	6963	0.052631579
chr21	33451809	33451846	6964	0.052631579
chr21	33523922	33523959	6965	0.052631579
chr21	33728780	33728817	6966	0.052631579
chr21	34167405	34167442	6967	0.052631579
chr21	34207485	34207522	6968	0.052631579
chr21	34353865	34353902	6969	0.052631579
chr21	34367903	34367940	6970	0.052631579
chr21	34704011	34704048	6971	0.052631579
chr21	35539398	35539435	6972	0.052631579
chr21	35879470	35879507	6973	0.052631579
chr21	35949087	35949124	6974	0.052631579
chr21	36104246	36104283	6975	0.052631579
chr21	36173271	36173308	6976	0.052631579
chr21	36222122	36222159	6977	0.052631579
chr21	36349503	36349540	6978	0.052631579
chr21	36762263	36762300	6979	0.052631579
chr21	36813305	36813342	6980	0.052631579
chr21	37014228	37014265	6981	0.052631579
chr21	37020334	37020371	6982	0.052631579
chr21	37038903	37038940	6983	0.052631579
chr21	37097591	37097628	6984	0.052631579
chr21	37232575	37232612	6985	0.052631579
chr21	37676001	37676038	6986	0.052631579
chr21	37914239	37914276	6987	0.052631579
chr21	38231577	38231614	6988	0.052631579
chr21	38261994	38262031	6989	0.052631579
chr21	38262710	38262747	6990	0.052631579
chr21	38504915	38504952	6991	0.052631579
chr21	38529725	38529762	6992	0.052631579
chr21	38937064	38937101	6993	0.052631579
chr21	39012571	39012608	6994	0.052631579
chr21	39196233	39196270	6995	0.052631579
chr21	39405817	39405854	6996	0.052631579
chr21	39609113	39609150	6997	0.052631579
chr21	40641463	40641500	6998	0.052631579
chr21	40645057	40645094	6999	0.052631579
chr21	40651200	40651237	7000	0.052631579

chr21	40927937	40927974	7001	0.052631579
chr21	41349879	41349916	7002	0.052631579
chr21	41599118	41599155	7003	0.052631579
chr21	41603535	41603572	7004	0.052631579
chr21	42061902	42061939	7005	0.052631579
chr21	42235575	42235612	7006	0.052631579
chr21	42252396	42252433	7007	0.052631579
chr21	42572374	42572411	7008	0.052631579
chr21	43144398	43144435	7009	0.052631579
chr21	43186283	43186320	7010	0.052631579
chr21	43648286	43648323	7011	0.052631579
chr21	43850285	43850322	7012	0.052631579
chr21	43990418	43990455	7013	0.052631579
chr21	44213499	44213536	7014	0.052631579
chr21	44338259	44338296	7015	0.052631579
chr21	44833464	44833501	7016	0.052631579
chr21	45147795	45147832	7017	0.052631579
chr21	45169107	45169144	7018	0.052631579
chr21	46134187	46134224	7019	0.052631579
chr21	46377065	46377102	7020	0.052631579
chr21	14654979	14655018	7021	0.075
chr21	21173352	21173391	7022	0.075
chr21	23200468	23200507	7023	0.075
chr21	26022143	26022182	7024	0.075
chr21	26302916	26302955	7025	0.075
chr21	28362137	28362176	7026	0.075
chr21	30248083	30248122	7027	0.075
chr21	31511745	31511784	7028	0.075
chr21	32188623	32188662	7029	0.075
chr21	33741801	33741840	7030	0.075
chr21	33837304	33837343	7031	0.075
chr21	33844803	33844842	7032	0.075
chr21	34448554	34448593	7033	0.075
chr21	34637804	34637843	7034	0.075
chr21	34852742	34852781	7035	0.075
chr21	35160273	35160312	7036	0.075
chr21	36448334	36448373	7037	0.075
chr21	36561302	36561341	7038	0.075
chr21	36663977	36664016	7039	0.075
chr21	36738694	36738733	7040	0.075
chr21	38648918	38648957	7041	0.075
chr21	38949753	38949792	7042	0.075
chr21	38964280	38964319	7043	0.075
chr21	39729876	39729915	7044	0.075
chr21	39798384	39798423	7045	0.075
chr21	40223400	40223439	7046	0.075
chr21	40954847	40954886	7047	0.075
chr21	41138055	41138094	7048	0.075
chr21	42188070	42188109	7049	0.075
chr21	42278244	42278283	7050	0.075

chr21	42943678	42943717	7051	0.075
chr21	43149061	43149100	7052	0.075
chr21	43694332	43694371	7053	0.075
chr21	44360451	44360490	7054	0.075
chr21	45759313	45759352	7055	0.075
chr21	46150723	46150762	7056	0.075
chr21	46247763	46247802	7057	0.075
chr21	46639323	46639362	7058	0.075
chr21	46811880	46811919	7059	0.075
chr22	14805522	14805559	7060	0.052631579
chr22	15771548	15771585	7061	0.052631579
chr22	15788365	15788402	7062	0.052631579
chr22	15971973	15972010	7063	0.052631579
chr22	16589507	16589544	7064	0.052631579
chr22	16691262	16691299	7065	0.052631579
chr22	16989371	16989408	7066	0.052631579
chr22	17773350	17773387	7067	0.052631579
chr22	17789775	17789812	7068	0.052631579
chr22	18074615	18074652	7069	0.052631579
chr22	18377686	18377723	7070	0.052631579
chr22	18480735	18480772	7071	0.052631579
chr22	18549520	18549557	7072	0.052631579
chr22	19215122	19215159	7073	0.052631579
chr22	19480818	19480855	7074	0.052631579
chr22	19551593	19551630	7075	0.052631579
chr22	19568260	19568297	7076	0.052631579
chr22	20132771	20132808	7077	0.052631579
chr22	20379070	20379107	7078	0.052631579
chr22	20442550	20442587	7079	0.052631579
chr22	20472579	20472616	7080	0.052631579
chr22	21083192	21083229	7081	0.052631579
chr22	21367554	21367591	7082	0.052631579
chr22	21367871	21367908	7083	0.052631579
chr22	22362553	22362590	7084	0.052631579
chr22	22508548	22508585	7085	0.052631579
chr22	22804774	22804811	7086	0.052631579
chr22	22968801	22968838	7087	0.052631579
chr22	23137410	23137447	7088	0.052631579
chr22	23860490	23860527	7089	0.052631579
chr22	23864971	23865008	7090	0.052631579
chr22	23889972	23890009	7091	0.052631579
chr22	24299913	24299950	7092	0.052631579
chr22	24811577	24811614	7093	0.052631579
chr22	25190426	25190463	7094	0.052631579
chr22	25333930	25333967	7095	0.052631579
chr22	25937197	25937234	7096	0.052631579
chr22	27521414	27521451	7097	0.052631579
chr22	27702898	27702935	7098	0.052631579
chr22	28031929	28031966	7099	0.052631579
chr22	28207045	28207082	7100	0.052631579

chr22	28390947	28390984	7101	0.052631579
chr22	28468452	28468489	7102	0.052631579
chr22	28587561	28587598	7103	0.052631579
chr22	28657350	28657387	7104	0.052631579
chr22	28968254	28968291	7105	0.052631579
chr22	29207231	29207268	7106	0.052631579
chr22	29269760	29269797	7107	0.052631579
chr22	29421041	29421078	7108	0.052631579
chr22	29613793	29613830	7109	0.052631579
chr22	29850874	29850911	7110	0.052631579
chr22	30706826	30706863	7111	0.052631579
chr22	30949791	30949828	7112	0.052631579
chr22	30991974	30992011	7113	0.052631579
chr22	31489082	31489119	7114	0.052631579
chr22	31557710	31557747	7115	0.052631579
chr22	31585208	31585245	7116	0.052631579
chr22	31878109	31878146	7117	0.052631579
chr22	32003054	32003091	7118	0.052631579
chr22	32423830	32423867	7119	0.052631579
chr22	32677852	32677889	7120	0.052631579
chr22	33215596	33215633	7121	0.052631579
chr22	33316882	33316919	7122	0.052631579
chr22	33380922	33380959	7123	0.052631579
chr22	33789823	33789860	7124	0.052631579
chr22	33841026	33841063	7125	0.052631579
chr22	33916161	33916198	7126	0.052631579
chr22	34142312	34142349	7127	0.052631579
chr22	34176507	34176544	7128	0.052631579
chr22	34340166	34340203	7129	0.052631579
chr22	34654370	34654407	7130	0.052631579
chr22	35020217	35020254	7131	0.052631579
chr22	35032404	35032441	7132	0.052631579
chr22	35295936	35295973	7133	0.052631579
chr22	35315467	35315504	7134	0.052631579
chr22	35483649	35483686	7135	0.052631579
chr22	35785337	35785374	7136	0.052631579
chr22	35912485	35912522	7137	0.052631579
chr22	36021023	36021060	7138	0.052631579
chr22	36038574	36038611	7139	0.052631579
chr22	36168993	36169030	7140	0.052631579
chr22	36542861	36542898	7141	0.052631579
chr22	36543690	36543727	7142	0.052631579
chr22	36699712	36699749	7143	0.052631579
chr22	36785294	36785331	7144	0.052631579
chr22	36799120	36799157	7145	0.052631579
chr22	37021472	37021509	7146	0.052631579
chr22	37024746	37024783	7147	0.052631579
chr22	37026770	37026807	7148	0.052631579
chr22	37152796	37152833	7149	0.052631579
chr22	37220607	37220644	7150	0.052631579

chr22	37227312	37227349	7151	0.052631579
chr22	37278599	37278636	7152	0.052631579
chr22	37434794	37434831	7153	0.052631579
chr22	37903983	37904020	7154	0.052631579
chr22	38494243	38494280	7155	0.052631579
chr22	38495095	38495132	7156	0.052631579
chr22	39038868	39038905	7157	0.052631579
chr22	39603407	39603444	7158	0.052631579
chr22	39817761	39817798	7159	0.052631579
chr22	40253924	40253961	7160	0.052631579
chr22	40619675	40619712	7161	0.052631579
chr22	40620388	40620425	7162	0.052631579
chr22	40772265	40772302	7163	0.052631579
chr22	40803801	40803838	7164	0.052631579
chr22	41208566	41208603	7165	0.052631579
chr22	41358603	41358640	7166	0.052631579
chr22	41853454	41853491	7167	0.052631579
chr22	42156067	42156104	7168	0.052631579
chr22	42567062	42567099	7169	0.052631579
chr22	42676458	42676495	7170	0.052631579
chr22	42834350	42834387	7171	0.052631579
chr22	43315461	43315498	7172	0.052631579
chr22	43316192	43316229	7173	0.052631579
chr22	43784896	43784933	7174	0.052631579
chr22	44250661	44250698	7175	0.052631579
chr22	44417787	44417824	7176	0.052631579
chr22	44751730	44751767	7177	0.052631579
chr22	44839657	44839694	7178	0.052631579
chr22	44898139	44898176	7179	0.052631579
chr22	45759842	45759879	7180	0.052631579
chr22	45843834	45843871	7181	0.052631579
chr22	45972555	45972592	7182	0.052631579
chr22	46023882	46023919	7183	0.052631579
chr22	46263696	46263733	7184	0.052631579
chr22	46654304	46654341	7185	0.052631579
chr22	47731887	47731924	7186	0.052631579
chr22	48214143	48214180	7187	0.052631579
chr22	48303795	48303832	7188	0.052631579
chr22	48488975	48489012	7189	0.052631579
chr22	48603212	48603249	7190	0.052631579
chr22	48844183	48844220	7191	0.052631579
chr22	49067832	49067869	7192	0.052631579
chr22	49247610	49247647	7193	0.052631579
chr22	49386124	49386161	7194	0.052631579
chr22	36401617	36401671	7195	0.054545455
chr22	43703806	43703865	7196	0.066666667
chr22	15979221	15979260	7197	0.075
chr22	15981989	15982028	7198	0.075
chr22	15999089	15999128	7199	0.075
chr22	16420167	16420206	7200	0.075

chr22	16657575	16657614	7201	0.075
chr22	18122147	18122186	7202	0.075
chr22	18133612	18133651	7203	0.075
chr22	18189752	18189791	7204	0.075
chr22	18453828	18453867	7205	0.075
chr22	19091199	19091238	7206	0.075
chr22	20419684	20419723	7207	0.075
chr22	21558388	21558427	7208	0.075
chr22	21806202	21806241	7209	0.075
chr22	21864366	21864405	7210	0.075
chr22	21951748	21951787	7211	0.075
chr22	22860373	22860412	7212	0.075
chr22	23266765	23266804	7213	0.075
chr22	24490416	24490455	7214	0.075
chr22	25396108	25396147	7215	0.075
chr22	25469940	25469979	7216	0.075
chr22	26062028	26062067	7217	0.075
chr22	26740231	26740270	7218	0.075
chr22	26971039	26971078	7219	0.075
chr22	27609596	27609635	7220	0.075
chr22	28182093	28182132	7221	0.075
chr22	28362836	28362875	7222	0.075
chr22	30016663	30016702	7223	0.075
chr22	30347023	30347062	7224	0.075
chr22	30352575	30352614	7225	0.075
chr22	30479888	30479927	7226	0.075
chr22	30480901	30480940	7227	0.075
chr22	30668499	30668538	7228	0.075
chr22	31138071	31138110	7229	0.075
chr22	31518693	31518732	7230	0.075
chr22	31625354	31625393	7231	0.075
chr22	31983490	31983529	7232	0.075
chr22	32306713	32306752	7233	0.075
chr22	32577449	32577488	7234	0.075
chr22	33252255	33252294	7235	0.075
chr22	33509349	33509388	7236	0.075
chr22	34736252	34736291	7237	0.075
chr22	35008037	35008076	7238	0.075
chr22	35340416	35340455	7239	0.075
chr22	35363135	35363174	7240	0.075
chr22	35751980	35752019	7241	0.075
chr22	36971974	36972013	7242	0.075
chr22	37209068	37209107	7243	0.075
chr22	38110580	38110619	7244	0.075
chr22	38333465	38333504	7245	0.075
chr22	38438456	38438495	7246	0.075
chr22	38531533	38531572	7247	0.075
chr22	38999501	38999540	7248	0.075
chr22	40031730	40031769	7249	0.075
chr22	40507787	40507826	7250	0.075

chr22	40936368	40936407	7251	0.075
chr22	41345781	41345820	7252	0.075
chr22	42127127	42127166	7253	0.075
chr22	43582887	43582926	7254	0.075
chr22	43638122	43638161	7255	0.075
chr22	43987518	43987557	7256	0.075
chr22	44736883	44736922	7257	0.075
chr22	44869910	44869949	7258	0.075
chr22	44871736	44871775	7259	0.075
chr22	45721072	45721111	7260	0.075
chr22	46637300	46637339	7261	0.075
chr22	46764577	46764616	7262	0.075
chr22	47942300	47942339	7263	0.075
chr22	48282048	48282087	7264	0.075
chr22	48701578	48701617	7265	0.075
chr22	49000402	49000441	7266	0.075
chr22	49190190	49190229	7267	0.075
chr22	49251745	49251784	7268	0.075
chr22	49499874	49499913	7269	0.075
chr3	192479402	192479514	7270	0.03539823
chr3	116185226	116185330	7271	0.038095238
chr3	10388645	10388739	7272	0.042105263
chr3	171193113	171193204	7273	0.043478261
chr3	59341412	59341500	7274	0.04494382
chr3	113569479	113569563	7275	0.047058824
chr3	163618	163657	7276	0.05
chr3	106959750	106959827	7277	0.051282051
chr3	87390579	87390656	7278	0.051282051
chr3	100739118	100739155	7279	0.052631579
chr3	100875966	100876003	7280	0.052631579
chr3	100894158	100894195	7281	0.052631579
chr3	101035964	101036001	7282	0.052631579
chr3	101206571	101206608	7283	0.052631579
chr3	101225424	101225461	7284	0.052631579
chr3	101273409	101273446	7285	0.052631579
chr3	101326691	101326728	7286	0.052631579
chr3	101504852	101504889	7287	0.052631579
chr3	10158506	10158543	7288	0.052631579
chr3	101843441	101843478	7289	0.052631579
chr3	102005593	102005630	7290	0.052631579
chr3	102571060	102571097	7291	0.052631579
chr3	102870922	102870959	7292	0.052631579
chr3	103149956	103149993	7293	0.052631579
chr3	10332014	10332051	7294	0.052631579
chr3	103427749	103427786	7295	0.052631579
chr3	103706610	103706647	7296	0.052631579
chr3	103811615	103811652	7297	0.052631579
chr3	10418993	10419030	7298	0.052631579
chr3	104678371	104678408	7299	0.052631579
chr3	104933908	104933945	7300	0.052631579

chr3	105770439	105770476	7301	0.052631579
chr3	106164465	106164502	7302	0.052631579
chr3	106165320	106165357	7303	0.052631579
chr3	106893033	106893070	7304	0.052631579
chr3	107034255	107034292	7305	0.052631579
chr3	107135853	107135890	7306	0.052631579
chr3	107222737	107222774	7307	0.052631579
chr3	107222993	107223030	7308	0.052631579
chr3	107905577	107905614	7309	0.052631579
chr3	107960824	107960861	7310	0.052631579
chr3	10800357	10800394	7311	0.052631579
chr3	108029608	108029645	7312	0.052631579
chr3	108175513	108175550	7313	0.052631579
chr3	109467345	109467382	7314	0.052631579
chr3	109728875	109728912	7315	0.052631579
chr3	110102116	110102153	7316	0.052631579
chr3	110122020	110122057	7317	0.052631579
chr3	111547660	111547697	7318	0.052631579
chr3	111918010	111918047	7319	0.052631579
chr3	112101639	112101676	7320	0.052631579
chr3	112515754	112515791	7321	0.052631579
chr3	113000323	113000360	7322	0.052631579
chr3	113086630	113086667	7323	0.052631579
chr3	113807186	113807223	7324	0.052631579
chr3	113898430	113898467	7325	0.052631579
chr3	113920232	113920269	7326	0.052631579
chr3	114966720	114966757	7327	0.052631579
chr3	115155462	115155499	7328	0.052631579
chr3	115553370	115553407	7329	0.052631579
chr3	115576924	115576961	7330	0.052631579
chr3	11607193	11607230	7331	0.052631579
chr3	116550200	116550237	7332	0.052631579
chr3	116822585	116822622	7333	0.052631579
chr3	117105327	117105364	7334	0.052631579
chr3	117269790	117269827	7335	0.052631579
chr3	117327234	117327271	7336	0.052631579
chr3	117498738	117498775	7337	0.052631579
chr3	117625017	117625054	7338	0.052631579
chr3	117975000	117975037	7339	0.052631579
chr3	118115090	118115127	7340	0.052631579
chr3	118460196	118460233	7341	0.052631579
chr3	118488195	118488232	7342	0.052631579
chr3	118929561	118929598	7343	0.052631579
chr3	119203291	119203328	7344	0.052631579
chr3	119836021	119836058	7345	0.052631579
chr3	12057599	12057636	7346	0.052631579
chr3	121038149	121038186	7347	0.052631579
chr3	121601462	121601499	7348	0.052631579
chr3	121641015	121641052	7349	0.052631579
chr3	12167683	12167720	7350	0.052631579

chr3	121854512	121854549	7351	0.052631579
chr3	12262026	12262063	7352	0.052631579
chr3	12295160	12295197	7353	0.052631579
chr3	12314454	12314491	7354	0.052631579
chr3	123207525	123207562	7355	0.052631579
chr3	123466407	123466444	7356	0.052631579
chr3	124080807	124080844	7357	0.052631579
chr3	124568581	124568618	7358	0.052631579
chr3	12506288	12506325	7359	0.052631579
chr3	12540338	12540375	7360	0.052631579
chr3	125618399	125618436	7361	0.052631579
chr3	126191208	126191245	7362	0.052631579
chr3	126529717	126529754	7363	0.052631579
chr3	126530200	126530237	7364	0.052631579
chr3	126757724	126757761	7365	0.052631579
chr3	127017754	127017791	7366	0.052631579
chr3	128151794	128151831	7367	0.052631579
chr3	128229490	128229527	7368	0.052631579
chr3	12831649	12831686	7369	0.052631579
chr3	128418170	128418207	7370	0.052631579
chr3	128670043	128670080	7371	0.052631579
chr3	128790952	128790989	7372	0.052631579
chr3	128800971	128801008	7373	0.052631579
chr3	128879822	128879859	7374	0.052631579
chr3	129268593	129268630	7375	0.052631579
chr3	129272526	129272563	7376	0.052631579
chr3	129272620	129272657	7377	0.052631579
chr3	129325202	129325239	7378	0.052631579
chr3	130142874	130142911	7379	0.052631579
chr3	130312522	130312559	7380	0.052631579
chr3	130329505	130329542	7381	0.052631579
chr3	130924533	130924570	7382	0.052631579
chr3	131163662	131163699	7383	0.052631579
chr3	131501304	131501341	7384	0.052631579
chr3	132803504	132803541	7385	0.052631579
chr3	133589297	133589334	7386	0.052631579
chr3	133606525	133606562	7387	0.052631579
chr3	133613380	133613417	7388	0.052631579
chr3	134075250	134075287	7389	0.052631579
chr3	134097146	134097183	7390	0.052631579
chr3	134131457	134131494	7391	0.052631579
chr3	134450253	134450290	7392	0.052631579
chr3	134519529	134519566	7393	0.052631579
chr3	134563577	134563614	7394	0.052631579
chr3	134564334	134564371	7395	0.052631579
chr3	13477656	13477693	7396	0.052631579
chr3	135238210	135238247	7397	0.052631579
chr3	135240751	135240788	7398	0.052631579
chr3	135572829	135572866	7399	0.052631579
chr3	135579542	135579579	7400	0.052631579

chr3	135879382	135879419	7401	0.052631579
chr3	13605660	13605697	7402	0.052631579
chr3	136670832	136670869	7403	0.052631579
chr3	136699728	136699765	7404	0.052631579
chr3	136786164	136786201	7405	0.052631579
chr3	136819045	136819082	7406	0.052631579
chr3	137168095	137168132	7407	0.052631579
chr3	137762932	137762969	7408	0.052631579
chr3	138242919	138242956	7409	0.052631579
chr3	13875374	13875411	7410	0.052631579
chr3	138844001	138844038	7411	0.052631579
chr3	13889062	13889099	7412	0.052631579
chr3	138975767	138975804	7413	0.052631579
chr3	141215587	141215624	7414	0.052631579
chr3	141254476	141254513	7415	0.052631579
chr3	141662611	141662648	7416	0.052631579
chr3	141918363	141918400	7417	0.052631579
chr3	142276074	142276111	7418	0.052631579
chr3	142678988	142679025	7419	0.052631579
chr3	142771077	142771114	7420	0.052631579
chr3	143581628	143581665	7421	0.052631579
chr3	143671471	143671508	7422	0.052631579
chr3	144026668	144026705	7423	0.052631579
chr3	144143043	144143080	7424	0.052631579
chr3	144143943	144143980	7425	0.052631579
chr3	144473672	144473709	7426	0.052631579
chr3	144481863	144481900	7427	0.052631579
chr3	144517444	144517481	7428	0.052631579
chr3	144589069	144589106	7429	0.052631579
chr3	144912280	144912317	7430	0.052631579
chr3	144944598	144944635	7431	0.052631579
chr3	144981649	144981686	7432	0.052631579
chr3	145027853	145027890	7433	0.052631579
chr3	145038891	145038928	7434	0.052631579
chr3	145811079	145811116	7435	0.052631579
chr3	147271160	147271197	7436	0.052631579
chr3	147967756	147967793	7437	0.052631579
chr3	148298658	148298695	7438	0.052631579
chr3	148381555	148381592	7439	0.052631579
chr3	149075236	149075273	7440	0.052631579
chr3	149075932	149075969	7441	0.052631579
chr3	149121000	149121037	7442	0.052631579
chr3	149539978	149540015	7443	0.052631579
chr3	149603177	149603214	7444	0.052631579
chr3	149603961	149603998	7445	0.052631579
chr3	149715998	149716035	7446	0.052631579
chr3	150577309	150577346	7447	0.052631579
chr3	151576567	151576604	7448	0.052631579
chr3	151737535	151737572	7449	0.052631579
chr3	152224385	152224422	7450	0.052631579

chr3	152467177	152467214	7451	0.052631579
chr3	152479712	152479749	7452	0.052631579
chr3	152591274	152591311	7453	0.052631579
chr3	152679553	152679590	7454	0.052631579
chr3	152696076	152696113	7455	0.052631579
chr3	152699334	152699371	7456	0.052631579
chr3	153499622	153499659	7457	0.052631579
chr3	153662923	153662960	7458	0.052631579
chr3	153727623	153727660	7459	0.052631579
chr3	154772290	154772327	7460	0.052631579
chr3	154987149	154987186	7461	0.052631579
chr3	155799933	155799970	7462	0.052631579
chr3	155847363	155847400	7463	0.052631579
chr3	155922520	155922557	7464	0.052631579
chr3	156794542	156794579	7465	0.052631579
chr3	156888424	156888461	7466	0.052631579
chr3	157053781	157053818	7467	0.052631579
chr3	157518019	157518056	7468	0.052631579
chr3	15753625	15753662	7469	0.052631579
chr3	157585014	157585051	7470	0.052631579
chr3	157679827	157679864	7471	0.052631579
chr3	157982145	157982182	7472	0.052631579
chr3	158012499	158012536	7473	0.052631579
chr3	158017966	158018003	7474	0.052631579
chr3	158348789	158348826	7475	0.052631579
chr3	158538760	158538797	7476	0.052631579
chr3	159508890	159508927	7477	0.052631579
chr3	159867181	159867218	7478	0.052631579
chr3	160061917	160061954	7479	0.052631579
chr3	160547754	160547791	7480	0.052631579
chr3	160587369	160587406	7481	0.052631579
chr3	161313487	161313524	7482	0.052631579
chr3	161543245	161543282	7483	0.052631579
chr3	162127578	162127615	7484	0.052631579
chr3	162191774	162191811	7485	0.052631579
chr3	16254872	16254909	7486	0.052631579
chr3	163172927	163172964	7487	0.052631579
chr3	163340836	163340873	7488	0.052631579
chr3	16394322	16394359	7489	0.052631579
chr3	16400151	16400188	7490	0.052631579
chr3	165565231	165565268	7491	0.052631579
chr3	165932868	165932905	7492	0.052631579
chr3	166041373	166041410	7493	0.052631579
chr3	166584554	166584591	7494	0.052631579
chr3	167064116	167064153	7495	0.052631579
chr3	168012293	168012330	7496	0.052631579
chr3	169416538	169416575	7497	0.052631579
chr3	169417291	169417328	7498	0.052631579
chr3	169421025	169421062	7499	0.052631579
chr3	169906589	169906626	7500	0.052631579

chr3	169916926	169916963	7501	0.052631579
chr3	170074080	170074117	7502	0.052631579
chr3	170347029	170347066	7503	0.052631579
chr3	170347770	170347807	7504	0.052631579
chr3	170565247	170565284	7505	0.052631579
chr3	170695053	170695090	7506	0.052631579
chr3	171321139	171321176	7507	0.052631579
chr3	171794540	171794577	7508	0.052631579
chr3	171893390	171893427	7509	0.052631579
chr3	172307137	172307174	7510	0.052631579
chr3	172347738	172347775	7511	0.052631579
chr3	172592607	172592644	7512	0.052631579
chr3	17291590	17291627	7513	0.052631579
chr3	17292385	17292422	7514	0.052631579
chr3	173084606	173084643	7515	0.052631579
chr3	173155487	173155524	7516	0.052631579
chr3	17328529	17328566	7517	0.052631579
chr3	17329262	17329299	7518	0.052631579
chr3	173302915	173302952	7519	0.052631579
chr3	173303338	173303375	7520	0.052631579
chr3	173601961	173601998	7521	0.052631579
chr3	174206545	174206582	7522	0.052631579
chr3	174564822	174564859	7523	0.052631579
chr3	174565597	174565634	7524	0.052631579
chr3	174635527	174635564	7525	0.052631579
chr3	174785118	174785155	7526	0.052631579
chr3	174807051	174807088	7527	0.052631579
chr3	175098403	175098440	7528	0.052631579
chr3	175498740	175498777	7529	0.052631579
chr3	175785501	175785538	7530	0.052631579
chr3	17588919	17588956	7531	0.052631579
chr3	175973790	175973827	7532	0.052631579
chr3	176072010	176072047	7533	0.052631579
chr3	176553298	176553335	7534	0.052631579
chr3	176604013	176604050	7535	0.052631579
chr3	177491039	177491076	7536	0.052631579
chr3	177799288	177799325	7537	0.052631579
chr3	177981480	177981517	7538	0.052631579
chr3	17820668	17820705	7539	0.052631579
chr3	178796979	178797016	7540	0.052631579
chr3	179083159	179083196	7541	0.052631579
chr3	180430771	180430808	7542	0.052631579
chr3	180727484	180727521	7543	0.052631579
chr3	180788814	180788851	7544	0.052631579
chr3	180961154	180961191	7545	0.052631579
chr3	181267154	181267191	7546	0.052631579
chr3	18130060	18130097	7547	0.052631579
chr3	181488050	181488087	7548	0.052631579
chr3	181945069	181945106	7549	0.052631579
chr3	182114953	182114990	7550	0.052631579

chr3	182256952	182256989	7551	0.052631579
chr3	182810851	182810888	7552	0.052631579
chr3	182912939	182912976	7553	0.052631579
chr3	184029771	184029808	7554	0.052631579
chr3	184092648	184092685	7555	0.052631579
chr3	18413673	18413710	7556	0.052631579
chr3	184218557	184218594	7557	0.052631579
chr3	18424973	18425010	7558	0.052631579
chr3	18511445	18511482	7559	0.052631579
chr3	185152225	185152262	7560	0.052631579
chr3	185460364	185460401	7561	0.052631579
chr3	185515926	185515963	7562	0.052631579
chr3	185790928	185790965	7563	0.052631579
chr3	18582494	18582531	7564	0.052631579
chr3	18668742	18668779	7565	0.052631579
chr3	186774433	186774470	7566	0.052631579
chr3	186852414	186852451	7567	0.052631579
chr3	186868008	186868045	7568	0.052631579
chr3	186870372	186870409	7569	0.052631579
chr3	187257684	187257721	7570	0.052631579
chr3	187268998	187269035	7571	0.052631579
chr3	187432072	187432109	7572	0.052631579
chr3	187989849	187989886	7573	0.052631579
chr3	18801201	18801238	7574	0.052631579
chr3	188192304	188192341	7575	0.052631579
chr3	188421527	188421564	7576	0.052631579
chr3	18931184	18931221	7577	0.052631579
chr3	189848911	189848948	7578	0.052631579
chr3	190151991	190152028	7579	0.052631579
chr3	190161341	190161378	7580	0.052631579
chr3	190984760	190984797	7581	0.052631579
chr3	191062962	191062999	7582	0.052631579
chr3	191127319	191127356	7583	0.052631579
chr3	191207433	191207470	7584	0.052631579
chr3	191236226	191236263	7585	0.052631579
chr3	191578065	191578102	7586	0.052631579
chr3	191581503	191581540	7587	0.052631579
chr3	191874319	191874356	7588	0.052631579
chr3	19243435	19243472	7589	0.052631579
chr3	192447894	192447931	7590	0.052631579
chr3	193196502	193196539	7591	0.052631579
chr3	193222826	193222863	7592	0.052631579
chr3	193511541	193511578	7593	0.052631579
chr3	193608646	193608683	7594	0.052631579
chr3	193614937	193614974	7595	0.052631579
chr3	193702964	193703001	7596	0.052631579
chr3	193703348	193703385	7597	0.052631579
chr3	194670290	194670327	7598	0.052631579
chr3	195081854	195081891	7599	0.052631579
chr3	195213552	195213589	7600	0.052631579

chr3	195342521	195342558	7601	0.052631579
chr3	195343274	195343311	7602	0.052631579
chr3	19539606	19539643	7603	0.052631579
chr3	19540313	19540350	7604	0.052631579
chr3	195425315	195425352	7605	0.052631579
chr3	195567755	195567792	7606	0.052631579
chr3	195919199	195919236	7607	0.052631579
chr3	195933374	195933411	7608	0.052631579
chr3	196210960	196210997	7609	0.052631579
chr3	196718659	196718696	7610	0.052631579
chr3	197170312	197170349	7611	0.052631579
chr3	197174629	197174666	7612	0.052631579
chr3	19773860	19773897	7613	0.052631579
chr3	197883168	197883205	7614	0.052631579
chr3	198314895	198314932	7615	0.052631579
chr3	198948523	198948560	7616	0.052631579
chr3	199165367	199165404	7617	0.052631579
chr3	20109987	20110024	7618	0.052631579
chr3	2042010	2042047	7619	0.052631579
chr3	2042095	2042132	7620	0.052631579
chr3	20886291	20886328	7621	0.052631579
chr3	21241553	21241590	7622	0.052631579
chr3	21332350	21332387	7623	0.052631579
chr3	21333123	21333160	7624	0.052631579
chr3	21427794	21427831	7625	0.052631579
chr3	2167170	2167207	7626	0.052631579
chr3	22311691	22311728	7627	0.052631579
chr3	22506555	22506592	7628	0.052631579
chr3	23143031	23143068	7629	0.052631579
chr3	23943964	23944001	7630	0.052631579
chr3	23970221	23970258	7631	0.052631579
chr3	23970411	23970448	7632	0.052631579
chr3	24059095	24059132	7633	0.052631579
chr3	24177126	24177163	7634	0.052631579
chr3	24286657	24286694	7635	0.052631579
chr3	24370003	24370040	7636	0.052631579
chr3	24523989	24524026	7637	0.052631579
chr3	25116075	25116112	7638	0.052631579
chr3	26106294	26106331	7639	0.052631579
chr3	26399529	26399566	7640	0.052631579
chr3	26447982	26448019	7641	0.052631579
chr3	27308070	27308107	7642	0.052631579
chr3	27420255	27420292	7643	0.052631579
chr3	27734185	27734222	7644	0.052631579
chr3	27814880	27814917	7645	0.052631579
chr3	2855398	2855435	7646	0.052631579
chr3	28757656	28757693	7647	0.052631579
chr3	2904575	2904612	7648	0.052631579
chr3	29131240	29131277	7649	0.052631579
chr3	29214099	29214136	7650	0.052631579

chr3	29221347	29221384	7651	0.052631579
chr3	29363305	29363342	7652	0.052631579
chr3	30272710	30272747	7653	0.052631579
chr3	3065273	3065310	7654	0.052631579
chr3	3086377	3086414	7655	0.052631579
chr3	30975827	30975864	7656	0.052631579
chr3	30976549	30976586	7657	0.052631579
chr3	31842337	31842374	7658	0.052631579
chr3	32122573	32122610	7659	0.052631579
chr3	32123347	32123384	7660	0.052631579
chr3	32884541	32884578	7661	0.052631579
chr3	33097636	33097673	7662	0.052631579
chr3	34086536	34086573	7663	0.052631579
chr3	34160052	34160089	7664	0.052631579
chr3	34177019	34177056	7665	0.052631579
chr3	34232733	34232770	7666	0.052631579
chr3	34260748	34260785	7667	0.052631579
chr3	34845633	34845670	7668	0.052631579
chr3	34846802	34846839	7669	0.052631579
chr3	35572668	35572705	7670	0.052631579
chr3	35755853	35755890	7671	0.052631579
chr3	35849543	35849580	7672	0.052631579
chr3	36322030	36322067	7673	0.052631579
chr3	36894916	36894953	7674	0.052631579
chr3	37153445	37153482	7675	0.052631579
chr3	37509201	37509238	7676	0.052631579
chr3	37605372	37605409	7677	0.052631579
chr3	37703679	37703716	7678	0.052631579
chr3	37795420	37795457	7679	0.052631579
chr3	37910088	37910125	7680	0.052631579
chr3	38027900	38027937	7681	0.052631579
chr3	38378699	38378736	7682	0.052631579
chr3	38592173	38592210	7683	0.052631579
chr3	38722312	38722349	7684	0.052631579
chr3	38725430	38725467	7685	0.052631579
chr3	38882024	38882061	7686	0.052631579
chr3	39014652	39014689	7687	0.052631579
chr3	39036303	39036340	7688	0.052631579
chr3	39424961	39424998	7689	0.052631579
chr3	40099170	40099207	7690	0.052631579
chr3	40111794	40111831	7691	0.052631579
chr3	40222629	40222666	7692	0.052631579
chr3	40269796	40269833	7693	0.052631579
chr3	40294066	40294103	7694	0.052631579
chr3	41263866	41263903	7695	0.052631579
chr3	41544470	41544507	7696	0.052631579
chr3	42068588	42068625	7697	0.052631579
chr3	42069383	42069420	7698	0.052631579
chr3	42169250	42169287	7699	0.052631579
chr3	42352906	42352943	7700	0.052631579

chr3	42358351	42358388	7701	0.052631579
chr3	42505429	42505466	7702	0.052631579
chr3	42506216	42506253	7703	0.052631579
chr3	42560504	42560541	7704	0.052631579
chr3	42638001	42638038	7705	0.052631579
chr3	43254279	43254316	7706	0.052631579
chr3	4329670	4329707	7707	0.052631579
chr3	43338703	43338740	7708	0.052631579
chr3	43565346	43565383	7709	0.052631579
chr3	43859954	43859991	7710	0.052631579
chr3	44013765	44013802	7711	0.052631579
chr3	44014634	44014671	7712	0.052631579
chr3	44573885	44573922	7713	0.052631579
chr3	44574466	44574503	7714	0.052631579
chr3	44607233	44607270	7715	0.052631579
chr3	44900899	44900936	7716	0.052631579
chr3	44901733	44901770	7717	0.052631579
chr3	45241803	45241840	7718	0.052631579
chr3	45481913	45481950	7719	0.052631579
chr3	4564508	4564545	7720	0.052631579
chr3	45869791	45869828	7721	0.052631579
chr3	45939022	45939059	7722	0.052631579
chr3	45939773	45939810	7723	0.052631579
chr3	46283115	46283152	7724	0.052631579
chr3	4640471	4640508	7725	0.052631579
chr3	46726006	46726043	7726	0.052631579
chr3	47429207	47429244	7727	0.052631579
chr3	47867548	47867585	7728	0.052631579
chr3	47907201	47907238	7729	0.052631579
chr3	48010695	48010732	7730	0.052631579
chr3	4834763	4834800	7731	0.052631579
chr3	48439343	48439380	7732	0.052631579
chr3	48536167	48536204	7733	0.052631579
chr3	48602906	48602943	7734	0.052631579
chr3	48656557	48656594	7735	0.052631579
chr3	48657218	48657255	7736	0.052631579
chr3	49039250	49039287	7737	0.052631579
chr3	49279955	49279992	7738	0.052631579
chr3	49545252	49545289	7739	0.052631579
chr3	49922599	49922636	7740	0.052631579
chr3	50081484	50081521	7741	0.052631579
chr3	50126493	50126530	7742	0.052631579
chr3	50164572	50164609	7743	0.052631579
chr3	50377875	50377912	7744	0.052631579
chr3	50454120	50454157	7745	0.052631579
chr3	51272579	51272616	7746	0.052631579
chr3	51387091	51387128	7747	0.052631579
chr3	51405007	51405044	7748	0.052631579
chr3	51788464	51788501	7749	0.052631579
chr3	51951307	51951344	7750	0.052631579

chr3	51972009	51972046	7751	0.052631579
chr3	51980665	51980702	7752	0.052631579
chr3	52395935	52395972	7753	0.052631579
chr3	52396521	52396558	7754	0.052631579
chr3	52530707	52530744	7755	0.052631579
chr3	52702786	52702823	7756	0.052631579
chr3	53067656	53067693	7757	0.052631579
chr3	53169276	53169313	7758	0.052631579
chr3	53562649	53562686	7759	0.052631579
chr3	53563305	53563342	7760	0.052631579
chr3	5385011	5385048	7761	0.052631579
chr3	54058554	54058591	7762	0.052631579
chr3	54059356	54059393	7763	0.052631579
chr3	54177473	54177510	7764	0.052631579
chr3	54375084	54375121	7765	0.052631579
chr3	54501900	54501937	7766	0.052631579
chr3	54622430	54622467	7767	0.052631579
chr3	54973425	54973462	7768	0.052631579
chr3	54981362	54981399	7769	0.052631579
chr3	55136543	55136580	7770	0.052631579
chr3	55500783	55500820	7771	0.052631579
chr3	55524950	55524987	7772	0.052631579
chr3	56367107	56367144	7773	0.052631579
chr3	56427954	56427991	7774	0.052631579
chr3	56633282	56633319	7775	0.052631579
chr3	57523690	57523727	7776	0.052631579
chr3	57524267	57524304	7777	0.052631579
chr3	57534048	57534085	7778	0.052631579
chr3	57871368	57871405	7779	0.052631579
chr3	5836700	5836737	7780	0.052631579
chr3	58703210	58703247	7781	0.052631579
chr3	58705885	58705922	7782	0.052631579
chr3	59379644	59379681	7783	0.052631579
chr3	59387622	59387659	7784	0.052631579
chr3	59715613	59715650	7785	0.052631579
chr3	59778359	59778396	7786	0.052631579
chr3	60384753	60384790	7787	0.052631579
chr3	60414759	60414796	7788	0.052631579
chr3	60487229	60487266	7789	0.052631579
chr3	60488021	60488058	7790	0.052631579
chr3	608210	608247	7791	0.052631579
chr3	61115263	61115300	7792	0.052631579
chr3	62156133	62156170	7793	0.052631579
chr3	62234283	62234320	7794	0.052631579
chr3	62697091	62697128	7795	0.052631579
chr3	62732540	62732577	7796	0.052631579
chr3	63350679	63350716	7797	0.052631579
chr3	63780239	63780276	7798	0.052631579
chr3	63780578	63780615	7799	0.052631579
chr3	63836638	63836675	7800	0.052631579

chr3	63956638	63956675	7801	0.052631579
chr3	63959336	63959373	7802	0.052631579
chr3	63979542	63979579	7803	0.052631579
chr3	65000420	65000457	7804	0.052631579
chr3	65074887	65074924	7805	0.052631579
chr3	65338514	65338551	7806	0.052631579
chr3	66490539	66490576	7807	0.052631579
chr3	66572731	66572768	7808	0.052631579
chr3	66599863	66599900	7809	0.052631579
chr3	66689157	66689194	7810	0.052631579
chr3	66726275	66726312	7811	0.052631579
chr3	66735554	66735591	7812	0.052631579
chr3	66850906	66850943	7813	0.052631579
chr3	67844952	67844989	7814	0.052631579
chr3	68179089	68179126	7815	0.052631579
chr3	68296139	68296176	7816	0.052631579
chr3	68971457	68971494	7817	0.052631579
chr3	69087988	69088025	7818	0.052631579
chr3	69312955	69312992	7819	0.052631579
chr3	69329646	69329683	7820	0.052631579
chr3	69689486	69689523	7821	0.052631579
chr3	70162089	70162126	7822	0.052631579
chr3	70162210	70162247	7823	0.052631579
chr3	70266259	70266296	7824	0.052631579
chr3	70541260	70541297	7825	0.052631579
chr3	7055147 7055184	7826	0.052631579	
chr3	7079307 7079344	7827	0.052631579	
chr3	70828091	70828128	7828	0.052631579
chr3	70964865	70964902	7829	0.052631579
chr3	71103990	71104027	7830	0.052631579
chr3	71393359	71393396	7831	0.052631579
chr3	71394166	71394203	7832	0.052631579
chr3	71493923	71493960	7833	0.052631579
chr3	71561538	71561575	7834	0.052631579
chr3	72294194	72294231	7835	0.052631579
chr3	72496613	72496650	7836	0.052631579
chr3	73179350	73179387	7837	0.052631579
chr3	73517541	73517578	7838	0.052631579
chr3	73676710	73676747	7839	0.052631579
chr3	73983296	73983333	7840	0.052631579
chr3	73998140	73998177	7841	0.052631579
chr3	74602454	74602491	7842	0.052631579
chr3	74659753	74659790	7843	0.052631579
chr3	75100813	75100850	7844	0.052631579
chr3	75314029	75314066	7845	0.052631579
chr3	75314942	75314979	7846	0.052631579
chr3	75568505	75568542	7847	0.052631579
chr3	75774012	75774049	7848	0.052631579
chr3	76299289	76299326	7849	0.052631579
chr3	76299568	76299605	7850	0.052631579

chr3	76437608	76437645	7851	0.052631579
chr3	76777373	76777410	7852	0.052631579
chr3	76886316	76886353	7853	0.052631579
chr3	77217146	77217183	7854	0.052631579
chr3	77472561	77472598	7855	0.052631579
chr3	7757054	7757091	7856	0.052631579
chr3	77625184	77625221	7857	0.052631579
chr3	78170356	78170393	7858	0.052631579
chr3	78608425	78608462	7859	0.052631579
chr3	78783634	78783671	7860	0.052631579
chr3	78930870	78930907	7861	0.052631579
chr3	78977670	78977707	7862	0.052631579
chr3	79148112	79148149	7863	0.052631579
chr3	79215342	79215379	7864	0.052631579
chr3	79706256	79706293	7865	0.052631579
chr3	80187765	80187802	7866	0.052631579
chr3	80230267	80230304	7867	0.052631579
chr3	80650846	80650883	7868	0.052631579
chr3	81013852	81013889	7869	0.052631579
chr3	81233718	81233755	7870	0.052631579
chr3	81304497	81304534	7871	0.052631579
chr3	8137910	8137947	7872	0.052631579
chr3	82769226	82769263	7873	0.052631579
chr3	8289584	8289621	7874	0.052631579
chr3	82934196	82934233	7875	0.052631579
chr3	83444133	83444170	7876	0.052631579
chr3	83619257	83619294	7877	0.052631579
chr3	83849301	83849338	7878	0.052631579
chr3	83898569	83898606	7879	0.052631579
chr3	83915692	83915729	7880	0.052631579
chr3	83940086	83940123	7881	0.052631579
chr3	8502521	8502558	7882	0.052631579
chr3	85645661	85645698	7883	0.052631579
chr3	85712535	85712572	7884	0.052631579
chr3	8587219	8587256	7885	0.052631579
chr3	87341377	87341414	7886	0.052631579
chr3	87917352	87917389	7887	0.052631579
chr3	88164063	88164100	7888	0.052631579
chr3	88263374	88263411	7889	0.052631579
chr3	88263459	88263496	7890	0.052631579
chr3	88420565	88420602	7891	0.052631579
chr3	89245718	89245755	7892	0.052631579
chr3	9128798	9128835	7893	0.052631579
chr3	9171050	9171087	7894	0.052631579
chr3	9387747	9387784	7895	0.052631579
chr3	95819910	95819947	7896	0.052631579
chr3	97131994	97132031	7897	0.052631579
chr3	98550785	98550822	7898	0.052631579
chr3	99052899	99052936	7899	0.052631579
chr3	99178049	99178086	7900	0.052631579

chr3	99254751	99254788	7901	0.052631579
chr3	9927779	9927816	7902	0.052631579
chr3	9950879	9950916	7903	0.052631579
chr3	99800235	99800272	7904	0.052631579
chr3	46237788	46237861	7905	0.054054054
chr3	172317251	172317322	7906	0.055555556
chr3	107356209	107356274	7907	0.060606061
chr3	112949854	112949916	7908	0.063492063
chr3	101156912	101156951	7909	0.075
chr3	102022214	102022253	7910	0.075
chr3	102051605	102051644	7911	0.075
chr3	102535336	102535375	7912	0.075
chr3	10405033	10405072	7913	0.075
chr3	10436614	10436653	7914	0.075
chr3	10590475	10590514	7915	0.075
chr3	106778337	106778376	7916	0.075
chr3	10742230	10742269	7917	0.075
chr3	109029694	109029733	7918	0.075
chr3	110258482	110258521	7919	0.075
chr3	11181592	11181631	7920	0.075
chr3	112792499	112792538	7921	0.075
chr3	113844830	113844869	7922	0.075
chr3	114325444	114325483	7923	0.075
chr3	114506608	114506647	7924	0.075
chr3	115045896	115045935	7925	0.075
chr3	115347317	115347356	7926	0.075
chr3	115602491	115602530	7927	0.075
chr3	115817476	115817515	7928	0.075
chr3	116365646	116365685	7929	0.075
chr3	116773260	116773299	7930	0.075
chr3	116964965	116965004	7931	0.075
chr3	117897867	117897906	7932	0.075
chr3	118467186	118467225	7933	0.075
chr3	120428336	120428375	7934	0.075
chr3	120639391	120639430	7935	0.075
chr3	120653749	120653788	7936	0.075
chr3	120671086	120671125	7937	0.075
chr3	121378682	121378721	7938	0.075
chr3	123487500	123487539	7939	0.075
chr3	12367150	12367189	7940	0.075
chr3	123753199	123753238	7941	0.075
chr3	124111773	124111812	7942	0.075
chr3	124649348	124649387	7943	0.075
chr3	124840344	124840383	7944	0.075
chr3	124887991	124888030	7945	0.075
chr3	125189569	125189608	7946	0.075
chr3	125305639	125305678	7947	0.075
chr3	125371347	125371386	7948	0.075
chr3	125469207	125469246	7949	0.075
chr3	126849485	126849524	7950	0.075

chr3	128360756	128360795	7951	0.075
chr3	128769432	128769471	7952	0.075
chr3	129254394	129254433	7953	0.075
chr3	129474453	129474492	7954	0.075
chr3	129828294	129828333	7955	0.075
chr3	129968134	129968173	7956	0.075
chr3	130015036	130015075	7957	0.075
chr3	130242414	130242453	7958	0.075
chr3	130814836	130814875	7959	0.075
chr3	132556175	132556214	7960	0.075
chr3	13336013	13336052	7961	0.075
chr3	13412865	13412904	7962	0.075
chr3	136308007	136308046	7963	0.075
chr3	136962239	136962278	7964	0.075
chr3	137653669	137653708	7965	0.075
chr3	138239481	138239520	7966	0.075
chr3	138493022	138493061	7967	0.075
chr3	138678843	138678882	7968	0.075
chr3	139942027	139942066	7969	0.075
chr3	141406409	141406448	7970	0.075
chr3	14144859	14144898	7971	0.075
chr3	141602409	141602448	7972	0.075
chr3	141706227	141706266	7973	0.075
chr3	141706326	141706365	7974	0.075
chr3	142103903	142103942	7975	0.075
chr3	142252785	142252824	7976	0.075
chr3	147235204	147235243	7977	0.075
chr3	148040717	148040756	7978	0.075
chr3	148412979	148413018	7979	0.075
chr3	148611695	148611734	7980	0.075
chr3	14915556	14915595	7981	0.075
chr3	151959836	151959875	7982	0.075
chr3	152318740	152318779	7983	0.075
chr3	152476388	152476427	7984	0.075
chr3	152508571	152508610	7985	0.075
chr3	156167664	156167703	7986	0.075
chr3	157164392	157164431	7987	0.075
chr3	159113187	159113226	7988	0.075
chr3	159755666	159755705	7989	0.075
chr3	160976263	160976302	7990	0.075
chr3	161470151	161470190	7991	0.075
chr3	16194955	16194994	7992	0.075
chr3	162760755	162760794	7993	0.075
chr3	162769179	162769218	7994	0.075
chr3	16502587	16502626	7995	0.075
chr3	16541913	16541952	7996	0.075
chr3	166388410	166388449	7997	0.075
chr3	166397168	166397207	7998	0.075
chr3	167128557	167128596	7999	0.075
chr3	167370708	167370747	8000	0.075

chr3	170864170	170864209	8001	0.075
chr3	171042690	171042729	8002	0.075
chr3	171303363	171303402	8003	0.075
chr3	171643057	171643096	8004	0.075
chr3	171693585	171693624	8005	0.075
chr3	171744609	171744648	8006	0.075
chr3	172327239	172327278	8007	0.075
chr3	172885014	172885053	8008	0.075
chr3	17364730	17364769	8009	0.075
chr3	17380235	17380274	8010	0.075
chr3	174155595	174155634	8011	0.075
chr3	175276140	175276179	8012	0.075
chr3	176371834	176371873	8013	0.075
chr3	179121293	179121332	8014	0.075
chr3	18007996	18008035	8015	0.075
chr3	182712918	182712957	8016	0.075
chr3	183223838	183223877	8017	0.075
chr3	183934803	183934842	8018	0.075
chr3	185088724	185088763	8019	0.075
chr3	185120776	185120815	8020	0.075
chr3	1853537	1853576	8021	0.075
chr3	185383293	185383332	8022	0.075
chr3	186618206	186618245	8023	0.075
chr3	187266379	187266418	8024	0.075
chr3	187464686	187464725	8025	0.075
chr3	187608282	187608321	8026	0.075
chr3	187631406	187631445	8027	0.075
chr3	189721382	189721421	8028	0.075
chr3	19094241	19094280	8029	0.075
chr3	191163199	191163238	8030	0.075
chr3	191183568	191183607	8031	0.075
chr3	191692560	191692599	8032	0.075
chr3	193957228	193957267	8033	0.075
chr3	194847629	194847668	8034	0.075
chr3	194958440	194958479	8035	0.075
chr3	195371470	195371509	8036	0.075
chr3	198158351	198158390	8037	0.075
chr3	198287844	198287883	8038	0.075
chr3	198583866	198583905	8039	0.075
chr3	198605902	198605941	8040	0.075
chr3	198668102	198668141	8041	0.075
chr3	199367795	199367834	8042	0.075
chr3	20502382	20502421	8043	0.075
chr3	21643393	21643432	8044	0.075
chr3	21765557	21765596	8045	0.075
chr3	22721073	22721112	8046	0.075
chr3	24661656	24661695	8047	0.075
chr3	25017142	25017181	8048	0.075
chr3	25580904	25580943	8049	0.075
chr3	26591132	26591171	8050	0.075

chr3	26642629	26642668	8051	0.075
chr3	28607459	28607498	8052	0.075
chr3	29298924	29298963	8053	0.075
chr3	30521998	30522037	8054	0.075
chr3	30933882	30933921	8055	0.075
chr3	31080131	31080170	8056	0.075
chr3	32376916	32376955	8057	0.075
chr3	34573782	34573821	8058	0.075
chr3	35218085	35218124	8059	0.075
chr3	37236684	37236723	8060	0.075
chr3	37802451	37802490	8061	0.075
chr3	38969926	38969965	8062	0.075
chr3	39698635	39698674	8063	0.075
chr3	40344763	40344802	8064	0.075
chr3	40640747	40640786	8065	0.075
chr3	41648552	41648591	8066	0.075
chr3	42713360	42713399	8067	0.075
chr3	43367157	43367196	8068	0.075
chr3	43707281	43707320	8069	0.075
chr3	45092770	45092809	8070	0.075
chr3	45287940	45287979	8071	0.075
chr3	45982100	45982139	8072	0.075
chr3	47006017	47006056	8073	0.075
chr3	47282682	47282721	8074	0.075
chr3	47426598	47426637	8075	0.075
chr3	47819680	47819719	8076	0.075
chr3	48484916	48484955	8077	0.075
chr3	48729628	48729667	8078	0.075
chr3	48792691	48792730	8079	0.075
chr3	48836311	48836350	8080	0.075
chr3	49117141	49117180	8081	0.075
chr3	49318277	49318316	8082	0.075
chr3	49370042	49370121	8083	0.075
chr3	49539208	49539247	8084	0.075
chr3	52062030	52062069	8085	0.075
chr3	5218574	5218613	8086	0.075
chr3	52743788	52743827	8087	0.075
chr3	53427623	53427662	8088	0.075
chr3	53731026	53731065	8089	0.075
chr3	54086309	54086348	8090	0.075
chr3	55027359	55027398	8091	0.075
chr3	57799103	57799142	8092	0.075
chr3	58003787	58003826	8093	0.075
chr3	58084002	58084041	8094	0.075
chr3	58109042	58109081	8095	0.075
chr3	58183927	58183966	8096	0.075
chr3	58489543	58489582	8097	0.075
chr3	59103567	59103606	8098	0.075
chr3	59467827	59467866	8099	0.075
chr3	6040531	6040570	8100	0.075

chr3	6087381	6087420	8101	0.075
chr3	60990281	60990320	8102	0.075
chr3	61162326	61162365	8103	0.075
chr3	61691458	61691497	8104	0.075
chr3	61997279	61997318	8105	0.075
chr3	63146325	63146364	8106	0.075
chr3	64832558	64832597	8107	0.075
chr3	65031077	65031116	8108	0.075
chr3	65068053	65068092	8109	0.075
chr3	65351873	65351912	8110	0.075
chr3	65593054	65593093	8111	0.075
chr3	65667033	65667072	8112	0.075
chr3	66050226	66050265	8113	0.075
chr3	66756058	66756097	8114	0.075
chr3	67161548	67161587	8115	0.075
chr3	67610402	67610441	8116	0.075
chr3	68360305	68360344	8117	0.075
chr3	68678950	68678989	8118	0.075
chr3	68838254	68838293	8119	0.075
chr3	69870062	69870101	8120	0.075
chr3	70800927	70800966	8121	0.075
chr3	71257323	71257362	8122	0.075
chr3	71336864	71336903	8123	0.075
chr3	71584378	71584417	8124	0.075
chr3	71827898	71827937	8125	0.075
chr3	73205234	73205273	8126	0.075
chr3	73250988	73251027	8127	0.075
chr3	7595350	7595389	8128	0.075
chr3	75984943	75984982	8129	0.075
chr3	77212123	77212162	8130	0.075
chr3	77442359	77442398	8131	0.075
chr3	78213654	78213693	8132	0.075
chr3	78512600	78512639	8133	0.075
chr3	78820405	78820444	8134	0.075
chr3	79738554	79738593	8135	0.075
chr3	81901565	81901604	8136	0.075
chr3	82199804	82199843	8137	0.075
chr3	82687006	82687045	8138	0.075
chr3	84107646	84107685	8139	0.075
chr3	86202577	86202616	8140	0.075
chr3	87010320	87010359	8141	0.075
chr3	87302582	87302621	8142	0.075
chr3	88696369	88696408	8143	0.075
chr3	89515386	89515425	8144	0.075
chr3	89563089	89563128	8145	0.075
chr3	9069198	9069237	8146	0.075
chr3	9394543	9394582	8147	0.075
chr3	95242025	95242064	8148	0.075
chr3	9734852	9734891	8149	0.075
chr3	9808076	9808115	8150	0.075

chr3	9823492	9823531	8151	0.075
chr3	98403108	98403147	8152	0.075
chr3	99165765	99165804	8153	0.075
chr3	9961478	9961517	8154	0.075
chr4	31864728	31864834	8155	0.037383178
chr4	13679578	13679683	8156	0.037735849
chr4	93432988	93433092	8157	0.038095238
chr4	88962124	88962221	8158	0.040816327
chr4	955619	955710	8159	0.043478261
chr4	186662891	186662972	8160	0.048780488
chr4	10180056	10180093	8161	0.052631579
chr4	102266532	102266569	8162	0.052631579
chr4	102348715	102348752	8163	0.052631579
chr4	102840785	102840822	8164	0.052631579
chr4	103396119	103396156	8165	0.052631579
chr4	103744175	103744212	8166	0.052631579
chr4	104859424	104859461	8167	0.052631579
chr4	106319404	106319441	8168	0.052631579
chr4	107442926	107442963	8169	0.052631579
chr4	107748106	107748143	8170	0.052631579
chr4	107748929	107748966	8171	0.052631579
chr4	10805247	10805284	8172	0.052631579
chr4	109011319	109011356	8173	0.052631579
chr4	109130597	109130634	8174	0.052631579
chr4	109310930	109310967	8175	0.052631579
chr4	109400527	109400564	8176	0.052631579
chr4	109966551	109966588	8177	0.052631579
chr4	110426752	110426789	8178	0.052631579
chr4	110734866	110734903	8179	0.052631579
chr4	11116003	11116040	8180	0.052631579
chr4	111208986	111209023	8181	0.052631579
chr4	11132347	11132384	8182	0.052631579
chr4	11289777	11289814	8183	0.052631579
chr4	11290660	11290697	8184	0.052631579
chr4	113290533	113290570	8185	0.052631579
chr4	113343197	113343234	8186	0.052631579
chr4	113665616	113665653	8187	0.052631579
chr4	113790104	113790141	8188	0.052631579
chr4	114064570	114064607	8189	0.052631579
chr4	114404680	114404717	8190	0.052631579
chr4	11665216	11665253	8191	0.052631579
chr4	116735320	116735357	8192	0.052631579
chr4	11681689	11681726	8193	0.052631579
chr4	11731949	11731986	8194	0.052631579
chr4	11773845	11773882	8195	0.052631579
chr4	118073453	118073490	8196	0.052631579
chr4	118328436	118328473	8197	0.052631579
chr4	118381173	118381210	8198	0.052631579
chr4	118599392	118599429	8199	0.052631579
chr4	118636209	118636246	8200	0.052631579

chr4	119146448	119146485	8201	0.052631579
chr4	119371471	119371508	8202	0.052631579
chr4	1199799	1199836	8203	0.052631579
chr4	119987678	119987715	8204	0.052631579
chr4	120119458	120119495	8205	0.052631579
chr4	120150645	120150682	8206	0.052631579
chr4	120228290	120228327	8207	0.052631579
chr4	120389767	120389804	8208	0.052631579
chr4	121404293	121404330	8209	0.052631579
chr4	121835847	121835884	8210	0.052631579
chr4	121970722	121970759	8211	0.052631579
chr4	122342076	122342113	8212	0.052631579
chr4	122677396	122677433	8213	0.052631579
chr4	122677684	122677721	8214	0.052631579
chr4	122810244	122810281	8215	0.052631579
chr4	122867998	122868035	8216	0.052631579
chr4	123490135	123490172	8217	0.052631579
chr4	123555876	123555913	8218	0.052631579
chr4	124253627	124253664	8219	0.052631579
chr4	124358055	124358092	8220	0.052631579
chr4	124376868	124376905	8221	0.052631579
chr4	124414945	124414982	8222	0.052631579
chr4	124661444	124661481	8223	0.052631579
chr4	124728853	124728890	8224	0.052631579
chr4	125138736	125138773	8225	0.052631579
chr4	125487141	125487178	8226	0.052631579
chr4	125772579	125772616	8227	0.052631579
chr4	125819730	125819767	8228	0.052631579
chr4	12608887	12608924	8229	0.052631579
chr4	127196480	127196517	8230	0.052631579
chr4	127939256	127939293	8231	0.052631579
chr4	128468858	128468895	8232	0.052631579
chr4	128523733	128523770	8233	0.052631579
chr4	128612217	128612254	8234	0.052631579
chr4	128621567	128621604	8235	0.052631579
chr4	129060268	129060305	8236	0.052631579
chr4	129138201	129138238	8237	0.052631579
chr4	129282744	129282781	8238	0.052631579
chr4	129283519	129283556	8239	0.052631579
chr4	129376256	129376293	8240	0.052631579
chr4	130071893	130071930	8241	0.052631579
chr4	130485815	130485852	8242	0.052631579
chr4	130496116	130496153	8243	0.052631579
chr4	130500465	130500502	8244	0.052631579
chr4	130842591	130842628	8245	0.052631579
chr4	131170955	131170992	8246	0.052631579
chr4	13211660	13211697	8247	0.052631579
chr4	132390238	132390275	8248	0.052631579
chr4	133385555	133385592	8249	0.052631579
chr4	133402051	133402088	8250	0.052631579

chr4	133553304	133553341	8251	0.052631579
chr4	133964486	133964523	8252	0.052631579
chr4	133964601	133964638	8253	0.052631579
chr4	134030827	134030864	8254	0.052631579
chr4	134115965	134116002	8255	0.052631579
chr4	134298421	134298458	8256	0.052631579
chr4	134753527	134753564	8257	0.052631579
chr4	134767542	134767579	8258	0.052631579
chr4	136356164	136356201	8259	0.052631579
chr4	136631430	136631467	8260	0.052631579
chr4	137003517	137003554	8261	0.052631579
chr4	137114471	137114508	8262	0.052631579
chr4	140038927	140038964	8263	0.052631579
chr4	140083326	140083363	8264	0.052631579
chr4	140217118	140217155	8265	0.052631579
chr4	141204910	141204947	8266	0.052631579
chr4	141265405	141265442	8267	0.052631579
chr4	141878680	141878717	8268	0.052631579
chr4	141929635	141929672	8269	0.052631579
chr4	143348693	143348730	8270	0.052631579
chr4	144556151	144556188	8271	0.052631579
chr4	144675485	144675522	8272	0.052631579
chr4	144922352	144922389	8273	0.052631579
chr4	145169567	145169604	8274	0.052631579
chr4	146623657	146623694	8275	0.052631579
chr4	146623773	146623810	8276	0.052631579
chr4	146742809	146742846	8277	0.052631579
chr4	14677499	14677536	8278	0.052631579
chr4	146915202	146915239	8279	0.052631579
chr4	147170213	147170250	8280	0.052631579
chr4	147418782	147418819	8281	0.052631579
chr4	147465920	147465957	8282	0.052631579
chr4	147962237	147962274	8283	0.052631579
chr4	148076485	148076522	8284	0.052631579
chr4	148310897	148310934	8285	0.052631579
chr4	148502314	148502351	8286	0.052631579
chr4	14864486	14864523	8287	0.052631579
chr4	149697871	149697908	8288	0.052631579
chr4	149940426	149940463	8289	0.052631579
chr4	150154883	150154920	8290	0.052631579
chr4	150304666	150304703	8291	0.052631579
chr4	150635846	150635883	8292	0.052631579
chr4	1506750	1506787	8293	0.052631579
chr4	150823597	150823634	8294	0.052631579
chr4	151265002	151265039	8295	0.052631579
chr4	151310974	151311011	8296	0.052631579
chr4	151687242	151687279	8297	0.052631579
chr4	151820993	151821030	8298	0.052631579
chr4	152317510	152317547	8299	0.052631579
chr4	152390294	152390331	8300	0.052631579

chr4	152624899	152624936	8301	0.052631579
chr4	152697655	152697692	8302	0.052631579
chr4	152812195	152812232	8303	0.052631579
chr4	153296485	153296522	8304	0.052631579
chr4	153658373	153658410	8305	0.052631579
chr4	153668765	153668802	8306	0.052631579
chr4	153802370	153802407	8307	0.052631579
chr4	154690880	154690917	8308	0.052631579
chr4	154788673	154788710	8309	0.052631579
chr4	15495391	15495428	8310	0.052631579
chr4	155103892	155103929	8311	0.052631579
chr4	155156604	155156641	8312	0.052631579
chr4	155434961	155434998	8313	0.052631579
chr4	155566274	155566311	8314	0.052631579
chr4	155820714	155820751	8315	0.052631579
chr4	156428217	156428254	8316	0.052631579
chr4	156428712	156428749	8317	0.052631579
chr4	15740463	15740500	8318	0.052631579
chr4	157725022	157725059	8319	0.052631579
chr4	158482036	158482073	8320	0.052631579
chr4	158598385	158598422	8321	0.052631579
chr4	158979262	158979299	8322	0.052631579
chr4	159133774	159133811	8323	0.052631579
chr4	159771552	159771589	8324	0.052631579
chr4	159772307	159772344	8325	0.052631579
chr4	160150643	160150680	8326	0.052631579
chr4	160538730	160538767	8327	0.052631579
chr4	160539612	160539649	8328	0.052631579
chr4	161111301	161111338	8329	0.052631579
chr4	162330500	162330537	8330	0.052631579
chr4	162422601	162422638	8331	0.052631579
chr4	164754478	164754515	8332	0.052631579
chr4	165022587	165022624	8333	0.052631579
chr4	165499500	165499537	8334	0.052631579
chr4	165833810	165833847	8335	0.052631579
chr4	166252437	166252474	8336	0.052631579
chr4	166253040	166253077	8337	0.052631579
chr4	166610363	166610400	8338	0.052631579
chr4	167371355	167371392	8339	0.052631579
chr4	167493496	167493533	8340	0.052631579
chr4	168551274	168551311	8341	0.052631579
chr4	169095925	169095962	8342	0.052631579
chr4	169671089	169671126	8343	0.052631579
chr4	169671906	169671943	8344	0.052631579
chr4	169821965	169822002	8345	0.052631579
chr4	170031947	170031984	8346	0.052631579
chr4	171005781	171005818	8347	0.052631579
chr4	171006367	171006404	8348	0.052631579
chr4	171083815	171083852	8349	0.052631579
chr4	171149625	171149662	8350	0.052631579

chr4	171725843	171725880	8351	0.052631579
chr4	172268943	172268980	8352	0.052631579
chr4	1726926	1726963	8353	0.052631579
chr4	17274916	17274953	8354	0.052631579
chr4	172842318	172842355	8355	0.052631579
chr4	173202590	173202627	8356	0.052631579
chr4	173525103	173525140	8357	0.052631579
chr4	174026627	174026664	8358	0.052631579
chr4	174293869	174293906	8359	0.052631579
chr4	174494773	174494810	8360	0.052631579
chr4	174498011	174498048	8361	0.052631579
chr4	174673066	174673103	8362	0.052631579
chr4	174673921	174673958	8363	0.052631579
chr4	177092966	177093003	8364	0.052631579
chr4	177169234	177169271	8365	0.052631579
chr4	177350588	177350625	8366	0.052631579
chr4	1777156	1777193	8367	0.052631579
chr4	177717443	177717480	8368	0.052631579
chr4	177799297	177799334	8369	0.052631579
chr4	178201458	178201495	8370	0.052631579
chr4	178202070	178202107	8371	0.052631579
chr4	178529851	178529888	8372	0.052631579
chr4	179109659	179109696	8373	0.052631579
chr4	179110449	179110486	8374	0.052631579
chr4	179126347	179126384	8375	0.052631579
chr4	179518911	179518948	8376	0.052631579
chr4	179519633	179519670	8377	0.052631579
chr4	179909226	179909263	8378	0.052631579
chr4	180449681	180449718	8379	0.052631579
chr4	181697805	181697842	8380	0.052631579
chr4	182741149	182741186	8381	0.052631579
chr4	182807457	182807494	8382	0.052631579
chr4	182893626	182893663	8383	0.052631579
chr4	182893710	182893747	8384	0.052631579
chr4	183209101	183209138	8385	0.052631579
chr4	183800056	183800093	8386	0.052631579
chr4	183996817	183996854	8387	0.052631579
chr4	184021789	184021826	8388	0.052631579
chr4	184188428	184188465	8389	0.052631579
chr4	184191141	184191178	8390	0.052631579
chr4	184568823	184568860	8391	0.052631579
chr4	184702852	184702889	8392	0.052631579
chr4	184702944	184702981	8393	0.052631579
chr4	184974119	184974156	8394	0.052631579
chr4	185175263	185175300	8395	0.052631579
chr4	185427562	185427599	8396	0.052631579
chr4	185507721	185507758	8397	0.052631579
chr4	187372686	187372723	8398	0.052631579
chr4	187410528	187410565	8399	0.052631579
chr4	187602862	187602899	8400	0.052631579

chr4	187806321	187806358	8401	0.052631579
chr4	188002604	188002641	8402	0.052631579
chr4	188313356	188313393	8403	0.052631579
chr4	188863108	188863145	8404	0.052631579
chr4	188863905	188863942	8405	0.052631579
chr4	189305164	189305201	8406	0.052631579
chr4	189610834	189610871	8407	0.052631579
chr4	189944707	189944744	8408	0.052631579
chr4	189977323	189977360	8409	0.052631579
chr4	190442909	190442946	8410	0.052631579
chr4	190617348	190617385	8411	0.052631579
chr4	190672542	190672579	8412	0.052631579
chr4	190694475	190694512	8413	0.052631579
chr4	191093931	191093968	8414	0.052631579
chr4	194356	194393	8415	0.052631579
chr4	1951011	1951048	8416	0.052631579
chr4	20027470	20027507	8417	0.052631579
chr4	21012092	21012129	8418	0.052631579
chr4	21324535	21324572	8419	0.052631579
chr4	21641677	21641714	8420	0.052631579
chr4	21670176	21670213	8421	0.052631579
chr4	22039480	22039517	8422	0.052631579
chr4	22060112	22060149	8423	0.052631579
chr4	22060754	22060791	8424	0.052631579
chr4	22077837	22077874	8425	0.052631579
chr4	22338545	22338582	8426	0.052631579
chr4	23651492	23651529	8427	0.052631579
chr4	23725310	23725347	8428	0.052631579
chr4	23756247	23756284	8429	0.052631579
chr4	23899139	23899176	8430	0.052631579
chr4	24443757	24443794	8431	0.052631579
chr4	25095303	25095340	8432	0.052631579
chr4	25441948	25441985	8433	0.052631579
chr4	25442062	25442099	8434	0.052631579
chr4	25687804	25687841	8435	0.052631579
chr4	25688166	25688203	8436	0.052631579
chr4	25838329	25838366	8437	0.052631579
chr4	26809369	26809406	8438	0.052631579
chr4	26927431	26927468	8439	0.052631579
chr4	27033854	27033891	8440	0.052631579
chr4	27293863	27293900	8441	0.052631579
chr4	28619344	28619381	8442	0.052631579
chr4	29651043	29651080	8443	0.052631579
chr4	30009851	30009888	8444	0.052631579
chr4	30334954	30334991	8445	0.052631579
chr4	30553771	30553808	8446	0.052631579
chr4	30628346	30628383	8447	0.052631579
chr4	31005222	31005259	8448	0.052631579
chr4	3144565	3144602	8449	0.052631579
chr4	31783177	31783214	8450	0.052631579

chr4	32410684	32410721	8451	0.052631579
chr4	32411546	32411583	8452	0.052631579
chr4	32818681	32818718	8453	0.052631579
chr4	33106981	33107018	8454	0.052631579
chr4	3335799	3335836	8455	0.052631579
chr4	3336206	3336243	8456	0.052631579
chr4	34166982	34167019	8457	0.052631579
chr4	35005067	35005104	8458	0.052631579
chr4	35705107	35705144	8459	0.052631579
chr4	36156353	36156390	8460	0.052631579
chr4	36208913	36208950	8461	0.052631579
chr4	36329839	36329876	8462	0.052631579
chr4	36330313	36330350	8463	0.052631579
chr4	3674380	3674417	8464	0.052631579
chr4	369876	369913	8465	0.052631579
chr4	370021	370058	8466	0.052631579
chr4	37745874	37745911	8467	0.052631579
chr4	38004989	38005026	8468	0.052631579
chr4	38345301	38345338	8469	0.052631579
chr4	38583430	38583467	8470	0.052631579
chr4	40135083	40135120	8471	0.052631579
chr4	40518754	40518791	8472	0.052631579
chr4	40618764	40618801	8473	0.052631579
chr4	42092707	42092744	8474	0.052631579
chr4	42670036	42670073	8475	0.052631579
chr4	42705729	42705766	8476	0.052631579
chr4	43066525	43066562	8477	0.052631579
chr4	43122692	43122729	8478	0.052631579
chr4	44022457	44022494	8479	0.052631579
chr4	44454166	44454203	8480	0.052631579
chr4	45451124	45451161	8481	0.052631579
chr4	46765872	46765909	8482	0.052631579
chr4	48251074	48251111	8483	0.052631579
chr4	52604500	52604537	8484	0.052631579
chr4	52667614	52667651	8485	0.052631579
chr4	52754415	52754452	8486	0.052631579
chr4	53699445	53699482	8487	0.052631579
chr4	5410380	5410417	8488	0.052631579
chr4	54578096	54578133	8489	0.052631579
chr4	54612468	54612505	8490	0.052631579
chr4	54956613	54956650	8491	0.052631579
chr4	54978100	54978137	8492	0.052631579
chr4	57050639	57050676	8493	0.052631579
chr4	57099339	57099376	8494	0.052631579
chr4	57417451	57417488	8495	0.052631579
chr4	5761572	5761609	8496	0.052631579
chr4	57952096	57952133	8497	0.052631579
chr4	58300262	58300299	8498	0.052631579
chr4	58650436	58650473	8499	0.052631579
chr4	60202435	60202472	8500	0.052631579

chr4	60808677	60808714	8501	0.052631579
chr4	62348662	62348699	8502	0.052631579
chr4	62577284	62577321	8503	0.052631579
chr4	62592234	62592271	8504	0.052631579
chr4	62609128	62609165	8505	0.052631579
chr4	62810534	62810571	8506	0.052631579
chr4	63641347	63641384	8507	0.052631579
chr4	640707	640744	8508	0.052631579
chr4	6470252	6470289	8509	0.052631579
chr4	65038151	65038188	8510	0.052631579
chr4	65552193	65552230	8511	0.052631579
chr4	66080240	66080277	8512	0.052631579
chr4	66132339	66132376	8513	0.052631579
chr4	66346456	66346493	8514	0.052631579
chr4	66392844	66392881	8515	0.052631579
chr4	66494210	66494247	8516	0.052631579
chr4	66598872	66598909	8517	0.052631579
chr4	67063566	67063603	8518	0.052631579
chr4	67188407	67188444	8519	0.052631579
chr4	67416557	67416594	8520	0.052631579
chr4	67601165	67601202	8521	0.052631579
chr4	67717026	67717063	8522	0.052631579
chr4	68402739	68402776	8523	0.052631579
chr4	6857397	6857434	8524	0.052631579
chr4	696547	696584	8525	0.052631579
chr4	69811981	69812018	8526	0.052631579
chr4	70036053	70036090	8527	0.052631579
chr4	7036122	7036159	8528	0.052631579
chr4	7043836	7043873	8529	0.052631579
chr4	70892254	70892291	8530	0.052631579
chr4	7099596	7099633	8531	0.052631579
chr4	71199770	71199807	8532	0.052631579
chr4	71454450	71454487	8533	0.052631579
chr4	717478	717515	8534	0.052631579
chr4	71827505	71827542	8535	0.052631579
chr4	71862394	71862431	8536	0.052631579
chr4	71882493	71882530	8537	0.052631579
chr4	72200877	72200914	8538	0.052631579
chr4	72235340	72235377	8539	0.052631579
chr4	72351070	72351107	8540	0.052631579
chr4	72798995	72799032	8541	0.052631579
chr4	72887989	72888026	8542	0.052631579
chr4	73276339	73276376	8543	0.052631579
chr4	7352971	7353008	8544	0.052631579
chr4	74224383	74224420	8545	0.052631579
chr4	74261861	74261898	8546	0.052631579
chr4	74336011	74336048	8547	0.052631579
chr4	74492289	74492326	8548	0.052631579
chr4	75451330	75451367	8549	0.052631579
chr4	77272500	77272537	8550	0.052631579

chr4	77562203	77562240	8551	0.052631579
chr4	77939255	77939292	8552	0.052631579
chr4	78835351	78835388	8553	0.052631579
chr4	79510190	79510227	8554	0.052631579
chr4	79591998	79592035	8555	0.052631579
chr4	79963145	79963182	8556	0.052631579
chr4	80190756	80190793	8557	0.052631579
chr4	8097846	8097883	8558	0.052631579
chr4	81073422	81073459	8559	0.052631579
chr4	81095679	81095716	8560	0.052631579
chr4	81339567	81339604	8561	0.052631579
chr4	81784915	81784952	8562	0.052631579
chr4	82574149	82574186	8563	0.052631579
chr4	82679053	82679090	8564	0.052631579
chr4	82715324	82715361	8565	0.052631579
chr4	83120275	83120312	8566	0.052631579
chr4	83269693	83269730	8567	0.052631579
chr4	83480520	83480557	8568	0.052631579
chr4	83801826	83801863	8569	0.052631579
chr4	83850538	83850575	8570	0.052631579
chr4	8395770	8395807	8571	0.052631579
chr4	84079752	84079789	8572	0.052631579
chr4	84319025	84319062	8573	0.052631579
chr4	84319521	84319558	8574	0.052631579
chr4	8547648	8547685	8575	0.052631579
chr4	85599804	85599841	8576	0.052631579
chr4	85600550	85600587	8577	0.052631579
chr4	85762151	85762188	8578	0.052631579
chr4	85789702	85789739	8579	0.052631579
chr4	85826138	85826175	8580	0.052631579
chr4	85869026	85869063	8581	0.052631579
chr4	85885633	85885670	8582	0.052631579
chr4	85918956	85918993	8583	0.052631579
chr4	86505705	86505742	8584	0.052631579
chr4	8658757	8658794	8585	0.052631579
chr4	86934463	86934500	8586	0.052631579
chr4	87322513	87322550	8587	0.052631579
chr4	87449393	87449430	8588	0.052631579
chr4	88347820	88347857	8589	0.052631579
chr4	89033487	89033524	8590	0.052631579
chr4	89045244	89045281	8591	0.052631579
chr4	89206000	89206037	8592	0.052631579
chr4	89654286	89654323	8593	0.052631579
chr4	89882581	89882618	8594	0.052631579
chr4	8995262	8995299	8595	0.052631579
chr4	90380449	90380486	8596	0.052631579
chr4	90434259	90434296	8597	0.052631579
chr4	90434711	90434748	8598	0.052631579
chr4	915374	915411	8599	0.052631579
chr4	91580487	91580524	8600	0.052631579

chr4	915950	915987	8601	0.052631579
chr4	91868375	91868412	8602	0.052631579
chr4	92080363	92080400	8603	0.052631579
chr4	92590482	92590519	8604	0.052631579
chr4	92728446	92728483	8605	0.052631579
chr4	93104439	93104476	8606	0.052631579
chr4	93241490	93241527	8607	0.052631579
chr4	93842272	93842309	8608	0.052631579
chr4	94324607	94324644	8609	0.052631579
chr4	94430940	94430977	8610	0.052631579
chr4	95489086	95489123	8611	0.052631579
chr4	96003976	96004013	8612	0.052631579
chr4	96043729	96043766	8613	0.052631579
chr4	9622514	9622551	8614	0.052631579
chr4	96475707	96475744	8615	0.052631579
chr4	96496585	96496622	8616	0.052631579
chr4	96774344	96774381	8617	0.052631579
chr4	9685365	9685402	8618	0.052631579
chr4	96879807	96879844	8619	0.052631579
chr4	9740875	9740912	8620	0.052631579
chr4	9742741	9742778	8621	0.052631579
chr4	97754762	97754799	8622	0.052631579
chr4	97754847	97754884	8623	0.052631579
chr4	98197977	98198014	8624	0.052631579
chr4	98947257	98947294	8625	0.052631579
chr4	9898761	9898798	8626	0.052631579
chr4	73857999	73858069	8627	0.056338028
chr4	174657690	174657757	8628	0.058823529
chr4	16461334	16461398	8629	0.061538462
chr4	183526169	183526233	8630	0.061538462
chr4	100560902	100560941	8631	0.075
chr4	102487994	102488033	8632	0.075
chr4	106454074	106454113	8633	0.075
chr4	107190182	107190221	8634	0.075
chr4	110971714	110971753	8635	0.075
chr4	112271096	112271135	8636	0.075
chr4	115471705	115471744	8637	0.075
chr4	116946838	116946877	8638	0.075
chr4	117775019	117775058	8639	0.075
chr4	120099533	120099572	8640	0.075
chr4	121569674	121569713	8641	0.075
chr4	121753484	121753523	8642	0.075
chr4	121880655	121880694	8643	0.075
chr4	122844823	122844862	8644	0.075
chr4	123644091	123644130	8645	0.075
chr4	123820672	123820711	8646	0.075
chr4	12853274	12853313	8647	0.075
chr4	128657877	128657916	8648	0.075
chr4	128768167	128768206	8649	0.075
chr4	129474563	129474602	8650	0.075

chr4	129815911	129815950	8651	0.075
chr4	129943924	129943963	8652	0.075
chr4	130277656	130277695	8653	0.075
chr4	130942089	130942128	8654	0.075
chr4	131311764	131311803	8655	0.075
chr4	131613001	131613040	8656	0.075
chr4	132643835	132643874	8657	0.075
chr4	134964069	134964108	8658	0.075
chr4	137185711	137185750	8659	0.075
chr4	137487944	137487983	8660	0.075
chr4	138446460	138446499	8661	0.075
chr4	139602162	139602201	8662	0.075
chr4	140700579	140700618	8663	0.075
chr4	140982580	140982619	8664	0.075
chr4	141282320	141282359	8665	0.075
chr4	142168676	142168715	8666	0.075
chr4	142947605	142947644	8667	0.075
chr4	14413684	14413723	8668	0.075
chr4	14413833	14413872	8669	0.075
chr4	144357015	144357054	8670	0.075
chr4	146694565	146694604	8671	0.075
chr4	147338541	147338580	8672	0.075
chr4	147662418	147662457	8673	0.075
chr4	14861741	14861780	8674	0.075
chr4	149500504	149500543	8675	0.075
chr4	149918168	149918207	8676	0.075
chr4	152008422	152008461	8677	0.075
chr4	154480084	154480123	8678	0.075
chr4	154990593	154990632	8679	0.075
chr4	155907592	155907631	8680	0.075
chr4	15783211	15783250	8681	0.075
chr4	158504238	158504277	8682	0.075
chr4	159091518	159091557	8683	0.075
chr4	16088977	16089016	8684	0.075
chr4	162377827	162377866	8685	0.075
chr4	164883377	164883416	8686	0.075
chr4	166332882	166332921	8687	0.075
chr4	168938681	168938720	8688	0.075
chr4	169928209	169928248	8689	0.075
chr4	17364153	17364192	8690	0.075
chr4	174022085	174022124	8691	0.075
chr4	174213877	174213916	8692	0.075
chr4	177375041	177375080	8693	0.075
chr4	177480347	177480386	8694	0.075
chr4	17882488	17882527	8695	0.075
chr4	182658698	182658737	8696	0.075
chr4	183391898	183391937	8697	0.075
chr4	184660524	184660563	8698	0.075
chr4	185412565	185412604	8699	0.075
chr4	185575757	185575796	8700	0.075

chr4	188005214	188005253	8701	0.075
chr4	189155020	189155059	8702	0.075
chr4	19546457	19546496	8703	0.075
chr4	21546426	21546465	8704	0.075
chr4	2227267	2227306	8705	0.075
chr4	24722876	24722915	8706	0.075
chr4	30539121	30539160	8707	0.075
chr4	32867766	32867805	8708	0.075
chr4	33798036	33798075	8709	0.075
chr4	33800213	33800252	8710	0.075
chr4	35414055	35414094	8711	0.075
chr4	35607240	35607279	8712	0.075
chr4	36283241	36283280	8713	0.075
chr4	36742371	36742410	8714	0.075
chr4	36912973	36913012	8715	0.075
chr4	38372870	38372909	8716	0.075
chr4	38916217	38916256	8717	0.075
chr4	39376691	39376730	8718	0.075
chr4	41815333	41815372	8719	0.075
chr4	42160467	42160506	8720	0.075
chr4	43106988	43107027	8721	0.075
chr4	44913615	44913654	8722	0.075
chr4	47098861	47098900	8723	0.075
chr4	47106899	47106938	8724	0.075
chr4	48168594	48168633	8725	0.075
chr4	48527136	48527175	8726	0.075
chr4	48599356	48599395	8727	0.075
chr4	511581	511620	8728	0.075
chr4	52441351	52441390	8729	0.075
chr4	52849294	52849333	8730	0.075
chr4	52993360	52993399	8731	0.075
chr4	53335813	53335852	8732	0.075
chr4	53497531	53497570	8733	0.075
chr4	54088648	54088687	8734	0.075
chr4	54325309	54325348	8735	0.075
chr4	5434030	5434069	8736	0.075
chr4	55402542	55402581	8737	0.075
chr4	5642737	5642776	8738	0.075
chr4	58554432	58554471	8739	0.075
chr4	60052620	60052659	8740	0.075
chr4	60197640	60197679	8741	0.075
chr4	60274124	60274163	8742	0.075
chr4	61143451	61143490	8743	0.075
chr4	61405406	61405445	8744	0.075
chr4	64250229	64250268	8745	0.075
chr4	6428446	6428485	8746	0.075
chr4	65075154	65075193	8747	0.075
chr4	65100199	65100238	8748	0.075
chr4	68853600	68853639	8749	0.075
chr4	70740231	70740270	8750	0.075

chr4	71326293	71326332	8751	0.075
chr4	7171969	7172008	8752	0.075
chr4	72195395	72195434	8753	0.075
chr4	72453670	72453709	8754	0.075
chr4	73285969	73286008	8755	0.075
chr4	738807	738846	8756	0.075
chr4	74184917	74184956	8757	0.075
chr4	74517223	74517262	8758	0.075
chr4	7527134	7527173	8759	0.075
chr4	75424224	75424263	8760	0.075
chr4	75565647	75565686	8761	0.075
chr4	77120388	77120427	8762	0.075
chr4	77836219	77836258	8763	0.075
chr4	78077121	78077160	8764	0.075
chr4	7814314	7814353	8765	0.075
chr4	78524879	78524918	8766	0.075
chr4	79310978	79311017	8767	0.075
chr4	8289269	8289308	8768	0.075
chr4	83625658	83625697	8769	0.075
chr4	8408997	8409036	8770	0.075
chr4	85181109	85181148	8771	0.075
chr4	85652741	85652780	8772	0.075
chr4	882788	882827	8773	0.075
chr4	88282089	88282128	8774	0.075
chr4	89571078	89571117	8775	0.075
chr4	902583	902622	8776	0.075
chr4	9219437	9219476	8777	0.075
chr4	96439023	96439062	8778	0.075
chr4	96682354	96682393	8779	0.075
chr4	98020939	98020978	8780	0.075
chr4	9819638	9819677	8781	0.075
chr4	8106137	8106175	8782	0.076923077
chr5	129076928	129077024	8783	0.030927835
chr5	51099857	51099965	8784	0.036697248
chr5	136800743	136800848	8785	0.037735849
chr5	161366936	161367037	8786	0.039215686
chr5	73066087	73066188	8787	0.039215686
chr5	135413143	135413240	8788	0.040816327
chr5	90141518	90141609	8789	0.043478261
chr5	10009472	10009509	8790	0.052631579
chr5	100242928	100242965	8791	0.052631579
chr5	100248574	100248611	8792	0.052631579
chr5	100265445	100265482	8793	0.052631579
chr5	100992896	100992933	8794	0.052631579
chr5	101266420	101266457	8795	0.052631579
chr5	101916701	101916738	8796	0.052631579
chr5	102785394	102785431	8797	0.052631579
chr5	102829946	102829983	8798	0.052631579
chr5	104097140	104097177	8799	0.052631579
chr5	104816922	104816959	8800	0.052631579

chr5	105337355	105337392	8801	0.052631579
chr5	10536111	10536148	8802	0.052631579
chr5	105508316	105508353	8803	0.052631579
chr5	106326176	106326213	8804	0.052631579
chr5	106736949	106736986	8805	0.052631579
chr5	10720564	10720601	8806	0.052631579
chr5	107526749	107526786	8807	0.052631579
chr5	107861079	107861116	8808	0.052631579
chr5	107928879	107928916	8809	0.052631579
chr5	108269026	108269063	8810	0.052631579
chr5	109451600	109451637	8811	0.052631579
chr5	10986111	10986148	8812	0.052631579
chr5	10998591	10998628	8813	0.052631579
chr5	110495970	110496007	8814	0.052631579
chr5	110884552	110884589	8815	0.052631579
chr5	111040559	111040596	8816	0.052631579
chr5	111737667	111737704	8817	0.052631579
chr5	112154917	112154954	8818	0.052631579
chr5	11223246	11223283	8819	0.052631579
chr5	112794471	112794508	8820	0.052631579
chr5	112911571	112911608	8821	0.052631579
chr5	113501171	113501208	8822	0.052631579
chr5	114113696	114113733	8823	0.052631579
chr5	114490452	114490489	8824	0.052631579
chr5	114593230	114593267	8825	0.052631579
chr5	114908142	114908179	8826	0.052631579
chr5	115222942	115222979	8827	0.052631579
chr5	115284847	115284884	8828	0.052631579
chr5	115432893	115432930	8829	0.052631579
chr5	11559261	11559298	8830	0.052631579
chr5	115842326	115842363	8831	0.052631579
chr5	115989731	115989768	8832	0.052631579
chr5	116661372	116661409	8833	0.052631579
chr5	11718779	11718816	8834	0.052631579
chr5	117461087	117461124	8835	0.052631579
chr5	11792045	11792082	8836	0.052631579
chr5	118975842	118975879	8837	0.052631579
chr5	119421558	119421595	8838	0.052631579
chr5	11950099	11950136	8839	0.052631579
chr5	119617522	119617559	8840	0.052631579
chr5	119772643	119772680	8841	0.052631579
chr5	120166370	120166407	8842	0.052631579
chr5	120710082	120710119	8843	0.052631579
chr5	121786781	121786818	8844	0.052631579
chr5	121814825	121814862	8845	0.052631579
chr5	122448854	122448891	8846	0.052631579
chr5	123034973	123035010	8847	0.052631579
chr5	123219093	123219130	8848	0.052631579
chr5	123446628	123446665	8849	0.052631579
chr5	123481215	123481252	8850	0.052631579

chr5	123496201	123496238	8851	0.052631579
chr5	123647129	123647166	8852	0.052631579
chr5	123673484	123673521	8853	0.052631579
chr5	124428152	124428189	8854	0.052631579
chr5	124428627	124428664	8855	0.052631579
chr5	124861480	124861517	8856	0.052631579
chr5	125019304	125019341	8857	0.052631579
chr5	125556267	125556304	8858	0.052631579
chr5	12583294	12583331	8859	0.052631579
chr5	126211630	126211667	8860	0.052631579
chr5	126310863	126310900	8861	0.052631579
chr5	126587661	126587698	8862	0.052631579
chr5	126967260	126967297	8863	0.052631579
chr5	127045290	127045327	8864	0.052631579
chr5	127125295	127125332	8865	0.052631579
chr5	127186555	127186592	8866	0.052631579
chr5	127716123	127716160	8867	0.052631579
chr5	127861553	127861590	8868	0.052631579
chr5	129175538	129175575	8869	0.052631579
chr5	129381725	129381762	8870	0.052631579
chr5	131571363	131571400	8871	0.052631579
chr5	131615440	131615477	8872	0.052631579
chr5	131658030	131658067	8873	0.052631579
chr5	132569277	132569314	8874	0.052631579
chr5	132605151	132605188	8875	0.052631579
chr5	133158022	133158059	8876	0.052631579
chr5	133372776	133372813	8877	0.052631579
chr5	133457822	133457859	8878	0.052631579
chr5	133504413	133504450	8879	0.052631579
chr5	133801969	133802006	8880	0.052631579
chr5	134288165	134288202	8881	0.052631579
chr5	134290636	134290673	8882	0.052631579
chr5	134715078	134715115	8883	0.052631579
chr5	13511898	13511935	8884	0.052631579
chr5	13512121	13512158	8885	0.052631579
chr5	135176724	135176761	8886	0.052631579
chr5	135409918	135409955	8887	0.052631579
chr5	135480213	135480250	8888	0.052631579
chr5	13561418	13561455	8889	0.052631579
chr5	135720605	135720642	8890	0.052631579
chr5	136357124	136357161	8891	0.052631579
chr5	136357341	136357378	8892	0.052631579
chr5	136628108	136628145	8893	0.052631579
chr5	136628299	136628336	8894	0.052631579
chr5	136739639	136739676	8895	0.052631579
chr5	137206325	137206362	8896	0.052631579
chr5	137445022	137445059	8897	0.052631579
chr5	137445556	137445593	8898	0.052631579
chr5	137828186	137828223	8899	0.052631579
chr5	137829011	137829048	8900	0.052631579

chr5	137882385	137882422	8901	0.052631579
chr5	138130023	138130060	8902	0.052631579
chr5	138398741	138398778	8903	0.052631579
chr5	138792169	138792206	8904	0.052631579
chr5	138875598	138875635	8905	0.052631579
chr5	138974658	138974695	8906	0.052631579
chr5	139004012	139004049	8907	0.052631579
chr5	13917178	13917215	8908	0.052631579
chr5	139253157	139253194	8909	0.052631579
chr5	139359582	139359619	8910	0.052631579
chr5	139474119	139474156	8911	0.052631579
chr5	139474223	139474260	8912	0.052631579
chr5	1394941	1394978	8913	0.052631579
chr5	13974846	13974883	8914	0.052631579
chr5	140244152	140244189	8915	0.052631579
chr5	140464140	140464177	8916	0.052631579
chr5	140482017	140482054	8917	0.052631579
chr5	140769768	140769805	8918	0.052631579
chr5	140864948	140864985	8919	0.052631579
chr5	140915748	140915785	8920	0.052631579
chr5	141000285	141000322	8921	0.052631579
chr5	141373800	141373837	8922	0.052631579
chr5	14153855	14153892	8923	0.052631579
chr5	141643772	141643809	8924	0.052631579
chr5	142103811	142103848	8925	0.052631579
chr5	142178635	142178672	8926	0.052631579
chr5	142179071	142179108	8927	0.052631579
chr5	14247374	14247411	8928	0.052631579
chr5	142547742	142547779	8929	0.052631579
chr5	142596233	142596270	8930	0.052631579
chr5	14259912	14259949	8931	0.052631579
chr5	142673770	142673807	8932	0.052631579
chr5	143829768	143829805	8933	0.052631579
chr5	144488229	144488266	8934	0.052631579
chr5	145065739	145065776	8935	0.052631579
chr5	145233882	145233919	8936	0.052631579
chr5	14530213	14530250	8937	0.052631579
chr5	145388001	145388038	8938	0.052631579
chr5	145395021	145395058	8939	0.052631579
chr5	14557638	14557675	8940	0.052631579
chr5	145849138	145849175	8941	0.052631579
chr5	146117700	146117737	8942	0.052631579
chr5	146212634	146212671	8943	0.052631579
chr5	146752705	146752742	8944	0.052631579
chr5	146753574	146753611	8945	0.052631579
chr5	14761230	14761267	8946	0.052631579
chr5	148414681	148414718	8947	0.052631579
chr5	148445036	148445073	8948	0.052631579
chr5	148665467	148665504	8949	0.052631579
chr5	148666171	148666208	8950	0.052631579

chr5	148877590	148877627	8951	0.052631579
chr5	149866386	149866423	8952	0.052631579
chr5	150142292	150142329	8953	0.052631579
chr5	150195842	150195879	8954	0.052631579
chr5	150255560	150255597	8955	0.052631579
chr5	150398299	150398336	8956	0.052631579
chr5	150530152	150530189	8957	0.052631579
chr5	150961217	150961254	8958	0.052631579
chr5	151006722	151006759	8959	0.052631579
chr5	151512543	151512580	8960	0.052631579
chr5	151513288	151513325	8961	0.052631579
chr5	15160810	15160847	8962	0.052631579
chr5	151823227	151823264	8963	0.052631579
chr5	152216748	152216785	8964	0.052631579
chr5	152620492	152620529	8965	0.052631579
chr5	15337813	15337850	8966	0.052631579
chr5	154176016	154176053	8967	0.052631579
chr5	155161993	155162030	8968	0.052631579
chr5	155324524	155324561	8969	0.052631579
chr5	15577499	15577536	8970	0.052631579
chr5	15584440	15584477	8971	0.052631579
chr5	155979380	155979417	8972	0.052631579
chr5	156154136	156154173	8973	0.052631579
chr5	156692932	156692969	8974	0.052631579
chr5	156880942	156880979	8975	0.052631579
chr5	156918657	156918694	8976	0.052631579
chr5	157302642	157302679	8977	0.052631579
chr5	157302778	157302815	8978	0.052631579
chr5	157382288	157382325	8979	0.052631579
chr5	157528352	157528389	8980	0.052631579
chr5	157733651	157733688	8981	0.052631579
chr5	157953441	157953478	8982	0.052631579
chr5	158102553	158102590	8983	0.052631579
chr5	158137007	158137044	8984	0.052631579
chr5	158156037	158156074	8985	0.052631579
chr5	158412482	158412519	8986	0.052631579
chr5	158545003	158545040	8987	0.052631579
chr5	158688796	158688833	8988	0.052631579
chr5	158849440	158849477	8989	0.052631579
chr5	158867597	158867634	8990	0.052631579
chr5	159440643	159440680	8991	0.052631579
chr5	159451652	159451689	8992	0.052631579
chr5	159829100	159829137	8993	0.052631579
chr5	1599001	1599038	8994	0.052631579
chr5	160607083	160607120	8995	0.052631579
chr5	160661634	160661671	8996	0.052631579
chr5	160662415	160662452	8997	0.052631579
chr5	160690613	160690650	8998	0.052631579
chr5	161082898	161082935	8999	0.052631579
chr5	161384118	161384155	9000	0.052631579

chr5	161497936	161497973	9001	0.052631579
chr5	161557206	161557243	9002	0.052631579
chr5	161576715	161576752	9003	0.052631579
chr5	161795248	161795285	9004	0.052631579
chr5	162124236	162124273	9005	0.052631579
chr5	162211287	162211324	9006	0.052631579
chr5	162374123	162374160	9007	0.052631579
chr5	162474047	162474084	9008	0.052631579
chr5	162661718	162661755	9009	0.052631579
chr5	163309713	163309750	9010	0.052631579
chr5	163367715	163367752	9011	0.052631579
chr5	163577841	163577878	9012	0.052631579
chr5	163839837	163839874	9013	0.052631579
chr5	163947611	163947648	9014	0.052631579
chr5	164788210	164788247	9015	0.052631579
chr5	165131683	165131720	9016	0.052631579
chr5	165993737	165993774	9017	0.052631579
chr5	166644497	166644534	9018	0.052631579
chr5	167280719	167280756	9019	0.052631579
chr5	16730106	16730143	9020	0.052631579
chr5	16740843	16740880	9021	0.052631579
chr5	168503585	168503622	9022	0.052631579
chr5	168586831	168586868	9023	0.052631579
chr5	16869579	16869616	9024	0.052631579
chr5	168765117	168765154	9025	0.052631579
chr5	168802227	168802264	9026	0.052631579
chr5	168932837	168932874	9027	0.052631579
chr5	169654166	169654203	9028	0.052631579
chr5	169848376	169848413	9029	0.052631579
chr5	170632413	170632450	9030	0.052631579
chr5	170633323	170633360	9031	0.052631579
chr5	170754264	170754301	9032	0.052631579
chr5	171548440	171548477	9033	0.052631579
chr5	171954551	171954588	9034	0.052631579
chr5	171955440	171955477	9035	0.052631579
chr5	172379934	172379971	9036	0.052631579
chr5	172552357	172552394	9037	0.052631579
chr5	172676862	172676899	9038	0.052631579
chr5	17307584	17307621	9039	0.052631579
chr5	173184570	173184607	9040	0.052631579
chr5	17346706	17346743	9041	0.052631579
chr5	173524506	173524543	9042	0.052631579
chr5	173524699	173524736	9043	0.052631579
chr5	173555664	173555701	9044	0.052631579
chr5	173951494	173951531	9045	0.052631579
chr5	174829496	174829533	9046	0.052631579
chr5	175241770	175241807	9047	0.052631579
chr5	175530386	175530423	9048	0.052631579
chr5	176228545	176228582	9049	0.052631579
chr5	176229245	176229282	9050	0.052631579

chr5	176490374	176490411	9051	0.052631579
chr5	176710799	176710836	9052	0.052631579
chr5	176806567	176806604	9053	0.052631579
chr5	176849651	176849688	9054	0.052631579
chr5	177625004	177625041	9055	0.052631579
chr5	177910945	177910982	9056	0.052631579
chr5	178129523	178129560	9057	0.052631579
chr5	178980950	178980987	9058	0.052631579
chr5	179063605	179063642	9059	0.052631579
chr5	179128634	179128671	9060	0.052631579
chr5	179148040	179148077	9061	0.052631579
chr5	179223688	179223725	9062	0.052631579
chr5	179453297	179453334	9063	0.052631579
chr5	179474099	179474136	9064	0.052631579
chr5	179707666	179707703	9065	0.052631579
chr5	179716442	179716479	9066	0.052631579
chr5	179924785	179924822	9067	0.052631579
chr5	179927564	179927601	9068	0.052631579
chr5	180481585	180481622	9069	0.052631579
chr5	190794	190831	9070	0.052631579
chr5	20386613	20386650	9071	0.052631579
chr5	204728	204765	9072	0.052631579
chr5	210315	210352	9073	0.052631579
chr5	21298017	21298054	9074	0.052631579
chr5	21898282	21898319	9075	0.052631579
chr5	2219704	2219741	9076	0.052631579
chr5	2229908	2229945	9077	0.052631579
chr5	22685871	22685908	9078	0.052631579
chr5	22716636	22716673	9079	0.052631579
chr5	24057855	24057892	9080	0.052631579
chr5	24198814	24198851	9081	0.052631579
chr5	24659254	24659291	9082	0.052631579
chr5	24980115	24980152	9083	0.052631579
chr5	25165570	25165607	9084	0.052631579
chr5	25766131	25766168	9085	0.052631579
chr5	26173656	26173693	9086	0.052631579
chr5	26271180	26271217	9087	0.052631579
chr5	26903126	26903163	9088	0.052631579
chr5	28054392	28054429	9089	0.052631579
chr5	28054672	28054709	9090	0.052631579
chr5	2920567	2920604	9091	0.052631579
chr5	30028855	30028892	9092	0.052631579
chr5	30029135	30029172	9093	0.052631579
chr5	30595242	30595279	9094	0.052631579
chr5	3061553	3061590	9095	0.052631579
chr5	31402971	31403008	9096	0.052631579
chr5	31448367	31448404	9097	0.052631579
chr5	31588286	31588323	9098	0.052631579
chr5	31858872	31858909	9099	0.052631579
chr5	32043199	32043236	9100	0.052631579

chr5	32162248	32162285	9101	0.052631579
chr5	32209829	32209866	9102	0.052631579
chr5	32426435	32426472	9103	0.052631579
chr5	32480527	32480564	9104	0.052631579
chr5	32743426	32743463	9105	0.052631579
chr5	33022582	33022619	9106	0.052631579
chr5	33678789	33678826	9107	0.052631579
chr5	34004451	34004488	9108	0.052631579
chr5	34622057	34622094	9109	0.052631579
chr5	34757275	34757312	9110	0.052631579
chr5	35030229	35030266	9111	0.052631579
chr5	35445922	35445959	9112	0.052631579
chr5	35520286	35520323	9113	0.052631579
chr5	35692474	35692511	9114	0.052631579
chr5	35899249	35899286	9115	0.052631579
chr5	35938377	35938414	9116	0.052631579
chr5	35945602	35945639	9117	0.052631579
chr5	3595845	3595882	9118	0.052631579
chr5	36430350	36430387	9119	0.052631579
chr5	36688139	36688176	9120	0.052631579
chr5	37056390	37056427	9121	0.052631579
chr5	37337273	37337310	9122	0.052631579
chr5	38039048	38039085	9123	0.052631579
chr5	38039764	38039801	9124	0.052631579
chr5	38081939	38081976	9125	0.052631579
chr5	38732584	38732621	9126	0.052631579
chr5	39176494	39176531	9127	0.052631579
chr5	39318980	39319017	9128	0.052631579
chr5	39319646	39319683	9129	0.052631579
chr5	39481405	39481442	9130	0.052631579
chr5	39539534	39539571	9131	0.052631579
chr5	3984932	3984969	9132	0.052631579
chr5	40022172	40022209	9133	0.052631579
chr5	40093275	40093312	9134	0.052631579
chr5	40791199	40791236	9135	0.052631579
chr5	4091586	4091623	9136	0.052631579
chr5	41287645	41287682	9137	0.052631579
chr5	41294981	41295018	9138	0.052631579
chr5	41342973	41343010	9139	0.052631579
chr5	41495681	41495718	9140	0.052631579
chr5	41651956	41651993	9141	0.052631579
chr5	42003959	42003996	9142	0.052631579
chr5	42397154	42397191	9143	0.052631579
chr5	42566288	42566325	9144	0.052631579
chr5	42625980	42626017	9145	0.052631579
chr5	42634894	42634931	9146	0.052631579
chr5	43549176	43549213	9147	0.052631579
chr5	43769691	43769728	9148	0.052631579
chr5	44367364	44367401	9149	0.052631579
chr5	44452742	44452779	9150	0.052631579

chr5	44649409	44649446	9151	0.052631579
chr5	4770114	4770151	9152	0.052631579
chr5	4785219	4785256	9153	0.052631579
chr5	50353118	50353155	9154	0.052631579
chr5	50375211	50375248	9155	0.052631579
chr5	50380496	50380533	9156	0.052631579
chr5	50616713	50616750	9157	0.052631579
chr5	50617562	50617599	9158	0.052631579
chr5	5076005	5076042	9159	0.052631579
chr5	50853022	50853059	9160	0.052631579
chr5	51896870	51896907	9161	0.052631579
chr5	51966816	51966853	9162	0.052631579
chr5	52235599	52235636	9163	0.052631579
chr5	52658398	52658435	9164	0.052631579
chr5	53232569	53232606	9165	0.052631579
chr5	53378997	53379034	9166	0.052631579
chr5	53771669	53771706	9167	0.052631579
chr5	54508183	54508220	9168	0.052631579
chr5	54807007	54807044	9169	0.052631579
chr5	555380	555417	9170	0.052631579
chr5	55700950	55700987	9171	0.052631579
chr5	55701680	55701717	9172	0.052631579
chr5	55741549	55741586	9173	0.052631579
chr5	5589592	5589629	9174	0.052631579
chr5	56180038	56180075	9175	0.052631579
chr5	56572686	56572723	9176	0.052631579
chr5	58058634	58058671	9177	0.052631579
chr5	58274371	58274408	9178	0.052631579
chr5	58307384	58307421	9179	0.052631579
chr5	58370482	58370519	9180	0.052631579
chr5	59824849	59824886	9181	0.052631579
chr5	60517030	60517067	9182	0.052631579
chr5	60583308	60583345	9183	0.052631579
chr5	60584118	60584155	9184	0.052631579
chr5	60620932	60620969	9185	0.052631579
chr5	60629702	60629739	9186	0.052631579
chr5	60950137	60950174	9187	0.052631579
chr5	61057896	61057933	9188	0.052631579
chr5	61232187	61232224	9189	0.052631579
chr5	6136500	6136537	9190	0.052631579
chr5	61530660	61530697	9191	0.052631579
chr5	61789319	61789356	9192	0.052631579
chr5	62371845	62371882	9193	0.052631579
chr5	62416733	62416770	9194	0.052631579
chr5	62451998	62452035	9195	0.052631579
chr5	6305107	6305144	9196	0.052631579
chr5	6355234	6355271	9197	0.052631579
chr5	63818002	63818039	9198	0.052631579
chr5	6388259	6388296	9199	0.052631579
chr5	63990427	63990464	9200	0.052631579

chr5	64688009	64688046	9201	0.052631579
chr5	6475495	6475532	9202	0.052631579
chr5	64780425	64780462	9203	0.052631579
chr5	64814368	64814405	9204	0.052631579
chr5	6487626	6487663	9205	0.052631579
chr5	6490577	6490614	9206	0.052631579
chr5	64943186	64943223	9207	0.052631579
chr5	65148289	65148326	9208	0.052631579
chr5	66497079	66497116	9209	0.052631579
chr5	66826220	66826257	9210	0.052631579
chr5	66977498	66977535	9211	0.052631579
chr5	67311502	67311539	9212	0.052631579
chr5	6752338	6752375	9213	0.052631579
chr5	67552253	67552290	9214	0.052631579
chr5	67553029	67553066	9215	0.052631579
chr5	67569112	67569149	9216	0.052631579
chr5	67598855	67598892	9217	0.052631579
chr5	67726270	67726307	9218	0.052631579
chr5	67951760	67951797	9219	0.052631579
chr5	68002679	68002716	9220	0.052631579
chr5	6896487	6896524	9221	0.052631579
chr5	70759659	70759696	9222	0.052631579
chr5	7119650	7119687	9223	0.052631579
chr5	7119935	7119972	9224	0.052631579
chr5	72341695	72341732	9225	0.052631579
chr5	72451678	72451715	9226	0.052631579
chr5	72830738	72830775	9227	0.052631579
chr5	72909294	72909331	9228	0.052631579
chr5	72912103	72912140	9229	0.052631579
chr5	73038537	73038574	9230	0.052631579
chr5	7332423	7332460	9231	0.052631579
chr5	73649463	73649500	9232	0.052631579
chr5	73650157	73650194	9233	0.052631579
chr5	73851006	73851043	9234	0.052631579
chr5	74131200	74131237	9235	0.052631579
chr5	74390190	74390227	9236	0.052631579
chr5	75175298	75175335	9237	0.052631579
chr5	75210293	75210330	9238	0.052631579
chr5	75527562	75527599	9239	0.052631579
chr5	75878526	75878563	9240	0.052631579
chr5	75932447	75932484	9241	0.052631579
chr5	76328724	76328761	9242	0.052631579
chr5	76930555	76930592	9243	0.052631579
chr5	77056940	77056977	9244	0.052631579
chr5	77134824	77134861	9245	0.052631579
chr5	77156431	77156468	9246	0.052631579
chr5	77206866	77206903	9247	0.052631579
chr5	77492210	77492247	9248	0.052631579
chr5	77546750	77546787	9249	0.052631579
chr5	77697402	77697439	9250	0.052631579

chr5	7772974	7773011	9251	0.052631579
chr5	77983949	77983986	9252	0.052631579
chr5	78103562	78103599	9253	0.052631579
chr5	78150584	78150621	9254	0.052631579
chr5	78779380	78779417	9255	0.052631579
chr5	79899593	79899630	9256	0.052631579
chr5	80093069	80093106	9257	0.052631579
chr5	80761704	80761741	9258	0.052631579
chr5	80762551	80762588	9259	0.052631579
chr5	80773543	80773580	9260	0.052631579
chr5	80967028	80967065	9261	0.052631579
chr5	81053401	81053438	9262	0.052631579
chr5	81053513	81053550	9263	0.052631579
chr5	81153947	81153984	9264	0.052631579
chr5	82148371	82148408	9265	0.052631579
chr5	82442710	82442747	9266	0.052631579
chr5	82562805	82562842	9267	0.052631579
chr5	82822891	82822928	9268	0.052631579
chr5	83226603	83226640	9269	0.052631579
chr5	83350052	83350089	9270	0.052631579
chr5	83396042	83396079	9271	0.052631579
chr5	83519717	83519754	9272	0.052631579
chr5	83892023	83892060	9273	0.052631579
chr5	83892491	83892528	9274	0.052631579
chr5	83911339	83911376	9275	0.052631579
chr5	84430508	84430545	9276	0.052631579
chr5	84648719	84648756	9277	0.052631579
chr5	86240005	86240042	9278	0.052631579
chr5	87193862	87193899	9279	0.052631579
chr5	8738952	8738989	9280	0.052631579
chr5	87445717	87445754	9281	0.052631579
chr5	87999524	87999561	9282	0.052631579
chr5	88720105	88720142	9283	0.052631579
chr5	89087390	89087427	9284	0.052631579
chr5	89276472	89276509	9285	0.052631579
chr5	89455817	89455854	9286	0.052631579
chr5	89458953	89458990	9287	0.052631579
chr5	89513150	89513187	9288	0.052631579
chr5	89558166	89558203	9289	0.052631579
chr5	89576385	89576422	9290	0.052631579
chr5	89946121	89946158	9291	0.052631579
chr5	90001362	90001399	9292	0.052631579
chr5	90203028	90203065	9293	0.052631579
chr5	90397712	90397749	9294	0.052631579
chr5	9050829	9050866	9295	0.052631579
chr5	90707104	90707141	9296	0.052631579
chr5	90865193	90865230	9297	0.052631579
chr5	91046458	91046495	9298	0.052631579
chr5	91075357	91075394	9299	0.052631579
chr5	91321880	91321917	9300	0.052631579

chr5	91322009	91322046	9301	0.052631579
chr5	91413935	91413972	9302	0.052631579
chr5	91415237	91415274	9303	0.052631579
chr5	91735216	91735253	9304	0.052631579
chr5	9221434	9221471	9305	0.052631579
chr5	92340385	92340422	9306	0.052631579
chr5	92489097	92489134	9307	0.052631579
chr5	92572391	92572428	9308	0.052631579
chr5	92802518	92802555	9309	0.052631579
chr5	92831485	92831522	9310	0.052631579
chr5	93253811	93253848	9311	0.052631579
chr5	93601455	93601492	9312	0.052631579
chr5	93601711	93601748	9313	0.052631579
chr5	93644567	93644604	9314	0.052631579
chr5	93729292	93729329	9315	0.052631579
chr5	94250225	94250262	9316	0.052631579
chr5	94253786	94253823	9317	0.052631579
chr5	9507958	9507995	9318	0.052631579
chr5	95493489	95493526	9319	0.052631579
chr5	95928319	95928356	9320	0.052631579
chr5	96183403	96183440	9321	0.052631579
chr5	96358889	96358926	9322	0.052631579
chr5	96861481	96861518	9323	0.052631579
chr5	9693825	9693862	9324	0.052631579
chr5	97244364	97244401	9325	0.052631579
chr5	97312343	97312380	9326	0.052631579
chr5	97728285	97728322	9327	0.052631579
chr5	98018748	98018785	9328	0.052631579
chr5	98136450	98136487	9329	0.052631579
chr5	98827857	98827894	9330	0.052631579
chr5	9887869	9887906	9331	0.052631579
chr5	9927206	9927243	9332	0.052631579
chr5	99294608	99294645	9333	0.052631579
chr5	38406976	38407044	9334	0.057971014
chr5	87279365	87279429	9335	0.061538462
chr5	54142828	54142890	9336	0.063492063
chr5	72048677	72048737	9337	0.06557377
chr5	132258879	132258936	9338	0.068965517
chr5	21841418	21841474	9339	0.070175439
chr5	153154423	153154463	9340	0.073170732
chr5	100001264	100001303	9341	0.075
chr5	102816167	102816206	9342	0.075
chr5	102886696	102886735	9343	0.075
chr5	103361377	103361416	9344	0.075
chr5	103384583	103384622	9345	0.075
chr5	10499155	10499194	9346	0.075
chr5	105648828	105648867	9347	0.075
chr5	105830841	105830880	9348	0.075
chr5	106548620	106548659	9349	0.075
chr5	106980597	106980636	9350	0.075

chr5	107306926	107306965	9351	0.075
chr5	10761015	10761054	9352	0.075
chr5	109815165	109815204	9353	0.075
chr5	110697795	110697834	9354	0.075
chr5	112065216	112065255	9355	0.075
chr5	114146658	114146697	9356	0.075
chr5	115765428	115765467	9357	0.075
chr5	115804280	115804319	9358	0.075
chr5	116311611	116311650	9359	0.075
chr5	117628454	117628493	9360	0.075
chr5	117860201	117860240	9361	0.075
chr5	118100325	118100364	9362	0.075
chr5	119321098	119321137	9363	0.075
chr5	119855532	119855571	9364	0.075
chr5	121988568	121988607	9365	0.075
chr5	122939383	122939422	9366	0.075
chr5	124091279	124091318	9367	0.075
chr5	127571026	127571065	9368	0.075
chr5	127627143	127627182	9369	0.075
chr5	129340848	129340887	9370	0.075
chr5	129393536	129393575	9371	0.075
chr5	129413815	129413854	9372	0.075
chr5	130107582	130107621	9373	0.075
chr5	130771709	130771748	9374	0.075
chr5	132256667	132256706	9375	0.075
chr5	134693376	134693415	9376	0.075
chr5	135427085	135427124	9377	0.075
chr5	135495648	135495687	9378	0.075
chr5	135980577	135980616	9379	0.075
chr5	137325818	137325857	9380	0.075
chr5	138642410	138642449	9381	0.075
chr5	139103198	139103237	9382	0.075
chr5	139154598	139154637	9383	0.075
chr5	139272906	139272945	9384	0.075
chr5	140181727	140181766	9385	0.075
chr5	140799302	140799341	9386	0.075
chr5	140996515	140996554	9387	0.075
chr5	141029426	141029465	9388	0.075
chr5	141145818	141145857	9389	0.075
chr5	141174618	141174657	9390	0.075
chr5	14125080	14125119	9391	0.075
chr5	141736003	141736042	9392	0.075
chr5	142140962	142141001	9393	0.075
chr5	142639675	142639714	9394	0.075
chr5	143232608	143232647	9395	0.075
chr5	14404423	14404462	9396	0.075
chr5	145185151	145185190	9397	0.075
chr5	14766949	14766988	9398	0.075
chr5	148654986	148655025	9399	0.075
chr5	148692532	148692571	9400	0.075

chr5	148872899	148872938	9401	0.075
chr5	149415781	149415820	9402	0.075
chr5	149807426	149807465	9403	0.075
chr5	150139070	150139109	9404	0.075
chr5	151027259	151027298	9405	0.075
chr5	151031362	151031401	9406	0.075
chr5	151169800	151169839	9407	0.075
chr5	151452030	151452069	9408	0.075
chr5	15332615	15332654	9409	0.075
chr5	153460568	153460607	9410	0.075
chr5	154159654	154159693	9411	0.075
chr5	154741822	154741861	9412	0.075
chr5	154933658	154933697	9413	0.075
chr5	155287759	155287798	9414	0.075
chr5	157668370	157668409	9415	0.075
chr5	157797490	157797529	9416	0.075
chr5	159179030	159179069	9417	0.075
chr5	159494799	159494838	9418	0.075
chr5	162206	162245	9419	0.075
chr5	16223231	16223270	9420	0.075
chr5	162308754	162308793	9421	0.075
chr5	163583547	163583586	9422	0.075
chr5	16409416	16409455	9423	0.075
chr5	164498054	164498093	9424	0.075
chr5	164761907	164761946	9425	0.075
chr5	166156080	166156119	9426	0.075
chr5	166503215	166503254	9427	0.075
chr5	166791120	166791159	9428	0.075
chr5	166873619	166873658	9429	0.075
chr5	167507213	167507252	9430	0.075
chr5	168551960	168551999	9431	0.075
chr5	169107671	169107710	9432	0.075
chr5	169302614	169302653	9433	0.075
chr5	169354103	169354142	9434	0.075
chr5	170095497	170095536	9435	0.075
chr5	170565170	170565209	9436	0.075
chr5	170671142	170671181	9437	0.075
chr5	172129205	172129244	9438	0.075
chr5	17300850	17300889	9439	0.075
chr5	173158396	173158435	9440	0.075
chr5	173468675	173468714	9441	0.075
chr5	173731118	173731157	9442	0.075
chr5	173918962	173919001	9443	0.075
chr5	174275411	174275450	9444	0.075
chr5	175973856	175973895	9445	0.075
chr5	176233730	176233769	9446	0.075
chr5	176424971	176425010	9447	0.075
chr5	177344175	177344214	9448	0.075
chr5	178149962	178150001	9449	0.075
chr5	178298879	178298918	9450	0.075

chr5	178420401	178420440	9451	0.075
chr5	179196078	179196117	9452	0.075
chr5	180157493	180157532	9453	0.075
chr5	180378448	180378487	9454	0.075
chr5	180544010	180544049	9455	0.075
chr5	1935041	1935080	9456	0.075
chr5	19540202	19540241	9457	0.075
chr5	19775296	19775335	9458	0.075
chr5	19990388	19990427	9459	0.075
chr5	21677913	21677952	9460	0.075
chr5	22187913	22187952	9461	0.075
chr5	22709689	22709728	9462	0.075
chr5	2310902	2310941	9463	0.075
chr5	24954617	24954656	9464	0.075
chr5	2692298	2692337	9465	0.075
chr5	27159164	27159203	9466	0.075
chr5	2724044	2724083	9467	0.075
chr5	29052924	29052963	9468	0.075
chr5	3150348	3150387	9469	0.075
chr5	31761701	31761740	9470	0.075
chr5	32989060	32989099	9471	0.075
chr5	33164869	33164908	9472	0.075
chr5	33444265	33444304	9473	0.075
chr5	34069189	34069228	9474	0.075
chr5	3565591	3565630	9475	0.075
chr5	35913755	35913794	9476	0.075
chr5	36294920	36294959	9477	0.075
chr5	36632138	36632177	9478	0.075
chr5	37379240	37379279	9479	0.075
chr5	37846495	37846534	9480	0.075
chr5	37910165	37910204	9481	0.075
chr5	38770811	38770850	9482	0.075
chr5	43347344	43347383	9483	0.075
chr5	43639240	43639279	9484	0.075
chr5	44424515	44424554	9485	0.075
chr5	44802172	44802211	9486	0.075
chr5	490864	490903	9487	0.075
chr5	4974665	4974704	9488	0.075
chr5	50291094	50291133	9489	0.075
chr5	50512489	50512528	9490	0.075
chr5	52497369	52497408	9491	0.075
chr5	52810593	52810632	9492	0.075
chr5	5348662	5348701	9493	0.075
chr5	54104780	54104819	9494	0.075
chr5	54492830	54492869	9495	0.075
chr5	55280451	55280490	9496	0.075
chr5	55787998	55788037	9497	0.075
chr5	56204906	56204945	9498	0.075
chr5	56801786	56801825	9499	0.075
chr5	57013142	57013181	9500	0.075

chr5	57787330	57787369	9501	0.075
chr5	58231152	58231191	9502	0.075
chr5	5846249	5846288	9503	0.075
chr5	60156017	60156056	9504	0.075
chr5	60825166	60825205	9505	0.075
chr5	62213214	62213253	9506	0.075
chr5	62976717	62976756	9507	0.075
chr5	63293178	63293217	9508	0.075
chr5	6360814	6360853	9509	0.075
chr5	64108067	64108106	9510	0.075
chr5	64579343	64579382	9511	0.075
chr5	65054663	65054702	9512	0.075
chr5	68081725	68081764	9513	0.075
chr5	71263781	71263820	9514	0.075
chr5	72770549	72770588	9515	0.075
chr5	73637663	73637702	9516	0.075
chr5	74959435	74959474	9517	0.075
chr5	75034664	75034703	9518	0.075
chr5	75982389	75982428	9519	0.075
chr5	76294844	76294883	9520	0.075
chr5	76408499	76408538	9521	0.075
chr5	77394467	77394506	9522	0.075
chr5	77970148	77970187	9523	0.075
chr5	78128937	78128976	9524	0.075
chr5	79167877	79167916	9525	0.075
chr5	79198879	79198918	9526	0.075
chr5	80084983	80085022	9527	0.075
chr5	82165811	82165850	9528	0.075
chr5	82234918	82234957	9529	0.075
chr5	82378967	82379006	9530	0.075
chr5	83968451	83968490	9531	0.075
chr5	85180009	85180048	9532	0.075
chr5	88009271	88009310	9533	0.075
chr5	88064894	88064933	9534	0.075
chr5	88252801	88252840	9535	0.075
chr5	89253179	89253218	9536	0.075
chr5	89721539	89721578	9537	0.075
chr5	92077736	92077775	9538	0.075
chr5	92397149	92397188	9539	0.075
chr5	92513810	92513849	9540	0.075
chr5	9261824	9261863	9541	0.075
chr5	92975665	92975704	9542	0.075
chr5	93178181	93178220	9543	0.075
chr5	93300092	93300131	9544	0.075
chr5	9335801	9335840	9545	0.075
chr5	93542409	93542448	9546	0.075
chr5	93591728	93591767	9547	0.075
chr5	95155211	95155250	9548	0.075
chr5	95241591	95241630	9549	0.075
chr5	95260854	95260893	9550	0.075

chr5	9533579	9533618	9551	0.075
chr5	95934392	95934431	9552	0.075
chr5	96296881	96296920	9553	0.075
chr5	9757419	9757458	9554	0.075
chr5	126919743	126919781	9555	0.076923077
chr6	149523909	149524024	9556	0.034482759
chr6	36271973	36272081	9557	0.036697248
chr6	120905252	120905336	9558	0.047058824
chr6	50668569	50668650	9559	0.048780488
chr6	152990834	152990912	9560	0.050632911
chr6	10041657	10041694	9561	0.052631579
chr6	100796592	100796629	9562	0.052631579
chr6	100886038	100886075	9563	0.052631579
chr6	101022033	101022070	9564	0.052631579
chr6	101022502	101022539	9565	0.052631579
chr6	101063172	101063209	9566	0.052631579
chr6	10126693	10126730	9567	0.052631579
chr6	102017009	102017046	9568	0.052631579
chr6	102438814	102438851	9569	0.052631579
chr6	1027124	1027161	9570	0.052631579
chr6	103343232	103343269	9571	0.052631579
chr6	103964955	103964992	9572	0.052631579
chr6	103965054	103965091	9573	0.052631579
chr6	10417242	10417279	9574	0.052631579
chr6	105620375	105620412	9575	0.052631579
chr6	105922631	105922668	9576	0.052631579
chr6	10631666	10631703	9577	0.052631579
chr6	106338310	106338347	9578	0.052631579
chr6	106349343	106349380	9579	0.052631579
chr6	106497234	106497271	9580	0.052631579
chr6	106508997	106509034	9581	0.052631579
chr6	106617079	106617116	9582	0.052631579
chr6	106672018	106672055	9583	0.052631579
chr6	107499424	107499461	9584	0.052631579
chr6	108010561	108010598	9585	0.052631579
chr6	108085289	108085326	9586	0.052631579
chr6	108273187	108273224	9587	0.052631579
chr6	108677551	108677588	9588	0.052631579
chr6	109108385	109108422	9589	0.052631579
chr6	109624697	109624734	9590	0.052631579
chr6	109810153	109810190	9591	0.052631579
chr6	109993599	109993636	9592	0.052631579
chr6	110620949	110620986	9593	0.052631579
chr6	11082258	11082295	9594	0.052631579
chr6	112099054	112099091	9595	0.052631579
chr6	112195831	112195868	9596	0.052631579
chr6	112455221	112455258	9597	0.052631579
chr6	112503959	112503996	9598	0.052631579
chr6	112743608	112743645	9599	0.052631579
chr6	113016392	113016429	9600	0.052631579

chr6	113303775	113303812	9601	0.052631579
chr6	113653927	113653964	9602	0.052631579
chr6	113708967	113709004	9603	0.052631579
chr6	114290285	114290322	9604	0.052631579
chr6	114591956	114591993	9605	0.052631579
chr6	115050818	115050855	9606	0.052631579
chr6	115762717	115762754	9607	0.052631579
chr6	115928598	115928635	9608	0.052631579
chr6	116091731	116091768	9609	0.052631579
chr6	116260860	116260897	9610	0.052631579
chr6	116484630	116484667	9611	0.052631579
chr6	116756070	116756107	9612	0.052631579
chr6	116878718	116878755	9613	0.052631579
chr6	117150524	117150561	9614	0.052631579
chr6	117712558	117712595	9615	0.052631579
chr6	11871562	11871599	9616	0.052631579
chr6	11873313	11873350	9617	0.052631579
chr6	11883926	11883963	9618	0.052631579
chr6	11912026	11912063	9619	0.052631579
chr6	119196638	119196675	9620	0.052631579
chr6	119264879	119264916	9621	0.052631579
chr6	119419629	119419666	9622	0.052631579
chr6	119878890	119878927	9623	0.052631579
chr6	120044657	120044694	9624	0.052631579
chr6	120050629	120050666	9625	0.052631579
chr6	121809789	121809826	9626	0.052631579
chr6	122620053	122620090	9627	0.052631579
chr6	122653034	122653071	9628	0.052631579
chr6	1226566	1226603	9629	0.052631579
chr6	122676128	122676165	9630	0.052631579
chr6	122809629	122809666	9631	0.052631579
chr6	123088708	123088745	9632	0.052631579
chr6	123182617	123182654	9633	0.052631579
chr6	123768395	123768432	9634	0.052631579
chr6	12438155	12438192	9635	0.052631579
chr6	124502831	124502868	9636	0.052631579
chr6	124626909	124626946	9637	0.052631579
chr6	12478493	12478530	9638	0.052631579
chr6	124961803	124961840	9639	0.052631579
chr6	124962632	124962669	9640	0.052631579
chr6	125020497	125020534	9641	0.052631579
chr6	125909456	125909493	9642	0.052631579
chr6	12644723	12644760	9643	0.052631579
chr6	12644803	12644840	9644	0.052631579
chr6	126718238	126718275	9645	0.052631579
chr6	12684750	12684787	9646	0.052631579
chr6	127023916	127023953	9647	0.052631579
chr6	127153696	127153733	9648	0.052631579
chr6	127171390	127171427	9649	0.052631579
chr6	127514979	127515016	9650	0.052631579

chr6	12765006	12765043	9651	0.052631579
chr6	127693268	127693305	9652	0.052631579
chr6	12770086	12770123	9653	0.052631579
chr6	127910916	127910953	9654	0.052631579
chr6	128266734	128266771	9655	0.052631579
chr6	129036409	129036446	9656	0.052631579
chr6	129203947	129203984	9657	0.052631579
chr6	129635318	129635355	9658	0.052631579
chr6	129822999	129823036	9659	0.052631579
chr6	12986748	12986785	9660	0.052631579
chr6	130210155	130210192	9661	0.052631579
chr6	13050333	13050370	9662	0.052631579
chr6	130650244	130650281	9663	0.052631579
chr6	130650469	130650506	9664	0.052631579
chr6	130656104	130656141	9665	0.052631579
chr6	130765149	130765186	9666	0.052631579
chr6	130988592	130988629	9667	0.052631579
chr6	131214922	131214959	9668	0.052631579
chr6	131831871	131831908	9669	0.052631579
chr6	132023475	132023512	9670	0.052631579
chr6	132040776	132040813	9671	0.052631579
chr6	132053972	132054009	9672	0.052631579
chr6	132312172	132312209	9673	0.052631579
chr6	133380258	133380295	9674	0.052631579
chr6	133617405	133617442	9675	0.052631579
chr6	134364725	134364762	9676	0.052631579
chr6	13473444	13473481	9677	0.052631579
chr6	134923577	134923614	9678	0.052631579
chr6	134952714	134952751	9679	0.052631579
chr6	136424012	136424049	9680	0.052631579
chr6	136561152	136561189	9681	0.052631579
chr6	13700940	13700977	9682	0.052631579
chr6	137604758	137604795	9683	0.052631579
chr6	137683021	137683058	9684	0.052631579
chr6	137729032	137729069	9685	0.052631579
chr6	137942479	137942516	9686	0.052631579
chr6	138104014	138104051	9687	0.052631579
chr6	138676333	138676370	9688	0.052631579
chr6	139158939	139158976	9689	0.052631579
chr6	139441176	139441213	9690	0.052631579
chr6	139738430	139738467	9691	0.052631579
chr6	140401231	140401268	9692	0.052631579
chr6	141316229	141316266	9693	0.052631579
chr6	142474660	142474697	9694	0.052631579
chr6	142475489	142475526	9695	0.052631579
chr6	143790937	143790974	9696	0.052631579
chr6	143953883	143953920	9697	0.052631579
chr6	144071111	144071148	9698	0.052631579
chr6	144071914	144071951	9699	0.052631579
chr6	144203997	144204034	9700	0.052631579

chr6	144465266	144465303	9701	0.052631579
chr6	144752973	144753010	9702	0.052631579
chr6	144881161	144881198	9703	0.052631579
chr6	145295211	145295248	9704	0.052631579
chr6	145478698	145478735	9705	0.052631579
chr6	146284257	146284294	9706	0.052631579
chr6	146323002	146323039	9707	0.052631579
chr6	146493222	146493259	9708	0.052631579
chr6	146493373	146493410	9709	0.052631579
chr6	146513872	146513909	9710	0.052631579
chr6	146547879	146547916	9711	0.052631579
chr6	147602036	147602073	9712	0.052631579
chr6	147872130	147872167	9713	0.052631579
chr6	148282798	148282835	9714	0.052631579
chr6	148650681	148650718	9715	0.052631579
chr6	148651459	148651496	9716	0.052631579
chr6	148971697	148971734	9717	0.052631579
chr6	148999713	148999750	9718	0.052631579
chr6	149244071	149244108	9719	0.052631579
chr6	14940544	14940581	9720	0.052631579
chr6	149543652	149543689	9721	0.052631579
chr6	149558083	149558120	9722	0.052631579
chr6	149681720	149681757	9723	0.052631579
chr6	149741577	149741614	9724	0.052631579
chr6	150181616	150181653	9725	0.052631579
chr6	150229119	150229156	9726	0.052631579
chr6	150855139	150855176	9727	0.052631579
chr6	151046199	151046236	9728	0.052631579
chr6	151192513	151192550	9729	0.052631579
chr6	151207271	151207308	9730	0.052631579
chr6	151510117	151510154	9731	0.052631579
chr6	151524512	151524549	9732	0.052631579
chr6	151711609	151711646	9733	0.052631579
chr6	152019291	152019328	9734	0.052631579
chr6	152308085	152308122	9735	0.052631579
chr6	152428133	152428170	9736	0.052631579
chr6	152477746	152477783	9737	0.052631579
chr6	153213761	153213798	9738	0.052631579
chr6	15379387	15379424	9739	0.052631579
chr6	153836986	153837023	9740	0.052631579
chr6	154010673	154010710	9741	0.052631579
chr6	155196204	155196241	9742	0.052631579
chr6	155366000	155366037	9743	0.052631579
chr6	155366771	155366808	9744	0.052631579
chr6	155398539	155398576	9745	0.052631579
chr6	155822106	155822143	9746	0.052631579
chr6	155822556	155822593	9747	0.052631579
chr6	155949158	155949195	9748	0.052631579
chr6	156763594	156763631	9749	0.052631579
chr6	157129282	157129319	9750	0.052631579

chr6	157130076	157130113	9751	0.052631579
chr6	157265349	157265386	9752	0.052631579
chr6	157509902	157509939	9753	0.052631579
chr6	157720598	157720635	9754	0.052631579
chr6	157753895	157753932	9755	0.052631579
chr6	157858707	157858744	9756	0.052631579
chr6	158068916	158068953	9757	0.052631579
chr6	158140304	158140341	9758	0.052631579
chr6	158655059	158655096	9759	0.052631579
chr6	158725152	158725189	9760	0.052631579
chr6	158785479	158785516	9761	0.052631579
chr6	158786167	158786204	9762	0.052631579
chr6	158820843	158820880	9763	0.052631579
chr6	159057166	159057203	9764	0.052631579
chr6	159115576	159115613	9765	0.052631579
chr6	159156002	159156039	9766	0.052631579
chr6	159818212	159818249	9767	0.052631579
chr6	159867156	159867193	9768	0.052631579
chr6	15988556	15988593	9769	0.052631579
chr6	160120230	160120267	9770	0.052631579
chr6	160762454	160762491	9771	0.052631579
chr6	160763246	160763283	9772	0.052631579
chr6	16197024	16197061	9773	0.052631579
chr6	162668267	162668304	9774	0.052631579
chr6	162938247	162938284	9775	0.052631579
chr6	163012576	163012613	9776	0.052631579
chr6	16398770	16398807	9777	0.052631579
chr6	164264395	164264432	9778	0.052631579
chr6	164626654	164626691	9779	0.052631579
chr6	16630060	16630097	9780	0.052631579
chr6	166304203	166304240	9781	0.052631579
chr6	166397797	166397834	9782	0.052631579
chr6	166398256	166398293	9783	0.052631579
chr6	16667082	16667119	9784	0.052631579
chr6	166719263	166719300	9785	0.052631579
chr6	166720033	166720070	9786	0.052631579
chr6	166918474	166918511	9787	0.052631579
chr6	166918934	166918971	9788	0.052631579
chr6	167015435	167015472	9789	0.052631579
chr6	167387100	167387137	9790	0.052631579
chr6	167413637	167413674	9791	0.052631579
chr6	167500363	167500400	9792	0.052631579
chr6	168521698	168521735	9793	0.052631579
chr6	168593034	168593071	9794	0.052631579
chr6	169677876	169677913	9795	0.052631579
chr6	169858109	169858146	9796	0.052631579
chr6	169858902	169858939	9797	0.052631579
chr6	17016565	17016602	9798	0.052631579
chr6	17029698	17029735	9799	0.052631579
chr6	170565595	170565632	9800	0.052631579

chr6	170572417	170572454	9801	0.052631579
chr6	1733198	1733235	9802	0.052631579
chr6	17765932	17765969	9803	0.052631579
chr6	17826762	17826799	9804	0.052631579
chr6	17916966	17917003	9805	0.052631579
chr6	18366211	18366248	9806	0.052631579
chr6	18494894	18494931	9807	0.052631579
chr6	18859022	18859059	9808	0.052631579
chr6	19267478	19267515	9809	0.052631579
chr6	19268403	19268440	9810	0.052631579
chr6	19660326	19660363	9811	0.052631579
chr6	20201530	20201567	9812	0.052631579
chr6	20949155	20949192	9813	0.052631579
chr6	2142420	2142457	9814	0.052631579
chr6	21873514	21873551	9815	0.052631579
chr6	21873704	21873741	9816	0.052631579
chr6	21980060	21980097	9817	0.052631579
chr6	2221039	2221076	9818	0.052631579
chr6	224305	224342	9819	0.052631579
chr6	22692344	22692381	9820	0.052631579
chr6	23506330	23506367	9821	0.052631579
chr6	24525530	24525567	9822	0.052631579
chr6	25037713	25037750	9823	0.052631579
chr6	25087927	25087964	9824	0.052631579
chr6	25532188	25532225	9825	0.052631579
chr6	25565133	25565170	9826	0.052631579
chr6	25761890	25761927	9827	0.052631579
chr6	26049926	26049963	9828	0.052631579
chr6	26125600	26125637	9829	0.052631579
chr6	26140144	26140181	9830	0.052631579
chr6	26274638	26274675	9831	0.052631579
chr6	26359861	26359898	9832	0.052631579
chr6	26473807	26473844	9833	0.052631579
chr6	26564452	26564489	9834	0.052631579
chr6	26565342	26565379	9835	0.052631579
chr6	26694275	26694312	9836	0.052631579
chr6	26706968	26707005	9837	0.052631579
chr6	27109760	27109797	9838	0.052631579
chr6	27139055	27139092	9839	0.052631579
chr6	27223098	27223135	9840	0.052631579
chr6	27773306	27773343	9841	0.052631579
chr6	27810646	27810683	9842	0.052631579
chr6	27845473	27845510	9843	0.052631579
chr6	27941423	27941460	9844	0.052631579
chr6	28229596	28229633	9845	0.052631579
chr6	28239385	28239422	9846	0.052631579
chr6	28439677	28439714	9847	0.052631579
chr6	28581511	28581548	9848	0.052631579
chr6	28840667	28840704	9849	0.052631579
chr6	28965411	28965448	9850	0.052631579

chr6	29053004	29053041	9851	0.052631579
chr6	29283669	29283706	9852	0.052631579
chr6	29284233	29284270	9853	0.052631579
chr6	29698668	29698705	9854	0.052631579
chr6	29894644	29894681	9855	0.052631579
chr6	29910868	29910905	9856	0.052631579
chr6	29911753	29911790	9857	0.052631579
chr6	30022759	30022796	9858	0.052631579
chr6	30145668	30145705	9859	0.052631579
chr6	30678416	30678453	9860	0.052631579
chr6	3100916	3100953	9861	0.052631579
chr6	3102874	3102911	9862	0.052631579
chr6	31365740	31365777	9863	0.052631579
chr6	31432121	31432158	9864	0.052631579
chr6	31492752	31492789	9865	0.052631579
chr6	31517748	31517785	9866	0.052631579
chr6	31816970	31817007	9867	0.052631579
chr6	3187839	3187876	9868	0.052631579
chr6	3188628	3188665	9869	0.052631579
chr6	32453251	32453288	9870	0.052631579
chr6	32522241	32522278	9871	0.052631579
chr6	32886401	32886438	9872	0.052631579
chr6	32927759	32927796	9873	0.052631579
chr6	33083515	33083552	9874	0.052631579
chr6	33685273	33685310	9875	0.052631579
chr6	33698600	33698637	9876	0.052631579
chr6	33766741	33766778	9877	0.052631579
chr6	33933720	33933757	9878	0.052631579
chr6	33980162	33980199	9879	0.052631579
chr6	33980754	33980791	9880	0.052631579
chr6	34348439	34348476	9881	0.052631579
chr6	3441494	3441531	9882	0.052631579
chr6	34692723	34692760	9883	0.052631579
chr6	34693331	34693368	9884	0.052631579
chr6	34736131	34736168	9885	0.052631579
chr6	3529196	3529233	9886	0.052631579
chr6	353000	353037	9887	0.052631579
chr6	35318819	35318856	9888	0.052631579
chr6	35544717	35544754	9889	0.052631579
chr6	35943201	35943238	9890	0.052631579
chr6	36185879	36185916	9891	0.052631579
chr6	36186662	36186699	9892	0.052631579
chr6	36373561	36373598	9893	0.052631579
chr6	37707151	37707188	9894	0.052631579
chr6	37832047	37832084	9895	0.052631579
chr6	38010744	38010781	9896	0.052631579
chr6	38657273	38657310	9897	0.052631579
chr6	38973796	38973833	9898	0.052631579
chr6	39136594	39136631	9899	0.052631579
chr6	39807526	39807563	9900	0.052631579

chr6	39982914	39982951	9901	0.052631579
chr6	4000868	4000905	9902	0.052631579
chr6	40151842	40151879	9903	0.052631579
chr6	40743570	40743607	9904	0.052631579
chr6	41118144	41118181	9905	0.052631579
chr6	41278846	41278883	9906	0.052631579
chr6	41515956	41515993	9907	0.052631579
chr6	41521088	41521163	9908	0.052631579
chr6	41628730	41628767	9909	0.052631579
chr6	41645016	41645053	9910	0.052631579
chr6	41940298	41940335	9911	0.052631579
chr6	43218099	43218136	9912	0.052631579
chr6	43274459	43274496	9913	0.052631579
chr6	43613082	43613119	9914	0.052631579
chr6	43705188	43705225	9915	0.052631579
chr6	44077857	44077894	9916	0.052631579
chr6	44224324	44224361	9917	0.052631579
chr6	44224607	44224644	9918	0.052631579
chr6	44238645	44238682	9919	0.052631579
chr6	45757403	45757440	9920	0.052631579
chr6	45788577	45788614	9921	0.052631579
chr6	46134901	46134938	9922	0.052631579
chr6	46148551	46148588	9923	0.052631579
chr6	46148944	46148981	9924	0.052631579
chr6	46155375	46155412	9925	0.052631579
chr6	46178700	46178737	9926	0.052631579
chr6	46512523	46512560	9927	0.052631579
chr6	46537882	46537919	9928	0.052631579
chr6	46593936	46593973	9929	0.052631579
chr6	46642422	46642459	9930	0.052631579
chr6	47087453	47087490	9931	0.052631579
chr6	47133336	47133373	9932	0.052631579
chr6	47869039	47869076	9933	0.052631579
chr6	48084656	48084693	9934	0.052631579
chr6	48148841	48148878	9935	0.052631579
chr6	48410547	48410584	9936	0.052631579
chr6	4928868	4928905	9937	0.052631579
chr6	49716066	49716103	9938	0.052631579
chr6	497860	497897	9939	0.052631579
chr6	50715670	50715707	9940	0.052631579
chr6	51072278	51072315	9941	0.052631579
chr6	51510235	51510272	9942	0.052631579
chr6	52227334	52227371	9943	0.052631579
chr6	52710266	52710303	9944	0.052631579
chr6	53010191	53010228	9945	0.052631579
chr6	53281869	53281906	9946	0.052631579
chr6	541006	541043	9947	0.052631579
chr6	54740459	54740496	9948	0.052631579
chr6	56022385	56022422	9949	0.052631579
chr6	56348557	56348594	9950	0.052631579

chr6	56824675	56824712	9951	0.052631579
chr6	57088790	57088827	9952	0.052631579
chr6	57236764	57236801	9953	0.052631579
chr6	57417874	57417911	9954	0.052631579
chr6	57626882	57626919	9955	0.052631579
chr6	5763368	5763405	9956	0.052631579
chr6	57948977	57949014	9957	0.052631579
chr6	62843954	62843991	9958	0.052631579
chr6	62896923	62896960	9959	0.052631579
chr6	63281328	63281365	9960	0.052631579
chr6	63521132	63521169	9961	0.052631579
chr6	64685289	64685326	9962	0.052631579
chr6	64688626	64688663	9963	0.052631579
chr6	65413911	65413948	9964	0.052631579
chr6	65780042	65780079	9965	0.052631579
chr6	65958939	65958976	9966	0.052631579
chr6	67293534	67293571	9967	0.052631579
chr6	67715457	67715494	9968	0.052631579
chr6	67942732	67942769	9969	0.052631579
chr6	68793803	68793840	9970	0.052631579
chr6	69188624	69188661	9971	0.052631579
chr6	69868135	69868172	9972	0.052631579
chr6	70852303	70852340	9973	0.052631579
chr6	71857358	71857395	9974	0.052631579
chr6	72075041	72075078	9975	0.052631579
chr6	72663027	72663064	9976	0.052631579
chr6	74015580	74015617	9977	0.052631579
chr6	74563063	74563100	9978	0.052631579
chr6	74563821	74563858	9979	0.052631579
chr6	7487264	7487301	9980	0.052631579
chr6	7612075	7612112	9981	0.052631579
chr6	76637093	76637130	9982	0.052631579
chr6	76800472	76800509	9983	0.052631579
chr6	78215963	78216000	9984	0.052631579
chr6	79793949	79793986	9985	0.052631579
chr6	79916187	79916224	9986	0.052631579
chr6	80336343	80336380	9987	0.052631579
chr6	80455408	80455445	9988	0.052631579
chr6	8082189	8082226	9989	0.052631579
chr6	81358284	81358321	9990	0.052631579
chr6	81426334	81426371	9991	0.052631579
chr6	82513557	82513594	9992	0.052631579
chr6	82832617	82832654	9993	0.052631579
chr6	82833227	82833264	9994	0.052631579
chr6	83319209	83319246	9995	0.052631579
chr6	8332694	8332731	9996	0.052631579
chr6	83906714	83906751	9997	0.052631579
chr6	84225466	84225503	9998	0.052631579
chr6	8439467	8439504	9999	0.052631579
chr6	84933841	84933878	10000	0.052631579

chr6	85229245	85229282	10001	0.052631579
chr6	85534014	85534051	10002	0.052631579
chr6	86232923	86232960	10003	0.052631579
chr6	86407743	86407780	10004	0.052631579
chr6	86502427	86502464	10005	0.052631579
chr6	86653626	86653663	10006	0.052631579
chr6	8714157	8714194	10007	0.052631579
chr6	8759237	8759274	10008	0.052631579
chr6	8759778	8759815	10009	0.052631579
chr6	87742439	87742476	10010	0.052631579
chr6	87743056	87743093	10011	0.052631579
chr6	87752041	87752078	10012	0.052631579
chr6	87794601	87794638	10013	0.052631579
chr6	88065727	88065764	10014	0.052631579
chr6	88153179	88153216	10015	0.052631579
chr6	88431079	88431116	10016	0.052631579
chr6	88912017	88912054	10017	0.052631579
chr6	89192090	89192127	10018	0.052631579
chr6	89399441	89399478	10019	0.052631579
chr6	90131449	90131486	10020	0.052631579
chr6	90139095	90139132	10021	0.052631579
chr6	9041090	9041127	10022	0.052631579
chr6	90660962	90660999	10023	0.052631579
chr6	91202376	91202413	10024	0.052631579
chr6	91629592	91629629	10025	0.052631579
chr6	92237134	92237171	10026	0.052631579
chr6	92261825	92261862	10027	0.052631579
chr6	92703962	92703999	10028	0.052631579
chr6	9366126	9366163	10029	0.052631579
chr6	9366530	9366567	10030	0.052631579
chr6	94414433	94414470	10031	0.052631579
chr6	94718120	94718157	10032	0.052631579
chr6	94856295	94856332	10033	0.052631579
chr6	9566315	9566352	10034	0.052631579
chr6	9566424	9566461	10035	0.052631579
chr6	9671935	9671972	10036	0.052631579
chr6	97166633	97166670	10037	0.052631579
chr6	97991019	97991056	10038	0.052631579
chr6	98318559	98318596	10039	0.052631579
chr6	98665028	98665065	10040	0.052631579
chr6	98807205	98807242	10041	0.052631579
chr6	99190034	99190071	10042	0.052631579
chr6	99252283	99252320	10043	0.052631579
chr6	9927919	9927956	10044	0.052631579
chr6	9928062	9928099	10045	0.052631579
chr6	99293560	99293597	10046	0.052631579
chr6	99389457	99389494	10047	0.052631579
chr6	99676398	99676435	10048	0.052631579
chr6	43350362	43350432	10049	0.056338028
chr6	64406377	64406434	10050	0.068965517

chr6	100123085	100123124	10051	0.075
chr6	100267877	100267916	10052	0.075
chr6	101695193	101695232	10053	0.075
chr6	102619468	102619507	10054	0.075
chr6	102937159	102937198	10055	0.075
chr6	10334739	10334778	10056	0.075
chr6	10404637	10404676	10057	0.075
chr6	10498425	10498464	10058	0.075
chr6	105849471	105849510	10059	0.075
chr6	106324892	106324931	10060	0.075
chr6	10685592	10685631	10061	0.075
chr6	107079843	107079882	10062	0.075
chr6	107768784	107768823	10063	0.075
chr6	107870500	107870539	10064	0.075
chr6	108133127	108133166	10065	0.075
chr6	109769768	109769807	10066	0.075
chr6	1098142 1098181	10067 0.075		
chr6	111992524	111992563	10068	0.075
chr6	113212663	113212702	10069	0.075
chr6	113467244	113467283	10070	0.075
chr6	113840331	113840370	10071	0.075
chr6	114161308	114161347	10072	0.075
chr6	114981796	114981835	10073	0.075
chr6	115752102	115752141	10074	0.075
chr6	116149931	116149970	10075	0.075
chr6	117734815	117734854	10076	0.075
chr6	120746354	120746393	10077	0.075
chr6	122051958	122051997	10078	0.075
chr6	122712205	122712244	10079	0.075
chr6	123143135	123143174	10080	0.075
chr6	12370420	12370459	10081	0.075
chr6	124563808	124563847	10082	0.075
chr6	124796181	124796220	10083	0.075
chr6	125043052	125043091	10084	0.075
chr6	125330971	125331010	10085	0.075
chr6	126014200	126014239	10086	0.075
chr6	126536243	126536282	10087	0.075
chr6	127481287	127481326	10088	0.075
chr6	129814413	129814452	10089	0.075
chr6	129920130	129920169	10090	0.075
chr6	132634870	132634909	10091	0.075
chr6	1337547 1337586	10092 0.075		
chr6	13416537	13416576	10093	0.075
chr6	13437514	13437553	10094	0.075
chr6	135052941	135052980	10095	0.075
chr6	135603933	135603972	10096	0.075
chr6	135630353	135630392	10097	0.075
chr6	135683536	135683575	10098	0.075
chr6	136543345	136543384	10099	0.075
chr6	137042442	137042481	10100	0.075

chr6	137330311	137330350	10101	0.075
chr6	137938647	137938686	10102	0.075
chr6	13796955	13796994	10103	0.075
chr6	138920767	138920806	10104	0.075
chr6	13898416	13898455	10105	0.075
chr6	139216343	139216382	10106	0.075
chr6	139305873	139305912	10107	0.075
chr6	139334331	139334370	10108	0.075
chr6	13935657	13935696	10109	0.075
chr6	142502447	142502486	10110	0.075
chr6	143038637	143038676	10111	0.075
chr6	143120059	143120098	10112	0.075
chr6	143866131	143866170	10113	0.075
chr6	14400019	14400058	10114	0.075
chr6	14454140	14454179	10115	0.075
chr6	146798655	146798694	10116	0.075
chr6	147268344	147268383	10117	0.075
chr6	148841824	148841863	10118	0.075
chr6	150213016	150213055	10119	0.075
chr6	151052901	151052940	10120	0.075
chr6	151384875	151384914	10121	0.075
chr6	151980164	151980203	10122	0.075
chr6	152298454	152298493	10123	0.075
chr6	152965893	152965932	10124	0.075
chr6	154118751	154118790	10125	0.075
chr6	154192604	154192643	10126	0.075
chr6	154845948	154845987	10127	0.075
chr6	156008339	156008378	10128	0.075
chr6	156051948	156051987	10129	0.075
chr6	156128829	156128868	10130	0.075
chr6	157495366	157495405	10131	0.075
chr6	158755753	158755792	10132	0.075
chr6	158841311	158841350	10133	0.075
chr6	159112284	159112323	10134	0.075
chr6	159906288	159906327	10135	0.075
chr6	160344020	160344059	10136	0.075
chr6	160783131	160783170	10137	0.075
chr6	16118679	16118718	10138	0.075
chr6	162057507	162057546	10139	0.075
chr6	162175409	162175448	10140	0.075
chr6	166797577	166797616	10141	0.075
chr6	167154966	167155005	10142	0.075
chr6	167240476	167240515	10143	0.075
chr6	168372258	168372297	10144	0.075
chr6	168653462	168653501	10145	0.075
chr6	168837800	168837839	10146	0.075
chr6	168927546	168927585	10147	0.075
chr6	168959767	168959806	10148	0.075
chr6	169357917	169357956	10149	0.075
chr6	169364234	169364273	10150	0.075

chr6	169587374	169587413	10151	0.075
chr6	17092213	17092252	10152	0.075
chr6	18097790	18097829	10153	0.075
chr6	18186677	18186716	10154	0.075
chr6	18309815	18309854	10155	0.075
chr6	18784929	18784968	10156	0.075
chr6	19947316	19947355	10157	0.075
chr6	19967553	19967592	10158	0.075
chr6	20000122	20000161	10159	0.075
chr6	20168449	20168488	10160	0.075
chr6	2037607	2037646	10161	0.075
chr6	20799666	20799705	10162	0.075
chr6	21371545	21371584	10163	0.075
chr6	21846707	21846746	10164	0.075
chr6	22793641	22793680	10165	0.075
chr6	25487496	25487535	10166	0.075
chr6	26292006	26292045	10167	0.075
chr6	27159742	27159781	10168	0.075
chr6	27330862	27330901	10169	0.075
chr6	27386097	27386136	10170	0.075
chr6	27477150	27477189	10171	0.075
chr6	27659933	27659972	10172	0.075
chr6	27813787	27813826	10173	0.075
chr6	27883358	27883397	10174	0.075
chr6	28152164	28152203	10175	0.075
chr6	28348426	28348465	10176	0.075
chr6	30870478	30870517	10177	0.075
chr6	30966810	30966849	10178	0.075
chr6	31222470	31222509	10179	0.075
chr6	31657433	31657472	10180	0.075
chr6	31703062	31703101	10181	0.075
chr6	31703807	31703846	10182	0.075
chr6	31745593	31745632	10183	0.075
chr6	33278733	33278772	10184	0.075
chr6	33493486	33493525	10185	0.075
chr6	33493671	33493710	10186	0.075
chr6	33906341	33906380	10187	0.075
chr6	35304659	35304698	10188	0.075
chr6	35335460	35335499	10189	0.075
chr6	35397357	35397396	10190	0.075
chr6	35500213	35500252	10191	0.075
chr6	36673271	36673310	10192	0.075
chr6	36744239	36744278	10193	0.075
chr6	37237126	37237165	10194	0.075
chr6	37609570	37609609	10195	0.075
chr6	3802263	3802302	10196	0.075
chr6	38459954	38459993	10197	0.075
chr6	38944075	38944114	10198	0.075
chr6	38950420	38950459	10199	0.075
chr6	39886055	39886094	10200	0.075

chr6	40268169	40268208	10201	0.075
chr6	40717151	40717190	10202	0.075
chr6	40718083	40718122	10203	0.075
chr6	40915305	40915344	10204	0.075
chr6	42253456	42253495	10205	0.075
chr6	42301417	42301456	10206	0.075
chr6	43849679	43849718	10207	0.075
chr6	438572	438611	10208	0.075
chr6	44457842	44457881	10209	0.075
chr6	44921984	44922023	10210	0.075
chr6	45857061	45857100	10211	0.075
chr6	47483087	47483126	10212	0.075
chr6	4932720	4932759	10213	0.075
chr6	51594572	51594611	10214	0.075
chr6	5288924	5288963	10215	0.075
chr6	53768058	53768097	10216	0.075
chr6	53784035	53784074	10217	0.075
chr6	56392010	56392049	10218	0.075
chr6	56898179	56898218	10219	0.075
chr6	5708404	5708443	10220	0.075
chr6	57434570	57434609	10221	0.075
chr6	6150777	6150816	10222	0.075
chr6	63393266	63393305	10223	0.075
chr6	6416398	6416437	10224	0.075
chr6	64383955	64383994	10225	0.075
chr6	71873056	71873095	10226	0.075
chr6	71936361	71936400	10227	0.075
chr6	75935660	75935699	10228	0.075
chr6	7616966	7617005	10229	0.075
chr6	7629748	7629787	10230	0.075
chr6	76625125	76625164	10231	0.075
chr6	7749385	7749424	10232	0.075
chr6	80440194	80440233	10233	0.075
chr6	81710410	81710449	10234	0.075
chr6	82254496	82254535	10235	0.075
chr6	83199269	83199308	10236	0.075
chr6	83508663	83508702	10237	0.075
chr6	8366010	8366049	10238	0.075
chr6	86469420	86469459	10239	0.075
chr6	87324848	87324887	10240	0.075
chr6	8736129	8736168	10241	0.075
chr6	9085735	9085774	10242	0.075
chr6	91355639	91355678	10243	0.075
chr6	91418690	91418729	10244	0.075
chr6	9264879	9264918	10245	0.075
chr6	93788022	93788061	10246	0.075
chr6	93986202	93986241	10247	0.075
chr6	9399321	9399360	10248	0.075
chr6	94652199	94652238	10249	0.075
chr6	94672958	94672997	10250	0.075

chr6	949206	949245	10251	0.075
chr6	95455969	95456008	10252	0.075
chr6	98937131	98937170	10253	0.075
chr6	98950335	98950374	10254	0.075
chr6	26393495	26393533	10255	0.076923077
chr6	27966332	27966370	10256	0.076923077
chr6	26265203	26265245	10257	0.093023256
chr6	24697002	24697042	10258	0.097560976
chr6	26342801	26342850	10259	0.1
chr7	6448001	6448111	10260	0.036036036
chr7	121002607	121002715	10261	0.036697248
chr7	44256241	44256348	10262	0.037037037
chr7	155067264	155067366	10263	0.038834951
chr7	133285486	133285578	10264	0.043010753
chr7	155652503	155652595	10265	0.043010753
chr7	111791718	111791804	10266	0.045977011
chr7	93899113	93899196	10267	0.047619048
chr7	77163471	77163551	10268	0.049382716
chr7	306551	306590	10269	0.05
chr7	100151529	100151566	10270	0.052631579
chr7	100152260	100152297	10271	0.052631579
chr7	100254110	100254147	10272	0.052631579
chr7	100370085	100370122	10273	0.052631579
chr7	100803714	100803751	10274	0.052631579
chr7	100872183	100872220	10275	0.052631579
chr7	101122000	101122037	10276	0.052631579
chr7	10121138	10121175	10277	0.052631579
chr7	101493342	101493379	10278	0.052631579
chr7	101711753	101711790	10279	0.052631579
chr7	101874161	101874198	10280	0.052631579
chr7	102462528	102462565	10281	0.052631579
chr7	103205419	103205456	10282	0.052631579
chr7	103212435	103212472	10283	0.052631579
chr7	103529669	103529706	10284	0.052631579
chr7	104027866	104027903	10285	0.052631579
chr7	104078258	104078295	10286	0.052631579
chr7	104184507	104184544	10287	0.052631579
chr7	104441332	104441369	10288	0.052631579
chr7	104545551	104545588	10289	0.052631579
chr7	105093805	105093842	10290	0.052631579
chr7	105202592	105202629	10291	0.052631579
chr7	105432281	105432318	10292	0.052631579
chr7	106036363	106036400	10293	0.052631579
chr7	106203175	106203212	10294	0.052631579
chr7	106307763	106307800	10295	0.052631579
chr7	106983281	106983318	10296	0.052631579
chr7	108398835	108398872	10297	0.052631579
chr7	108503314	108503351	10298	0.052631579
chr7	108503705	108503742	10299	0.052631579
chr7	10996476	10996513	10300	0.052631579

chr7	11003260	11003297	10301	0.052631579
chr7	110193119	110193156	10302	0.052631579
chr7	111736873	111736910	10303	0.052631579
chr7	111737689	111737726	10304	0.052631579
chr7	111800083	111800120	10305	0.052631579
chr7	112968326	112968363	10306	0.052631579
chr7	113253582	113253619	10307	0.052631579
chr7	113273572	113273609	10308	0.052631579
chr7	113451668	113451705	10309	0.052631579
chr7	113853828	113853865	10310	0.052631579
chr7	113958806	113958843	10311	0.052631579
chr7	114050678	114050715	10312	0.052631579
chr7	114148350	114148387	10313	0.052631579
chr7	114183678	114183715	10314	0.052631579
chr7	11430955	11430992	10315	0.052631579
chr7	114451004	114451041	10316	0.052631579
chr7	114540424	114540461	10317	0.052631579
chr7	114814592	114814629	10318	0.052631579
chr7	114963189	114963226	10319	0.052631579
chr7	115329892	115329929	10320	0.052631579
chr7	115689103	115689140	10321	0.052631579
chr7	116038917	116038954	10322	0.052631579
chr7	116404496	116404533	10323	0.052631579
chr7	116728634	116728671	10324	0.052631579
chr7	117028707	117028744	10325	0.052631579
chr7	117049647	117049684	10326	0.052631579
chr7	117211623	117211660	10327	0.052631579
chr7	117773711	117773748	10328	0.052631579
chr7	117773859	117773896	10329	0.052631579
chr7	117794667	117794704	10330	0.052631579
chr7	117807526	117807563	10331	0.052631579
chr7	11813131	11813168	10332	0.052631579
chr7	118404076	118404113	10333	0.052631579
chr7	119855000	119855037	10334	0.052631579
chr7	120267331	120267368	10335	0.052631579
chr7	120294530	120294567	10336	0.052631579
chr7	120357142	120357179	10337	0.052631579
chr7	120383399	120383436	10338	0.052631579
chr7	120442329	120442366	10339	0.052631579
chr7	121656483	121656520	10340	0.052631579
chr7	121857811	121857848	10341	0.052631579
chr7	122036035	122036072	10342	0.052631579
chr7	122121452	122121489	10343	0.052631579
chr7	122915047	122915084	10344	0.052631579
chr7	123119858	123119895	10345	0.052631579
chr7	123494872	123494909	10346	0.052631579
chr7	123814522	123814559	10347	0.052631579
chr7	125056665	125056702	10348	0.052631579
chr7	125169248	125169285	10349	0.052631579
chr7	125575621	125575658	10350	0.052631579

chr7	125862981	125863018	10351	0.052631579
chr7	126035475	126035512	10352	0.052631579
chr7	126459824	126459861	10353	0.052631579
chr7	126540574	126540611	10354	0.052631579
chr7	126690309	126690346	10355	0.052631579
chr7	127016399	127016436	10356	0.052631579
chr7	127455147	127455184	10357	0.052631579
chr7	12798636	12798673	10358	0.052631579
chr7	129064355	129064392	10359	0.052631579
chr7	129409282	129409319	10360	0.052631579
chr7	129582027	129582064	10361	0.052631579
chr7	129682824	129682861	10362	0.052631579
chr7	129721608	129721645	10363	0.052631579
chr7	129857941	129857978	10364	0.052631579
chr7	129867957	129867994	10365	0.052631579
chr7	130058669	130058706	10366	0.052631579
chr7	130064223	130064260	10367	0.052631579
chr7	130424671	130424708	10368	0.052631579
chr7	130433085	130433122	10369	0.052631579
chr7	130909819	130909856	10370	0.052631579
chr7	131033781	131033818	10371	0.052631579
chr7	131476046	131476083	10372	0.052631579
chr7	131503729	131503766	10373	0.052631579
chr7	131558586	131558623	10374	0.052631579
chr7	13157044	13157081	10375	0.052631579
chr7	131671437	131671474	10376	0.052631579
chr7	131683901	131683938	10377	0.052631579
chr7	131812486	131812523	10378	0.052631579
chr7	132531563	132531600	10379	0.052631579
chr7	132744726	132744763	10380	0.052631579
chr7	13290799	13290836	10381	0.052631579
chr7	133213964	133214001	10382	0.052631579
chr7	133960774	133960811	10383	0.052631579
chr7	134029508	134029545	10384	0.052631579
chr7	13420677	13420714	10385	0.052631579
chr7	134537607	134537644	10386	0.052631579
chr7	134845332	134845369	10387	0.052631579
chr7	136386303	136386340	10388	0.052631579
chr7	136410744	136410781	10389	0.052631579
chr7	137532498	137532535	10390	0.052631579
chr7	138201663	138201700	10391	0.052631579
chr7	138302283	138302320	10392	0.052631579
chr7	139196479	139196516	10393	0.052631579
chr7	139199319	139199356	10394	0.052631579
chr7	139906293	139906330	10395	0.052631579
chr7	140060542	140060579	10396	0.052631579
chr7	140579404	140579441	10397	0.052631579
chr7	140833680	140833717	10398	0.052631579
chr7	140876787	140876824	10399	0.052631579
chr7	141051312	141051349	10400	0.052631579

chr7	141242195	141242232	10401	0.052631579
chr7	141383723	141383760	10402	0.052631579
chr7	141675469	141675506	10403	0.052631579
chr7	142397948	142397985	10404	0.052631579
chr7	142712192	142712229	10405	0.052631579
chr7	142759367	142759404	10406	0.052631579
chr7	142760158	142760195	10407	0.052631579
chr7	143761672	143761709	10408	0.052631579
chr7	144107733	144107770	10409	0.052631579
chr7	144481397	144481434	10410	0.052631579
chr7	145059251	145059288	10411	0.052631579
chr7	145412256	145412293	10412	0.052631579
chr7	146529612	146529649	10413	0.052631579
chr7	146530332	146530369	10414	0.052631579
chr7	146731847	146731884	10415	0.052631579
chr7	146847934	146847971	10416	0.052631579
chr7	148164949	148164986	10417	0.052631579
chr7	148222900	148222937	10418	0.052631579
chr7	148370217	148370254	10419	0.052631579
chr7	148509174	148509211	10420	0.052631579
chr7	148523736	148523773	10421	0.052631579
chr7	149094482	149094519	10422	0.052631579
chr7	150547108	150547145	10423	0.052631579
chr7	150566463	150566500	10424	0.052631579
chr7	151163886	151163923	10425	0.052631579
chr7	151662056	151662093	10426	0.052631579
chr7	152086834	152086871	10427	0.052631579
chr7	152252588	152252625	10428	0.052631579
chr7	152403720	152403757	10429	0.052631579
chr7	152838396	152838433	10430	0.052631579
chr7	152879715	152879752	10431	0.052631579
chr7	153164087	153164124	10432	0.052631579
chr7	154012797	154012834	10433	0.052631579
chr7	154395988	154396025	10434	0.052631579
chr7	154414456	154414493	10435	0.052631579
chr7	154543132	154543169	10436	0.052631579
chr7	15513937	15513974	10437	0.052631579
chr7	155247806	155247843	10438	0.052631579
chr7	156125776	156125813	10439	0.052631579
chr7	15640215	15640252	10440	0.052631579
chr7	156683020	156683057	10441	0.052631579
chr7	157049212	157049249	10442	0.052631579
chr7	157208785	157208822	10443	0.052631579
chr7	157209560	157209597	10444	0.052631579
chr7	157689444	157689481	10445	0.052631579
chr7	157689925	157689962	10446	0.052631579
chr7	158119005	158119042	10447	0.052631579
chr7	158358724	158358761	10448	0.052631579
chr7	158360928	158360965	10449	0.052631579
chr7	16334423	16334460	10450	0.052631579

chr7	16404750	16404787	10451	0.052631579
chr7	17928939	17928976	10452	0.052631579
chr7	17946516	17946553	10453	0.052631579
chr7	18428440	18428477	10454	0.052631579
chr7	18634142	18634179	10455	0.052631579
chr7	18783869	18783906	10456	0.052631579
chr7	18788035	18788072	10457	0.052631579
chr7	18852843	18852880	10458	0.052631579
chr7	20347201	20347238	10459	0.052631579
chr7	20537789	20537826	10460	0.052631579
chr7	20895452	20895489	10461	0.052631579
chr7	21416531	21416568	10462	0.052631579
chr7	21495221	21495258	10463	0.052631579
chr7	21739592	21739629	10464	0.052631579
chr7	2181088	2181125	10465	0.052631579
chr7	22417054	22417091	10466	0.052631579
chr7	22619563	22619600	10467	0.052631579
chr7	24127470	24127507	10468	0.052631579
chr7	24304324	24304361	10469	0.052631579
chr7	24366175	24366212	10470	0.052631579
chr7	25104556	25104593	10471	0.052631579
chr7	2642337	2642374	10472	0.052631579
chr7	27126879	27126916	10473	0.052631579
chr7	27548895	27548932	10474	0.052631579
chr7	27578827	27578864	10475	0.052631579
chr7	27741180	27741217	10476	0.052631579
chr7	28304723	28304760	10477	0.052631579
chr7	28407112	28407149	10478	0.052631579
chr7	28436698	28436735	10479	0.052631579
chr7	28735609	28735646	10480	0.052631579
chr7	2917547	2917584	10481	0.052631579
chr7	29462750	29462787	10482	0.052631579
chr7	29486277	29486314	10483	0.052631579
chr7	30099196	30099233	10484	0.052631579
chr7	30887880	30887917	10485	0.052631579
chr7	30922350	30922387	10486	0.052631579
chr7	32645865	32645902	10487	0.052631579
chr7	32902459	32902496	10488	0.052631579
chr7	33001673	33001710	10489	0.052631579
chr7	33460180	33460217	10490	0.052631579
chr7	33465656	33465693	10491	0.052631579
chr7	33814594	33814631	10492	0.052631579
chr7	34042948	34042985	10493	0.052631579
chr7	35355814	35355851	10494	0.052631579
chr7	35596291	35596328	10495	0.052631579
chr7	36011411	36011448	10496	0.052631579
chr7	36992401	36992438	10497	0.052631579
chr7	37870379	37870416	10498	0.052631579
chr7	38011646	38011683	10499	0.052631579
chr7	3817679	3817716	10500	0.052631579

chr7	38440918	38440955	10501	0.052631579
chr7	38618759	38618796	10502	0.052631579
chr7	3873025	3873062	10503	0.052631579
chr7	39233508	39233545	10504	0.052631579
chr7	39277991	39278028	10505	0.052631579
chr7	39324922	39324959	10506	0.052631579
chr7	39614542	39614579	10507	0.052631579
chr7	40201699	40201736	10508	0.052631579
chr7	40201785	40201822	10509	0.052631579
chr7	40756855	40756892	10510	0.052631579
chr7	41135437	41135474	10511	0.052631579
chr7	41231137	41231174	10512	0.052631579
chr7	41431664	41431701	10513	0.052631579
chr7	41907526	41907563	10514	0.052631579
chr7	42139190	42139227	10515	0.052631579
chr7	42145448	42145485	10516	0.052631579
chr7	42159738	42159775	10517	0.052631579
chr7	42205794	42205831	10518	0.052631579
chr7	42947956	42947993	10519	0.052631579
chr7	43215888	43215925	10520	0.052631579
chr7	43577250	43577287	10521	0.052631579
chr7	43598452	43598489	10522	0.052631579
chr7	44137125	44137162	10523	0.052631579
chr7	44240764	44240801	10524	0.052631579
chr7	44249370	44249407	10525	0.052631579
chr7	44249538	44249575	10526	0.052631579
chr7	44566324	44566361	10527	0.052631579
chr7	44767937	44767974	10528	0.052631579
chr7	44973529	44973566	10529	0.052631579
chr7	45080478	45080515	10530	0.052631579
chr7	45107950	45107987	10531	0.052631579
chr7	45709839	45709876	10532	0.052631579
chr7	45871638	45871675	10533	0.052631579
chr7	45991503	45991540	10534	0.052631579
chr7	46419928	46419965	10535	0.052631579
chr7	46571167	46571204	10536	0.052631579
chr7	46637710	46637747	10537	0.052631579
chr7	46746348	46746385	10538	0.052631579
chr7	46757561	46757598	10539	0.052631579
chr7	46865968	46866005	10540	0.052631579
chr7	47006118	47006155	10541	0.052631579
chr7	47352558	47352595	10542	0.052631579
chr7	47397724	47397761	10543	0.052631579
chr7	47398528	47398565	10544	0.052631579
chr7	47465151	47465188	10545	0.052631579
chr7	47549242	47549279	10546	0.052631579
chr7	47689889	47689926	10547	0.052631579
chr7	48358917	48358954	10548	0.052631579
chr7	48847840	48847877	10549	0.052631579
chr7	49075261	49075298	10550	0.052631579

chr7	49321039	49321076	10551	0.052631579
chr7	4990752	4990789	10552	0.052631579
chr7	50127672	50127709	10553	0.052631579
chr7	50446760	50446797	10554	0.052631579
chr7	50675190	50675227	10555	0.052631579
chr7	50893267	50893304	10556	0.052631579
chr7	51014288	51014325	10557	0.052631579
chr7	51039944	51039981	10558	0.052631579
chr7	5118118	5118155	10559	0.052631579
chr7	51267000	51267037	10560	0.052631579
chr7	51748105	51748142	10561	0.052631579
chr7	53947799	53947836	10562	0.052631579
chr7	54220790	54220827	10563	0.052631579
chr7	54784496	54784533	10564	0.052631579
chr7	55070022	55070059	10565	0.052631579
chr7	55234875	55234912	10566	0.052631579
chr7	55581470	55581507	10567	0.052631579
chr7	556687	556724	10568	0.052631579
chr7	55731078	55731115	10569	0.052631579
chr7	5634645	5634682	10570	0.052631579
chr7	56599128	56599165	10571	0.052631579
chr7	57239123	57239160	10572	0.052631579
chr7	57419490	57419527	10573	0.052631579
chr7	57664401	57664438	10574	0.052631579
chr7	576949	576986	10575	0.052631579
chr7	6044776	6044813	10576	0.052631579
chr7	6097870	6097907	10577	0.052631579
chr7	6177080	6177117	10578	0.052631579
chr7	63011706	63011743	10579	0.052631579
chr7	63035680	63035717	10580	0.052631579
chr7	6471990	6472027	10581	0.052631579
chr7	66482212	66482249	10582	0.052631579
chr7	66550822	66550859	10583	0.052631579
chr7	66608009	66608046	10584	0.052631579
chr7	66725105	66725142	10585	0.052631579
chr7	67167933	67167970	10586	0.052631579
chr7	67370575	67370612	10587	0.052631579
chr7	68158002	68158039	10588	0.052631579
chr7	68172177	68172214	10589	0.052631579
chr7	68177216	68177253	10590	0.052631579
chr7	6836025	6836062	10591	0.052631579
chr7	68525589	68525626	10592	0.052631579
chr7	68961970	68962007	10593	0.052631579
chr7	68966560	68966597	10594	0.052631579
chr7	68967378	68967415	10595	0.052631579
chr7	69114573	69114610	10596	0.052631579
chr7	7053517	7053554	10597	0.052631579
chr7	70972507	70972544	10598	0.052631579
chr7	70978333	70978370	10599	0.052631579
chr7	71116537	71116574	10600	0.052631579

chr7	71384636	71384673	10601	0.052631579
chr7	71778521	71778558	10602	0.052631579
chr7	71841646	71841683	10603	0.052631579
chr7	72486864	72486901	10604	0.052631579
chr7	72634471	72634508	10605	0.052631579
chr7	72885874	72885911	10606	0.052631579
chr7	73657170	73657207	10607	0.052631579
chr7	7381689	7381726	10608	0.052631579
chr7	75030442	75030479	10609	0.052631579
chr7	75110522	75110559	10610	0.052631579
chr7	75311334	75311371	10611	0.052631579
chr7	7542087	7542124	10612	0.052631579
chr7	7542343	7542380	10613	0.052631579
chr7	75437419	75437456	10614	0.052631579
chr7	75541515	75541552	10615	0.052631579
chr7	75715355	75715392	10616	0.052631579
chr7	75796849	75796886	10617	0.052631579
chr7	77033142	77033179	10618	0.052631579
chr7	77407401	77407438	10619	0.052631579
chr7	77645290	77645327	10620	0.052631579
chr7	77761495	77761532	10621	0.052631579
chr7	77761912	77761949	10622	0.052631579
chr7	77935807	77935844	10623	0.052631579
chr7	78215291	78215328	10624	0.052631579
chr7	78686633	78686670	10625	0.052631579
chr7	80069462	80069499	10626	0.052631579
chr7	80141604	80141641	10627	0.052631579
chr7	80221856	80221893	10628	0.052631579
chr7	80694515	80694552	10629	0.052631579
chr7	80836940	80836977	10630	0.052631579
chr7	80936901	80936938	10631	0.052631579
chr7	81179860	81179897	10632	0.052631579
chr7	82396989	82397026	10633	0.052631579
chr7	82423003	82423040	10634	0.052631579
chr7	82489735	82489772	10635	0.052631579
chr7	83175347	83175384	10636	0.052631579
chr7	83175443	83175480	10637	0.052631579
chr7	8327386	8327423	10638	0.052631579
chr7	83935842	83935879	10639	0.052631579
chr7	83960515	83960552	10640	0.052631579
chr7	84413746	84413783	10641	0.052631579
chr7	84493663	84493700	10642	0.052631579
chr7	84550670	84550707	10643	0.052631579
chr7	84962531	84962568	10644	0.052631579
chr7	85161209	85161246	10645	0.052631579
chr7	85656127	85656164	10646	0.052631579
chr7	86202375	86202412	10647	0.052631579
chr7	86569980	86570017	10648	0.052631579
chr7	87638141	87638178	10649	0.052631579
chr7	87917100	87917137	10650	0.052631579

chr7	88031886	88031923	10651	0.052631579
chr7	8811876	8811913	10652	0.052631579
chr7	88631724	88631761	10653	0.052631579
chr7	89778407	89778444	10654	0.052631579
chr7	89779162	89779199	10655	0.052631579
chr7	89879927	89879964	10656	0.052631579
chr7	89880755	89880792	10657	0.052631579
chr7	90068583	90068620	10658	0.052631579
chr7	90119137	90119174	10659	0.052631579
chr7	90154413	90154450	10660	0.052631579
chr7	90215338	90215375	10661	0.052631579
chr7	90500087	90500124	10662	0.052631579
chr7	90501824	90501861	10663	0.052631579
chr7	90545110	90545147	10664	0.052631579
chr7	91525210	91525247	10665	0.052631579
chr7	91702656	91702693	10666	0.052631579
chr7	91759927	91759964	10667	0.052631579
chr7	930082	930119	10668	0.052631579
chr7	93357481	93357518	10669	0.052631579
chr7	93361067	93361104	10670	0.052631579
chr7	93481632	93481669	10671	0.052631579
chr7	93887647	93887684	10672	0.052631579
chr7	93890222	93890259	10673	0.052631579
chr7	94137989	94138026	10674	0.052631579
chr7	94892729	94892766	10675	0.052631579
chr7	94943029	94943066	10676	0.052631579
chr7	94993113	94993150	10677	0.052631579
chr7	95056995	95057032	10678	0.052631579
chr7	9524739	9524776	10679	0.052631579
chr7	95365194	95365231	10680	0.052631579
chr7	95476919	95476956	10681	0.052631579
chr7	95477803	95477840	10682	0.052631579
chr7	95533680	95533717	10683	0.052631579
chr7	96103611	96103648	10684	0.052631579
chr7	9621879	9621916	10685	0.052631579
chr7	96479386	96479423	10686	0.052631579
chr7	96886172	96886209	10687	0.052631579
chr7	9689518	9689555	10688	0.052631579
chr7	9689629	9689666	10689	0.052631579
chr7	97023702	97023739	10690	0.052631579
chr7	98354720	98354757	10691	0.052631579
chr7	98671719	98671756	10692	0.052631579
chr7	98820284	98820321	10693	0.052631579
chr7	99120164	99120201	10694	0.052631579
chr7	99865375	99865412	10695	0.052631579
chr7	99870789	99870826	10696	0.052631579
chr7	69868110	69868176	10697	0.059701493
chr7	100114091	100114130	10698	0.075
chr7	100325802	100325841	10699	0.075
chr7	100333625	100333664	10700	0.075

chr7	100462602	100462641	10701	0.075
chr7	101009026	101009065	10702	0.075
chr7	10183032	10183071	10703	0.075
chr7	102258138	102258177	10704	0.075
chr7	102559812	102559851	10705	0.075
chr7	105856212	105856251	10706	0.075
chr7	106295775	106295814	10707	0.075
chr7	107156987	107157026	10708	0.075
chr7	107427783	107427822	10709	0.075
chr7	108385604	108385643	10710	0.075
chr7	110374668	110374707	10711	0.075
chr7	110692155	110692194	10712	0.075
chr7	113842896	113842935	10713	0.075
chr7	115638347	115638386	10714	0.075
chr7	115684761	115684800	10715	0.075
chr7	115848837	115848876	10716	0.075
chr7	116263962	116264001	10717	0.075
chr7	116634348	116634387	10718	0.075
chr7	117612206	117612245	10719	0.075
chr7	1177227	1177266	10720	0.075
chr7	120067355	120067394	10721	0.075
chr7	120709708	120709747	10722	0.075
chr7	120757133	120757172	10723	0.075
chr7	121575040	121575079	10724	0.075
chr7	121749989	121750028	10725	0.075
chr7	123616704	123616743	10726	0.075
chr7	124227961	124228000	10727	0.075
chr7	126225045	126225084	10728	0.075
chr7	127221999	127222038	10729	0.075
chr7	127369498	127369537	10730	0.075
chr7	127468142	127468181	10731	0.075
chr7	127762954	127762993	10732	0.075
chr7	127836358	127836397	10733	0.075
chr7	127884628	127884667	10734	0.075
chr7	128283867	128283906	10735	0.075
chr7	128851959	128851998	10736	0.075
chr7	129429100	129429139	10737	0.075
chr7	129466600	129466639	10738	0.075
chr7	129792907	129792946	10739	0.075
chr7	1308278	1308317	10740	0.075
chr7	131025748	131025787	10741	0.075
chr7	131264863	131264902	10742	0.075
chr7	131656941	131656980	10743	0.075
chr7	132588393	132588432	10744	0.075
chr7	132969263	132969302	10745	0.075
chr7	134081133	134081172	10746	0.075
chr7	134170098	134170137	10747	0.075
chr7	134501888	134501927	10748	0.075
chr7	136766789	136766828	10749	0.075
chr7	139234638	139234677	10750	0.075

chr7	139622125	139622164	10751	0.075
chr7	140836420	140836459	10752	0.075
chr7	141664811	141664850	10753	0.075
chr7	142171136	142171175	10754	0.075
chr7	1460092	1460131	10755	0.075
chr7	146658669	146658708	10756	0.075
chr7	147572335	147572374	10757	0.075
chr7	148760056	148760095	10758	0.075
chr7	149070048	149070087	10759	0.075
chr7	149178356	149178395	10760	0.075
chr7	150297107	150297146	10761	0.075
chr7	150405926	150405965	10762	0.075
chr7	151040389	151040428	10763	0.075
chr7	151087510	151087549	10764	0.075
chr7	15234872	15234911	10765	0.075
chr7	154222337	154222376	10766	0.075
chr7	154569868	154569907	10767	0.075
chr7	154870072	154870111	10768	0.075
chr7	155087367	155087406	10769	0.075
chr7	155235071	155235110	10770	0.075
chr7	155258491	155258530	10771	0.075
chr7	155955886	155955925	10772	0.075
chr7	156168207	156168246	10773	0.075
chr7	157450950	157450989	10774	0.075
chr7	157749755	157749794	10775	0.075
chr7	157770290	157770329	10776	0.075
chr7	16188836	16188875	10777	0.075
chr7	17145920	17145959	10778	0.075
chr7	19122947	19122986	10779	0.075
chr7	20246686	20246725	10780	0.075
chr7	20800090	20800129	10781	0.075
chr7	21099258	21099297	10782	0.075
chr7	21275735	21275774	10783	0.075
chr7	2252138	2252177	10784	0.075
chr7	2286735	2286774	10785	0.075
chr7	2501961	2502000	10786	0.075
chr7	25324499	25324538	10787	0.075
chr7	25373016	25373055	10788	0.075
chr7	26220720	26220759	10789	0.075
chr7	26284113	26284152	10790	0.075
chr7	26464798	26464837	10791	0.075
chr7	26550818	26550857	10792	0.075
chr7	26606834	26606873	10793	0.075
chr7	2667847	2667886	10794	0.075
chr7	27888258	27888297	10795	0.075
chr7	30050552	30050591	10796	0.075
chr7	30514125	30514164	10797	0.075
chr7	32212751	32212790	10798	0.075
chr7	33156015	33156054	10799	0.075
chr7	33570708	33570747	10800	0.075

chr7	3567320	3567359	10801	0.075
chr7	35722030	35722069	10802	0.075
chr7	3651376	3651415	10803	0.075
chr7	3942938	3942977	10804	0.075
chr7	39958213	39958252	10805	0.075
chr7	40719952	40719991	10806	0.075
chr7	40990996	40991035	10807	0.075
chr7	41692303	41692342	10808	0.075
chr7	43170638	43170677	10809	0.075
chr7	43333835	43333874	10810	0.075
chr7	43884413	43884452	10811	0.075
chr7	44236751	44236790	10812	0.075
chr7	44976232	44976271	10813	0.075
chr7	4529714	4529753	10814	0.075
chr7	45893101	45893140	10815	0.075
chr7	4685974	4686013	10816	0.075
chr7	47087197	47087236	10817	0.075
chr7	48311091	48311130	10818	0.075
chr7	49086168	49086207	10819	0.075
chr7	49759119	49759158	10820	0.075
chr7	50481108	50481147	10821	0.075
chr7	50807306	50807345	10822	0.075
chr7	51647113	51647152	10823	0.075
chr7	51760244	51760283	10824	0.075
chr7	53090611	53090650	10825	0.075
chr7	53265711	53265750	10826	0.075
chr7	53537168	53537207	10827	0.075
chr7	54694319	54694358	10828	0.075
chr7	54838375	54838414	10829	0.075
chr7	55138793	55138832	10830	0.075
chr7	55220704	55220743	10831	0.075
chr7	5534232	5534271	10832	0.075
chr7	5535397	5535436	10833	0.075
chr7	55776110	55776149	10834	0.075
chr7	57268728	57268767	10835	0.075
chr7	57450728	57450767	10836	0.075
chr7	5757002	5757041	10837	0.075
chr7	6236481	6236520	10838	0.075
chr7	65082288	65082327	10839	0.075
chr7	6652821	6652860	10840	0.075
chr7	6755665	6755704	10841	0.075
chr7	67976495	67976534	10842	0.075
chr7	68386895	68386934	10843	0.075
chr7	70310253	70310292	10844	0.075
chr7	72751721	72751760	10845	0.075
chr7	76670576	76670615	10846	0.075
chr7	76948396	76948435	10847	0.075
chr7	77234485	77234524	10848	0.075
chr7	80254850	80254889	10849	0.075
chr7	80897845	80897884	10850	0.075

chr7	82629670	82629709	10851	0.075
chr7	83672944	83672983	10852	0.075
chr7	8408914	8408953	10853	0.075
chr7	84431185	84431224	10854	0.075
chr7	84638469	84638508	10855	0.075
chr7	85397597	85397636	10856	0.075
chr7	85622035	85622074	10857	0.075
chr7	86384018	86384057	10858	0.075
chr7	87646249	87646288	10859	0.075
chr7	90242353	90242392	10860	0.075
chr7	90762332	90762371	10861	0.075
chr7	90865179	90865218	10862	0.075
chr7	92302755	92302794	10863	0.075
chr7	93080822	93080861	10864	0.075
chr7	93830726	93830765	10865	0.075
chr7	93862268	93862307	10866	0.075
chr7	94825464	94825503	10867	0.075
chr7	96980950	96980989	10868	0.075
chr7	98252469	98252508	10869	0.075
chr7	98815667	98815706	10870	0.075
chr7	98941914	98941953	10871	0.075
chr7	99173509	99173548	10872	0.075
chr7	99694278	99694317	10873	0.075
chr7	63132956	63132996	10874	0.097560976
chr7	63986344	63986384	10875	0.097560976
chr8	49472052	49472147	10876	0.03125
chr8	119307731	119307846	10877	0.034482759
chr8	119157899	119158011	10878	0.03539823
chr8	140880960	140881059	10879	0.04
chr8	102239589	102239687	10880	0.04040404
chr8	37612648	37612731	10881	0.047619048
chr8	100178711	100178748	10882	0.052631579
chr8	100211105	100211142	10883	0.052631579
chr8	10054128	10054165	10884	0.052631579
chr8	100629876	100629913	10885	0.052631579
chr8	10072555	10072592	10886	0.052631579
chr8	100805305	100805342	10887	0.052631579
chr8	100811539	100811576	10888	0.052631579
chr8	10141461	10141498	10889	0.052631579
chr8	101629275	101629312	10890	0.052631579
chr8	101632468	101632505	10891	0.052631579
chr8	101632877	101632914	10892	0.052631579
chr8	101670278	101670315	10893	0.052631579
chr8	1019227	1019264	10894	0.052631579
chr8	102119104	102119141	10895	0.052631579
chr8	102747986	102748023	10896	0.052631579
chr8	102983702	102983739	10897	0.052631579
chr8	103068383	103068420	10898	0.052631579
chr8	103069153	103069190	10899	0.052631579
chr8	103348619	103348656	10900	0.052631579

chr8	103382708	103382745	10901	0.052631579
chr8	103543301	103543338	10902	0.052631579
chr8	103584676	103584713	10903	0.052631579
chr8	103976271	103976308	10904	0.052631579
chr8	104048250	104048287	10905	0.052631579
chr8	104163484	104163521	10906	0.052631579
chr8	10418746	10418783	10907	0.052631579
chr8	104519669	104519706	10908	0.052631579
chr8	104739530	104739567	10909	0.052631579
chr8	10527252	10527289	10910	0.052631579
chr8	10528932	10528969	10911	0.052631579
chr8	105464326	105464363	10912	0.052631579
chr8	105481622	105481659	10913	0.052631579
chr8	10583854	10583891	10914	0.052631579
chr8	106179873	106179910	10915	0.052631579
chr8	106207848	106207885	10916	0.052631579
chr8	106227309	106227346	10917	0.052631579
chr8	10625176	10625213	10918	0.052631579
chr8	106813760	106813797	10919	0.052631579
chr8	107017791	107017828	10920	0.052631579
chr8	107846436	107846473	10921	0.052631579
chr8	10793500	10793537	10922	0.052631579
chr8	10793602	10793639	10923	0.052631579
chr8	108011825	108011862	10924	0.052631579
chr8	108389116	108389153	10925	0.052631579
chr8	108532569	108532606	10926	0.052631579
chr8	108550403	108550440	10927	0.052631579
chr8	108634933	108634970	10928	0.052631579
chr8	108985915	108985952	10929	0.052631579
chr8	109181810	109181847	10930	0.052631579
chr8	109341450	109341487	10931	0.052631579
chr8	109666496	109666533	10932	0.052631579
chr8	109778740	109778777	10933	0.052631579
chr8	109927521	109927558	10934	0.052631579
chr8	11017326	11017363	10935	0.052631579
chr8	110620777	110620814	10936	0.052631579
chr8	11122839	11122876	10937	0.052631579
chr8	111519149	111519186	10938	0.052631579
chr8	111917387	111917424	10939	0.052631579
chr8	11217509	11217546	10940	0.052631579
chr8	11218253	11218290	10941	0.052631579
chr8	1124424	1124461	10942	0.052631579
chr8	1124776	1124813	10943	0.052631579
chr8	112809247	112809284	10944	0.052631579
chr8	112990193	112990230	10945	0.052631579
chr8	113314816	113314853	10946	0.052631579
chr8	114142609	114142646	10947	0.052631579
chr8	114429202	114429239	10948	0.052631579
chr8	11469544	11469581	10949	0.052631579
chr8	11472191	11472228	10950	0.052631579

chr8	115767484	115767521	10951	0.052631579
chr8	11597890	11597927	10952	0.052631579
chr8	116528047	116528084	10953	0.052631579
chr8	116617359	116617396	10954	0.052631579
chr8	117260553	117260590	10955	0.052631579
chr8	11739825	11739862	10956	0.052631579
chr8	117414973	117415010	10957	0.052631579
chr8	11745806	11745843	10958	0.052631579
chr8	117522332	117522369	10959	0.052631579
chr8	117845595	117845632	10960	0.052631579
chr8	118224404	118224441	10961	0.052631579
chr8	118256336	118256373	10962	0.052631579
chr8	118645045	118645082	10963	0.052631579
chr8	118833043	118833080	10964	0.052631579
chr8	118898160	118898197	10965	0.052631579
chr8	119105502	119105539	10966	0.052631579
chr8	119286750	119286787	10967	0.052631579
chr8	119841508	119841545	10968	0.052631579
chr8	119954269	119954306	10969	0.052631579
chr8	119954707	119954744	10970	0.052631579
chr8	120683933	120683970	10971	0.052631579
chr8	121527861	121527898	10972	0.052631579
chr8	121528036	121528073	10973	0.052631579
chr8	122038409	122038446	10974	0.052631579
chr8	122076720	122076757	10975	0.052631579
chr8	122086849	122086886	10976	0.052631579
chr8	122302573	122302610	10977	0.052631579
chr8	122563825	122563862	10978	0.052631579
chr8	122668520	122668557	10979	0.052631579
chr8	122697348	122697385	10980	0.052631579
chr8	122963805	122963842	10981	0.052631579
chr8	123396005	123396042	10982	0.052631579
chr8	123963400	123963437	10983	0.052631579
chr8	124722355	124722392	10984	0.052631579
chr8	125452605	125452642	10985	0.052631579
chr8	125453014	125453051	10986	0.052631579
chr8	125725619	125725656	10987	0.052631579
chr8	125795576	125795613	10988	0.052631579
chr8	126099533	126099570	10989	0.052631579
chr8	126242307	126242344	10990	0.052631579
chr8	126253762	126253799	10991	0.052631579
chr8	126674086	126674123	10992	0.052631579
chr8	127638305	127638342	10993	0.052631579
chr8	127775009	127775046	10994	0.052631579
chr8	128054022	128054059	10995	0.052631579
chr8	128748404	128748441	10996	0.052631579
chr8	128820327	128820364	10997	0.052631579
chr8	129019599	129019636	10998	0.052631579
chr8	129455603	129455640	10999	0.052631579
chr8	131598267	131598304	11000	0.052631579

chr8	1316806	1316843	11001	0.052631579
chr8	1323450	1323487	11002	0.052631579
chr8	13235060	13235097	11003	0.052631579
chr8	132897866	132897903	11004	0.052631579
chr8	133209056	133209093	11005	0.052631579
chr8	133533399	133533436	11006	0.052631579
chr8	134696800	134696837	11007	0.052631579
chr8	134826062	134826099	11008	0.052631579
chr8	135074536	135074573	11009	0.052631579
chr8	135340922	135340959	11010	0.052631579
chr8	135681669	135681706	11011	0.052631579
chr8	135701714	135701751	11012	0.052631579
chr8	135937670	135937707	11013	0.052631579
chr8	136323316	136323353	11014	0.052631579
chr8	136471909	136471946	11015	0.052631579
chr8	136505835	136505872	11016	0.052631579
chr8	137916818	137916855	11017	0.052631579
chr8	138495556	138495593	11018	0.052631579
chr8	138757990	138758027	11019	0.052631579
chr8	138941621	138941658	11020	0.052631579
chr8	139447514	139447551	11021	0.052631579
chr8	140554460	140554497	11022	0.052631579
chr8	140908806	140908843	11023	0.052631579
chr8	141054367	141054404	11024	0.052631579
chr8	1417535	1417572	11025	0.052631579
chr8	142299822	142299859	11026	0.052631579
chr8	143448202	143448239	11027	0.052631579
chr8	143542885	143542922	11028	0.052631579
chr8	143674714	143674751	11029	0.052631579
chr8	143675491	143675528	11030	0.052631579
chr8	143818125	143818162	11031	0.052631579
chr8	144326777	144326814	11032	0.052631579
chr8	144374679	144374716	11033	0.052631579
chr8	144501551	144501588	11034	0.052631579
chr8	145066144	145066181	11035	0.052631579
chr8	145069254	145069291	11036	0.052631579
chr8	145080830	145080867	11037	0.052631579
chr8	145511809	145511846	11038	0.052631579
chr8	145594123	145594160	11039	0.052631579
chr8	146078182	146078219	11040	0.052631579
chr8	14804188	14804225	11041	0.052631579
chr8	1571938	1571975	11042	0.052631579
chr8	16528875	16528912	11043	0.052631579
chr8	17563459	17563496	11044	0.052631579
chr8	18175546	18175583	11045	0.052631579
chr8	18385348	18385385	11046	0.052631579
chr8	18455823	18455860	11047	0.052631579
chr8	18468653	18468690	11048	0.052631579
chr8	18938328	18938365	11049	0.052631579
chr8	19143029	19143066	11050	0.052631579

chr8	19233360	19233397	11051	0.052631579
chr8	19319149	19319186	11052	0.052631579
chr8	19520817	19520854	11053	0.052631579
chr8	19551796	19551833	11054	0.052631579
chr8	19853586	19853623	11055	0.052631579
chr8	20232743	20232780	11056	0.052631579
chr8	21019742	21019779	11057	0.052631579
chr8	21200197	21200234	11058	0.052631579
chr8	21502503	21502540	11059	0.052631579
chr8	21542927	21542964	11060	0.052631579
chr8	21543779	21543816	11061	0.052631579
chr8	22045419	22045456	11062	0.052631579
chr8	22074482	22074519	11063	0.052631579
chr8	22075385	22075422	11064	0.052631579
chr8	22115493	22115530	11065	0.052631579
chr8	2221558	2221595	11066	0.052631579
chr8	22441968	22442005	11067	0.052631579
chr8	23604024	23604061	11068	0.052631579
chr8	23758042	23758079	11069	0.052631579
chr8	24641100	24641137	11070	0.052631579
chr8	25200505	25200542	11071	0.052631579
chr8	25229564	25229601	11072	0.052631579
chr8	25423762	25423799	11073	0.052631579
chr8	25662248	25662285	11074	0.052631579
chr8	26267858	26267895	11075	0.052631579
chr8	26495178	26495215	11076	0.052631579
chr8	28073867	28073904	11077	0.052631579
chr8	28113376	28113413	11078	0.052631579
chr8	29288785	29288822	11079	0.052631579
chr8	29437754	29437791	11080	0.052631579
chr8	29459806	29459843	11081	0.052631579
chr8	29683362	29683399	11082	0.052631579
chr8	29690733	29690770	11083	0.052631579
chr8	30590663	30590700	11084	0.052631579
chr8	3121914	3121951	11085	0.052631579
chr8	31277157	31277194	11086	0.052631579
chr8	31358300	31358337	11087	0.052631579
chr8	31572224	31572261	11088	0.052631579
chr8	31852951	31852988	11089	0.052631579
chr8	31906432	31906469	11090	0.052631579
chr8	3204062	3204099	11091	0.052631579
chr8	32205383	32205420	11092	0.052631579
chr8	32312652	32312689	11093	0.052631579
chr8	32530945	32530982	11094	0.052631579
chr8	3336097	3336134	11095	0.052631579
chr8	34018398	34018435	11096	0.052631579
chr8	3493433	3493470	11097	0.052631579
chr8	35441859	35441896	11098	0.052631579
chr8	35743358	35743395	11099	0.052631579
chr8	35868157	35868194	11100	0.052631579

chr8	36245389	36245426	11101	0.052631579
chr8	36753562	36753599	11102	0.052631579
chr8	36754350	36754387	11103	0.052631579
chr8	36839778	36839815	11104	0.052631579
chr8	37068520	37068557	11105	0.052631579
chr8	37146436	37146473	11106	0.052631579
chr8	37687986	37688023	11107	0.052631579
chr8	37814584	37814621	11108	0.052631579
chr8	37819199	37819236	11109	0.052631579
chr8	38294139	38294176	11110	0.052631579
chr8	3832169	3832206	11111	0.052631579
chr8	38391507	38391544	11112	0.052631579
chr8	38562732	38562769	11113	0.052631579
chr8	39164308	39164345	11114	0.052631579
chr8	39647966	39648003	11115	0.052631579
chr8	40193202	40193239	11116	0.052631579
chr8	40239164	40239201	11117	0.052631579
chr8	40523944	40523981	11118	0.052631579
chr8	40771663	40771700	11119	0.052631579
chr8	41239103	41239140	11120	0.052631579
chr8	413673	413710	11121	0.052631579
chr8	41857418	41857455	11122	0.052631579
chr8	41909124	41909161	11123	0.052631579
chr8	42687111	42687148	11124	0.052631579
chr8	4301307	4301344	11125	0.052631579
chr8	43058010	43058047	11126	0.052631579
chr8	4741156	4741193	11127	0.052631579
chr8	48151771	48151808	11128	0.052631579
chr8	4842525	4842562	11129	0.052631579
chr8	49390226	49390263	11130	0.052631579
chr8	49450802	49450839	11131	0.052631579
chr8	49662557	49662594	11132	0.052631579
chr8	50414493	50414530	11133	0.052631579
chr8	50546857	50546894	11134	0.052631579
chr8	50699618	50699655	11135	0.052631579
chr8	52192163	52192200	11136	0.052631579
chr8	54974154	54974191	11137	0.052631579
chr8	56441456	56441493	11138	0.052631579
chr8	56584709	56584746	11139	0.052631579
chr8	56628658	56628695	11140	0.052631579
chr8	5676092	5676129	11141	0.052631579
chr8	5684968	5685005	11142	0.052631579
chr8	5685253	5685290	11143	0.052631579
chr8	56907697	56907734	11144	0.052631579
chr8	57040840	57040877	11145	0.052631579
chr8	57156063	57156100	11146	0.052631579
chr8	57243333	57243370	11147	0.052631579
chr8	58062255	58062292	11148	0.052631579
chr8	58973682	58973719	11149	0.052631579
chr8	59633189	59633226	11150	0.052631579

chr8	59907780	59907817	11151	0.052631579
chr8	60415680	60415717	11152	0.052631579
chr8	60511646	60511683	11153	0.052631579
chr8	60705443	60705480	11154	0.052631579
chr8	60706183	60706220	11155	0.052631579
chr8	60768207	60768244	11156	0.052631579
chr8	61147139	61147176	11157	0.052631579
chr8	61437299	61437336	11158	0.052631579
chr8	61693712	61693749	11159	0.052631579
chr8	61704946	61704983	11160	0.052631579
chr8	61938010	61938047	11161	0.052631579
chr8	6257210	6257247	11162	0.052631579
chr8	62783404	62783441	11163	0.052631579
chr8	63008344	63008381	11164	0.052631579
chr8	63515096	63515133	11165	0.052631579
chr8	63537595	63537632	11166	0.052631579
chr8	63538148	63538185	11167	0.052631579
chr8	63788821	63788858	11168	0.052631579
chr8	6408186	6408223	11169	0.052631579
chr8	64114259	64114296	11170	0.052631579
chr8	64114938	64114975	11171	0.052631579
chr8	64680013	64680050	11172	0.052631579
chr8	65454316	65454353	11173	0.052631579
chr8	65524953	65524990	11174	0.052631579
chr8	65813392	65813429	11175	0.052631579
chr8	66177608	66177645	11176	0.052631579
chr8	66302080	66302117	11177	0.052631579
chr8	66549948	66549985	11178	0.052631579
chr8	66794251	66794288	11179	0.052631579
chr8	66799546	66799583	11180	0.052631579
chr8	66867474	66867511	11181	0.052631579
chr8	67252007	67252044	11182	0.052631579
chr8	67284035	67284072	11183	0.052631579
chr8	67284166	67284203	11184	0.052631579
chr8	67289520	67289557	11185	0.052631579
chr8	67292978	67293015	11186	0.052631579
chr8	67300849	67300886	11187	0.052631579
chr8	67504723	67504760	11188	0.052631579
chr8	67710329	67710366	11189	0.052631579
chr8	6773207	6773244	11190	0.052631579
chr8	68011028	68011065	11191	0.052631579
chr8	68057170	68057207	11192	0.052631579
chr8	6868874	6868911	11193	0.052631579
chr8	6939295	6939332	11194	0.052631579
chr8	69469476	69469513	11195	0.052631579
chr8	69639826	69639863	11196	0.052631579
chr8	70362237	70362274	11197	0.052631579
chr8	70392517	70392554	11198	0.052631579
chr8	70705258	70705295	11199	0.052631579
chr8	70705454	70705491	11200	0.052631579

chr8	70783361	70783398	11201	0.052631579
chr8	70908211	70908248	11202	0.052631579
chr8	71690417	71690454	11203	0.052631579
chr8	72253566	72253603	11204	0.052631579
chr8	72519644	72519681	11205	0.052631579
chr8	74190979	74191016	11206	0.052631579
chr8	74597062	74597099	11207	0.052631579
chr8	74910979	74911016	11208	0.052631579
chr8	77442656	77442693	11209	0.052631579
chr8	77880390	77880427	11210	0.052631579
chr8	77930150	77930187	11211	0.052631579
chr8	78701260	78701297	11212	0.052631579
chr8	78724723	78724760	11213	0.052631579
chr8	79065740	79065777	11214	0.052631579
chr8	79096960	79096997	11215	0.052631579
chr8	79296506	79296543	11216	0.052631579
chr8	79882112	79882149	11217	0.052631579
chr8	80166039	80166076	11218	0.052631579
chr8	80852140	80852177	11219	0.052631579
chr8	80878297	80878334	11220	0.052631579
chr8	80895859	80895896	11221	0.052631579
chr8	81210276	81210313	11222	0.052631579
chr8	8131385	8131422	11223	0.052631579
chr8	81486928	81486965	11224	0.052631579
chr8	82089541	82089578	11225	0.052631579
chr8	82110303	82110340	11226	0.052631579
chr8	82196494	82196531	11227	0.052631579
chr8	82731803	82731840	11228	0.052631579
chr8	82832422	82832459	11229	0.052631579
chr8	84414580	84414617	11230	0.052631579
chr8	84738394	84738431	11231	0.052631579
chr8	84740265	84740302	11232	0.052631579
chr8	84896080	84896117	11233	0.052631579
chr8	85231871	85231908	11234	0.052631579
chr8	85451635	85451672	11235	0.052631579
chr8	85683249	85683286	11236	0.052631579
chr8	86374872	86374909	11237	0.052631579
chr8	86670234	86670271	11238	0.052631579
chr8	87155188	87155225	11239	0.052631579
chr8	87235822	87235859	11240	0.052631579
chr8	87245253	87245290	11241	0.052631579
chr8	8751842	8751879	11242	0.052631579
chr8	8947465	8947502	11243	0.052631579
chr8	90374603	90374640	11244	0.052631579
chr8	9105152	9105189	11245	0.052631579
chr8	91802637	91802674	11246	0.052631579
chr8	92526715	92526752	11247	0.052631579
chr8	92725919	92725956	11248	0.052631579
chr8	92837618	92837655	11249	0.052631579
chr8	92899467	92899504	11250	0.052631579

chr8	92907770	92907807	11251	0.052631579
chr8	93400257	93400294	11252	0.052631579
chr8	93867016	93867053	11253	0.052631579
chr8	94727859	94727896	11254	0.052631579
chr8	9490456	9490493	11255	0.052631579
chr8	949675	949712	11256	0.052631579
chr8	94985702	94985739	11257	0.052631579
chr8	95753822	95753859	11258	0.052631579
chr8	95848533	95848570	11259	0.052631579
chr8	95962312	95962349	11260	0.052631579
chr8	96599208	96599245	11261	0.052631579
chr8	97804467	97804504	11262	0.052631579
chr8	98013041	98013078	11263	0.052631579
chr8	98114250	98114287	11264	0.052631579
chr8	981940	981977	11265	0.052631579
chr8	98555836	98555873	11266	0.052631579
chr8	98713413	98713450	11267	0.052631579
chr8	99126414	99126451	11268	0.052631579
chr8	99509772	99509809	11269	0.052631579
chr8	129914372	129914445	11270	0.054054054
chr8	4082979	4083045	11271	0.059701493
chr8	100944246	100944285	11272	0.075
chr8	10229653	10229692	11273	0.075
chr8	10323138	10323177	11274	0.075
chr8	10323607	10323646	11275	0.075
chr8	103396654	103396693	11276	0.075
chr8	104033058	104033097	11277	0.075
chr8	104824364	104824403	11278	0.075
chr8	107750122	107750161	11279	0.075
chr8	107852067	107852106	11280	0.075
chr8	108950156	108950195	11281	0.075
chr8	10943785	10943824	11282	0.075
chr8	109681331	109681370	11283	0.075
chr8	110200694	110200733	11284	0.075
chr8	112328632	112328671	11285	0.075
chr8	112959282	112959321	11286	0.075
chr8	11402617	11402656	11287	0.075
chr8	114672149	114672188	11288	0.075
chr8	11636000	11636039	11289	0.075
chr8	117067870	117067909	11290	0.075
chr8	117641064	117641103	11291	0.075
chr8	117712775	117712814	11292	0.075
chr8	118611962	118612001	11293	0.075
chr8	119670800	119670839	11294	0.075
chr8	120434782	120434821	11295	0.075
chr8	121492863	121492902	11296	0.075
chr8	121723617	121723656	11297	0.075
chr8	122576511	122576550	11298	0.075
chr8	123826310	123826349	11299	0.075
chr8	124708479	124708518	11300	0.075

chr8	127214504	127214543	11301	0.075
chr8	127408782	127408821	11302	0.075
chr8	128377036	128377075	11303	0.075
chr8	129028782	129028821	11304	0.075
chr8	130674509	130674548	11305	0.075
chr8	131110243	131110282	11306	0.075
chr8	131172748	131172787	11307	0.075
chr8	132206080	132206119	11308	0.075
chr8	132625797	132625836	11309	0.075
chr8	132972019	132972058	11310	0.075
chr8	133959038	133959077	11311	0.075
chr8	134201423	134201462	11312	0.075
chr8	134302217	134302256	11313	0.075
chr8	134608238	134608277	11314	0.075
chr8	135544195	135544234	11315	0.075
chr8	135809979	135810018	11316	0.075
chr8	138610412	138610451	11317	0.075
chr8	141611717	141611756	11318	0.075
chr8	142228042	142228081	11319	0.075
chr8	142500171	142500210	11320	0.075
chr8	142684315	142684354	11321	0.075
chr8	142886724	142886763	11322	0.075
chr8	143617019	143617058	11323	0.075
chr8	144583529	144583568	11324	0.075
chr8	144620917	144620956	11325	0.075
chr8	144680871	144680910	11326	0.075
chr8	144758588	144758627	11327	0.075
chr8	144970937	144970976	11328	0.075
chr8	145523344	145523383	11329	0.075
chr8	145663242	145663281	11330	0.075
chr8	15579742	15579781	11331	0.075
chr8	17446945	17446984	11332	0.075
chr8	18050343	18050382	11333	0.075
chr8	18823492	18823531	11334	0.075
chr8	21719286	21719325	11335	0.075
chr8	2205329	2205368	11336	0.075
chr8	22225081	22225120	11337	0.075
chr8	22334142	22334181	11338	0.075
chr8	22520470	22520509	11339	0.075
chr8	22569229	22569268	11340	0.075
chr8	23224975	23225014	11341	0.075
chr8	23351155	23351194	11342	0.075
chr8	23710548	23710587	11343	0.075
chr8	2525769	2525808	11344	0.075
chr8	25893037	25893076	11345	0.075
chr8	25951181	25951220	11346	0.075
chr8	28651041	28651080	11347	0.075
chr8	31418996	31419035	11348	0.075
chr8	35238127	35238166	11349	0.075
chr8	37302845	37302884	11350	0.075

chr8	37394968	37395007	11351	0.075
chr8	37449196	37449235	11352	0.075
chr8	37497631	37497670	11353	0.075
chr8	37546533	37546572	11354	0.075
chr8	37691256	37691295	11355	0.075
chr8	38153391	38153430	11356	0.075
chr8	38737639	38737678	11357	0.075
chr8	40841611	40841650	11358	0.075
chr8	41298428	41298467	11359	0.075
chr8	41337517	41337556	11360	0.075
chr8	4135537 4135576 11361 0.075			
chr8	41809258	41809297	11362	0.075
chr8	43033376	43033415	11363	0.075
chr8	4558015 4558054 11364 0.075			
chr8	47796619	47796658	11365	0.075
chr8	48434872	48434911	11366	0.075
chr8	48771565	48771604	11367	0.075
chr8	50139803	50139842	11368	0.075
chr8	51315440	51315479	11369	0.075
chr8	52928003	52928042	11370	0.075
chr8	53151789	53151828	11371	0.075
chr8	53789773	53789812	11372	0.075
chr8	54483881	54483920	11373	0.075
chr8	55048079	55048118	11374	0.075
chr8	56243704	56243743	11375	0.075
chr8	58854012	58854051	11376	0.075
chr8	59204500	59204539	11377	0.075
chr8	59278744	59278783	11378	0.075
chr8	59825351	59825390	11379	0.075
chr8	61127529	61127568	11380	0.075
chr8	61362905	61362944	11381	0.075
chr8	61868199	61868238	11382	0.075
chr8	61912778	61912817	11383	0.075
chr8	6353916 6353955 11384 0.075			
chr8	63753473	63753512	11385	0.075
chr8	64249317	64249356	11386	0.075
chr8	6451545 6451584 11387 0.075			
chr8	64899518	64899557	11388	0.075
chr8	65652219	65652258	11389	0.075
chr8	65992232	65992271	11390	0.075
chr8	66062832	66062871	11391	0.075
chr8	66264778	66264817	11392	0.075
chr8	66779381	66779420	11393	0.075
chr8	67352162	67352201	11394	0.075
chr8	67537009	67537048	11395	0.075
chr8	67974157	67974196	11396	0.075
chr8	6802453 6802492 11397 0.075			
chr8	6927340 6927379 11398 0.075			
chr8	69992839	69992878	11399	0.075
chr8	70408396	70408435	11400	0.075

chr8	70539319	70539358	11401	0.075
chr8	70756632	70756671	11402	0.075
chr8	71201688	71201727	11403	0.075
chr8	72250732	72250771	11404	0.075
chr8	72388014	72388053	11405	0.075
chr8	72999647	72999686	11406	0.075
chr8	73169316	73169355	11407	0.075
chr8	73348006	73348045	11408	0.075
chr8	74325332	74325371	11409	0.075
chr8	75246679	75246718	11410	0.075
chr8	76994922	76994961	11411	0.075
chr8	77676734	77676773	11412	0.075
chr8	77938955	77938994	11413	0.075
chr8	78073779	78073818	11414	0.075
chr8	79087322	79087361	11415	0.075
chr8	79244432	79244471	11416	0.075
chr8	80268771	80268810	11417	0.075
chr8	81259352	81259391	11418	0.075
chr8	81385034	81385073	11419	0.075
chr8	81979827	81979866	11420	0.075
chr8	86614672	86614711	11421	0.075
chr8	87639730	87639769	11422	0.075
chr8	9095499	9095538	11423	0.075
chr8	90971249	90971288	11424	0.075
chr8	91088338	91088377	11425	0.075
chr8	91726932	91726971	11426	0.075
chr8	92502198	92502237	11427	0.075
chr8	93795287	93795326	11428	0.075
chr8	93904147	93904186	11429	0.075
chr8	94220935	94220974	11430	0.075
chr8	94817834	94817873	11431	0.075
chr8	94958719	94958758	11432	0.075
chr8	95056801	95056840	11433	0.075
chr8	95800796	95800835	11434	0.075
chr8	96977905	96977944	11435	0.075
chr8	97106029	97106068	11436	0.075
chr8	97162753	97162792	11437	0.075
chr8	97532763	97532802	11438	0.075
chr8	99114476	99114515	11439	0.075
chr8	94108154	94108192	11440	0.076923077
chr8	97517832	97517870	11441	0.076923077
chr9	83497107	83497218	11442	0.035714286
chr9	138294186	138294287	11443	0.039215686
chr9	6907088	6907149	11444	0.048387097
chr9	14428083	14428163	11445	0.049382716
chr9	100116489	100116526	11446	0.052631579
chr9	100691781	100691818	11447	0.052631579
chr9	100755822	100755859	11448	0.052631579
chr9	101098575	101098612	11449	0.052631579
chr9	101169952	101169989	11450	0.052631579

chr9	101170625	101170662	11451	0.052631579
chr9	10123595	10123632	11452	0.052631579
chr9	101602469	101602506	11453	0.052631579
chr9	101954955	101954992	11454	0.052631579
chr9	102527022	102527059	11455	0.052631579
chr9	102584441	102584478	11456	0.052631579
chr9	102828649	102828686	11457	0.052631579
chr9	102971248	102971285	11458	0.052631579
chr9	103002674	103002711	11459	0.052631579
chr9	10311766	10311803	11460	0.052631579
chr9	103151353	103151390	11461	0.052631579
chr9	103219474	103219511	11462	0.052631579
chr9	103274598	103274635	11463	0.052631579
chr9	103308765	103308802	11464	0.052631579
chr9	103647387	103647424	11465	0.052631579
chr9	103678125	103678162	11466	0.052631579
chr9	104317243	104317280	11467	0.052631579
chr9	104317334	104317371	11468	0.052631579
chr9	104672792	104672829	11469	0.052631579
chr9	104988117	104988154	11470	0.052631579
chr9	105488815	105488852	11471	0.052631579
chr9	106089489	106089526	11472	0.052631579
chr9	106192363	106192400	11473	0.052631579
chr9	106192899	106192936	11474	0.052631579
chr9	106626439	106626476	11475	0.052631579
chr9	107050068	107050105	11476	0.052631579
chr9	1070530	1070567	11477	0.052631579
chr9	107156468	107156505	11478	0.052631579
chr9	107688163	107688200	11479	0.052631579
chr9	107733362	107733399	11480	0.052631579
chr9	107782205	107782242	11481	0.052631579
chr9	108132461	108132498	11482	0.052631579
chr9	108198192	108198229	11483	0.052631579
chr9	108390787	108390824	11484	0.052631579
chr9	109753037	109753074	11485	0.052631579
chr9	109810492	109810529	11486	0.052631579
chr9	10991278	10991315	11487	0.052631579
chr9	109942436	109942473	11488	0.052631579
chr9	109942839	109942876	11489	0.052631579
chr9	11061686	11061723	11490	0.052631579
chr9	110723672	110723709	11491	0.052631579
chr9	110752914	110752951	11492	0.052631579
chr9	111422655	111422692	11493	0.052631579
chr9	111423112	111423149	11494	0.052631579
chr9	112696279	112696316	11495	0.052631579
chr9	112743964	112744001	11496	0.052631579
chr9	11308746	11308783	11497	0.052631579
chr9	113463787	113463824	11498	0.052631579
chr9	113588185	113588222	11499	0.052631579
chr9	113634735	113634772	11500	0.052631579

chr9	113773572	113773609	11501	0.052631579
chr9	114428592	114428629	11502	0.052631579
chr9	114691801	114691838	11503	0.052631579
chr9	114941348	114941385	11504	0.052631579
chr9	115202413	115202450	11505	0.052631579
chr9	1161935 1161972 11506 0.052631579			
chr9	117547261	117547298	11507	0.052631579
chr9	118378686	118378723	11508	0.052631579
chr9	118709458	118709495	11509	0.052631579
chr9	118776381	118776418	11510	0.052631579
chr9	118866227	118866264	11511	0.052631579
chr9	119507110	119507147	11512	0.052631579
chr9	119910068	119910105	11513	0.052631579
chr9	120126583	120126620	11514	0.052631579
chr9	120151353	120151390	11515	0.052631579
chr9	120259557	120259594	11516	0.052631579
chr9	120740565	120740602	11517	0.052631579
chr9	120952333	120952370	11518	0.052631579
chr9	121283998	121284035	11519	0.052631579
chr9	121421353	121421390	11520	0.052631579
chr9	121573968	121574005	11521	0.052631579
chr9	122205277	122205314	11522	0.052631579
chr9	122262931	122262968	11523	0.052631579
chr9	122305940	122305977	11524	0.052631579
chr9	122663865	122663902	11525	0.052631579
chr9	122947578	122947615	11526	0.052631579
chr9	123166554	123166591	11527	0.052631579
chr9	123565951	123565988	11528	0.052631579
chr9	123570592	123570629	11529	0.052631579
chr9	123585688	123585725	11530	0.052631579
chr9	123617886	123617923	11531	0.052631579
chr9	124115018	124115055	11532	0.052631579
chr9	124115447	124115484	11533	0.052631579
chr9	124650104	124650141	11534	0.052631579
chr9	124938535	124938572	11535	0.052631579
chr9	125192123	125192160	11536	0.052631579
chr9	12562948	12562985	11537	0.052631579
chr9	125631703	125631740	11538	0.052631579
chr9	125731102	125731139	11539	0.052631579
chr9	125731319	125731356	11540	0.052631579
chr9	126180530	126180567	11541	0.052631579
chr9	126328897	126328934	11542	0.052631579
chr9	126581791	126581828	11543	0.052631579
chr9	126666089	126666126	11544	0.052631579
chr9	127041357	127041394	11545	0.052631579
chr9	12726205	12726242	11546	0.052631579
chr9	127296365	127296402	11547	0.052631579
chr9	127686156	127686193	11548	0.052631579
chr9	127789436	127789473	11549	0.052631579
chr9	127859714	127859751	11550	0.052631579

chr9	128440906	128440943	11551	0.052631579
chr9	128545964	128546001	11552	0.052631579
chr9	128546723	128546760	11553	0.052631579
chr9	128662620	128662657	11554	0.052631579
chr9	128910395	128910432	11555	0.052631579
chr9	128978998	128979035	11556	0.052631579
chr9	129185053	129185090	11557	0.052631579
chr9	129246848	129246885	11558	0.052631579
chr9	129455819	129455856	11559	0.052631579
chr9	129710796	129710833	11560	0.052631579
chr9	130046833	130046870	11561	0.052631579
chr9	130138308	130138345	11562	0.052631579
chr9	130377912	130377949	11563	0.052631579
chr9	130417895	130417932	11564	0.052631579
chr9	130434922	130434959	11565	0.052631579
chr9	130441093	130441130	11566	0.052631579
chr9	130710770	130710807	11567	0.052631579
chr9	130869900	130869937	11568	0.052631579
chr9	130900171	130900208	11569	0.052631579
chr9	130949679	130949716	11570	0.052631579
chr9	130950894	130950931	11571	0.052631579
chr9	131136451	131136488	11572	0.052631579
chr9	13115142	13115179	11573	0.052631579
chr9	13116555	13116592	11574	0.052631579
chr9	131262638	131262675	11575	0.052631579
chr9	131427032	131427069	11576	0.052631579
chr9	131671461	131671498	11577	0.052631579
chr9	131677823	131677860	11578	0.052631579
chr9	131805089	131805126	11579	0.052631579
chr9	132546556	132546593	11580	0.052631579
chr9	132547959	132547996	11581	0.052631579
chr9	132603530	132603567	11582	0.052631579
chr9	132725905	132725942	11583	0.052631579
chr9	132769193	132769230	11584	0.052631579
chr9	133162455	133162492	11585	0.052631579
chr9	133577416	133577453	11586	0.052631579
chr9	133637570	133637607	11587	0.052631579
chr9	13405470	13405507	11588	0.052631579
chr9	134201504	134201541	11589	0.052631579
chr9	134616651	134616688	11590	0.052631579
chr9	13491737	13491774	11591	0.052631579
chr9	135362197	135362234	11592	0.052631579
chr9	135457011	135457048	11593	0.052631579
chr9	135489285	135489322	11594	0.052631579
chr9	135685195	135685232	11595	0.052631579
chr9	13632619	13632656	11596	0.052631579
chr9	13633279	13633316	11597	0.052631579
chr9	136729107	136729144	11598	0.052631579
chr9	136739859	136739896	11599	0.052631579
chr9	136827156	136827193	11600	0.052631579

chr9	136856472	136856509	11601	0.052631579
chr9	137787368	137787405	11602	0.052631579
chr9	137788432	137788469	11603	0.052631579
chr9	138697658	138697695	11604	0.052631579
chr9	138864326	138864363	11605	0.052631579
chr9	138877673	138877710	11606	0.052631579
chr9	138919517	138919554	11607	0.052631579
chr9	138956706	138956743	11608	0.052631579
chr9	139009968	139010005	11609	0.052631579
chr9	139194758	139194795	11610	0.052631579
chr9	139234588	139234625	11611	0.052631579
chr9	139593401	139593438	11612	0.052631579
chr9	14033071	14033108	11613	0.052631579
chr9	14317391	14317428	11614	0.052631579
chr9	14320671	14320708	11615	0.052631579
chr9	14321920	14321957	11616	0.052631579
chr9	14457231	14457268	11617	0.052631579
chr9	15463309	15463346	11618	0.052631579
chr9	16302987	16303024	11619	0.052631579
chr9	16415522	16415559	11620	0.052631579
chr9	16427223	16427260	11621	0.052631579
chr9	16782904	16782941	11622	0.052631579
chr9	17592058	17592095	11623	0.052631579
chr9	17597821	17597858	11624	0.052631579
chr9	17976920	17976957	11625	0.052631579
chr9	17986457	17986494	11626	0.052631579
chr9	18335029	18335066	11627	0.052631579
chr9	18647261	18647298	11628	0.052631579
chr9	18819975	18820012	11629	0.052631579
chr9	18905634	18905671	11630	0.052631579
chr9	19132746	19132783	11631	0.052631579
chr9	19208667	19208704	11632	0.052631579
chr9	19557632	19557669	11633	0.052631579
chr9	19936476	19936513	11634	0.052631579
chr9	2007530 2007567	11635	0.052631579	
chr9	21617069	21617106	11636	0.052631579
chr9	22292328	22292365	11637	0.052631579
chr9	2232320 2232357	11638	0.052631579	
chr9	22347898	22347935	11639	0.052631579
chr9	22908284	22908321	11640	0.052631579
chr9	22995790	22995827	11641	0.052631579
chr9	22996205	22996242	11642	0.052631579
chr9	2310563 2310600	11643	0.052631579	
chr9	23741175	23741212	11644	0.052631579
chr9	24969316	24969353	11645	0.052631579
chr9	26466422	26466459	11646	0.052631579
chr9	26504805	26504842	11647	0.052631579
chr9	26937198	26937235	11648	0.052631579
chr9	27116720	27116757	11649	0.052631579
chr9	27295418	27295455	11650	0.052631579

chr9	27390261	27390298	11651	0.052631579
chr9	2761581	2761618	11652	0.052631579
chr9	27866923	27866960	11653	0.052631579
chr9	28021932	28021969	11654	0.052631579
chr9	28092611	28092648	11655	0.052631579
chr9	28420305	28420342	11656	0.052631579
chr9	28448000	28448037	11657	0.052631579
chr9	28763382	28763419	11658	0.052631579
chr9	29001429	29001466	11659	0.052631579
chr9	29024612	29024649	11660	0.052631579
chr9	29025128	29025165	11661	0.052631579
chr9	29580572	29580609	11662	0.052631579
chr9	31179175	31179212	11663	0.052631579
chr9	31421516	31421553	11664	0.052631579
chr9	31705180	31705217	11665	0.052631579
chr9	32273949	32273986	11666	0.052631579
chr9	33636812	33636849	11667	0.052631579
chr9	33649098	33649135	11668	0.052631579
chr9	33805757	33805794	11669	0.052631579
chr9	34639044	34639081	11670	0.052631579
chr9	35337312	35337349	11671	0.052631579
chr9	35747393	35747430	11672	0.052631579
chr9	35776253	35776290	11673	0.052631579
chr9	35804833	35804870	11674	0.052631579
chr9	35970205	35970242	11675	0.052631579
chr9	36081235	36081272	11676	0.052631579
chr9	36181118	36181155	11677	0.052631579
chr9	36698643	36698680	11678	0.052631579
chr9	36977763	36977800	11679	0.052631579
chr9	36993069	36993106	11680	0.052631579
chr9	37148527	37148564	11681	0.052631579
chr9	37149270	37149307	11682	0.052631579
chr9	37447795	37447832	11683	0.052631579
chr9	3748481	3748518	11684	0.052631579
chr9	37501158	37501195	11685	0.052631579
chr9	37735661	37735698	11686	0.052631579
chr9	38328586	38328623	11687	0.052631579
chr9	38477664	38477701	11688	0.052631579
chr9	38729849	38729886	11689	0.052631579
chr9	4090622	4090659	11690	0.052631579
chr9	42898871	42898908	11691	0.052631579
chr9	5003798	5003835	11692	0.052631579
chr9	5372077	5372114	11693	0.052631579
chr9	5776040	5776077	11694	0.052631579
chr9	6078647	6078684	11695	0.052631579
chr9	6109134	6109171	11696	0.052631579
chr9	67907501	67907538	11697	0.052631579
chr9	67926858	67926895	11698	0.052631579
chr9	70927115	70927152	11699	0.052631579
chr9	70962560	70962597	11700	0.052631579

chr9	71072966	71073003	11701	0.052631579
chr9	71190607	71190644	11702	0.052631579
chr9	71205284	71205321	11703	0.052631579
chr9	71278945	71278982	11704	0.052631579
chr9	71564621	71564658	11705	0.052631579
chr9	71865420	71865457	11706	0.052631579
chr9	72161675	72161712	11707	0.052631579
chr9	72302756	72302793	11708	0.052631579
chr9	72352271	72352308	11709	0.052631579
chr9	72473642	72473679	11710	0.052631579
chr9	72474409	72474446	11711	0.052631579
chr9	72697408	72697445	11712	0.052631579
chr9	72805740	72805777	11713	0.052631579
chr9	73307626	73307663	11714	0.052631579
chr9	73755516	73755553	11715	0.052631579
chr9	7394552	7394589	11716	0.052631579
chr9	74017473	74017510	11717	0.052631579
chr9	74159113	74159150	11718	0.052631579
chr9	74229158	74229195	11719	0.052631579
chr9	74566422	74566459	11720	0.052631579
chr9	74581805	74581842	11721	0.052631579
chr9	74943852	74943889	11722	0.052631579
chr9	75670988	75671025	11723	0.052631579
chr9	75725157	75725194	11724	0.052631579
chr9	75865309	75865346	11725	0.052631579
chr9	76451704	76451741	11726	0.052631579
chr9	76574966	76575003	11727	0.052631579
chr9	76622292	76622329	11728	0.052631579
chr9	7677632	7677669	11729	0.052631579
chr9	76820938	76820975	11730	0.052631579
chr9	76928542	76928579	11731	0.052631579
chr9	76957046	76957083	11732	0.052631579
chr9	77163450	77163487	11733	0.052631579
chr9	7766051	7766088	11734	0.052631579
chr9	77697199	77697236	11735	0.052631579
chr9	77697855	77697892	11736	0.052631579
chr9	792251	792288	11737	0.052631579
chr9	80549026	80549063	11738	0.052631579
chr9	81092432	81092469	11739	0.052631579
chr9	81194797	81194834	11740	0.052631579
chr9	81310368	81310405	11741	0.052631579
chr9	81375874	81375911	11742	0.052631579
chr9	81439061	81439098	11743	0.052631579
chr9	8146192	8146229	11744	0.052631579
chr9	81576946	81576983	11745	0.052631579
chr9	81589179	81589216	11746	0.052631579
chr9	82084334	82084371	11747	0.052631579
chr9	82084547	82084584	11748	0.052631579
chr9	82419661	82419698	11749	0.052631579
chr9	83777865	83777902	11750	0.052631579

chr9	84109178	84109215	11751	0.052631579
chr9	84256636	84256673	11752	0.052631579
chr9	84293798	84293835	11753	0.052631579
chr9	84535529	84535566	11754	0.052631579
chr9	84791417	84791454	11755	0.052631579
chr9	84839332	84839369	11756	0.052631579
chr9	84944976	84945013	11757	0.052631579
chr9	84945218	84945255	11758	0.052631579
chr9	85707792	85707829	11759	0.052631579
chr9	85857808	85857845	11760	0.052631579
chr9	86266232	86266269	11761	0.052631579
chr9	86396853	86396890	11762	0.052631579
chr9	86654557	86654594	11763	0.052631579
chr9	86672304	86672341	11764	0.052631579
chr9	86721633	86721670	11765	0.052631579
chr9	86831225	86831262	11766	0.052631579
chr9	8687192	8687229	11767	0.052631579
chr9	87343755	87343792	11768	0.052631579
chr9	8807892	8807929	11769	0.052631579
chr9	88082993	88083030	11770	0.052631579
chr9	88708252	88708289	11771	0.052631579
chr9	90872412	90872449	11772	0.052631579
chr9	91403053	91403090	11773	0.052631579
chr9	91490779	91490816	11774	0.052631579
chr9	92750655	92750692	11775	0.052631579
chr9	9277713	9277750	11776	0.052631579
chr9	92965006	92965043	11777	0.052631579
chr9	92992653	92992690	11778	0.052631579
chr9	93139901	93139938	11779	0.052631579
chr9	93292122	93292159	11780	0.052631579
chr9	93465045	93465082	11781	0.052631579
chr9	93810725	93810762	11782	0.052631579
chr9	94889184	94889221	11783	0.052631579
chr9	95040438	95040475	11784	0.052631579
chr9	95095731	95095768	11785	0.052631579
chr9	95096497	95096534	11786	0.052631579
chr9	95254235	95254272	11787	0.052631579
chr9	95387856	95387893	11788	0.052631579
chr9	95388660	95388697	11789	0.052631579
chr9	9553481	9553518	11790	0.052631579
chr9	96670426	96670463	11791	0.052631579
chr9	96767387	96767424	11792	0.052631579
chr9	96794594	96794631	11793	0.052631579
chr9	96885884	96885921	11794	0.052631579
chr9	97577184	97577221	11795	0.052631579
chr9	97638766	97638803	11796	0.052631579
chr9	98103187	98103224	11797	0.052631579
chr9	98194857	98194894	11798	0.052631579
chr9	98415673	98415710	11799	0.052631579
chr9	98422039	98422076	11800	0.052631579

chr9	98523492	98523529	11801	0.052631579
chr9	98978533	98978570	11802	0.052631579
chr9	99422005	99422042	11803	0.052631579
chr9	9953535	9953572	11804	0.052631579
chr9	99820476	99820513	11805	0.052631579
chr9	8170510	8170584	11806	0.053333333
chr9	89512248	89512308	11807	0.06557377
chr9	100044626	100044665	11808	0.075
chr9	100203362	100203401	11809	0.075
chr9	100841999	100842038	11810	0.075
chr9	101119562	101119601	11811	0.075
chr9	101402115	101402154	11812	0.075
chr9	104980678	104980717	11813	0.075
chr9	105081690	105081729	11814	0.075
chr9	105187959	105187998	11815	0.075
chr9	106604273	106604312	11816	0.075
chr9	108622651	108622690	11817	0.075
chr9	108730367	108730406	11818	0.075
chr9	109062320	109062359	11819	0.075
chr9	109364946	109364985	11820	0.075
chr9	109546845	109546884	11821	0.075
chr9	109636711	109636750	11822	0.075
chr9	111411412	111411451	11823	0.075
chr9	111849799	111849838	11824	0.075
chr9	112134038	112134077	11825	0.075
chr9	113251024	113251063	11826	0.075
chr9	113591729	113591768	11827	0.075
chr9	114583138	114583177	11828	0.075
chr9	115766476	115766515	11829	0.075
chr9	116011542	116011581	11830	0.075
chr9	116970497	116970536	11831	0.075
chr9	118189257	118189296	11832	0.075
chr9	118499937	118499976	11833	0.075
chr9	118844369	118844408	11834	0.075
chr9	119758243	119758282	11835	0.075
chr9	120472448	120472487	11836	0.075
chr9	122057715	122057754	11837	0.075
chr9	123128656	123128695	11838	0.075
chr9	123270992	123271031	11839	0.075
chr9	123660619	123660658	11840	0.075
chr9	124776801	124776840	11841	0.075
chr9	125173509	125173548	11842	0.075
chr9	125244499	125244538	11843	0.075
chr9	126391158	126391197	11844	0.075
chr9	126460513	126460552	11845	0.075
chr9	127632136	127632175	11846	0.075
chr9	127847382	127847421	11847	0.075
chr9	130026419	130026458	11848	0.075
chr9	130028161	130028200	11849	0.075
chr9	130635476	130635515	11850	0.075

chr9	130812247	130812286	11851	0.075
chr9	130815091	130815130	11852	0.075
chr9	131968718	131968757	11853	0.075
chr9	133126033	133126072	11854	0.075
chr9	133934623	133934662	11855	0.075
chr9	134511221	134511260	11856	0.075
chr9	134611834	134611873	11857	0.075
chr9	134623221	134623260	11858	0.075
chr9	134659470	134659509	11859	0.075
chr9	134701641	134701680	11860	0.075
chr9	134798143	134798182	11861	0.075
chr9	134856113	134856152	11862	0.075
chr9	135014059	135014098	11863	0.075
chr9	135885768	135885807	11864	0.075
chr9	136732856	136732895	11865	0.075
chr9	137302788	137302827	11866	0.075
chr9	138056196	138056235	11867	0.075
chr9	138231522	138231561	11868	0.075
chr9	138243360	138243399	11869	0.075
chr9	138281956	138281995	11870	0.075
chr9	138759648	138759687	11871	0.075
chr9	138989852	138989891	11872	0.075
chr9	139042903	139042942	11873	0.075
chr9	139178622	139178661	11874	0.075
chr9	139187490	139187529	11875	0.075
chr9	140130519	140130558	11876	0.075
chr9	14269455	14269494	11877	0.075
chr9	14428877	14428916	11878	0.075
chr9	15629952	15629991	11879	0.075
chr9	15671708	15671747	11880	0.075
chr9	17385130	17385169	11881	0.075
chr9	18656568	18656607	11882	0.075
chr9	18791137	18791176	11883	0.075
chr9	19546745	19546784	11884	0.075
chr9	19964393	19964432	11885	0.075
chr9	2071753	2071792	11886	0.075
chr9	21561305	21561344	11887	0.075
chr9	21967015	21967054	11888	0.075
chr9	22205447	22205486	11889	0.075
chr9	24894366	24894405	11890	0.075
chr9	28034801	28034840	11891	0.075
chr9	3055496	3055535	11892	0.075
chr9	31521241	31521280	11893	0.075
chr9	32083235	32083274	11894	0.075
chr9	32604521	32604560	11895	0.075
chr9	32823307	32823346	11896	0.075
chr9	33028692	33028731	11897	0.075
chr9	33114289	33114328	11898	0.075
chr9	33240526	33240565	11899	0.075
chr9	33450803	33450842	11900	0.075

chr9	33453084	33453123	11901	0.075
chr9	33786288	33786327	11902	0.075
chr9	34142114	34142153	11903	0.075
chr9	34448497	34448536	11904	0.075
chr9	34484259	34484298	11905	0.075
chr9	34511607	34511646	11906	0.075
chr9	34962408	34962447	11907	0.075
chr9	34979741	34979780	11908	0.075
chr9	35695790	35695829	11909	0.075
chr9	367082	367121	11910	0.075
chr9	36892609	36892648	11911	0.075
chr9	36903039	36903078	11912	0.075
chr9	37005051	37005090	11913	0.075
chr9	38335216	38335255	11914	0.075
chr9	38417244	38417283	11915	0.075
chr9	38518442	38518481	11916	0.075
chr9	38631829	38631868	11917	0.075
chr9	4028393	4028432	11918	0.075
chr9	4200285	4200324	11919	0.075
chr9	4712689	4712728	11920	0.075
chr9	4846834	4846873	11921	0.075
chr9	76422418	76422457	11922	0.075
chr9	77842864	77842903	11923	0.075
chr9	77993438	77993477	11924	0.075
chr9	78793017	78793056	11925	0.075
chr9	78927116	78927155	11926	0.075
chr9	80134320	80134359	11927	0.075
chr9	80173579	80173618	11928	0.075
chr9	81530932	81530971	11929	0.075
chr9	81999297	81999336	11930	0.075
chr9	83623993	83624032	11931	0.075
chr9	8455093	8455132	11932	0.075
chr9	84699829	84699868	11933	0.075
chr9	85779285	85779324	11934	0.075
chr9	85780963	85781002	11935	0.075
chr9	86663867	86663906	11936	0.075
chr9	867267	867306	11937	0.075
chr9	87394371	87394410	11938	0.075
chr9	88091190	88091229	11939	0.075
chr9	88949392	88949431	11940	0.075
chr9	89018626	89018665	11941	0.075
chr9	90809285	90809324	11942	0.075
chr9	9101775	9101814	11943	0.075
chr9	91297866	91297905	11944	0.075
chr9	92645632	92645671	11945	0.075
chr9	92795181	92795220	11946	0.075
chr9	93215923	93215962	11947	0.075
chr9	93979883	93979922	11948	0.075
chr9	94457017	94457056	11949	0.075
chr9	94519907	94519946	11950	0.075

chr9	95653884	95653923	11951	0.075
chr9	97970749	97970788	11952	0.075
chr9	98000317	98000356	11953	0.075
chr9	98987129	98987168	11954	0.075
chr9	99754744	99754783	11955	0.075
chrM	2749	2885	11956	0.116788321
chrX	129657917	129658009	11957	0.043010753
chrX	126061320	126061387	11958	0.044117647
chrX	32259703	32259787	11959	0.047058824
chrX	86659670	86659751	11960	0.048780488
chrX	100845751	100845788	11961	0.052631579
chrX	102008816	102008853	11962	0.052631579
chrX	103234830	103234867	11963	0.052631579
chrX	103483309	103483346	11964	0.052631579
chrX	103609333	103609370	11965	0.052631579
chrX	104085525	104085562	11966	0.052631579
chrX	105004796	105004833	11967	0.052631579
chrX	106152685	106152722	11968	0.052631579
chrX	108494507	108494544	11969	0.052631579
chrX	108993471	108993508	11970	0.052631579
chrX	109303253	109303290	11971	0.052631579
chrX	109566206	109566243	11972	0.052631579
chrX	109566917	109566954	11973	0.052631579
chrX	109832151	109832188	11974	0.052631579
chrX	110629870	110629907	11975	0.052631579
chrX	111135748	111135785	11976	0.052631579
chrX	111398216	111398253	11977	0.052631579
chrX	111819834	111819871	11978	0.052631579
chrX	111925927	111925964	11979	0.052631579
chrX	112099917	112099954	11980	0.052631579
chrX	112840978	112841015	11981	0.052631579
chrX	11332865	11332902	11982	0.052631579
chrX	114172865	114172902	11983	0.052631579
chrX	114298908	114298945	11984	0.052631579
chrX	115370762	115370799	11985	0.052631579
chrX	115371248	115371285	11986	0.052631579
chrX	116027605	116027642	11987	0.052631579
chrX	116126838	116126875	11988	0.052631579
chrX	11627983	11628020	11989	0.052631579
chrX	116902946	116902983	11990	0.052631579
chrX	117230821	117230858	11991	0.052631579
chrX	117355423	117355460	11992	0.052631579
chrX	117787632	117787669	11993	0.052631579
chrX	118211289	118211326	11994	0.052631579
chrX	118400616	118400653	11995	0.052631579
chrX	118421164	118421201	11996	0.052631579
chrX	118426945	118426982	11997	0.052631579
chrX	118693810	118693847	11998	0.052631579
chrX	11926975	11927012	11999	0.052631579
chrX	119858162	119858199	12000	0.052631579

chrX	120155044	120155081	12001	0.052631579
chrX	120155424	120155461	12002	0.052631579
chrX	120521710	120521747	12003	0.052631579
chrX	12180902	12180939	12004	0.052631579
chrX	12268930	12268967	12005	0.052631579
chrX	122760836	122760873	12006	0.052631579
chrX	122847059	122847096	12007	0.052631579
chrX	122847732	122847769	12008	0.052631579
chrX	123330316	123330353	12009	0.052631579
chrX	123596809	123596846	12010	0.052631579
chrX	12406029	12406066	12011	0.052631579
chrX	124168496	124168533	12012	0.052631579
chrX	12433184	12433221	12013	0.052631579
chrX	125054067	125054104	12014	0.052631579
chrX	125690802	125690839	12015	0.052631579
chrX	126577304	126577341	12016	0.052631579
chrX	127432732	127432769	12017	0.052631579
chrX	128607401	128607438	12018	0.052631579
chrX	128978886	128978923	12019	0.052631579
chrX	130485728	130485765	12020	0.052631579
chrX	131034770	131034807	12021	0.052631579
chrX	131549766	131549803	12022	0.052631579
chrX	131733790	131733827	12023	0.052631579
chrX	131796418	131796455	12024	0.052631579
chrX	132235019	132235056	12025	0.052631579
chrX	133506217	133506254	12026	0.052631579
chrX	133914406	133914443	12027	0.052631579
chrX	134509668	134509705	12028	0.052631579
chrX	135772269	135772306	12029	0.052631579
chrX	136422866	136422903	12030	0.052631579
chrX	13713095	13713132	12031	0.052631579
chrX	137464278	137464315	12032	0.052631579
chrX	137579810	137579847	12033	0.052631579
chrX	13801958	13801995	12034	0.052631579
chrX	138676156	138676193	12035	0.052631579
chrX	13955330	13955367	12036	0.052631579
chrX	139570975	139571012	12037	0.052631579
chrX	139571954	139571991	12038	0.052631579
chrX	139673800	139673837	12039	0.052631579
chrX	14023690	14023727	12040	0.052631579
chrX	140541458	140541495	12041	0.052631579
chrX	141600731	141600768	12042	0.052631579
chrX	144767176	144767213	12043	0.052631579
chrX	144793353	144793390	12044	0.052631579
chrX	145264527	145264564	12045	0.052631579
chrX	145265249	145265286	12046	0.052631579
chrX	146210343	146210380	12047	0.052631579
chrX	146824483	146824520	12048	0.052631579
chrX	146919856	146919893	12049	0.052631579
chrX	146920240	146920277	12050	0.052631579

chrX	148151633	148151670	12051	0.052631579
chrX	148151745	148151782	12052	0.052631579
chrX	149541558	149541595	12053	0.052631579
chrX	149690489	149690526	12054	0.052631579
chrX	150097240	150097277	12055	0.052631579
chrX	150335791	150335828	12056	0.052631579
chrX	15045500	15045537	12057	0.052631579
chrX	15070528	15070565	12058	0.052631579
chrX	15093466	15093503	12059	0.052631579
chrX	151108963	151109000	12060	0.052631579
chrX	151482137	151482174	12061	0.052631579
chrX	152138021	152138058	12062	0.052631579
chrX	152266150	152266187	12063	0.052631579
chrX	152384937	152384974	12064	0.052631579
chrX	15259745	15259782	12065	0.052631579
chrX	152661889	152661926	12066	0.052631579
chrX	152684742	152684779	12067	0.052631579
chrX	152740867	152740904	12068	0.052631579
chrX	152746788	152746825	12069	0.052631579
chrX	152943320	152943357	12070	0.052631579
chrX	153233833	153233870	12071	0.052631579
chrX	153235813	153235850	12072	0.052631579
chrX	153354765	153354802	12073	0.052631579
chrX	16434244	16434281	12074	0.052631579
chrX	16435056	16435093	12075	0.052631579
chrX	16671996	16672033	12076	0.052631579
chrX	17673508	17673545	12077	0.052631579
chrX	18563349	18563386	12078	0.052631579
chrX	19601893	19601930	12079	0.052631579
chrX	19605233	19605270	12080	0.052631579
chrX	19803982	19804019	12081	0.052631579
chrX	20825849	20825886	12082	0.052631579
chrX	22788382	22788419	12083	0.052631579
chrX	22940005	22940042	12084	0.052631579
chrX	22943749	22943786	12085	0.052631579
chrX	23248841	23248878	12086	0.052631579
chrX	24405834	24405871	12087	0.052631579
chrX	24406650	24406687	12088	0.052631579
chrX	24575300	24575337	12089	0.052631579
chrX	24584324	24584361	12090	0.052631579
chrX	26757051	26757088	12091	0.052631579
chrX	26973675	26973712	12092	0.052631579
chrX	27795279	27795316	12093	0.052631579
chrX	27858836	27858873	12094	0.052631579
chrX	28555500	28555537	12095	0.052631579
chrX	29555710	29555747	12096	0.052631579
chrX	29851055	29851092	12097	0.052631579
chrX	30938303	30938340	12098	0.052631579
chrX	31242614	31242651	12099	0.052631579
chrX	31273467	31273504	12100	0.052631579

chrX	31429712	31429749	12101	0.052631579
chrX	31894149	31894186	12102	0.052631579
chrX	33065334	33065371	12103	0.052631579
chrX	33065490	33065527	12104	0.052631579
chrX	33324986	33325023	12105	0.052631579
chrX	34290698	34290735	12106	0.052631579
chrX	35172751	35172788	12107	0.052631579
chrX	35868475	35868512	12108	0.052631579
chrX	36592470	36592507	12109	0.052631579
chrX	38817940	38817977	12110	0.052631579
chrX	38895077	38895114	12111	0.052631579
chrX	38959906	38959943	12112	0.052631579
chrX	39456774	39456811	12113	0.052631579
chrX	39866509	39866546	12114	0.052631579
chrX	39867193	39867230	12115	0.052631579
chrX	39913006	39913043	12116	0.052631579
chrX	40367590	40367627	12117	0.052631579
chrX	40396037	40396074	12118	0.052631579
chrX	40428718	40428755	12119	0.052631579
chrX	40915680	40915717	12120	0.052631579
chrX	41549268	41549305	12121	0.052631579
chrX	41574650	41574687	12122	0.052631579
chrX	41667139	41667176	12123	0.052631579
chrX	4345837 4345874	12124	0.052631579	
chrX	46359257	46359294	12125	0.052631579
chrX	46834177	46834214	12126	0.052631579
chrX	47329607	47329644	12127	0.052631579
chrX	48816400	48816437	12128	0.052631579
chrX	49022063	49022100	12129	0.052631579
chrX	49264144	49264181	12130	0.052631579
chrX	49341784	49341821	12131	0.052631579
chrX	49749735	49749772	12132	0.052631579
chrX	50749879	50749916	12133	0.052631579
chrX	50925125	50925162	12134	0.052631579
chrX	5097925 5097962	12135	0.052631579	
chrX	51085379	51085416	12136	0.052631579
chrX	51166516	51166553	12137	0.052631579
chrX	53753838	53753875	12138	0.052631579
chrX	54734683	54734720	12139	0.052631579
chrX	55226174	55226211	12140	0.052631579
chrX	5546251 5546288	12141	0.052631579	
chrX	5546403 5546440	12142	0.052631579	
chrX	55796766	55796803	12143	0.052631579
chrX	56272567	56272604	12144	0.052631579
chrX	56272738	56272775	12145	0.052631579
chrX	56333574	56333611	12146	0.052631579
chrX	56608509	56608546	12147	0.052631579
chrX	57834668	57834705	12148	0.052631579
chrX	62861255	62861292	12149	0.052631579
chrX	63056769	63056806	12150	0.052631579

chrX	63129703	63129740	12151	0.052631579
chrX	63231612	63231649	12152	0.052631579
chrX	63443093	63443130	12153	0.052631579
chrX	64641566	64641603	12154	0.052631579
chrX	64671230	64671267	12155	0.052631579
chrX	64866126	64866163	12156	0.052631579
chrX	65918709	65918746	12157	0.052631579
chrX	67058016	67058053	12158	0.052631579
chrX	68067741	68067778	12159	0.052631579
chrX	68298038	68298075	12160	0.052631579
chrX	68416251	68416288	12161	0.052631579
chrX	68562189	68562226	12162	0.052631579
chrX	68562609	68562646	12163	0.052631579
chrX	68707468	68707505	12164	0.052631579
chrX	68720086	68720123	12165	0.052631579
chrX	68720520	68720557	12166	0.052631579
chrX	69199233	69199270	12167	0.052631579
chrX	69555933	69555970	12168	0.052631579
chrX	70303844	70303881	12169	0.052631579
chrX	71164118	71164155	12170	0.052631579
chrX	71614295	71614332	12171	0.052631579
chrX	7509748	7509785	12172	0.052631579
chrX	7512303	7512340	12173	0.052631579
chrX	80406011	80406048	12174	0.052631579
chrX	80406759	80406796	12175	0.052631579
chrX	81849996	81850033	12176	0.052631579
chrX	82144677	82144714	12177	0.052631579
chrX	82724005	82724042	12178	0.052631579
chrX	83198627	83198664	12179	0.052631579
chrX	83365415	83365452	12180	0.052631579
chrX	84284331	84284368	12181	0.052631579
chrX	84319765	84319802	12182	0.052631579
chrX	85009520	85009557	12183	0.052631579
chrX	85363312	85363349	12184	0.052631579
chrX	86776880	86776917	12185	0.052631579
chrX	87708695	87708732	12186	0.052631579
chrX	88292502	88292539	12187	0.052631579
chrX	88347989	88348026	12188	0.052631579
chrX	90221244	90221281	12189	0.052631579
chrX	93591885	93591922	12190	0.052631579
chrX	9397163	9397200	12191	0.052631579
chrX	94499722	94499759	12192	0.052631579
chrX	94697942	94697979	12193	0.052631579
chrX	95521774	95521811	12194	0.052631579
chrX	95521888	95521925	12195	0.052631579
chrX	95646262	95646299	12196	0.052631579
chrX	96585880	96585917	12197	0.052631579
chrX	96741834	96741871	12198	0.052631579
chrX	97745489	97745526	12199	0.052631579
chrX	97815236	97815273	12200	0.052631579

chrX	98293865	98293902	12201	0.052631579
chrX	99134660	99134697	12202	0.052631579
chrX	99135429	99135466	12203	0.052631579
chrX	9986354	9986391	12204	0.052631579
chrX	100152263	100152302	12205	0.075
chrX	10244244	10244283	12206	0.075
chrX	102839792	102839831	12207	0.075
chrX	102923564	102923603	12208	0.075
chrX	102970719	102970758	12209	0.075
chrX	103102231	103102270	12210	0.075
chrX	103635875	103635914	12211	0.075
chrX	106419112	106419151	12212	0.075
chrX	106505447	106505486	12213	0.075
chrX	106691638	106691677	12214	0.075
chrX	107296187	107296226	12215	0.075
chrX	109330628	109330667	12216	0.075
chrX	110078721	110078760	12217	0.075
chrX	111797675	111797714	12218	0.075
chrX	11182991	11183030	12219	0.075
chrX	11295783	11295822	12220	0.075
chrX	11568804	11568843	12221	0.075
chrX	116152343	116152382	12222	0.075
chrX	11798458	11798497	12223	0.075
chrX	11921247	11921286	12224	0.075
chrX	119628551	119628590	12225	0.075
chrX	120351851	120351890	12226	0.075
chrX	122184738	122184777	12227	0.075
chrX	122441602	122441641	12228	0.075
chrX	125298234	125298273	12229	0.075
chrX	129953902	129953941	12230	0.075
chrX	131441508	131441547	12231	0.075
chrX	131635781	131635820	12232	0.075
chrX	132306451	132306490	12233	0.075
chrX	136551358	136551397	12234	0.075
chrX	137436074	137436113	12235	0.075
chrX	141531199	141531238	12236	0.075
chrX	147396580	147396619	12237	0.075
chrX	149159657	149159696	12238	0.075
chrX	150255887	150255926	12239	0.075
chrX	150558326	150558365	12240	0.075
chrX	151763743	151763782	12241	0.075
chrX	152780402	152780441	12242	0.075
chrX	153034126	153034165	12243	0.075
chrX	153247726	153247765	12244	0.075
chrX	16569314	16569353	12245	0.075
chrX	16647515	16647554	12246	0.075
chrX	16798343	16798382	12247	0.075
chrX	17653796	17653835	12248	0.075
chrX	18604954	18604993	12249	0.075
chrX	19704957	19704996	12250	0.075

chrX	19809733	19809772	12251	0.075
chrX	20479431	20479470	12252	0.075
chrX	22880152	22880191	12253	0.075
chrX	23792673	23792712	12254	0.075
chrX	23916628	23916667	12255	0.075
chrX	24825993	24826032	12256	0.075
chrX	28430145	28430184	12257	0.075
chrX	28479713	28479752	12258	0.075
chrX	30896927	30896966	12259	0.075
chrX	31708584	31708623	12260	0.075
chrX	34829942	34829981	12261	0.075
chrX	35346302	35346341	12262	0.075
chrX	38403029	38403068	12263	0.075
chrX	39266015	39266054	12264	0.075
chrX	39489614	39489653	12265	0.075
chrX	40576353	40576392	12266	0.075
chrX	41081529	41081568	12267	0.075
chrX	48267287	48267326	12268	0.075
chrX	48283091	48283130	12269	0.075
chrX	48497151	48497190	12270	0.075
chrX	48846406	48846445	12271	0.075
chrX	50230847	50230886	12272	0.075
chrX	5200929	5200968	12273	0.075
chrX	54404794	54404833	12274	0.075
chrX	54853320	54853359	12275	0.075
chrX	5557198	5557237	12276	0.075
chrX	57010917	57010956	12277	0.075
chrX	62970440	62970479	12278	0.075
chrX	64467568	64467607	12279	0.075
chrX	64805503	64805542	12280	0.075
chrX	68236851	68236890	12281	0.075
chrX	68367264	68367303	12282	0.075
chrX	69562247	69562286	12283	0.075
chrX	71189259	71189298	12284	0.075
chrX	7162870	7162909	12285	0.075
chrX	7224494	7224533	12286	0.075
chrX	72953399	72953438	12287	0.075
chrX	73670598	73670637	12288	0.075
chrX	7468736	7468775	12289	0.075
chrX	77799627	77799666	12290	0.075
chrX	77912318	77912357	12291	0.075
chrX	8768748	8768787	12292	0.075
chrX	94404804	94404843	12293	0.075
chrX	97499016	97499055	12294	0.075
chrX	98226042	98226081	12295	0.075
chrX	14423290	14423336	12296	0.085106383
chrY	11771710	11771797	12297	0.045454545
chrY	11922634	11922671	12298	0.052631579
chrY	12543805	12543842	12299	0.052631579
chrY	13461524	13461561	12300	0.052631579

chrY	13536412	13536449	12301	0.052631579
chrY	15275873	15275910	12302	0.052631579
chrY	15682917	15682954	12303	0.052631579
chrY	21159625	21159662	12304	0.052631579
chrY	4956943	4956980	12305	0.052631579
chrY	57377835	57377872	12306	0.052631579
chrY	10540071	10540110	12307	0.075
chrY	13348007	13348046	12308	0.075
chrY	14377763	14377802	12309	0.075
chrY	17918949	17918988	12310	0.075
chrY	8620833	8620872	12311	0.075
