# Supplementary material for: High-throughput sequencing identifies STAT3 as the DNA-associated factor for p53-NF-κB-complex-dependent gene expression in human heart failure
Source: Genome Med. 2010 Jun 14;2(6):37. doi: 10.1186/gm158 (PMC2905097; doi:10.1186/gm158)
Supplement: Additional file 11 — List of transcription factor motifs enriched in the subset of p53-RELA binding sites (total of 1,344 sites) containing the bona fide κB motif. [file gm158-S11.DOC]

**Additional file 11.** List of transcription factor motifs enriched in p53-RELA re-ChIP-sequencing using human cardiomyopathic samples.

| **Motif** | **p value** |
| --- | --- |
| c-Rel | 1.16 x 10^-73^ |
| Churchill | 2.20 x 10^-39^ |
| STAT3 | 3.42 x 10^-37^ |
| STAT6 | 3.45 x 10^-33^ |
| MZF_1-4 | 2.87 x 10^-31^ |
| STAT1 | 2.92 x 10^-31^ |
| E2F-1 | 1.97 x 10^-24^ |
| Zic3 | 3.69 x 10^-24^ |
| HMG | 1.20 x 10^-21^ |
| Dorsal_2 | 1.11 x 10^-18^ |
| NF-AT | 2.14 x 10^-17^ |
| SPI-1 | 4.10 x 10^-17^ |
| Ets | 2.77 x 10^-16^ |
| Ik-2 | 3.51 x 10^-16^ |
| NF-κB | 3.70 x 10^-16^ |
| MZF1 | 6.48 x 10^-15^ |
| Zic1 | 4.75 x 10^-13^ |
| STAT5A | 1.49 x 10^-12^ |
