# Supplementary material for: High-throughput sequencing identifies STAT3 as the DNA-associated factor for p53-NF-κB-complex-dependent gene expression in human heart failure
Source: Genome Med. 2010 Jun 14;2(6):37. doi: 10.1186/gm158 (PMC2905097; doi:10.1186/gm158)
Supplement: Additional file 12 — List of transcription factor motifs enriched in the previously published [22]genome-wide RELA binding sites dataset (PET2 and PET3 clusters). [file gm158-S12.DOC]

**Additional file 12.** List of transcription factor motifs enriched in the previously published (21) genome-wide RELA binding sites dataset (PET2 and 3 clusters).

| **Motif** | **p value** |
| --- | --- |
| AP2α | 0.0 |
| E2F-1 | 3.84 x 10^-316^ |
| AP-2γ | 6.72 x 10^-286^ |
| NF-κB | 1.96 x 10^-285^ |
| ZF5 | 2.57 x 10^-254^ |
| ETF | 4.38 x 10^-236^ |
| c-Rel | 5.48 x 10^-184^ |
| Sp1 | 1.20 x 10^-143^ |
| c-MycMax | 1.88 x 10^-115^ |
| Elk-1 | 1.32 x 10^-114^ |
| Spz1 | 3.54 x 10^-103^ |
| E2F-1DP1 | 4.90 x 10^-92^ |
| Hairy | 3.30 x 10^-91^ |
| Dorsal_2 | 2.51 x 10^-87^ |
| Pax-4 | 3.35 x 10^-77^ |
| Ahr-ARNT | 2.80 x 10^-72^ |
| p50 | 7.80 x 10^-66^ |
| E2F-4DP-2 | 1.66 x 10^-56^ |
